# Substrate Length Controls Atropopeptide Complexity and Triggers Excision-Driven Scaffold Hop to Diketopiperazine Alkaloids

**DOI:** 10.1021/jacs.6c12504

**Published:** 2026-10-12

**Authors:** Bin Tan, Ziheng Cui, Yuya Kakumu, Sandra J. Panczyk, Mai Ikeda, Dongyu Liu, Christine M. Pestalozzi, Julia A. Vorholt, Allison S. Walker, Kornelia Hardes, Martin Grininger, Eric J. N. Helfrich

**Affiliations:** † Institute for Molecular Bio Science, 9173 Goethe University Frankfurt, Max-von-Laue Strasse 9, 60438 Frankfurt am Main, Germany; ‡ Buchmann Institute for Molecular Life Science; Institute of Organic Chemistry and Chemical Biology, Goethe University Frankfurt, Max-von-Laue Strasse 9, 60438 Frankfurt am Main, Germany; § Institute of Microbiology, ETH Zurich, 8093 Zurich, Switzerland; ∥ Department of Chemistry, Vanderbilt University, Stevenson Center 7330, Nashville, Tennessee 37240, United States; ⊥ Department of Biological Sciences, Vanderbilt University, VU Station B, Box 35-1634, Nashville, Tennessee 37235, United States; # Department of Bioresources, Fraunhofer Institute for Molecular Biology and Applied Ecology, Ohlebergsweg 12, 35392 Giessen, Germany; ∇ Senckenberg Research Institute and Natural History Museum, Senckenberganlage 25, 60325 Frankfurt, Germany

## Abstract

Atropopeptides are a family of ribosomally synthesized and posttranslationally modified peptides (RiPPs) that share conserved cross-link-forming amino acids in their core peptides, yet adopt architectures ranging from simple monocyclic to complex polycyclic structures with intriguing three-dimensional shapes. These complex architectures arise from multiple C–C and C–N cross-links that are installed by a single cytochrome P450 enzyme, but the factors governing these divergent cross-linking patterns and resulting structural complexities have remained elusive. Recent studies on RiPP biosynthesis have revealed a form of substrate-controlled catalysis, in which the precursor sequence, rather than the modifying enzyme, dictates the reaction course. Here, we describe a distinct form of substrate-controlled catalysis in atropopeptide biosynthesis, demonstrating that core peptide length, rather than sequence, determines the cross-linking pattern installed by an atropopeptide-forming cytochrome P450. We further uncover an unusual proteolytic excision of an internal amino acid in mature atropopeptides, generating peptides that spontaneously convert into pyrroloindoline-containing diketopiperazines (DKPs) and thereby effecting a nonenzymatic scaffold-hop from atropopeptides to DKP-derived alkaloids, for which we provide mechanistic insight. Both the atropopeptide and the corresponding DKP exhibit antiviral activity, and their structural diversity can be further expanded through precursor engineering. Our work reveals substrate length as a key determinant of atropopeptide complexity, provides insights into the catalysis of a multicross-link-forming P450, and establishes an unprecedented route connecting atropopeptides to pyrroloindoline DKP alkaloids.

## Graphical abstract

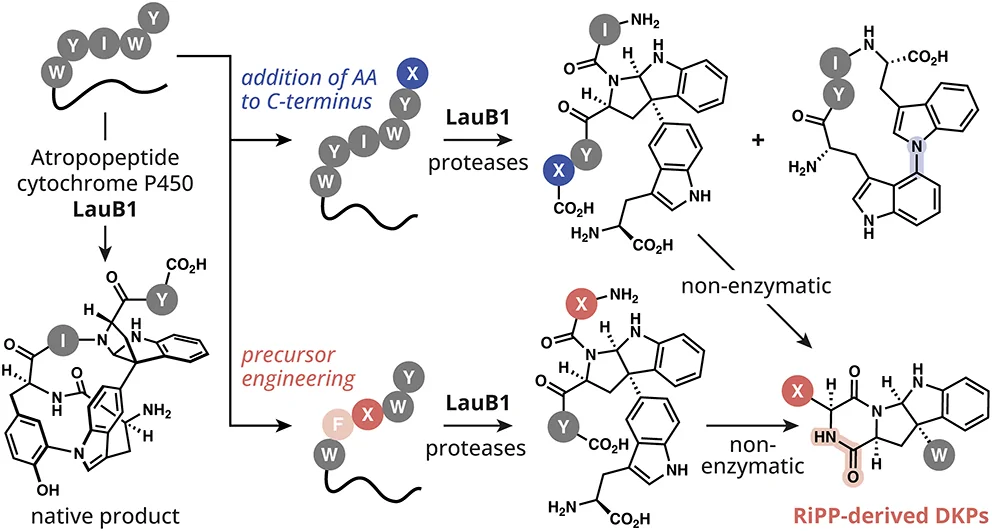

## Introduction

Enzymes are renowned for their remarkable regio-, chemo-, and stereoselectivity, which typically governs product formation.^1^ However, recent studies on the biosynthesis of ribosomally synthesized and post-translationally modified peptides (RiPPs), a large class of natural products derived from ribosomally translated peptides that undergo post-translational modifications, have uncovered an alternative model: substrate-controlled catalysis, in which the substrate rather than the enzyme primarily determines the reaction outcome.^2–6^ In these reactions, a single enzyme catalyzes distinct biochemical transformations depending on variations in the precursor peptide sequence. For instance, the radical S-adenosylmethionine (SAM) enzyme PasB involved in daropeptide biosynthesis introduces distinct cross-linking patterns in response to variations in the substrate sequence.^5^ Similar substrate-controlled transformations have also been observed during lanthipeptide biosynthesis.^3,4,6^ These findings expand our knowledge of enzymatic catalysis and provide new opportunities for the rational design of biosynthetic pathways to access structurally diverse bioactive molecules.

Atropopetides are a large RiPP family characterized by unique (poly-)­cyclic peptide architectures.^7^ Their biosynthetic gene clusters (BGCs) are highly conserved and typically consist of two genes: one encoding a precursor peptide and the other encoding a cytochrome P450 enzyme that installs between one and three cross-links in the core peptide. The mature atropopeptide is subsequently released by an as-yet-unknown protease^8^ (Figure 1A). Some clusters also contain genes encoding additional tailoring enzymes.^8^ Atropopeptide precursors share conserved cross-link-forming amino acids in the core peptide region. Despite this conservation in both BGC architectures and core peptide sequences, atropopeptides exhibit remarkable structural diversity, especially when it comes to the observed cross-linking patterns. For example, both tryptorubin B (**1**) and scabrirubin (**2**) contain Trp at positions 1 and 4 in their core peptides as cross-link-forming residues; **1** possesses three cross-links (two C–N and one C–C), whereas **2** harbors only a single C–N cross-link (Figure 1B).^8^ This divergence raises an intriguing question: Are atropopeptide cross-link patterns dictated primarily by the leader peptide, the core peptide, the associated P450 enzyme, or a combination of all elements?

**Figure 1:**
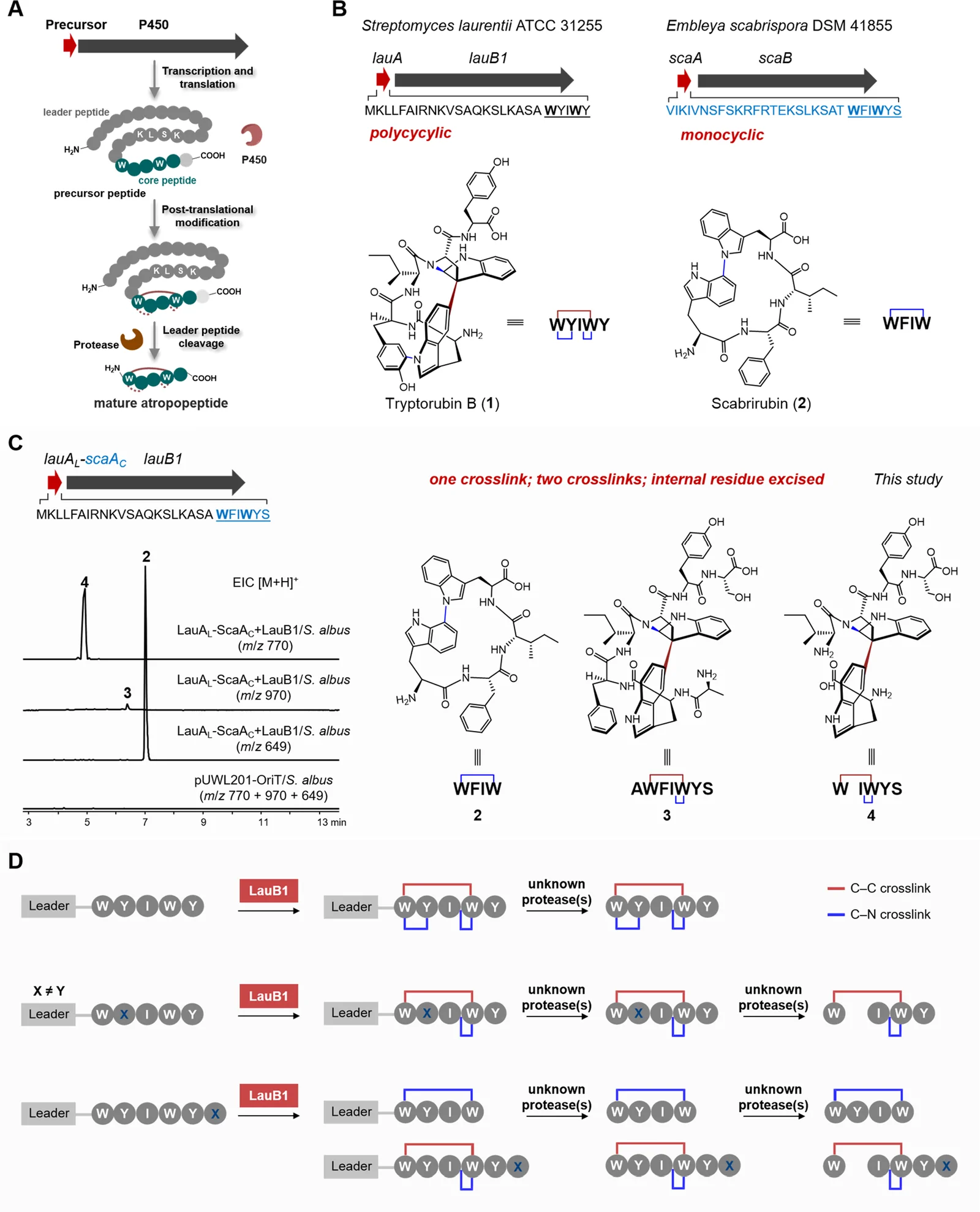
Native and engineered atropopeptide BGCs with their corresponding products. (A) General scheme of atropopeptide biosynthesis. The solid line between the two Trp residues indicates a C–C or C–N bond present in all atropopeptides; dashed lines indicate C–N bonds found only in a subset. (B) Native precursor peptide genes *lauA* and *scaA*, cytochrome P450 genes *lauB1* and *scaB*, and their corresponding products tryptorubin B (**1**) and scabrirubin (**2**). (C) Chimeric precursor *lauA*_*L*_ *-scaA*_*C*_ combined with *lauB1*, extracted ion chromatograms (EICs), and structures of products **2**, **3** and **4**. Core peptides are underlined. (D) Formation of native atropopeptides (top), derivatives lacking the second amino acid (middle) and atropopeptides with a single cross-link installed by LauB1 (bottom).

Recent combinatorial biosynthesis studies showed that coexpressing the cytochrome P450 ScaB (scabrirubin BGC) with various atropopeptide precursors, including LauA, the precursor of the polycyclic tryptorubin B and laurentirubin, consistently yielded monocyclic products containing only a single cross-link.^9^ This observation suggests that ScaB inherently dictates monocyclic product formation, independent of the core peptide sequence.^9^ However, it remains unclear whether the P450s that catalyze the formation of multiple cross-links, such as LauB1, function analogously, as coexpression of *lauB1* with *scaA*, the scabrirubin precursor gene, did not yield any products,^9^ indicating potential incompatibilities between the P450 LauB1 and its non-native precursor ScaA.

Here, we employed a chimeric precursor-based combinatorial biosynthesis strategy to elucidate the factors governing the formation of divergent atropopeptide cross-linking patterns. Unexpectedly, we found that core peptide length is a key determinant for the cross-linking patterns of the resulting products. Furthermore, we identified several atropopeptides, in which internal amino acids have been excised after maturation by the P450. Notably, these products can spontaneously transform into pyrroloindoline-containing diketopiperazines (DKPs), representing an unusual nonenzymatic scaffold-hopping route from RiPPs to DKP-derived alkaloids. Both the atropopeptide in which an internal amino acid has been excised and its derived DKP exhibit moderate antiviral activity. Moreover, precursor engineering further expands the structural diversity of these scaffolds. Overall, our study demonstrates that core peptide length modulates atropopeptide complexity and enables the generation of atropopeptide libraries that can be further converted into the corresponding DKP libraries.

## Results

### A Chimeric Precursor Enables ScaA Core Peptide Recognition by the P450 LauB1

Given that most RiPP tailoring enzymes recognize their substrate in a leader-dependent manner,^10^ the inability to detect any product upon coexpression of *lauB1* (P450 gene in the tryptorubin B/laurentirubin BGC) with *scaA* (the scabrirubin precursor gene)^9^ suggested that LauB1 might require its native leader peptide for substrate recognition. To investigate whether a P450 capable of introducing multiple cross-links can install this polycyclic pattern into a precursor that is naturally associated with a monocyclic scaffold, we designed a chimeric precursor, LauA_L_-ScaA_C_, in which the LauA leader peptide (LauA_L_) was fused to the ScaA core peptide (ScaA_C_, WFIWYS)­(Figure 1C). Coexpression of *lauB1* with the chimeric precursor resulted in the production of three atropopeptides. To determine their structures, a 10 L fermentation of *Streptomyces albus*/pUWL-LauA_L_-ScaA_C_+LauB1 was performed, enabling the isolation of compounds **2**–**4** (Figures 1C, S1). To our surprise, NMR analysis identified the main product **2** as scabrirubin^8^ (Figures 1C, S2–S4), indicating that LauB1, which natively generates a multicyclic product, cyclized the chimeric precursor into a monocyclic tetrapeptide featuring a single C–N biaryl cross-link, which is the native product of the P450 ScaB.^8^ Compound **3** was identified as the heptapeptide AWFIWYS with one C–C cross-link between the two Trp residues and one C–N linkage between the side chain of a Trp and its own amide nitrogen (Figures 1C, S5–S11). When compared with the cross-linking pattern of tryptorubin B (**1**), **3** lacks the C–N cross-link between Trp and Phe. Compound **4** was characterized as a pentapeptide W–IWYS that differed from **3** by the absence of the Ala and Phe residues (− indicates the excision of F) (Figures 1C, S12–S18). These findings demonstrate that the P450 LauB1 modifies the ScaA core peptide (WFIWYS) when paired with its native leader peptide, generating three products whose cross-linking patterns differ from that of its native product tryptorubin B: a monocyclic tetrapeptide with a C–N biaryl cross-link, a heptapeptide lacking a C–N cross-link and a pentapeptide in which Phe has been excised (Figure 1C).

### Core Peptide Length Dictates Atropopeptide Cross-Linking Patterns

The combination of LauB1 with the chimeric precursor LauA_L_-ScaA_C_ (WFIWYS) generated three unusual products. Notably, compound **4**, which lacks the internal Phe at position 2 of the core peptide, and the major product **2**, which contains a single C–N biaryl cross-link, prompted us to investigate how this chimeric precursor dictates the formation of these products. Compared to LauA (WYIWY), the chimeric substrate LauA_L_-ScaA_C_ (W**F**IWY**S**) differs by only two residues: an additional C-terminal Ser and a Phe replacing Tyr at the second position of the core peptide. To investigate which amino acid influences product formation, we designed two precursor mutants based on LauA: LauA-6S (WYIWY**S**), in which a single amino acid (Ser) was added to the C-terminus of LauA, and LauA-Y2F (W**F**IWY), in which the original core peptide length was maintained but the second core amino acid of LauA was substituted with Phe (Figure 2). These two engineered precursor genes were coexpressed with *lauB1*, respectively.

**Figure 2:**
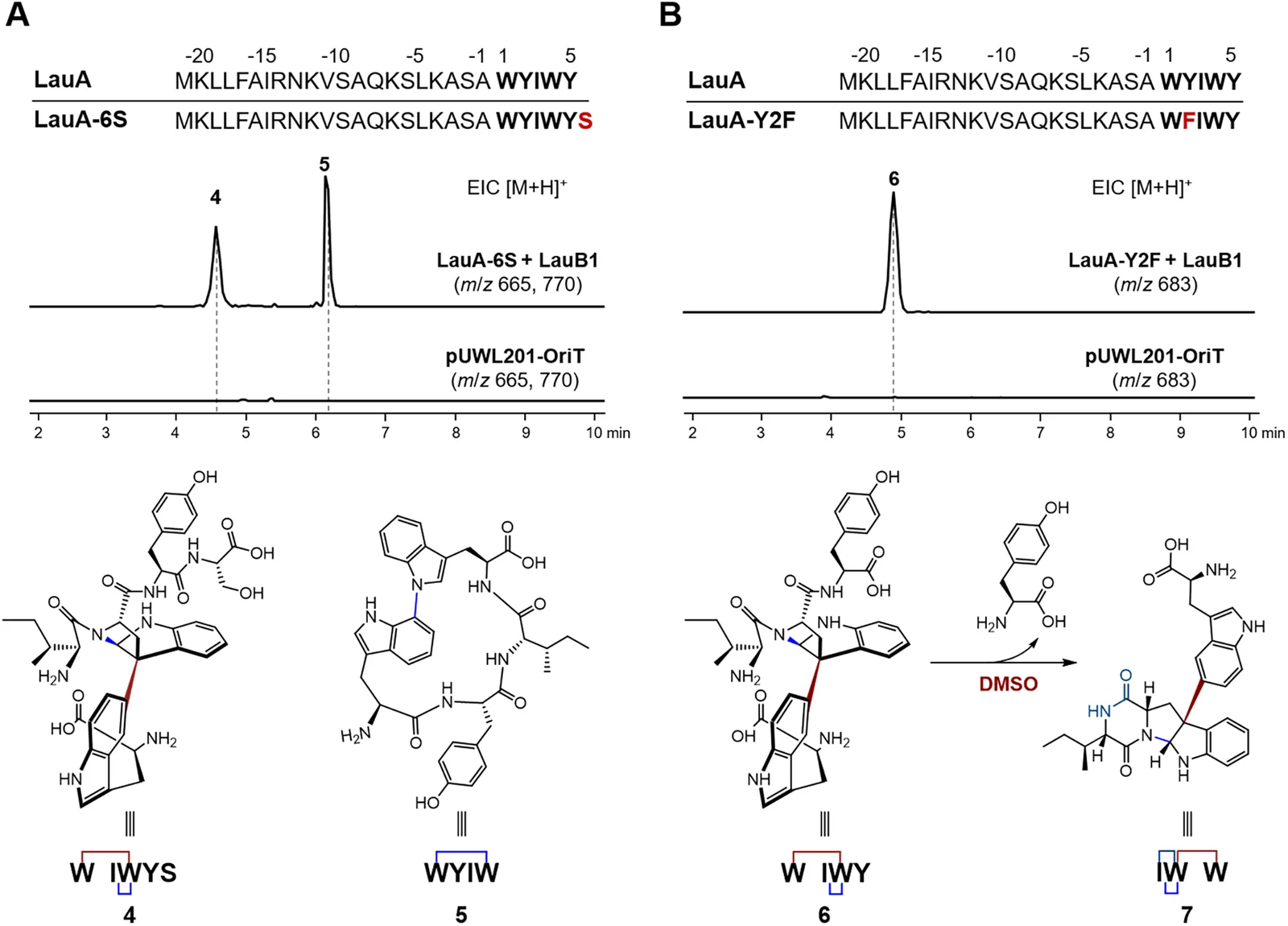
Combinatorial biosynthesis of mutated precursor peptides with the cytochrome P450 LauB1. (A) Combination of the mutated precursor LauA-6S with P450 LauB1 and its corresponding products **4** and **5**; (B) Combination of the mutated precursor LauA-Y2F with P450 LauB1 and its corresponding product **6**. **6** was spontaneously transformed into **7** in DMSO.

The combination of LauA-6S (WYIWY**S**) and LauB1 resulted in the generation of **4** (minor product) and **5** (major product) (Figures 2A, S19). 1D and 2D NMR data revealed that **5** contains a C–N biaryl cross-link, consistent with the structure of scabrirubin CB-1, that we previously obtained from the coexpression of *scaB* (scabrirubin P450-encoding gene) and *lauA* (tryptorubin B precursor gene) (Figures S20–S22).^9^ This finding indicates that the addition of a single Ser to the C-terminus of the native precursor peptide LauA alters the cross-linking pattern introduced by the P450 LauB1 from three cross-links (one C–C and two C–N) to a single C–N biaryl bond.

To investigate whether this cross-linking pattern was specific to Ser or can also be obtained when Ser is replaced by other amino acids, we performed site-directed mutagenesis studies to append various individual amino acids to the C-terminus of LauA. Remarkably, the addition of any selected amino acid, including Ala, Glu, Val, Tyr, Thr, Arg, Asn, His, Cys, or Gly, resulted in the formation of compound **5**, and the corresponding products with the second amino acid excised (Figures S23–S33). These results demonstrate that the introduction of an extra amino acid at the C-terminus of LauA, regardless of its identity, is sufficient to reprogram LauB1 to install a single C–N biaryl cross-link. This observation suggests that core peptide length plays a key role in determining the cross-linking pattern formed by LauB1 (Figure 1D).

### Nonenzymatic Transformation of Atropopeptides into Diketopiperazine-Alkaloids

Coexpression of LauA-Y2F (W**F**IWY) with LauB1 resulted in the production of a single product, compound **6** (*m*/*z* 683.3188 [M + H]^+^, Figures 2B, S34), whose mass suggested a modified tetrapeptide W–IWY, lacking Phe at position 2 (core peptide WFIWY, calc. 814. 3922 *m*/*z* [M + H]^+^). To investigate its cross-linking pattern, a 10 L fermentation of *S. albus*/pUWL-LauA-Y2F+LauB1 was performed, and compound **6** was isolated from the corresponding extract and purified to homogeneity. Initial dissolution of **6** in deuterated DMSO for NMR analysis resulted in its gradual conversion into compound **7** (*m*/*z* 502.2446 [M + H]^+^, Figure S35) and Tyr which precipitated in the NMR tube (Figure S36). Surprisingly, **7** was isolated and structurally characterized by NMR as a pyrroloindoline-containing diketopiperazine (DKP) (Figures 2B and S37–S43), indicating that **6** spontaneously loses its terminal Tyr in DMSO to yield the DKP-alkaloid **7** (Figure 2B). Stability studies showed that **6** was stable in water and exhibited minor transformation in methanol but underwent substantial conversion in DMSO within 48 h (Figure S44). The structure of **6** was subsequently determined by NMR in deuterated methanol, which revealed that **6** shares the same modifications as **4** but lacks the C-terminal Ser residue (Figures 2B, S45–S51).

### Mechanistic Insights into Diketopiperazine Formation

The unexpected conversion of **6** into the DKP **7** in DMSO prompted us to investigate the underlying mechanism. We hypothesized that the DKP moiety of **7** is formed through nucleophilic addition of the primary amine of Ile to the Tyr carbonyl, accompanied by the excision of the C-terminal Tyr (Figure 3). The rigid pyrroloindoline substructure of **6** likely constrains the conformational flexibility of Ile and Tyr in DMSO, preorganizing the reaction sites in close proximity. Molecular dynamics simulations of **6** in DMSO showed that conformations of **6** were geometrically accessible for transannular amidation (Figure S52).

**Figure 3:**
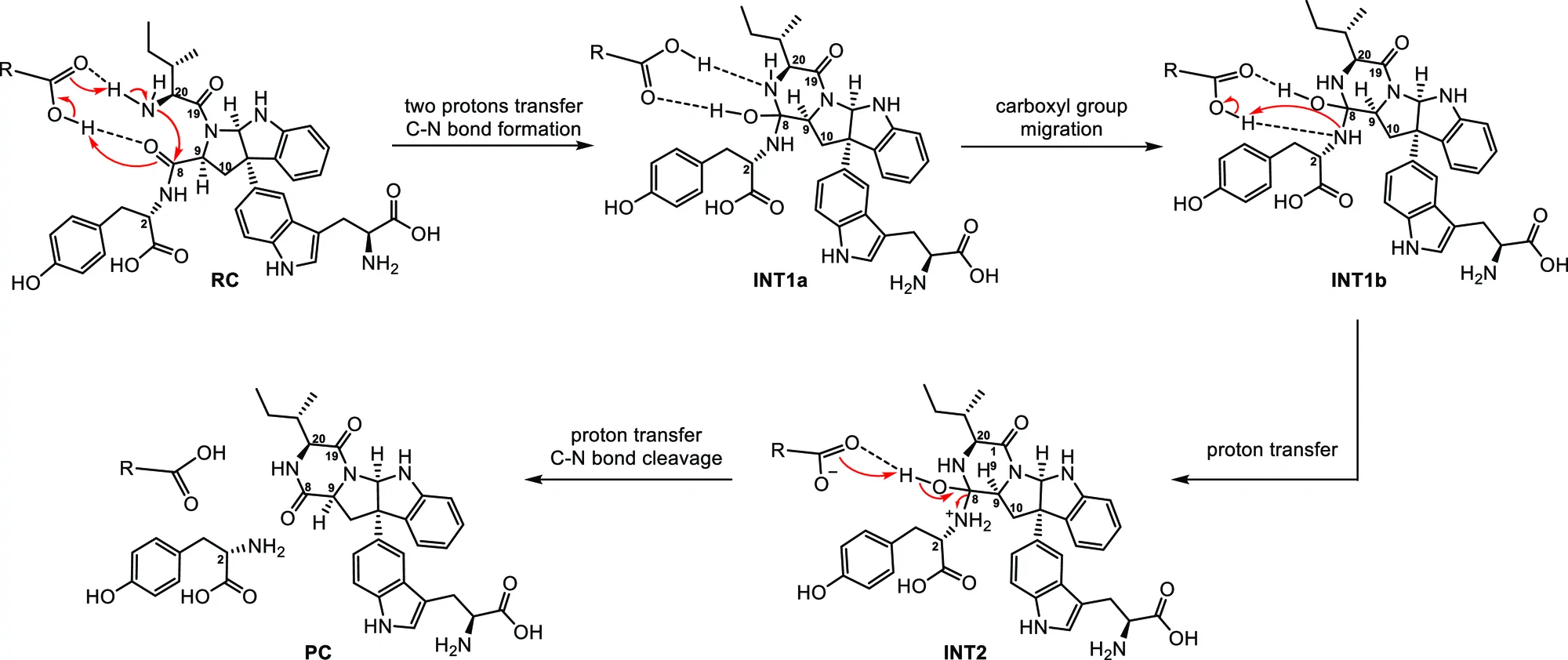
Scheme for the proposed diketopiperazine formation.

Since DKP formation proceeds in DMSO without catalyst supplementation, we proposed that a carboxyl group of another atropopeptide molecule could act as a proton shuttle to facilitate the transannular amidation. Incubation of **6** in DMSO containing 0.5% acetic acid significantly enhanced the conversion of **6** into **7** (Figure S53), supporting the potential involvement of a carboxyl group in this transformation. Density functional theory (DFT) calculations support the proposed role of the carboxyl group of a neighboring atropopeptide molecule as both proton donor and acceptor during nucleophilic addition and C–N bond cleavage, leading to DKP formation (Figure S54). Specifically, initial nucleophilic addition of NH_2_-20 to the carbonyl C-8 affords the hydroxyaminal **INT1a** (Figure 3). Subsequent conformational rearrangement of the carboxylic acid affords **INT1b**, followed by proton transfer from the carboxyl group to NH-2 (**INT2**). Finally, proton transfer coupled with cleavage of the C–N bond to release the C-terminal Tyr results in the formation of the DKP scaffold (Figure 3).

### Investigating the Molecular Basis for Internal Amino Acid Excision

The absence of the internal Phe residue in **6** from the coproduction of LauA-Y2F (WFIWY) and LauB1 prompted us to investigate the mechanism of the excision of the internal amino acid. Given that coexpression of LauA (WYIWY) with LauB1 does not yield the product lacking the internal amino acid, we thus hypothesized that the Tyr at the second position of the LauA core peptide (WYIWY) is required for C–N cross-link formation with the first Trp.

We predicted that substitutions of this residue with other amino acids, such as Phe, would prevent C–N bond formation, rendering the peptide susceptible to cleavage at the second position. To test this hypothesis, we performed extensive site-directed mutagenesis experiments, replacing Tyr at position 2 of the LauA core peptide (WYIWY) with His, Pro, Trp, Ala, Glu, Thr, Val, or Arg. LC-MS analysis revealed that all variants except the Tyr-to-Pro substitution, produced the same product that lacks the second amino acid (Figures 4A, S55–S63). The Pro-substituted mutant yielded a product (*m*/*z* 762 [M + H]^+^, Figure S63) consistent with a pentapeptide with a 2 Da mass loss (WPIWY, calc. *m*/*z* 764 [M + H]^+^), indicating that Pro was not excised. To determine whether the excision is mediated by the cytochrome P450 LauB1, we conducted a feeding experiment in which compound **3** was added to cultures of *S. albus*, *S. albus* expressing *lauB1* and TSB medium as a control. **3** was converted to **4** in both cultures of the *S. albus* WT and *S. albus* expressing *lauB1* but showed no conversion in TSB medium (Figure 4B). Furthermore, crude enzyme extracts from *S. albus* but not heat-inactivated crude enzyme extracts or buffer control catalyzed the conversion of **3** to **4** (Figure S64). These results demonstrate that the excision is catalyzed by a host-derived protease rather than by LauB1.

**Figure 4:**
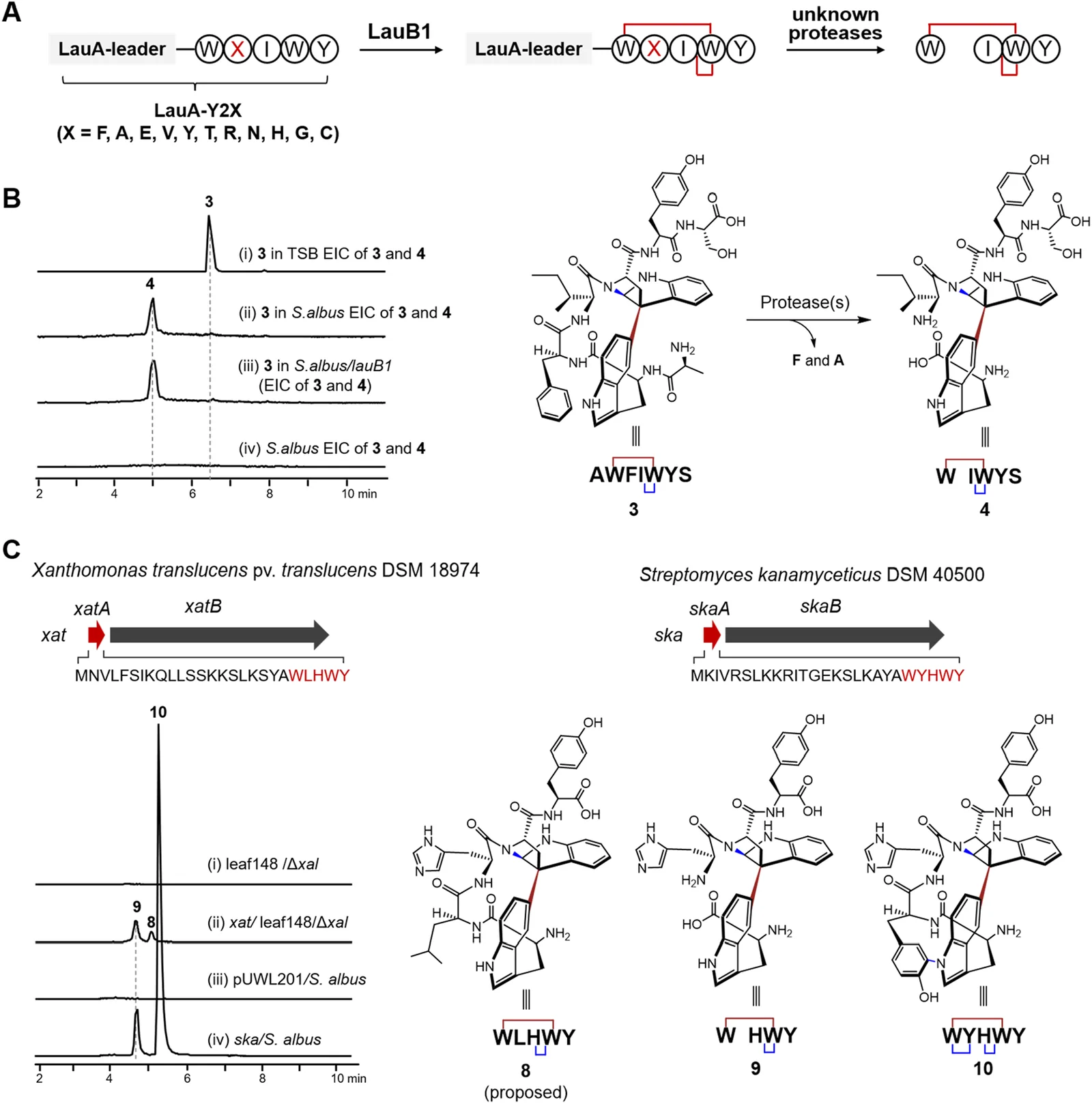
Excision of the second core-peptide amino acid in atropopeptides. (A) Proposed model for the formation of atropopeptides that lack the second amino acid from the LauA-Y23X mutant series. (B) Conversion of **3** upon feeding to the cultures of *S. albus*, *S. albus* expressing *lauB1* and TSB medium. (C) Heterologous expression of *the xat* and *ska* BGCs and their corresponding products.

### Genome Mining-Guided Discovery of Atropopeptides in Which an Internal Amino Acid is Excised During the Maturation Process

To further verify our hypothesis that the second core peptide residue is excised when it is not a Tyr, we re-examined all atropopeptide precursor sequences identified by AtropoFinder, a machine learning-based genome mining algorithm recently developed in our lab for the identification of atropopeptide BGCs,^8^ and found several precursors featuring alternative amino acids at this position. We selected the *xat* BGC from *Xanthomonas translucens* pv. translucens DSM 18974, whose core peptide sequence is WLHWY. Because the wild type strain did not produce detectable atropopeptides, we expressed this BGC in *Xanthomonas* sp. leaf 148 that was previously reported to produce atropopeptides.^11^ To eliminate interference from the native atropopeptide BGC (*xal*), *xal* was deleted to generate the *Xanthomonas* sp. leaf 148/Δ*xal* mutant. Heterologous expression of the *xat* BGC in *Xanthomonas* sp. leaf 148/Δ*xal* yielded two products: a major product (*m*/*z* 354 [M+2H]^2+^) and a minor product (*m*/*z* 401.5 [M+2H]^2+^) (Figures 4C and S65). The minor product **8** corresponds to the pentapeptide WLHWY with a 2 Da mass loss (calc. *m*/*z* 804 [M + H]^+^, Figure S65), whereas the major product was identified as the tetrapeptide **9** (W–HWY, Figure 4C) in which Leu at position 2 has been excised (calc. *m*/*z* 707 [M + H]^+^) (Figure S65). The identity of the major product from the *xat* BGC was confirmed by comparison with an authentic standard of **9** obtained from the heterologous expression of the *ska* BGC, an atropopeptide BGC encoding the precursor peptide with the core peptide sequence WYHWY (Figures 4C, S66–S67). The expression of *ska* BGC also yields the known compound **10**.^12^ Compound **9** has not been previously reported, and both **9** and **10** are fully characterized in the Supporting Information (Figures S68–S77). Expression of the native *xat* BGC yielded **9**, in which the Leu residue at position 2 had been excised. This result further supports our hypothesis that the second amino acid of the core-peptide may be excised during atropopeptide formation when it is not Tyr (Figure 1D). We next investigated whether this unusual internal residue excision also occurs in the native producers. Incubation of **3** with crude enzyme extracts from *X.* *translucens* pv. translucens, which contains the *xat* BGC, or *Streptomyces kanamyceticus*, which contains the *ska* BGC, resulted in the excision of the second core amino acid (Figures S78–S81). These results suggest that atropopeptides in which the second amino acid of the core peptide has been excised may also be generated in the native producers.

Notably, even when Tyr occupies the second position of the core peptide, the corresponding BGC may generate an atropopeptide in which the C–N bond between Tyr and Trp has not been installed. This product can subsequently undergo proteolytic excision of the second amino acid, thereby yielding an atropopeptide in which the internal Tyr residue has been excised, as observed for the *ska* BGC. These findings provide valuable insight into the order of cross-link formation during polycyclic atropopeptide biosynthesis. Specifically, the C–C bond between the two Trp residues and the C–N bond between Trp and the amide nitrogen are likely formed first, followed by late-stage C–N bond formation between the N-terminal Trp and Tyr.

### Precursor Engineering to Generate Atropopeptide Libraries That Can be Converted into DKP Libraries

To investigate whether other atropopeptides that lack the second amino acid can also be converted into the corresponding DKPs, compounds **4** (W–IWYS) and **9** (W–HWY) were incubated in DMSO. Similar to the transformation of **6** into DKP **7**, **4** was transformed into **7**, accompanied by the release of the C-terminal Tyr-Ser dipeptide (Figure S82), indicating the length of the C-terminal peptide did not affect the intramolecular nucleophilic addition. Compound **9** was converted into **11** (*m*/*z* 526 [M + H]^+^) (Figure S83). **11** showed a mass loss of 181 Da compared to **9**, consistent with the loss of one Tyr residue and the formation of a new amide bond. Accordingly, **11** was proposed to be a DKP containing a histidine residue. In both cases, the transformation efficiency was also significantly enhanced by the addition of 0.5% acetic acid (Figures S82 and S83).

To explore strategies for expanding the structural diversity of these atropopeptide derivatives and related DKPs, two sets of site-directed mutagenesis experiments were performed using the mutated precursor LauA-Y2F (WFIWY) as template. Amino acids were randomly selected to replace the third and fifth amino acid of the core peptide, respectively. Specifically, LauA-Y2F-3I (third amino acid) was mutated to generate the LauA-Y2F–I3X mutant series (X = H, R, Q, E, L, T, C, P, A) (Figure 5A) and LauA-Y2F-5Y was mutated to create the LauA-Y2F–Y5X mutant series (X = H, F, P, W, R, Q, E, L, T, C, A) (Figure 5B). The results showed that among the LauA-Y2F–I3X series, all variants produced the corresponding atropopeptides W–**X**WY, in which the second amino acid has been excised and different amino acids were incorporated at the third position (Figures 5A, S84–S92), except for LauA-Y2F–I3C (no product formation) and LauA-Y2F–I3P, which resulted in the production of a single product (*m*/*z* 867 [M + H]^+^), consistent with a hexapeptide with a 2 Da mass loss (AWFPWY, *m*/*z* 869 [M + H]^+^) (Figure S92). The LauA-Y2F–Y5X mutant series yielded atropopeptides W–IW**X** with the second amino acid excised and diverse C-terminal residues (Figures 5B, S93–S100). Together, both mutant series significantly expand the structural diversity of the atropopeptides from which an internal amino acid has been excised.

**Figure 5:**
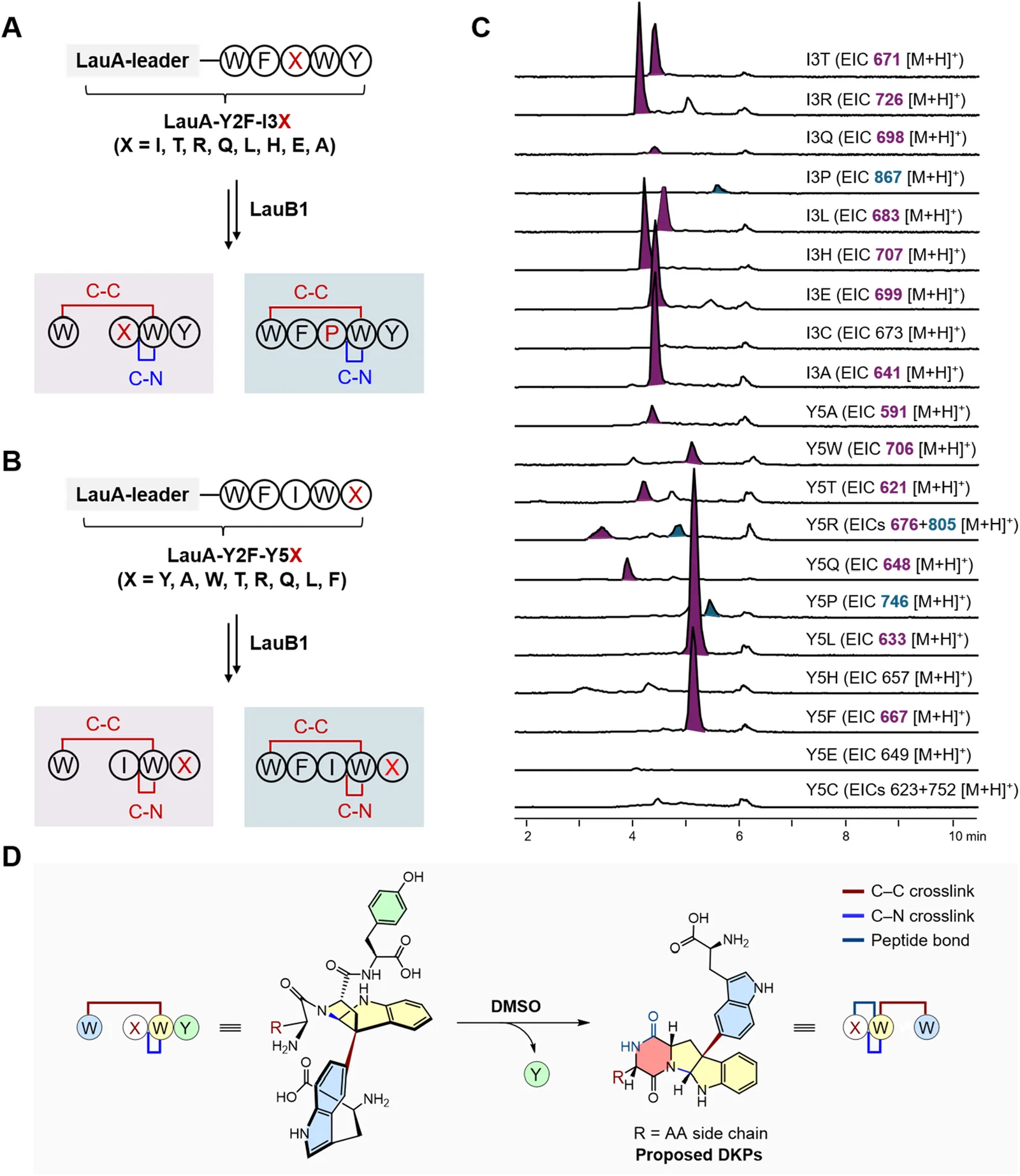
Precursor engineering results in the formation of diverse atropopeptides and atropopeptide-derived diketopiperazines. (A) The LauA-Y2F–I3X mutant series and their corresponding atropopeptide products with structures proposed based on MS/MS analysis. (B) The LauA-Y2F–Y5X mutant series and their corresponding atropopeptide products with structures proposed based on MS/MS analysis. (C) Extracted ion chromatograms (EICs) of products generated from the coexpression of *lauA-Y2F–I3X* with *lauB1* and *lauA-Y2F–Y5X* with *lauB1*. (D) Proposed conversion of atropopeptides into the corresponding diketopiperazines.

Resuspension of crude extracts containing atropopeptides that had undergone excision of the second core amino acid in DMSO consistently resulted in the formation of the corresponding DKPs (Figure S101). These results highlight the potential of precursor engineering to generate atropopeptide libraries, which can be transformed into the corresponding DKP libraries (Figure 5D).

### Bioactivity of the Atropopeptide **6** and Its Corresponding Diketopiperazine **7**

We evaluated **6** and its corresponding DKP **7** for their antimicrobial activities against all ESKAPE pathogens. No antibacterial activities were observed in agar diffusion assays. Given the moderate antiviral activity previously reported for scabrirubins,^9^ we next assessed their antiviral potential. Compounds **6** and **7** did not show cytotoxicity toward MDCK II cells at the concentration used (Figure S102A), and both compounds exhibited moderate antiviral activity against the Influenza virus subtype H1N1pdm, with **6** showing slightly higher efficacy than **7** (Figure S102B).

## Discussion and Conclusion

Atropopetide BGCs share a common architecture and their encoded precursor peptides contain conserved residues involved in cross-link formation. Nevertheless, the resulting atropopeptides display distinct cross-linking patterns. Our study unveiled that core peptide length determines the cross-linking pattern of atropopeptides modified by the multicross-link-forming P450 LauB1. Specifically, the addition of a single amino acid to the C-terminus of LauA changes the cross-linking pattern introduced by LauB1 from a polycyclic peptide with three cross-links (two C–N bonds and one C–C bond) to a monocyclic peptide containing a single C–N biaryl cross-link. Notably, this single C–N biaryl cross-link corresponds to that of the native product of ScaB, a P450 located close to the root position of the phylogenetic tree of atropopeptide-modifying P450s.^9^ These findings suggest that more recently evolved members of atropopeptide-modifying P450s, such as LauB1, have acquired the ability to install multiple cross-links while still retaining ancestral catalytic activity, for instance, when acting on a core peptide extended by a single amino acid residue. AlphaFold2-Multimer models of LauB1-LauA, LauB1-Y2F, LauB1–6S and ScaB-ScaA complexes suggest that the addition of a single residue at the C-terminus of LauA alters the binding mode of the precursor peptide. Specifically, the predicted placement of the LauA-6S core peptide within LauB1 differs markedly from that of LauA or LauA-Y2F. Compared with these precursors, the LauA-6S core peptide adopts an orientation more closely resembling that observed in the modeled ScaB-ScaA complex that is associated with the formation of a single cross-link (Figure S103). This model provides a possible structural rationale for the formation of the products containing a single C–N biaryl cross-link. However, it only represents a static model and does not fully capture the dynamic behavior of the precursor within the active site of the enzyme. Further structural and computational studies will be required to elucidate the molecular mechanism by which substrate length influences the formation of atropopeptides with single or multiple-cross-links.

Several examples of substrate sequence-controlled enzymatic catalysis have been reported in other RiPP families, including lanthionine synthetase in lanthipeptide biosynthesis,^3,4,6,13^ radical SAM enzymes in daropeptide biosynthesis,^5^ and most recently, a cytochrome P450 in aminopyruvatide biosynthesis.^14^ In these cases, the precursor sequence was shown to play a key role in determining the regioselectivity or^4,6^ stereoselectivity,^3,13^ of RiPP tailoring enzymes or the modification patterns they generate.^5,14^ In contrast to these studies, our work uncovers a distinct mode of substrate-controlled catalysis, in which substrate length, rather than substrate sequence, tunes enzymatic catalysis.

Pyrroloindoline-containing diketopiperazines (DKPs) are important indole alkaloids, featuring a pyrroloindoline moiety fused to a DKP skeleton. Members of this family exhibit a broad range of bioactivities, such as antimicrobial,^15–18^ antiviral,^16,19^ antitumor,^20,21^ and neuroprotective activities.^22,23^ The biosynthesis of pyrroloindoline-containing DKPs typically begins with the assembly of the DKP core by nonribosomal peptide synthetases (NRPSs) or cyclodipeptide synthases (CDPSs).^24^ The preformed DKP is then modified by tailoring enzymes that catalyze C3 hydroxylation,^25–27^ methylation,^28–30^ prenylation^31–34^ or radical-mediated reactions on the indole ring.^22,35–37^ These transformations result in the formation of a highly reactive iminium ion intermediate, which is then intramolecularly trapped by the adjacent amide nitrogen to form the pyrroloindoline alkaloid framework. In contrast to these canonical enzyme-mediated pathways, in which the pyrroloindoline moiety is assembled after DKP formation, we report an unprecedented, nonenzymatic route to pyrroloindoline-containing DKPs. In the atropopeptides that lack the second amino acid, the rigid, pre-existing pyrroloindoline structure positions the N-terminal amino nitrogen and the amide carbonyl carbon in close proximity. This conformational constraint enables the spontaneous intramolecular cyclization to form the DKP ring, representing an unprecedented route to pyrroloindoline-containing DKP-alkaloids. To the best of our knowledge, these atropopeptide derived pyrroloindoline-containing DKP-alkaloids represent the first reported RiPP-derived compounds containing a 2,5-diketopiperazine (DKP) scaffold. Other piperazine-containing RiPPs have been described, including klebsazolicin^38,39^ and streptamidine,^40^ both of which contain six-membered cyclic amidine motifs that are structurally related to, but distinct from, the DKP ring system.

A similar spontaneous DKP formation has been observed for synthetic peptides featuring a tetrahydroisoquinoline-3-carboxylic acid (Tic) scaffold in DMSO.^41,42^ The conformational rigidity of the Tic moiety has been proposed to facilitate the transannular amidation to form the corresponding DKP.^42^ Similarly, the pyrroloindoline moiety in **6** provides analogous conformational constraint, offering a plausible structural basis for the observed DKP formation. DFT calculations indicate that a second atropopeptide molecule assists this intramolecular cyclization. This mechanism is analogous to the carboxylic acid-mediated intramolecular aminolysis that leads to DKP byproduct formation in solid-phase peptide synthesis.^43^

The first atropopeptide lacking the second amino acid was discovered during the combinatorial expression of genes encoding the chimeric precursors and the P450 LauB1, in which the second core peptide residue was replaced by Phe. Site-directed mutagenesis of the second residue, along with heterologous expression of a native atropopeptide BGC that naturally lacks Tyr at this position consistently produced peptides with the second amino acid excised. These results demonstrate that Tyr at the second core peptide position is essential for C–N bond formation between the first two residues. These findings enable prediction of whether the second core amino acid will be retained or excised during atropopeptide maturation—and thus allow partial structural prediction of uncharacterized atropopeptides—based on their core peptide sequences.

Cyclic peptides are generally known for their proteolytic stability owing to their conformational rigidity.^44,45^ Internal residue excision in cyclic RiPPs and nonribosomal peptides is rarely observed. Examples include pyrroloquinoline quinone (PQQ), where a metalloendopeptidase encoded within the *pqq* BGC cleaves three internal amino acids following Glu-Tyr cross-link formation;^46,47^ mobilipeptins, where internal residues within the tetracyclic peptide moiety are cleaved by an as-of-yet-unknown mechanism;^48^ and archaeal lanthipeptides such as medlan A1, larlans A2/A5, and archalan β3, where amino acids within the thioether ring are hydrolyzed by unknown proteases.^49^ The excision of an internal amino acid in atropopeptides is particularly intriguing as cleavage occurs only in pyrroloindoline-containing frameworks lacking the C–N bond between the first and second amino acid while retaining a cyclic structure with two additional cross-links. Identifying the protease that specifically recognizes and cleaves such atropopeptides is currently underway.

In summary, our study reveals that atropopeptide cross-linking patterns are dictated by core peptide length. In addition, we identified multiple atropopeptides lacking the second amino acid from native and engineered atropopeptide BGCs. Remarkably, these products can spontaneously morph into pyrroloindoline-containing DKPs, representing an unprecedented nonenzymatic scaffold-hopping pathway connecting RiPPs to DKP alkaloids. Both the atropopeptide and the corresponding DKP exhibit moderate antiviral activity, and their chemical diversity can be further expanded through precursor engineering. These findings establish a platform for generating atropopeptide libraries that can be converted to DKP-alkaloid libraries.

## Supplementary Material



## Notes

Materials and methods, experimental details, structural elucidation of isolated compounds; LC-HRMS and MS/MS spectra; NMR spectra (Figures S1–S103, Tables S1–S7) (PDF)
