# Supplementary material for: Substrate Length Controls Atropopeptide Complexity and Triggers Excision-Driven Scaffold Hop to Diketopiperazine Alkaloids
Source: J Am Chem Soc. 2026 Oct 12;148(42):45664–74. doi: 10.1021/jacs.6c12504 (PMC13721148; doi:10.1021/jacs.6c12504)
Supplement: Supplementary file 1 [file ja6c12504_si_001.pdf]

Supporting Information

Substrate Length Controls Atropopeptide Complexity and Triggers Excision-Driven Scaffold Hop to Diketopiperazine Alkaloids

Bin Tan,[†] Ziheng Cui,[‡] Yuya Kakumu,[†] Sandra J. Panczyk,[†] Mai Ikeda,[†] Dongyu Liu,[†] Christine M. Pestalozzi,[§] Julia A. Vorholt,[§] Allison S. Walker,[‖,⊥] Kornelia Hardes,[#] Martin Grininger,[‡] Eric J. N. Helfrich*[,†,▽]

[†]Institute for Molecular Bio Science, Goethe University Frankfurt, Max-von-Laue Strasse 9, 60438 Frankfurt am Main, Germany

[‡]Buchmann Institute for Molecular Life Science, Institute of Organic Chemistry and Chemical Biology, Goethe University Frankfurt, Max-von-Laue Strasse 9, 60438 Frankfurt am Main, Germany

[§]Institute of Microbiology, ETH Zurich, 8093 Zurich, Switzerland

[‖]Department of Chemistry, Vanderbilt University, Stevenson Center 7330, Nashville, Tennessee 37240, United States

[⊥]Department of Biological Sciences, Vanderbilt University, VU Station B, Box 35-1634, Nashville, Tennessee 37235, United States

[#]Department of Bioresources, Fraunhofer Institute for Molecular Biology and Applied Ecology, Ohlebergsweg 12, 35392 Giessen, Germany

[▽]Senckenberg Research Institute and Natural History Museum, Senckenberganlage 25, 60325 Frankfurt, Germany

*Correspondence: eric.helfrich@bio.uni-frankfurt.de

Contents

Figure S1. Coexpression of *lauA_L-scaA_C* with *lauB1.* S5
Figure S2. HPLC-ESI-QTOF-HRMS analysis of **2**............ S6
Figure S3. ^{1}H NMR spectrum (600 MHz) of **2** in DMSO-d_6. .... S7
Figure S4. ^{13}C NMR spectrum (125 MHz) of **2** in DMSO-d_6. .... S8
Figure S5. HPLC-ESI-QTOF-HRMS analysis of **3**............ S9
Figure S6. ^{1}H NMR spectrum (500 MHz) of **3** in DMSO-d_6. .... S10
Figure S7. ^{13}C NMR spectrum (125 MHz) of **3** in DMSO-d_6. .... S11
Figure S8. COSY NMR spectrum (500 MHz) of **3** in DMSO-d_6. .... S12
Figure S9. HSQC NMR spectrum (500 MHz) of **3** in DMSO-d_6. .... S13
Figure S10. HMBC NMR spectrum (500 MHz) of **3** in DMSO-d_6. .... S14
Figure S11. ROESY NMR spectrum (500 MHz) of **3** in DMSO-d_6. .... S15
Figure S12. HPLC-ESI-QTOF-HRMS analysis of **4**............ S16
Figure S13. ^{1}H NMR spectrum (600 MHz) of **4** in D_2O. S17
Figure S14. ^{13}C NMR spectrum (125 MHz) of **4** in D_2O............ S18
Figure S15. COSY NMR spectrum (500 MHz) of **4** in D_2O............ S19
Figure S16. HSQC NMR spectrum (500 MHz) of **4** in D_2O. S20
Figure S17. HMBC NMR spectrum (500 MHz) of **4** in D_2O. S21
Figure S18. NOESY NMR spectrum (500 MHz) of **4** in D_2O. S22
Figure S19. Coexpression of *lauA-6S* with *lauB1*.. .... S23
Figure S20. HPLC-ESI-QTOF-HRMS analysis of **5**............ S24
Figure S21. ^{1}H NMR spectrum (600 MHz) of **5** in DMSO-d_6. .... S25
Figure S22. ^{13}C NMR spectrum (125 MHz) of **5** in DMSO-d_6. .... S26
Figure S23. Coexpression of *lauA-6X* variants with *lauB1*.. .... S27
Figure S24. HRMS analysis of extracts of the recombinant *S. albus* harboring *lauA*-6A and *lauB1*.. .... S28
Figure S25. HRMS analysis of extracts of the recombinant *S. albus* harboring *lauA*-6C and *lauB1*. S29
Figure S26. HRMS analysis of extracts of the recombinant *S. albus* harboring *lauA*-6E and *lauB1* S30
Figure S27. HRMS analysis of extracts of the recombinant *S. albus* harboring *lauA*-6G and *lauB1*. S31
Figure S28. HRMS analysis of extracts of the recombinant *S. albus* harboring *lauA*-6H and *lauB1*. S32
Figure S29. HRMS analysis of extracts of the recombinant *S. albus* harboring *lauA*-6N and *lauB1*. S33
Figure S30. HRMS analysis of extracts of the recombinant *S. albus* harboring *lauA*-6R and *lauB1*. S34
Figure S31. HRMS analysis of extracts of the recombinant *S. albus* harboring *lauA*-6T and *lauB1*............ S35
Figure S32. HRMS analysis of extracts of the recombinant *S. albus* harboring *lauA*-6V and *lauB1*. S36
Figure S33. HRMS analysis of extracts of the recombinant *S. albus* harboring *lauA*-6Y and *lauB1* S37
Figure S34. Coexpression of *lauA-Y2F* with *lauB1*............ S38
Figure S35. Spontaneous transformation of **6** into **7**.. .... S39
Figure S36. ^{1}H NMR spectrum (600 MHz) of Tyr isolated from NMR sample of **6** in D_2O. S40
Figure S37. HPLC-ESI-QTOF-HRMS analysis of **7**............ S41
Figure S38. ^{1}H NMR spectrum (600 MHz) of **7** in DMSO-d_6. .... S42
Figure S39. ^{13}C NMR spectrum (150 MHz) of **7** in DMSO-d_6. .... S43
Figure S40. COSY NMR spectrum (600 MHz) of **7** in DMSO-d_6. .... S44
Figure S41. HSQC NMR spectrum (600 MHz) of **7** in DMSO-d_6. .... S45
Figure S42. HMBC NMR spectrum (600 MHz) of **7** in DMSO-d_6. .... S46
Figure S43. NOESY NMR spectrum (600 MHz) of **7** in DMSO-d_6. .... S47
Figure S44. Stability test of **6** in different solvents............ S48
Figure S45. HPLC-ESI-QTOF-HRMS analysis of **6**............ S49
Figure S46. ^{1}H NMR spectrum (600 MHz) of **6** in MeOD. S50
Figure S47. ^{13}C NMR spectrum (125 MHz) of **6** in MeOD. S51
Figure S48. COSY NMR spectrum (500 MHz) of **6** in MeOD. S52
Figure S49. HSQC NMR spectrum (500 MHz) of **6** in MeOD. S53
Figure S50. HMBC NMR spectrum (500 MHz) of **6** in MeOD. S54
Figure S51. NOESY NMR spectrum (500 MHz) of **6** in MeOD. S55
Figure S52. Molecular dynamics simulations of **6**. S56
Figure S53. Spontaneous transformation of **6** into **7** in different conditions S57
Figure S54. Mechanistic investigation of the proposed diketopiperazine formation. S58
Figure S55. Coexpression of *lauA-Y2X* variants with *lauB1*. S59

Figure S56. HRMS analysis of extracts of the recombinant *S. albus* harboring *lauA*-Y2A and *lauB1* S60
Figure S57. HRMS analysis of extracts of the recombinant *S. albus* harboring *lauA*-Y2E and *lauB1* S61
Figure S58. HRMS analysis of extracts of the recombinant *S. albus* harboring *lauA*-Y2H and *lauB1*...................... S62
Figure S59. HRMS analysis of extracts of the recombinant *S. albus* harboring *lauA*-Y2R and *lauB1*. S63
Figure S60. HRMS analysis of extracts of the recombinant *S. albus* harboring *lauA*-Y2T and *lauB1*.. .... S64
Figure S61. HRMS analysis of extracts of the recombinant *S. albus* harboring *lauA*-Y2V and *lauB1*. S65
Figure S62. HRMS analysis of extracts of the recombinant *S. albus* harboring *lauA*-Y2W and *lauB1*. S66
Figure S63. HRMS analysis of extracts of the recombinant *S. albus* harboring *lauA*-Y2P and *lauB1* S67
Figure S64. Bioconversion assays of **3** using crude enzyme extracts from *S. albus*. S68
Figure S65. Heterologous expression of the *xat* BGC.. .... S69
Figure S66. MS/MS fragmentation comparison of **9** and the major product from the *xat* BGC. S70
Figure S67. Heterologous expression of *ska* BGC S71
Figure S68. HPLC-ESI-QTOF-HRMS analysis of **9**. S72
Figure S69. ^{1}H NMR spectrum (600 MHz) of **9** in D_2O. S73
Figure S70. ^{13}C NMR spectrum (125 MHz) of **9** in D_2O. S74
Figure S71. COSY spectrum (600 MHz) of **9** in D_2O. S75
Figure S72. HSQC spectrum (500 MHz) of **9** in D_2O. S76
Figure S73. HMBC spectrum (500 MHz) of **9** in D_2O. S77
Figure S74. NOESY spectrum (500 MHz) of **9** in D_2O. S78
Figure S75. HPLC-ESI-QTOF-HRMS analysis of **10**. S79
Figure S76. ^{1}H NMR spectrum (500 MHz) of **10** in DMSO-d_6. .... S80
Figure S77. ^{13}C NMR spectrum (125 MHz) of **10** in DMSO-d_6. .... S81
Figure S78. Bioconversion assays of **3** using crude enzyme extracts from *S. kanamyceticus*. S82
Figure S79. Bioconversion assays of **3** using crude enzyme extracts from *X. translucens* pv. translucens.. .... S83
Figure S80. MS/MS spectrum of the atropopeptide detected at *m*/*z* 494.7318 $[M+2H]^2$. S84
Figure S81. MS/MS spectrum of the atropopeptide detected at *m*/*z* 459.2139 $[M+2H]^{2+}$ S85
Figure S82. Spontaneous transformation of **4** into a diketopiperazine. S86
Figure S83. Spontaneous transformation of **9** into a diketopiperazine.. .... S87
Figure S84. Coexpression of *lauA-Y2F-I3X* variants with *lauB1* S88
Figure S85. HRMS analysis of extracts of the recombinant *S. albus* harboring *lauA*-Y2F-I3A and *lauB1*................ S89
Figure S86. HRMS analysis of extracts of the recombinant *S. albus* harboring *lauA*-Y2F-I3E and *lauB1*................ S90
Figure S87. HRMS analysis of extracts of the recombinant *S. albus* harboring *lauA*-Y2F-I3H and *lauB1*.. .... S91
Figure S88. HRMS analysis of extracts of the recombinant *S. albus* harboring *lauA*-Y2F-I3L and *lauB1*.. .... S92
Figure S89. HRMS analysis of extracts of the recombinant *S. albus* harboring *lauA*-Y2F-I3Q and *lauB1*.. .... S93
Figure S90. HRMS analysis of extracts of the recombinant *S. albus* harboring *lauA*-Y2F-I3R and *lauB1*. S94
Figure S91. HRMS analysis of extracts of the recombinant *S. albus* harboring *lauA*-Y2F-I3T and *lauB1*. S95
Figure S92. HRMS analysis of extracts of the recombinant *S. albus* strain harboring *lauA*-Y2F-I3P and *lauB1*....... S96
Figure S93. Coexpression of *lauA-Y2F-Y5X* variants with *lauB1*.. .... S97
Figure S94. HRMS analysis of extracts of the recombinant *S. albus* harboring *lauA*-Y2F-Y5F and *lauB1*. S98
Figure S95. HRMS analysis of extracts of the recombinant *S. albus* harboring *lauA*-Y2F-Y5L and *lauB1*............... S99
Figure S96. HRMS analysis of extracts of the recombinant *S. albus* harboring *lauA*-Y2F-Y5P and *lauB1*. S100
Figure S97. HRMS analysis of extracts of the recombinant *S. albus* harboring *lauA*-Y2F-Y5Q and *lauB1*............ S101
Figure S98. HRMS analysis of extracts of the recombinant *S. albus* harboring *lauA*-Y2F-Y5T and *lauB1* S102
Figure S99. HRMS analysis of extracts of the recombinant *S. albus* harboring *lauA*-Y2F-Y5W and *lauB1* S103
Figure S100. HRMS analysis of extracts of the recombinant *S. albus* harboring *lauA*-Y2F-Y5A and *lauB1* S104
Figure S101. Investigation of DKP formation from crude extracts resuspended in DMSO S105
Figure S102. Antiviral activity of **6** and **7**. S106
Figure S103. Predicted structural models of the LauB1-LauA, LauB1-LauA-Y2F, LauB1-LauA-6S and ScaB-ScaA complexes generated by AlphaFold2.. .... S107
Table S1. ^{1}H (δ_H, 600 MHz) and ^{13}C NMR (δ_C, 125 MHz) NMR data for **3** in DMSO-d_6. .... S108
Table S2. ^{1}H (δ_H, 600 MHz) and ^{13}C NMR (δ_C, 125 MHz) NMR data for **4** in D_2O S110
Table S3. ^{1}H (δ_H, 600 MHz) and ^{13}C NMR (δ_C, 125 MHz) NMR data for **6** in MeOD. S111
Table S4. ^{1}H (δ_H, 600 MHz) and ^{13}C NMR (δ_C, 150 MHz) NMR data for **7** in DMSO-d_6. .... S112
Table S5. ^{1}H (δ_H, 600 MHz) and ^{13}C NMR (δ_C, 125 MHz) NMR data for **9** in D_2O S113
Table S6. Strains and plasmids used in this study S114
Table S7. Primers used in this study S117
Materials and methods S120
General materials S120

Strains and culture conditions S120
DNA isolation, manipulation and sequencing S120
Construction of recombinant plasmids S121
Generation of *Xanthomonas* sp. Leaf148/Δ*xal* S122
Heterologous expression of engineered or native BGCs S122
Small-scale fermentation and LC-MS analysis S123
Large-scale fermentation and purification of compounds S124
Physical and spectroscopic data for the isolated compounds S126
NMR analysis S127
Structure elucidation S127
Investigation of conditions for the spontaneous transformation of atropopeptides into DKPs S131
Molecular dynamic simulations S132
DTF calculations S132
Detailed mechanistic insights of diketopiperazine formation S133
Feeding experiments S134
Crude enzyme extract assays S134
Bioactivity investigation S135
Prediction of P450-precursor complex S136
DNA sequences S137
References: S139

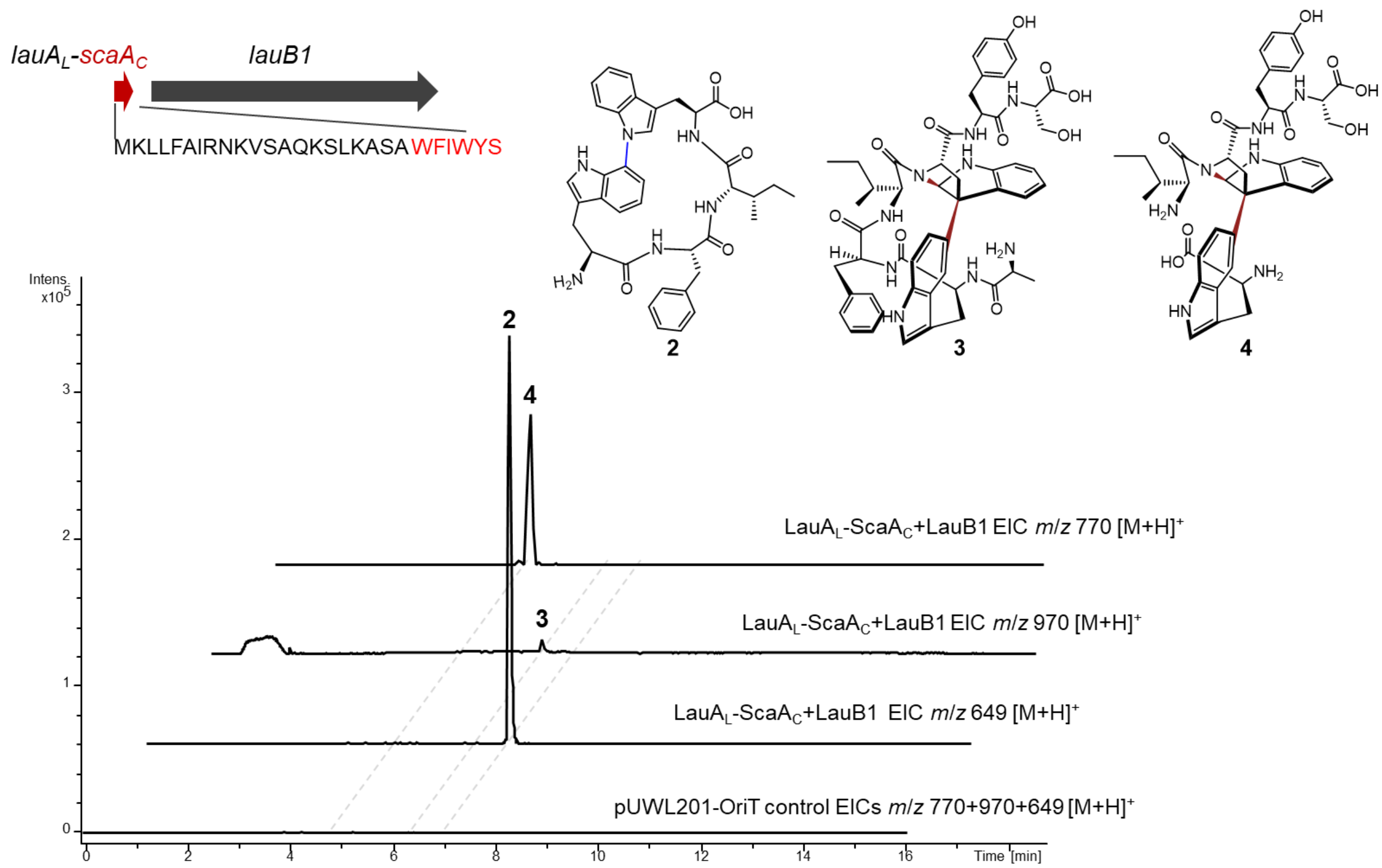

Figure S1. Coexpression of *lauA*$_L$*-scaA*$_C$ with *lauB1.* Extracted ion chromatograms of **2**, **3** and **4** from extracts of *S. albus* harboring pUWL201-OriT-LauA$_L$-ScaA$_C$+LauB1 and *S. albus* harboring pUWL201-OriT as a negative control.

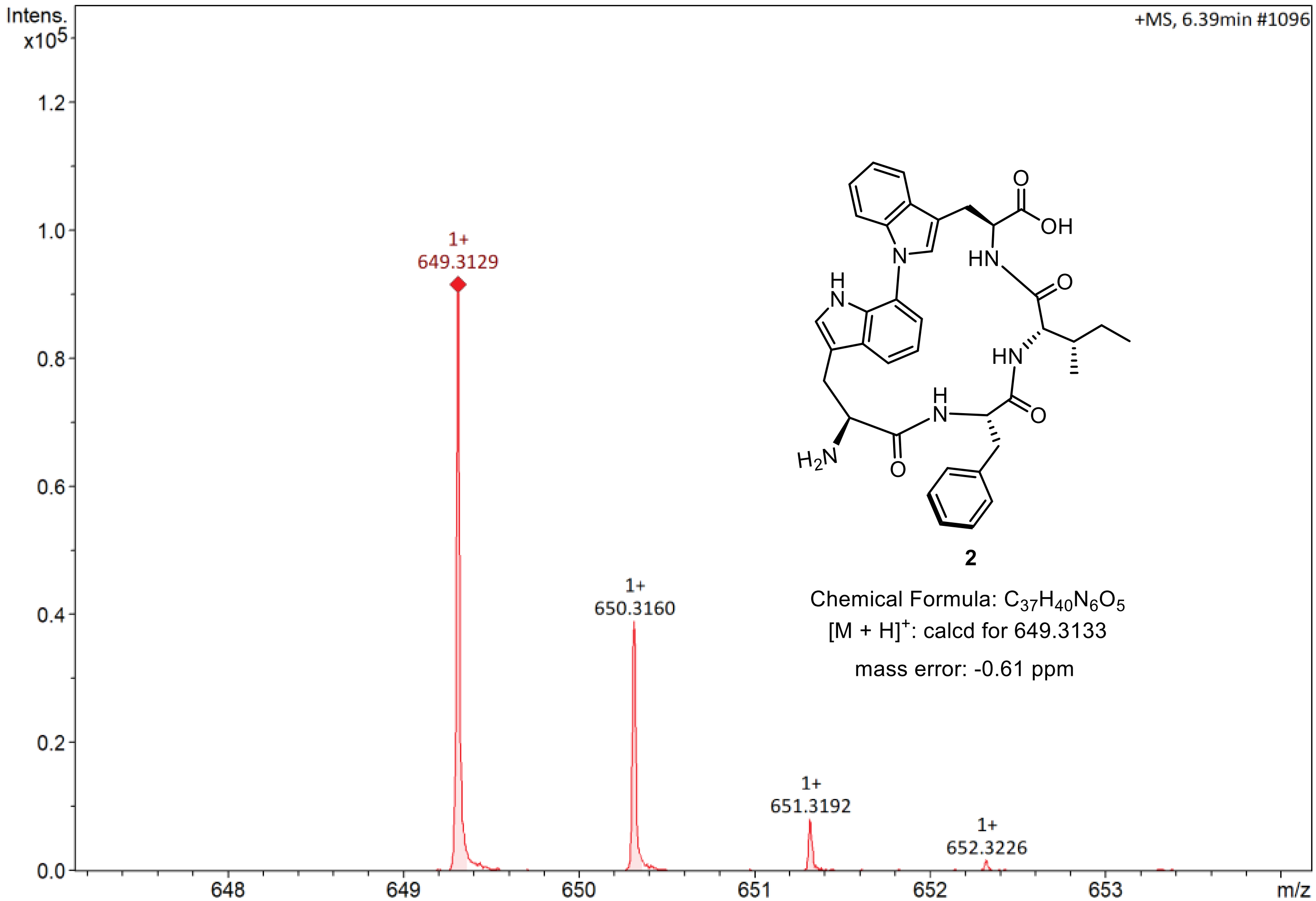

Figure S2. HPLC-ESI-QTOF-HRMS analysis of **2**.

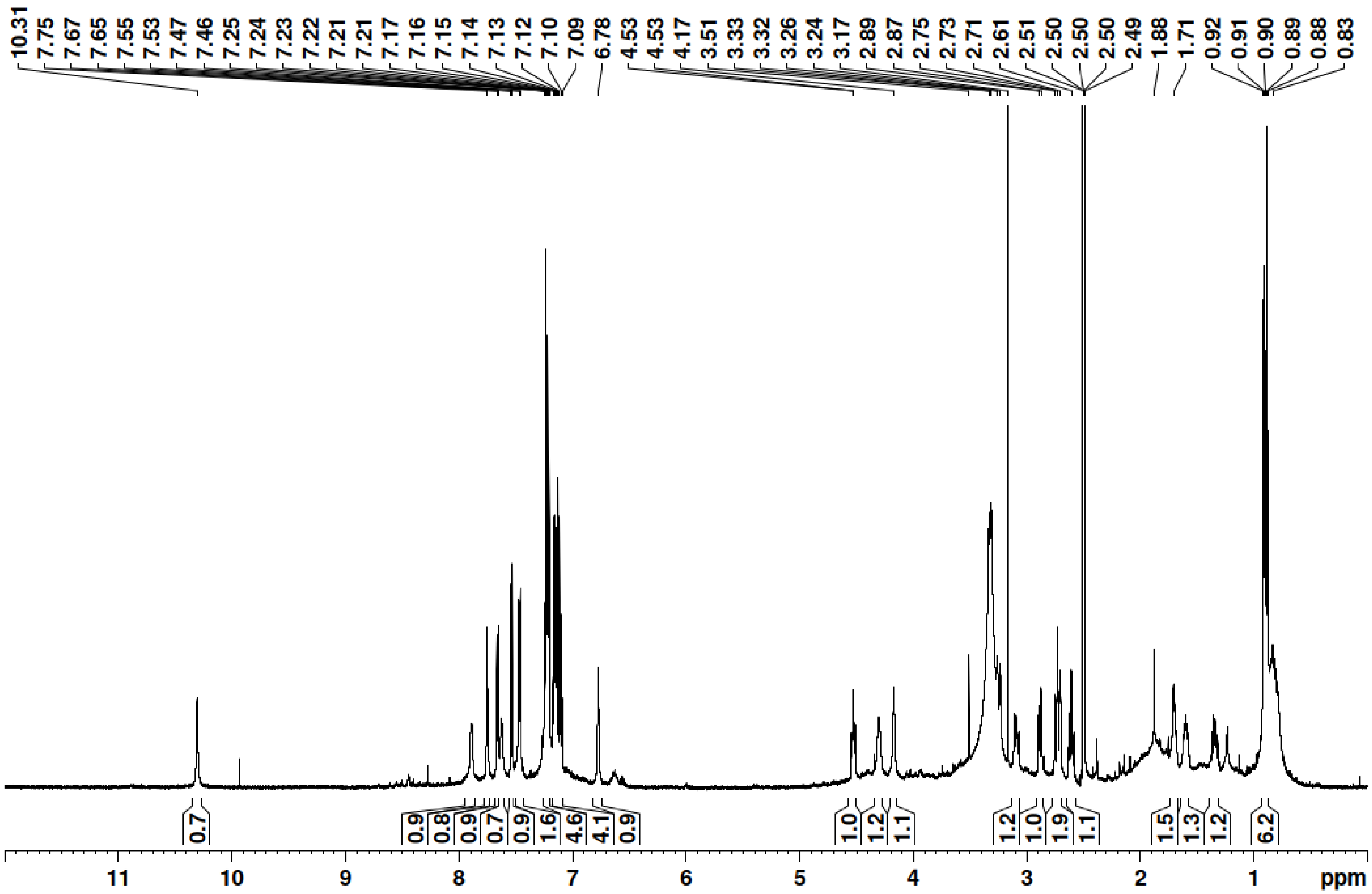

Figure S3. ^{1}H NMR spectrum (600 MHz) of **2** in DMSO-d_6.

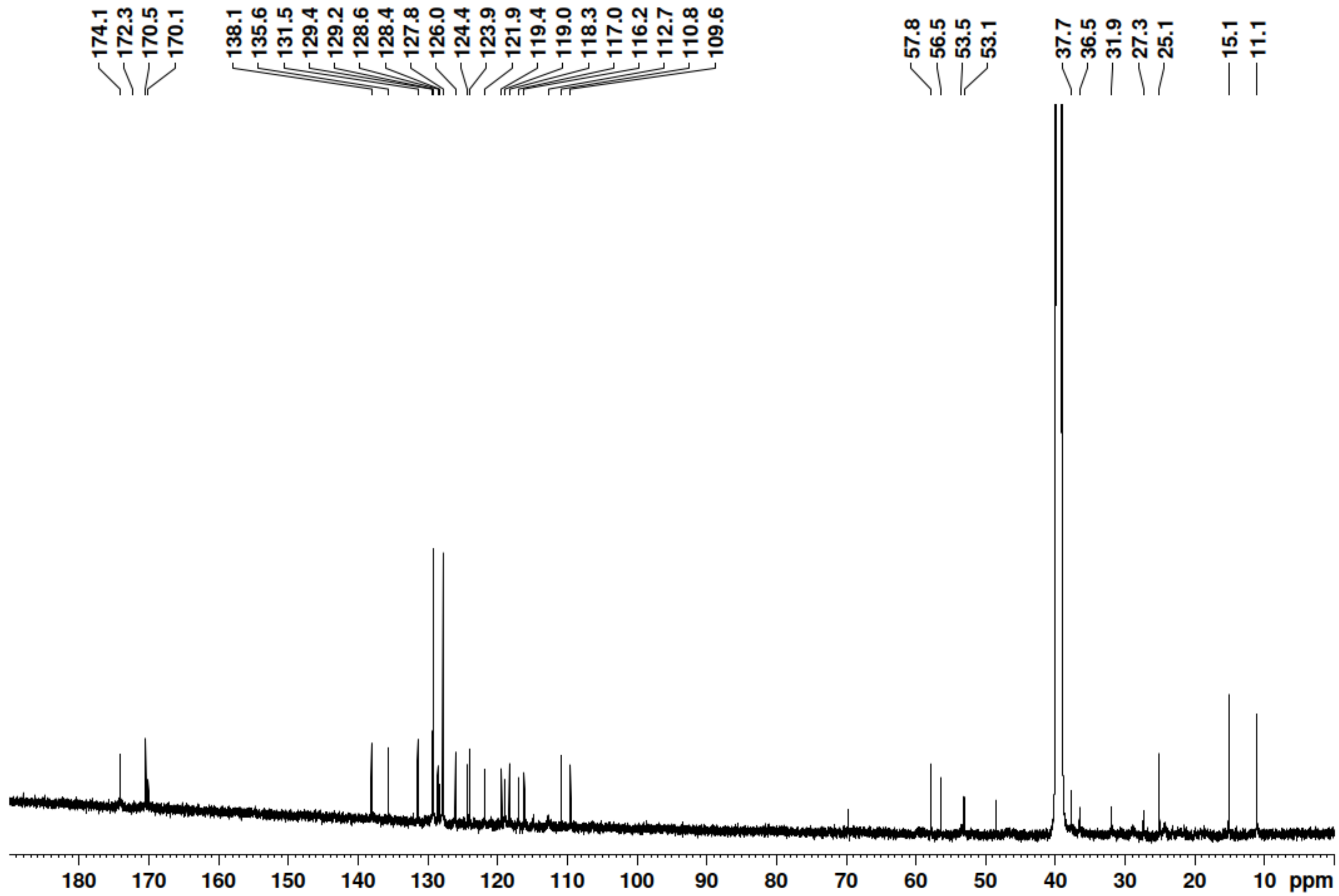

Figure S4. ^{13}C NMR spectrum (125 MHz) of **2** in DMSO-d_6.

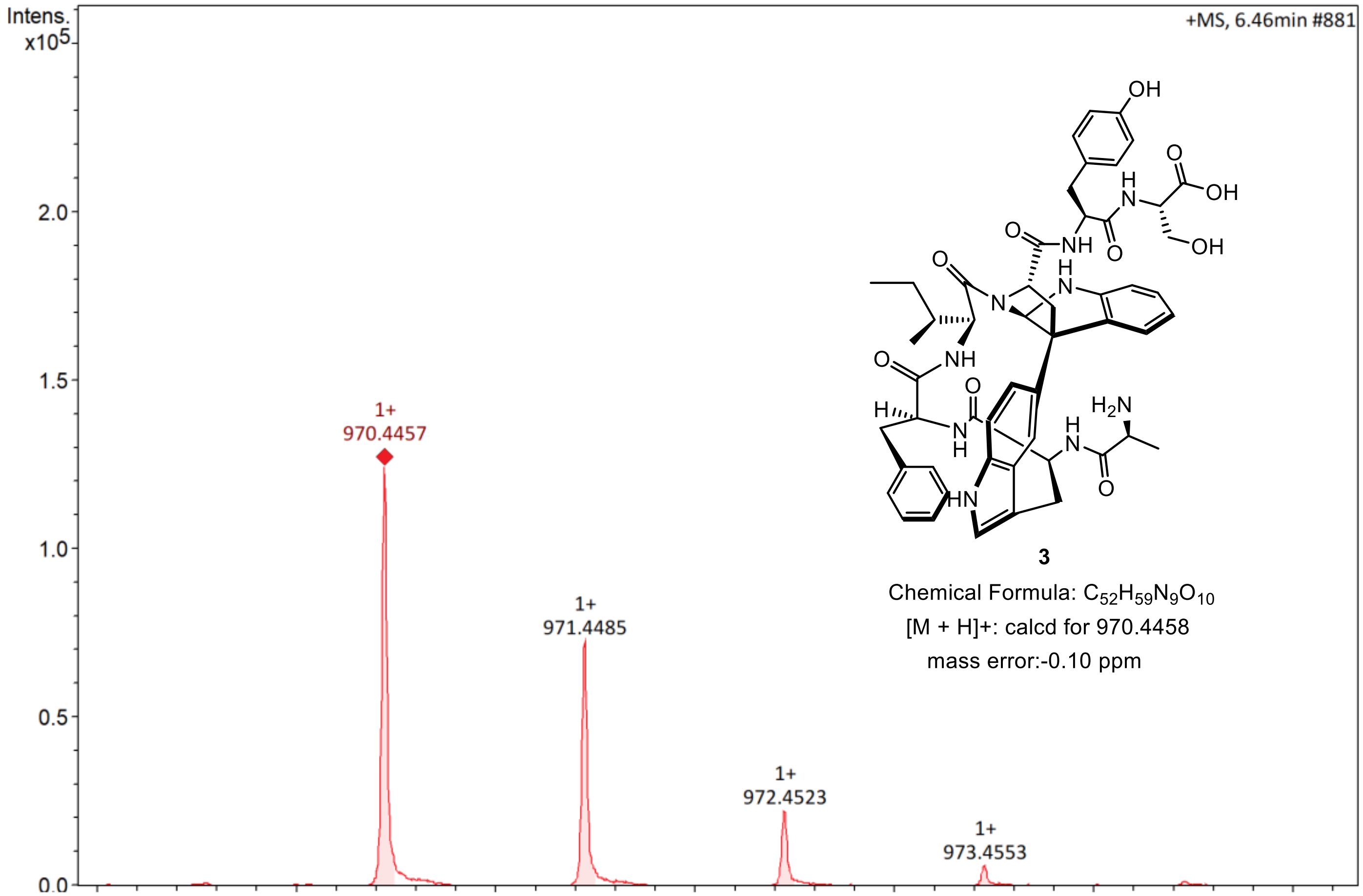

Figure S5. HPLC-ESI-QTOF-HRMS analysis of **3**.

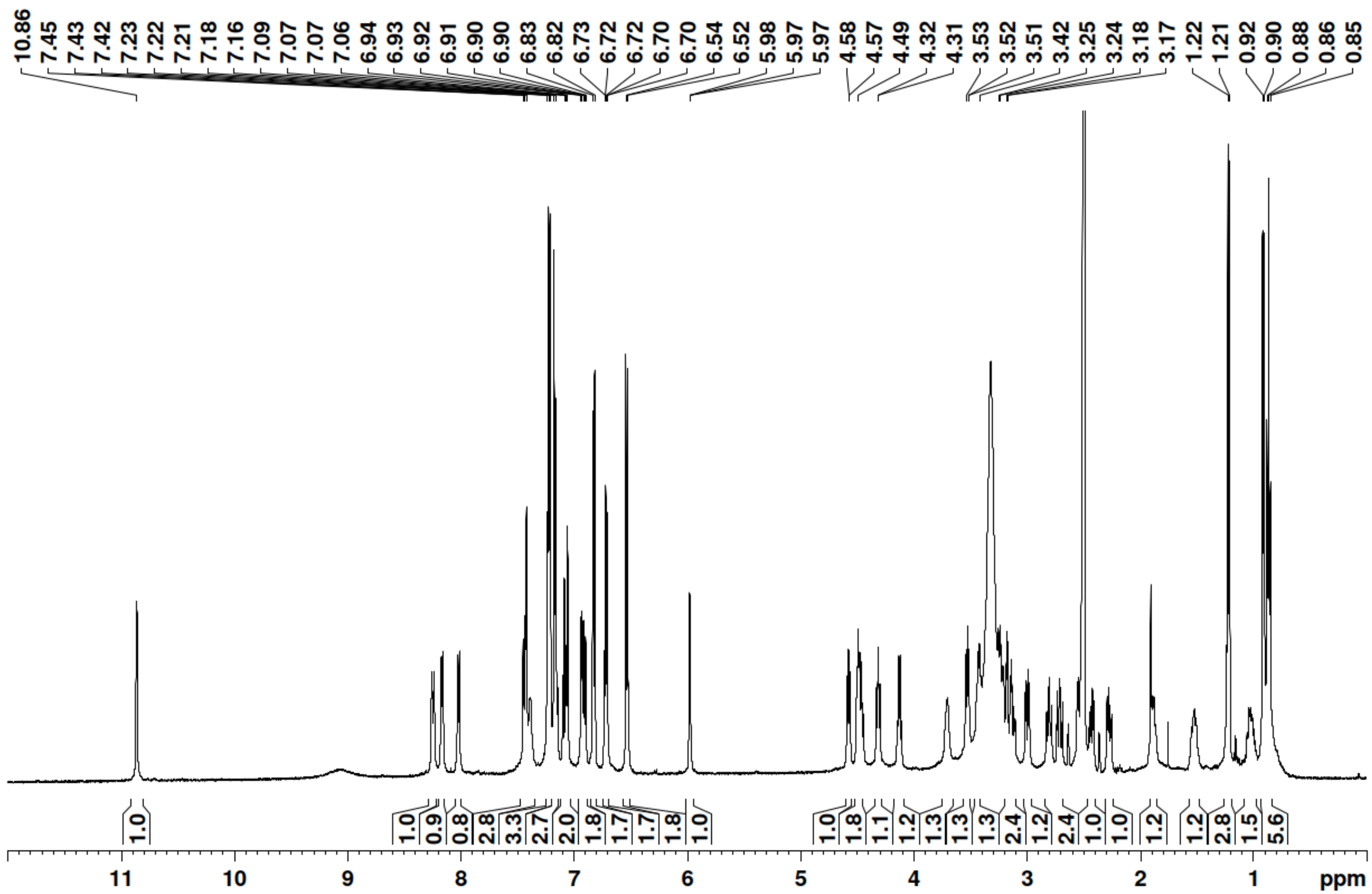

Figure S6. ^{1}H NMR spectrum (500 MHz) of **3** in DMSO-d_6.

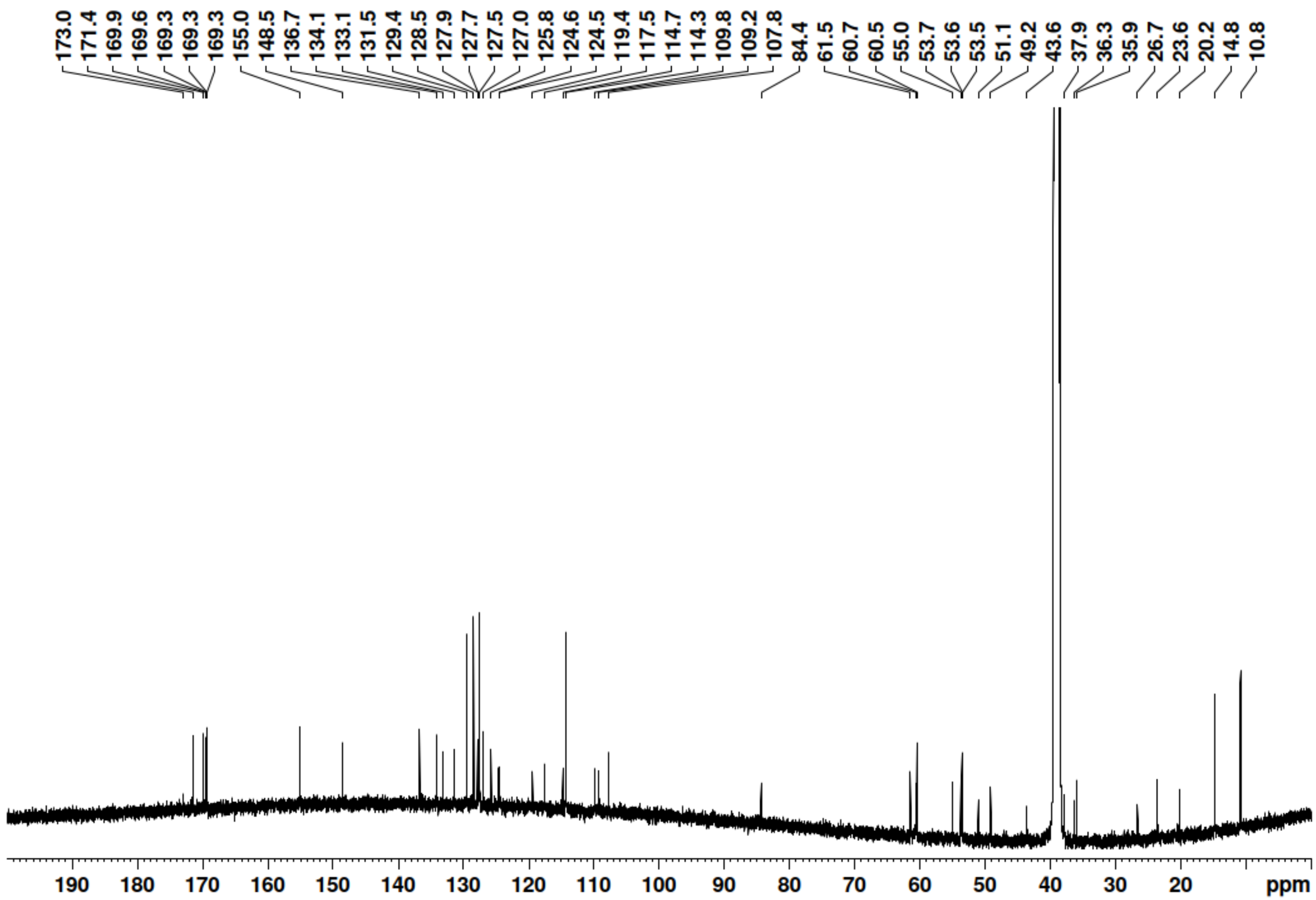

Figure S7. ^{13}C NMR spectrum (125 MHz) of **3** in DMSO-d_6.

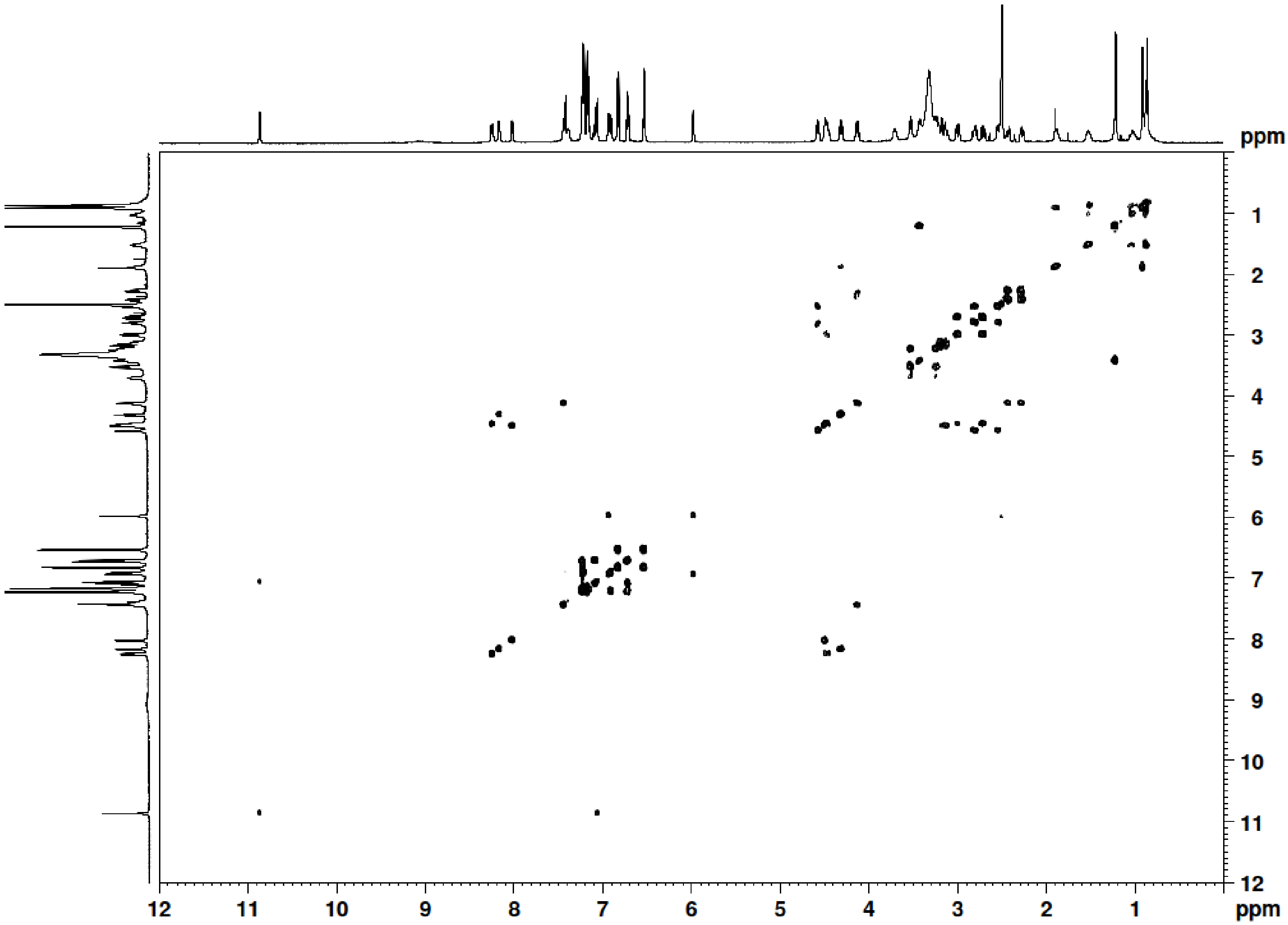

Figure S8. COSY NMR spectrum (500 MHz) of **3** in DMSO-d_6.

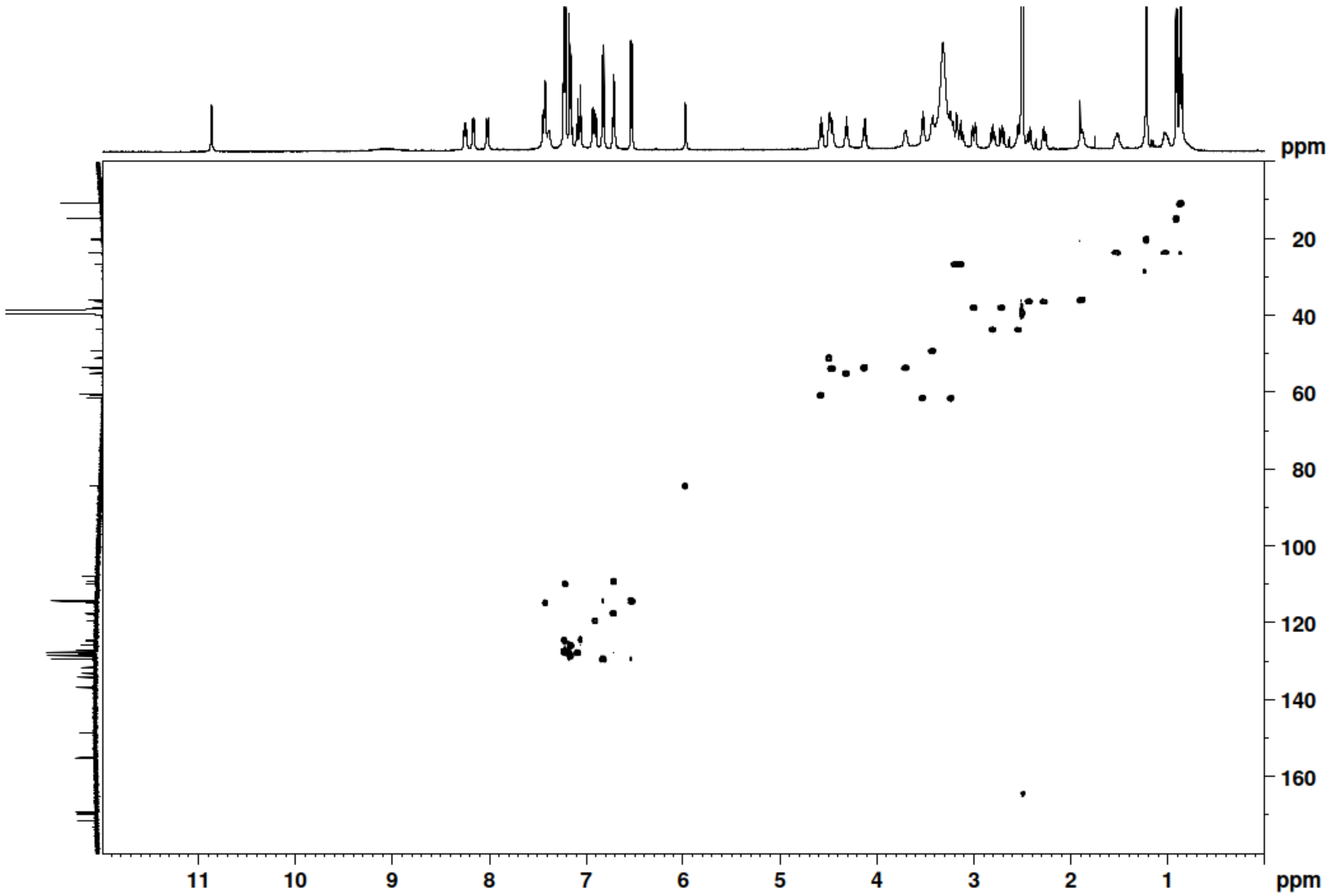

Figure S9. HSQC NMR spectrum (500 MHz) of **3** in DMSO-d_6.

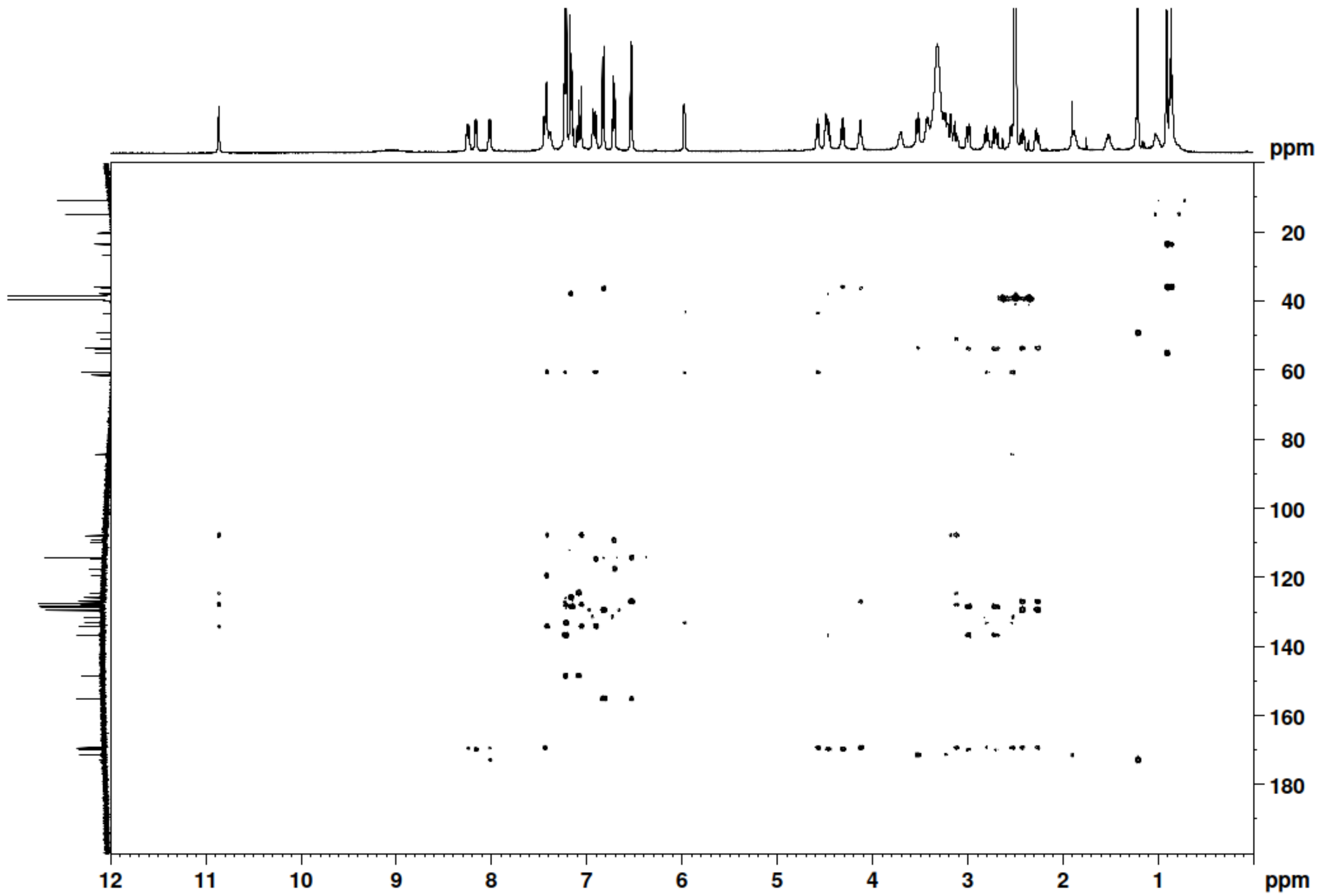

Figure S10. HMBC NMR spectrum (500 MHz) of **3** in DMSO-d_6.

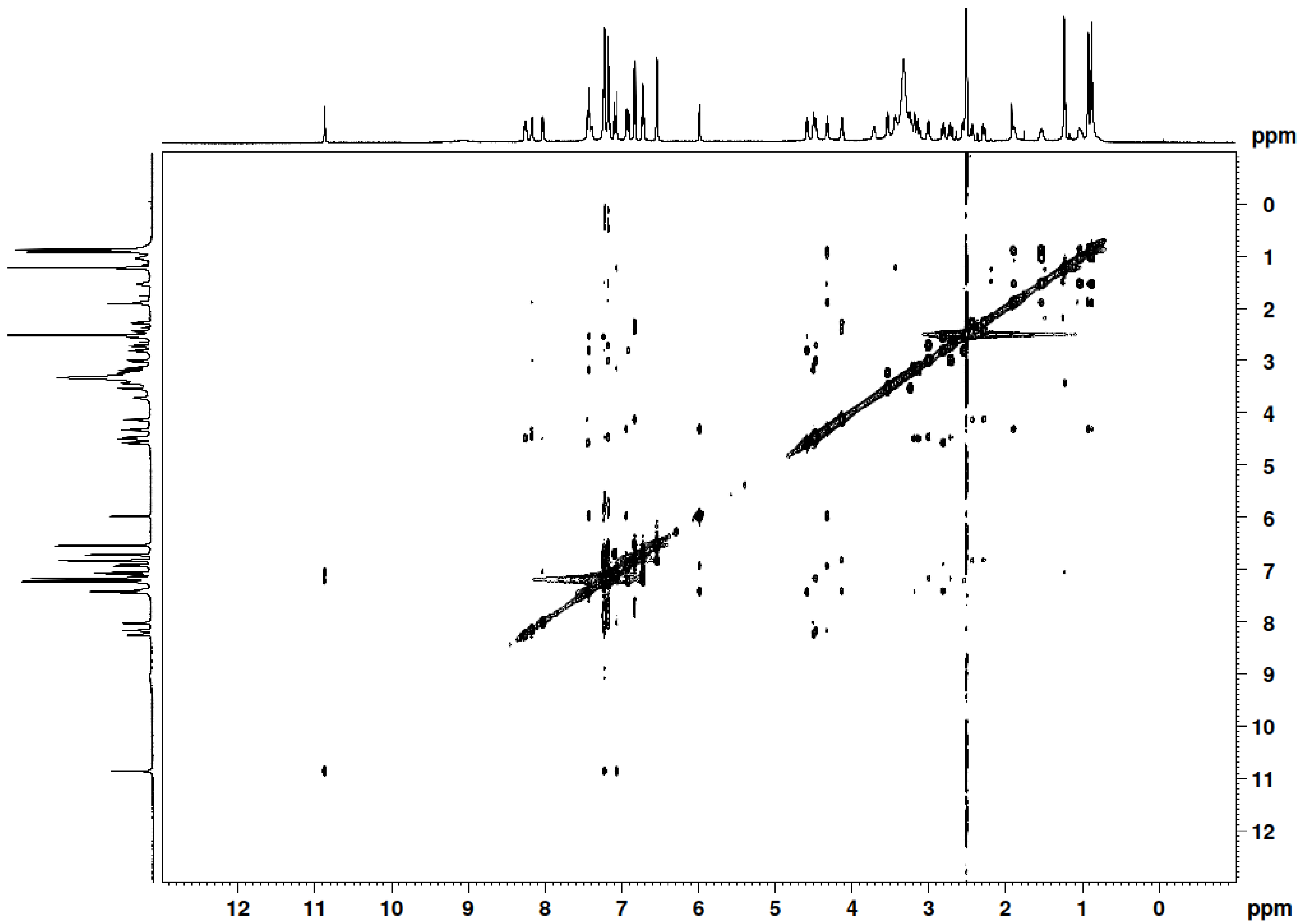

Figure S11. ROESY NMR spectrum (500 MHz) of **3** in DMSO-d_6.

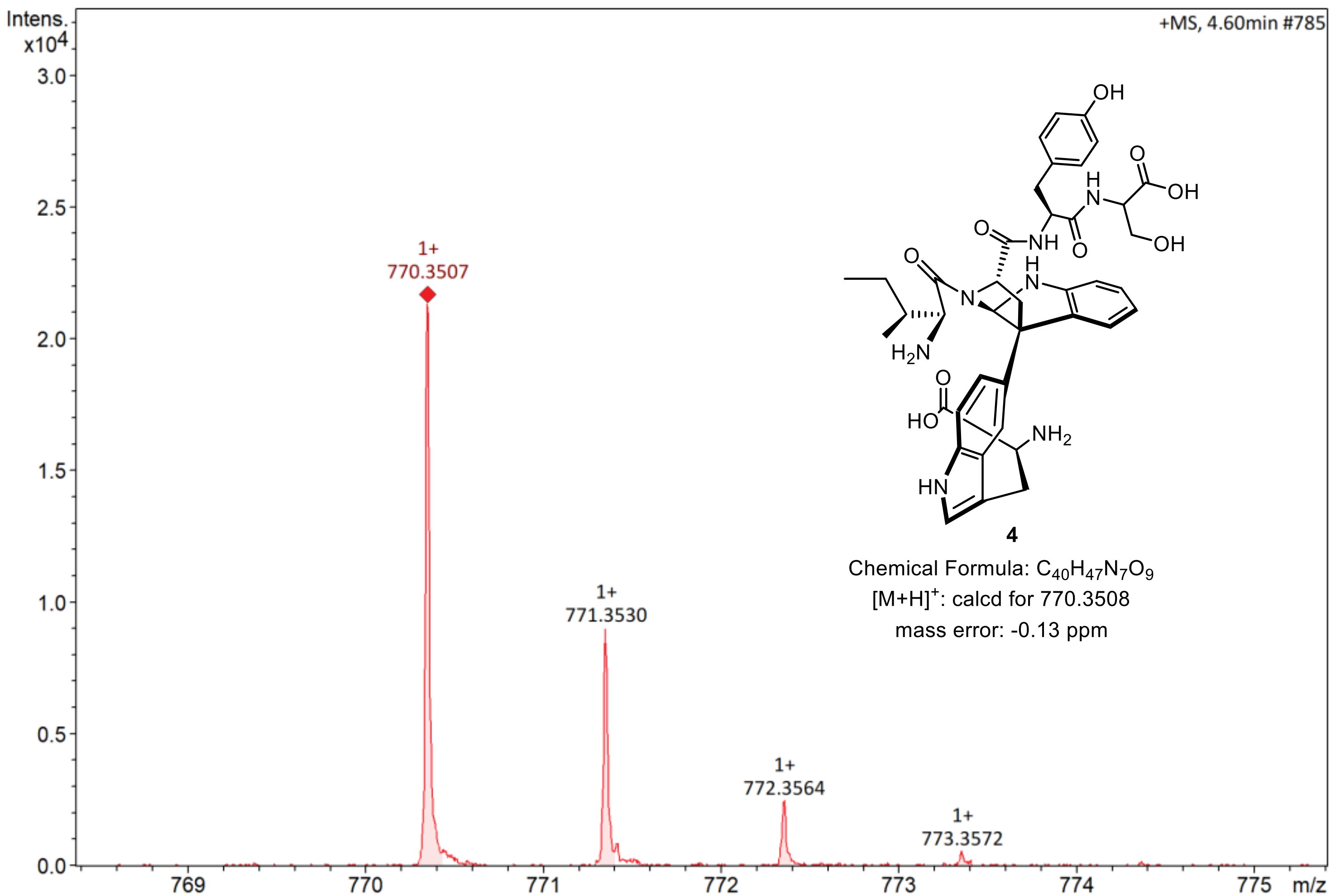

Figure S12. HPLC-ESI-QTOF-HRMS analysis of **4**.

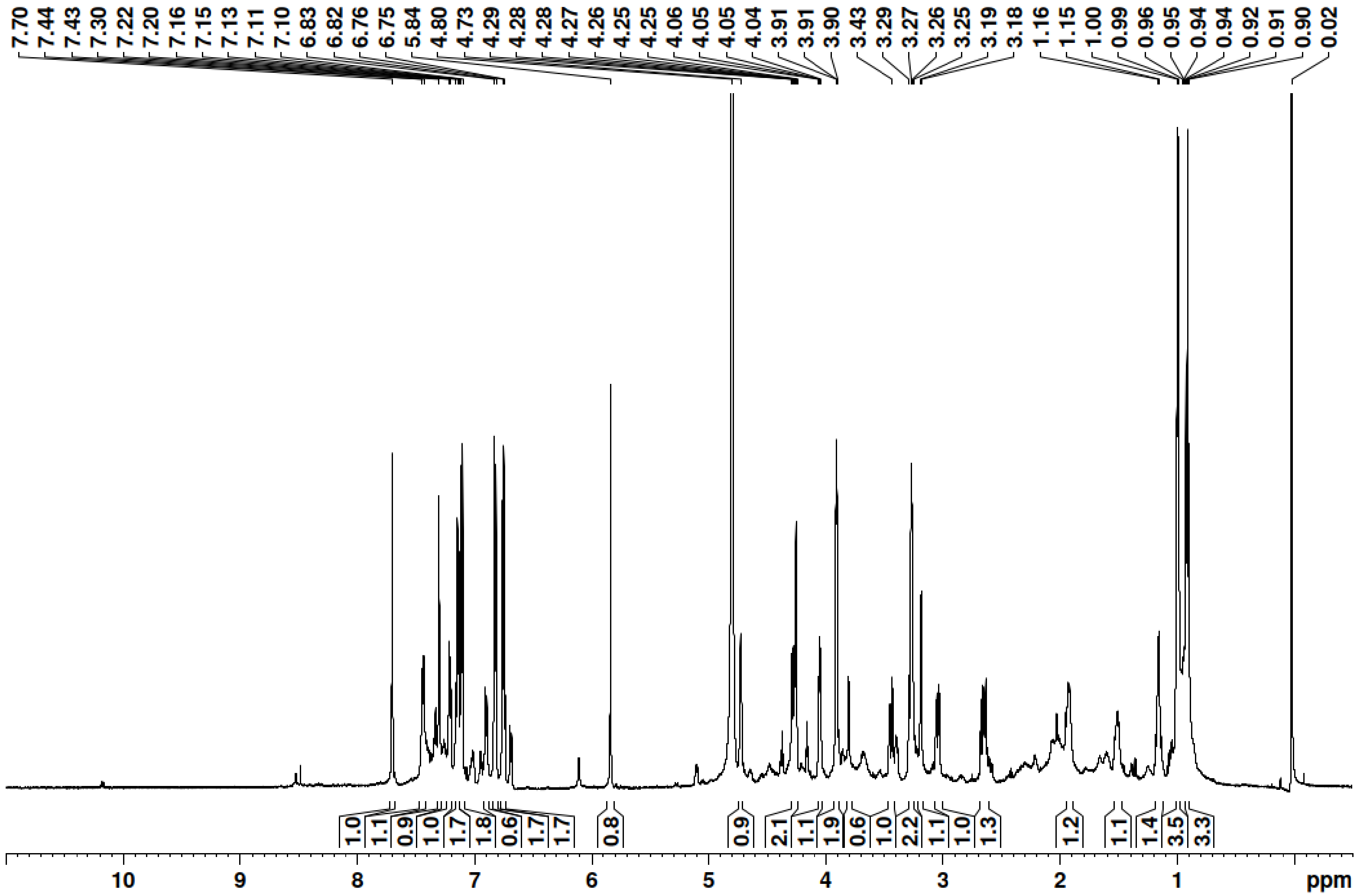

Figure S13. ^{1}H NMR spectrum (600 MHz) of **4** in D_2O.

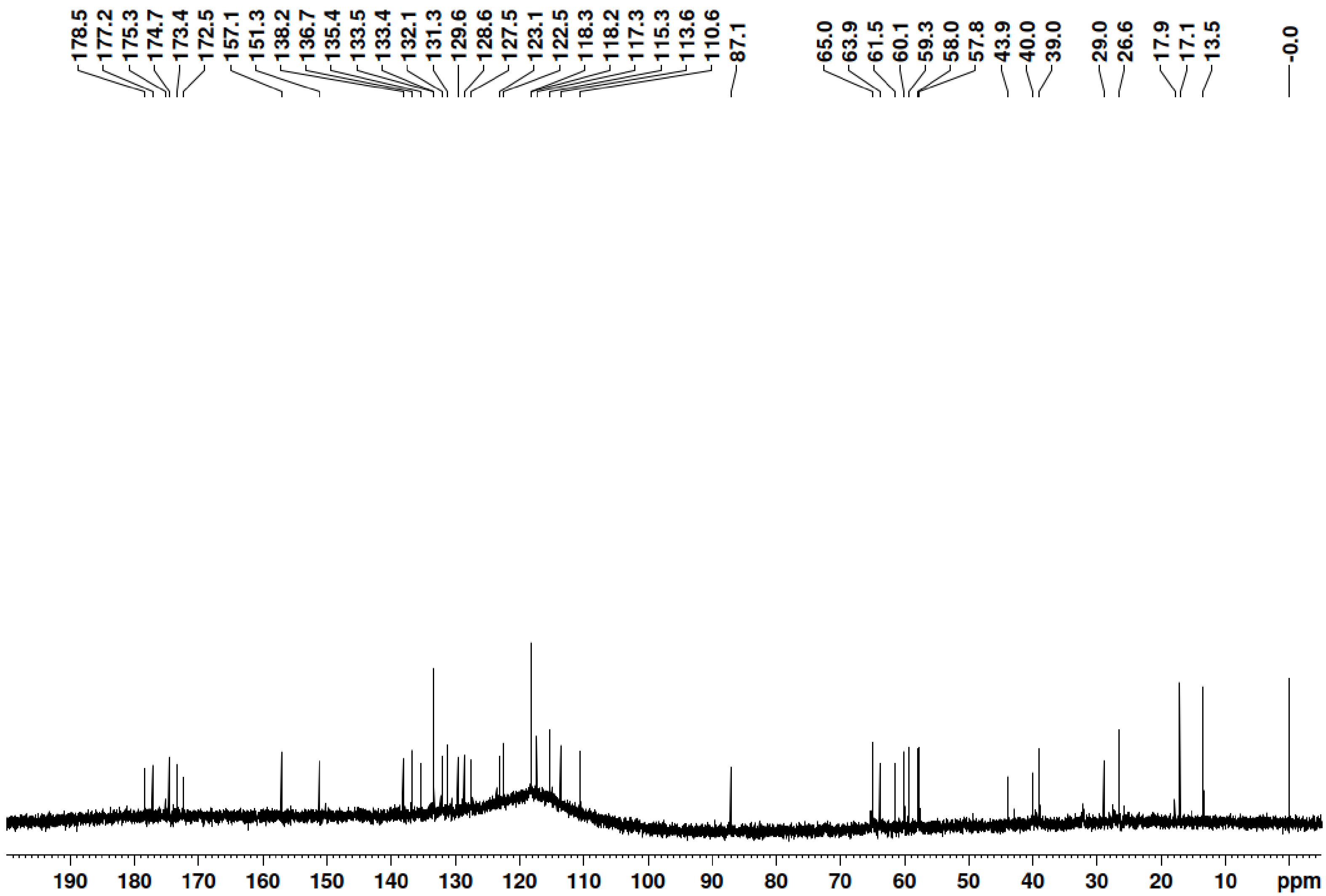

Figure S14. ^{13}C NMR spectrum (125 MHz) of **4** in D_2O.

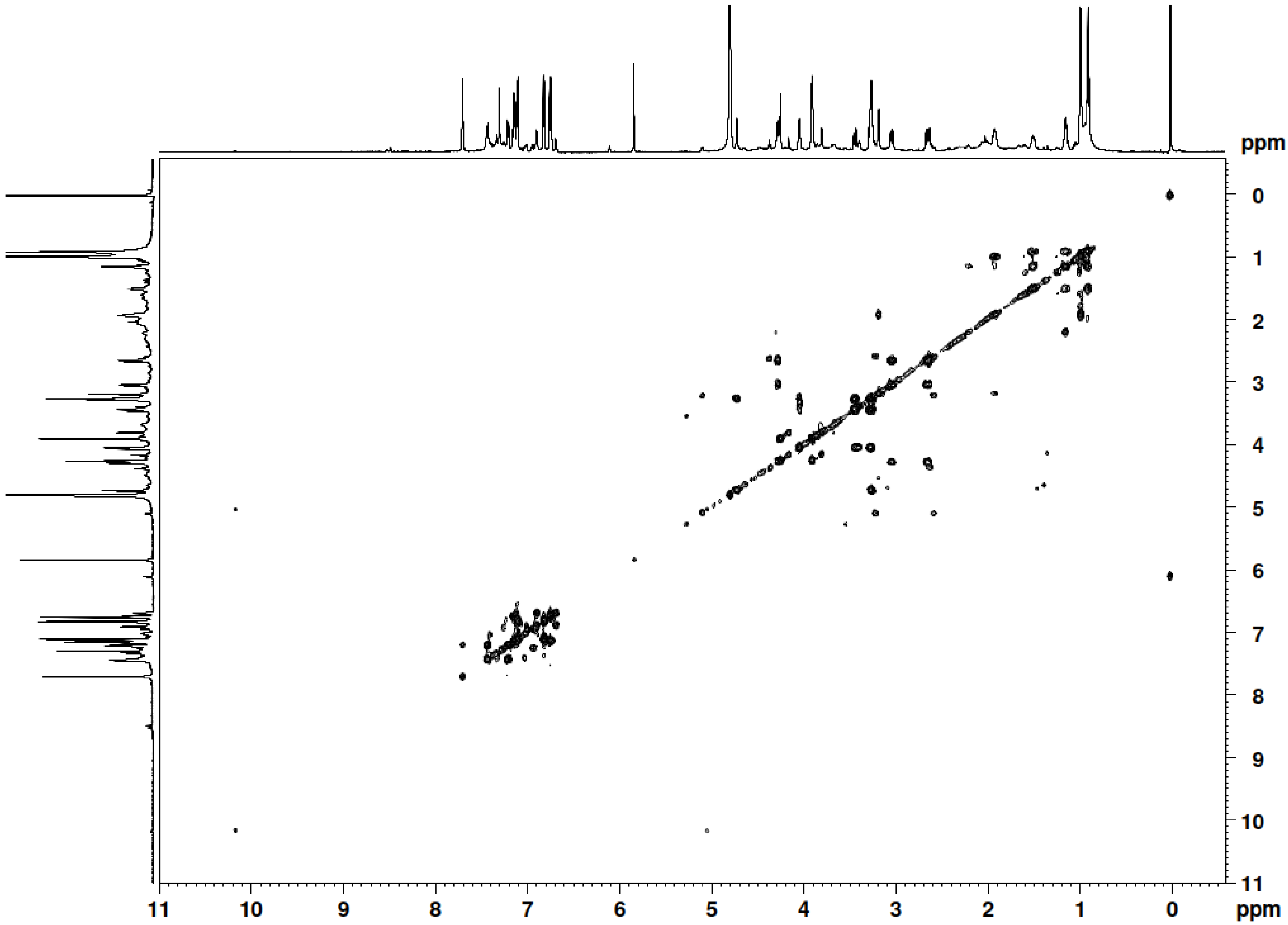

Figure S15. COSY NMR spectrum (500 MHz) of **4** in D_2O.

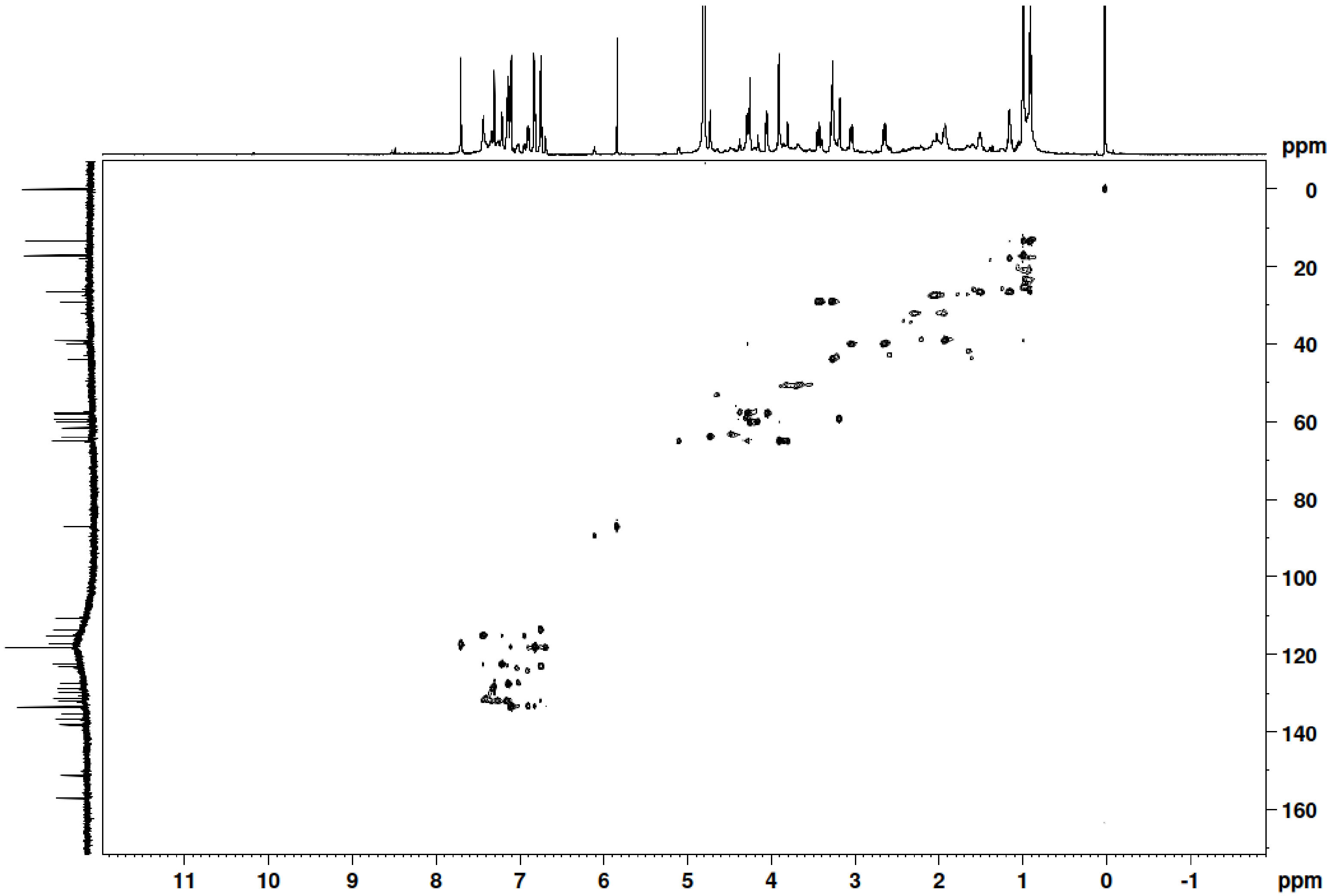

Figure S16. HSQC NMR spectrum (500 MHz) of **4** in D_2O.

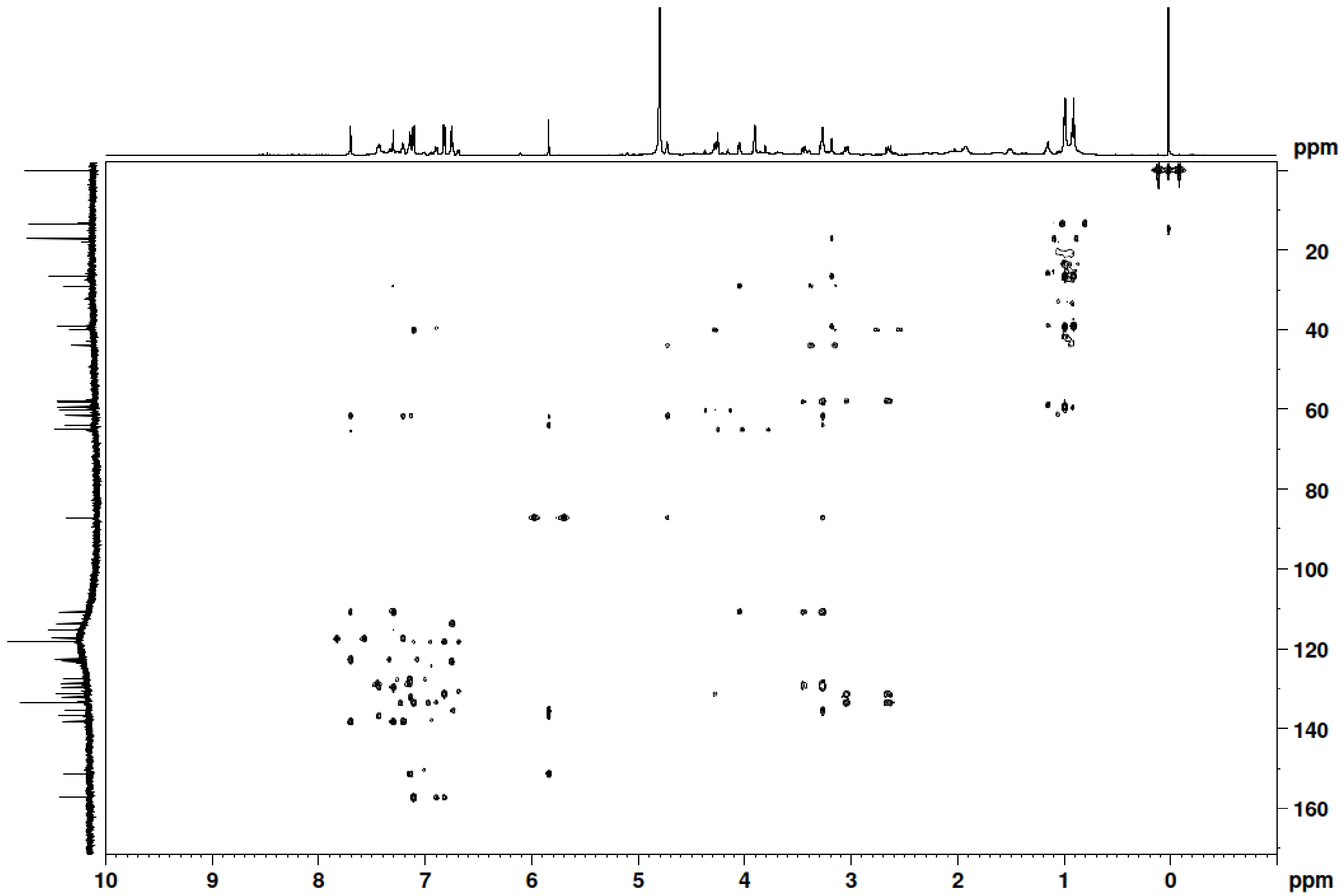

Figure S17. HMBC NMR spectrum (500 MHz) of **4** in D_2O.

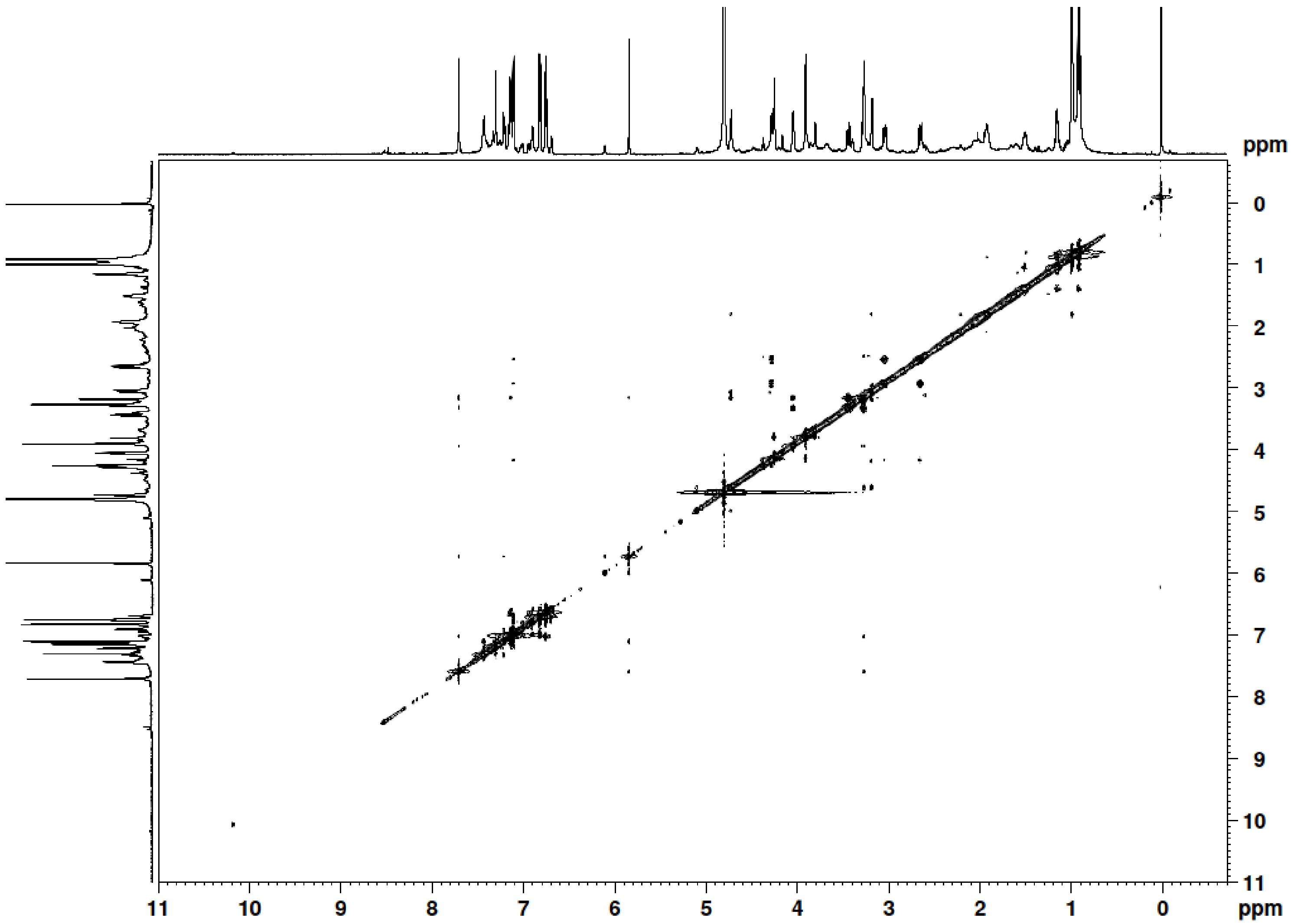

Figure S18. NOESY NMR spectrum (500 MHz) of **4** in D_2O.

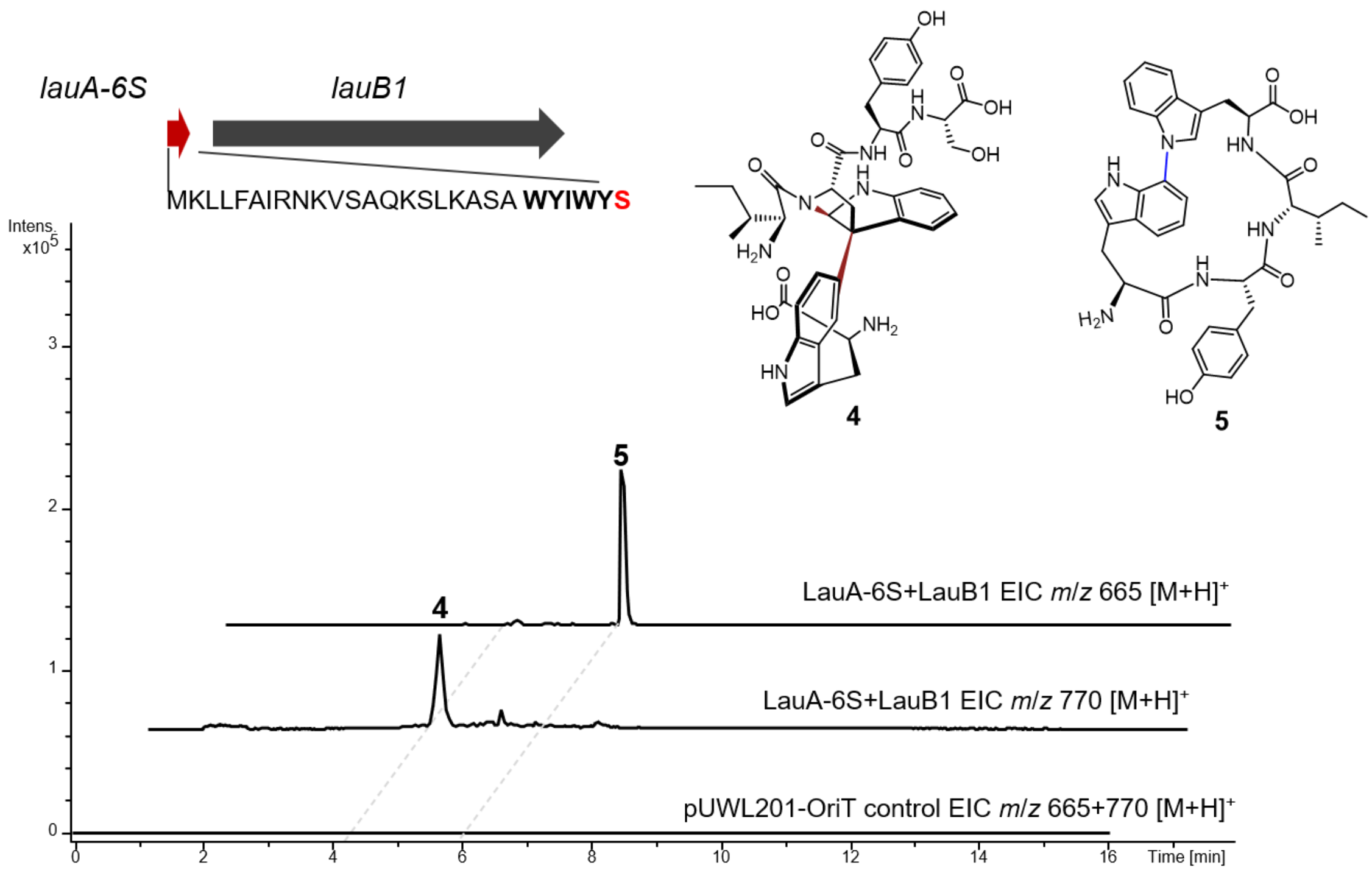

Figure S19. Coexpression of *lauA-6S* with *lauB1*. Extracted ion chromatogram of compounds **4** and **5** from extracts of *S. albus* harboring pUWL201-OriT-LauA-6S + LauB1 and *S. albus* harboring pUWL201-OriT as a negative control.

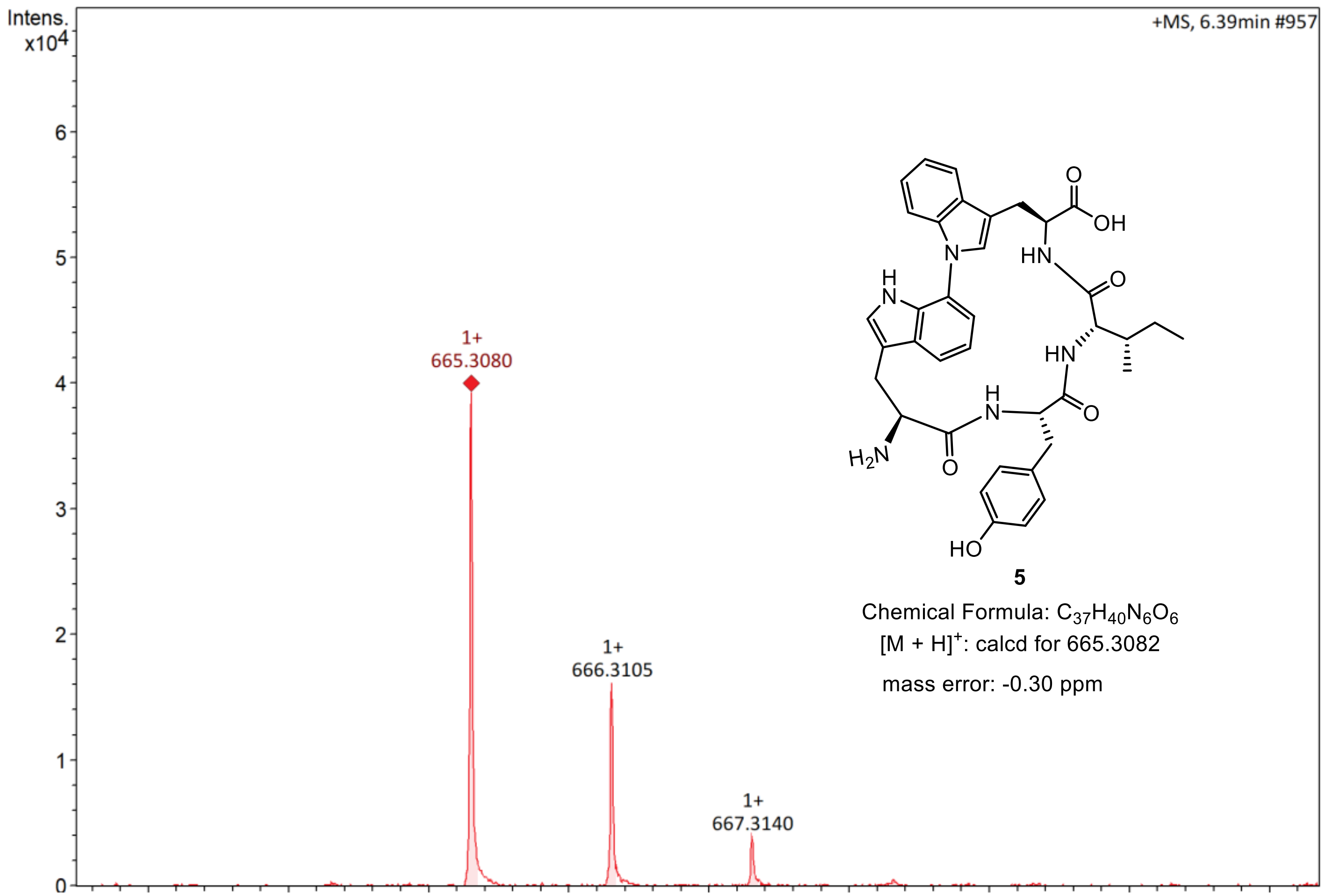

Figure S20. HPLC-ESI-QTOF-HRMS analysis of **5**.

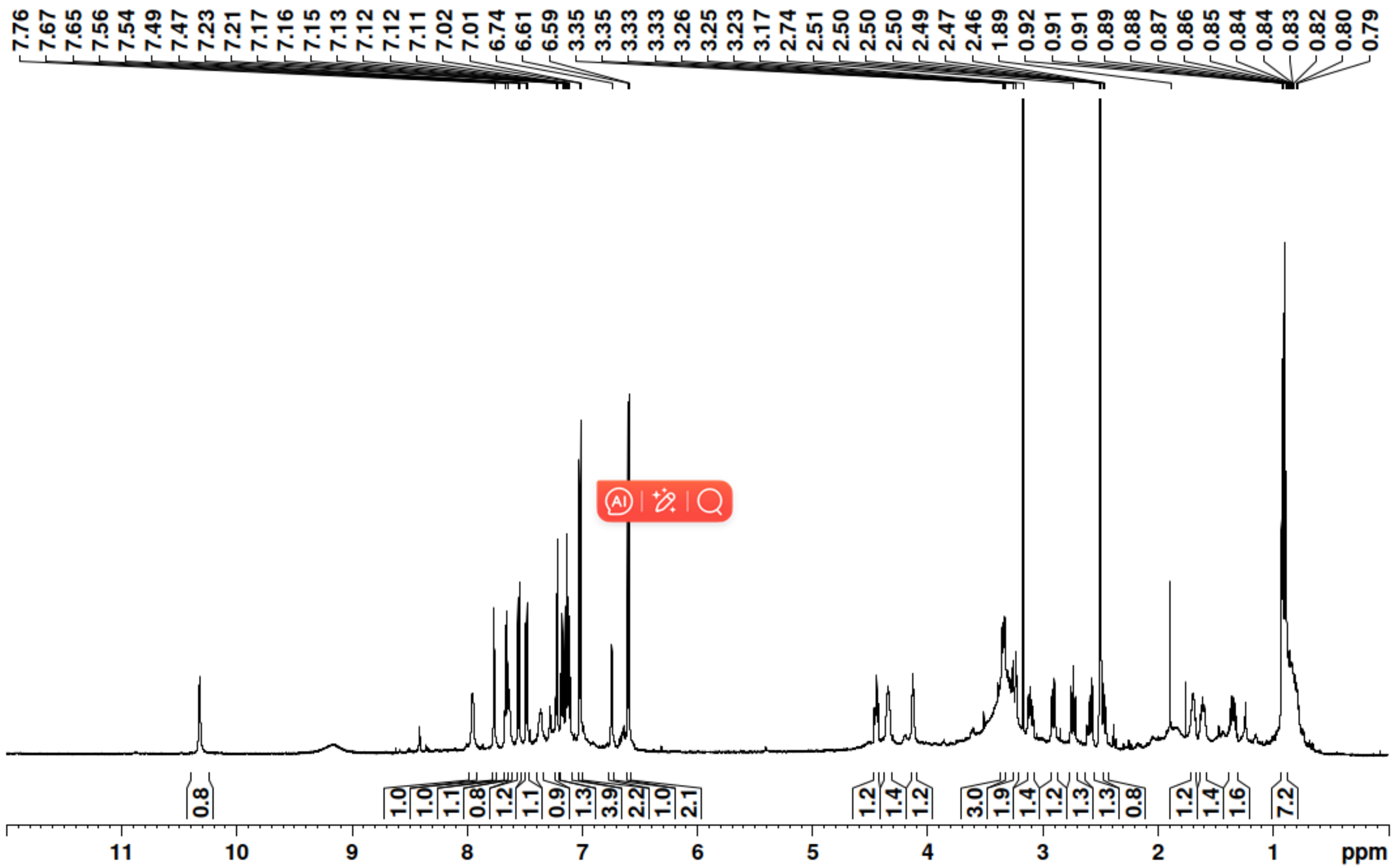

Figure S21. ^{1}H NMR spectrum (600 MHz) of **5** in DMSO-d_6.

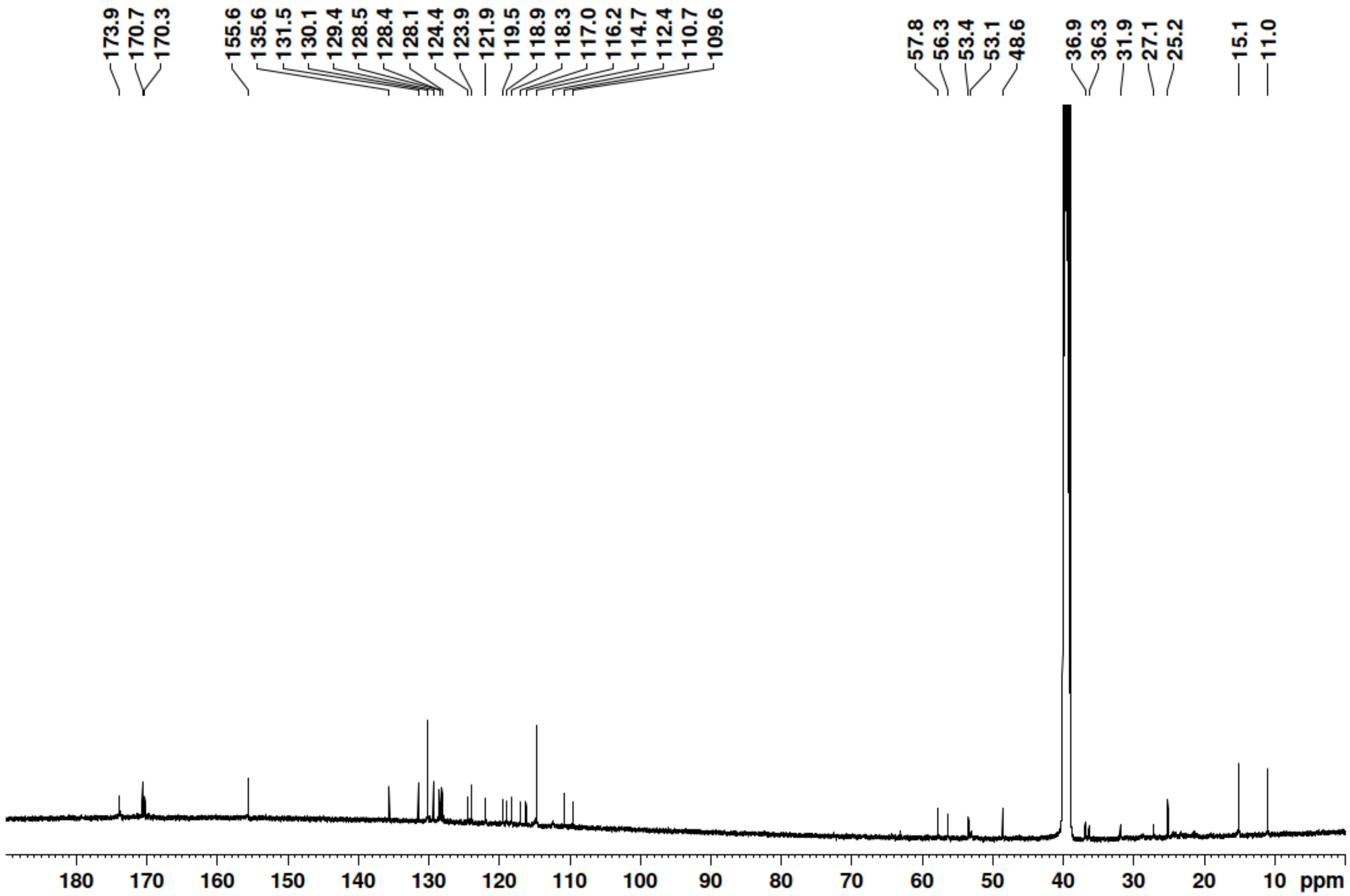

Figure S22. ^{13}C NMR spectrum (125 MHz) of **5** in DMSO-*d*$_6$.

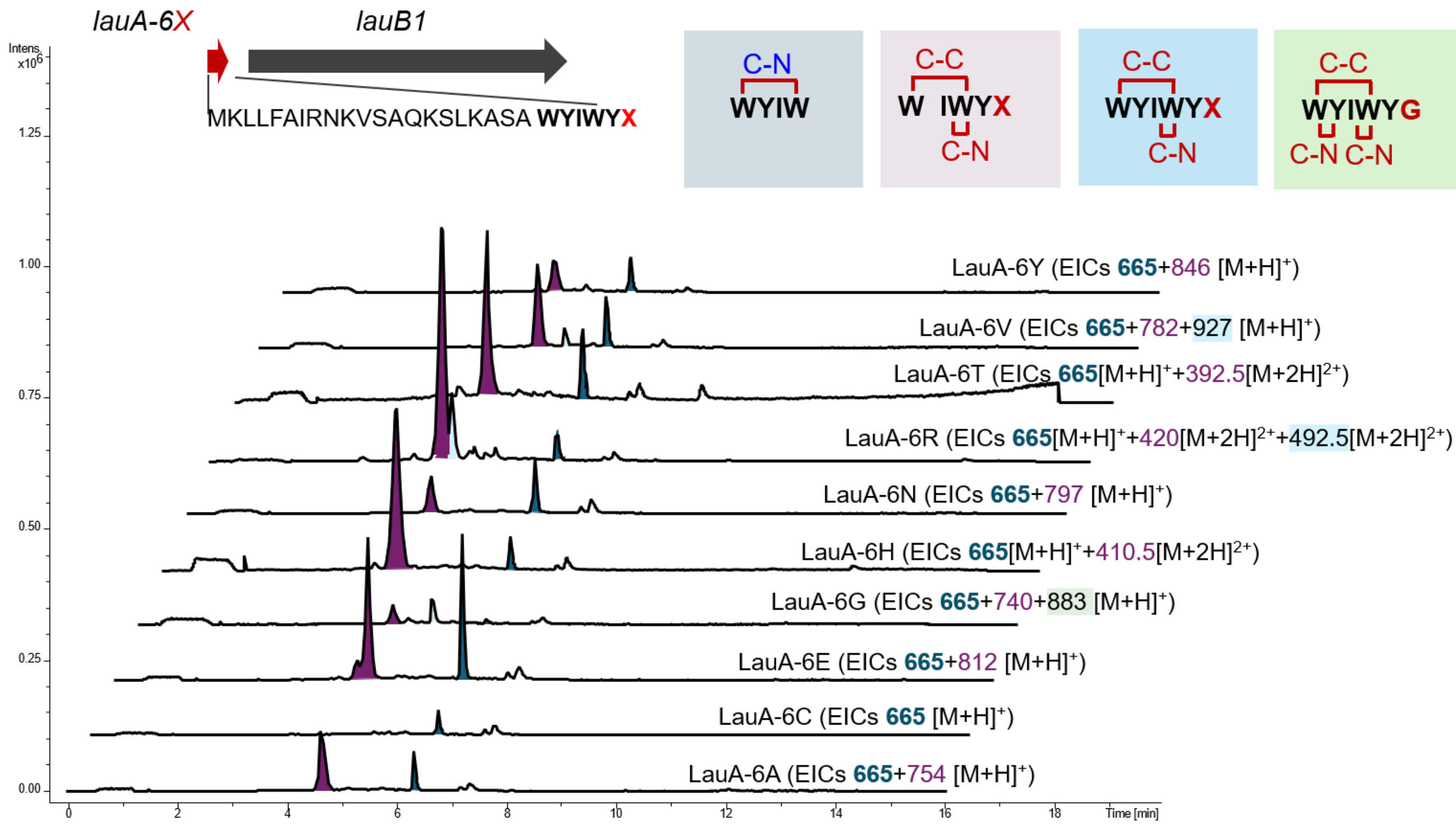

Figure S23. Coexpression of *lauA-6X* variants with *lauB1*. Extracted ion chromatogram of products generated from the coexpression of engineered precursor encoded gene *lauA-6X* with *lauB1*.

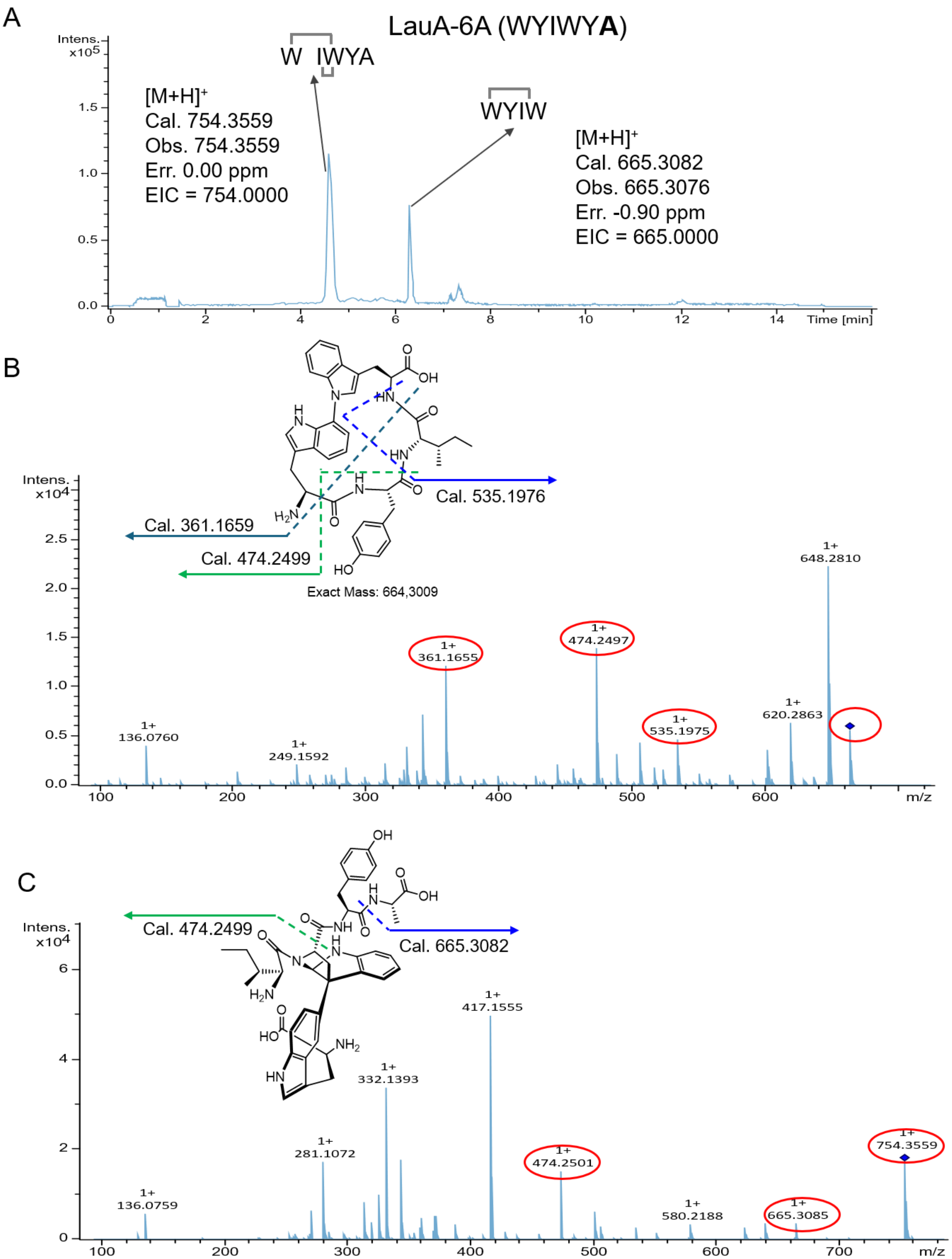

Figure S24. HRMS analysis of extracts of the recombinant *S. albus* strain harboring *lauA*-6A and *lauB1*. A) Extracted ion chromatogram of the product from the coexpression of *lauA*-6A with *lauB1*; B) MS/MS spectrum of the corresponding atropopeptide detected at *m*/*z* 665.3076 $[M+H]^+$ with key fragments of the cyclic tetrapeptide highlighted; C) MS/MS spectrum of the corresponding atropopeptide detected at *m*/*z* 754.3559 $[M+H]^+$ with key fragments indicating the loss of tyrosine at the second position of core peptide highlighted.

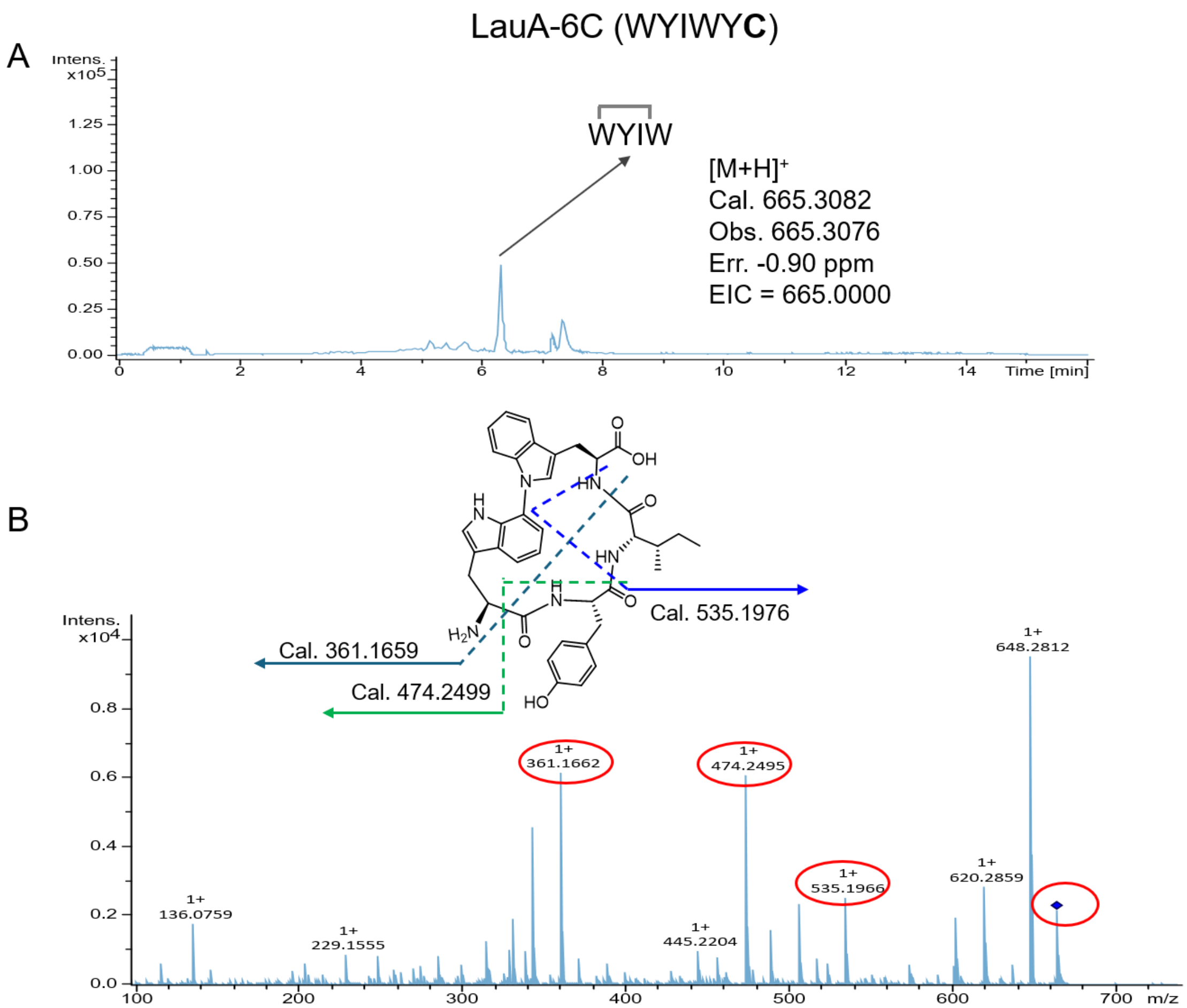

Figure S25. HRMS analysis of extracts of the recombinant *S. albus* strain harboring *lauA*-6C and *lauB1*. A) Extracted ion chromatogram of the product from the coexpression of *lauA*-6C with *lauB1*; B) MS/MS spectrum of the corresponding atropopeptide detected at *m/z* 665.3076 $[M+H]^+$ with key fragments of the cyclic tetrapeptide highlighted.

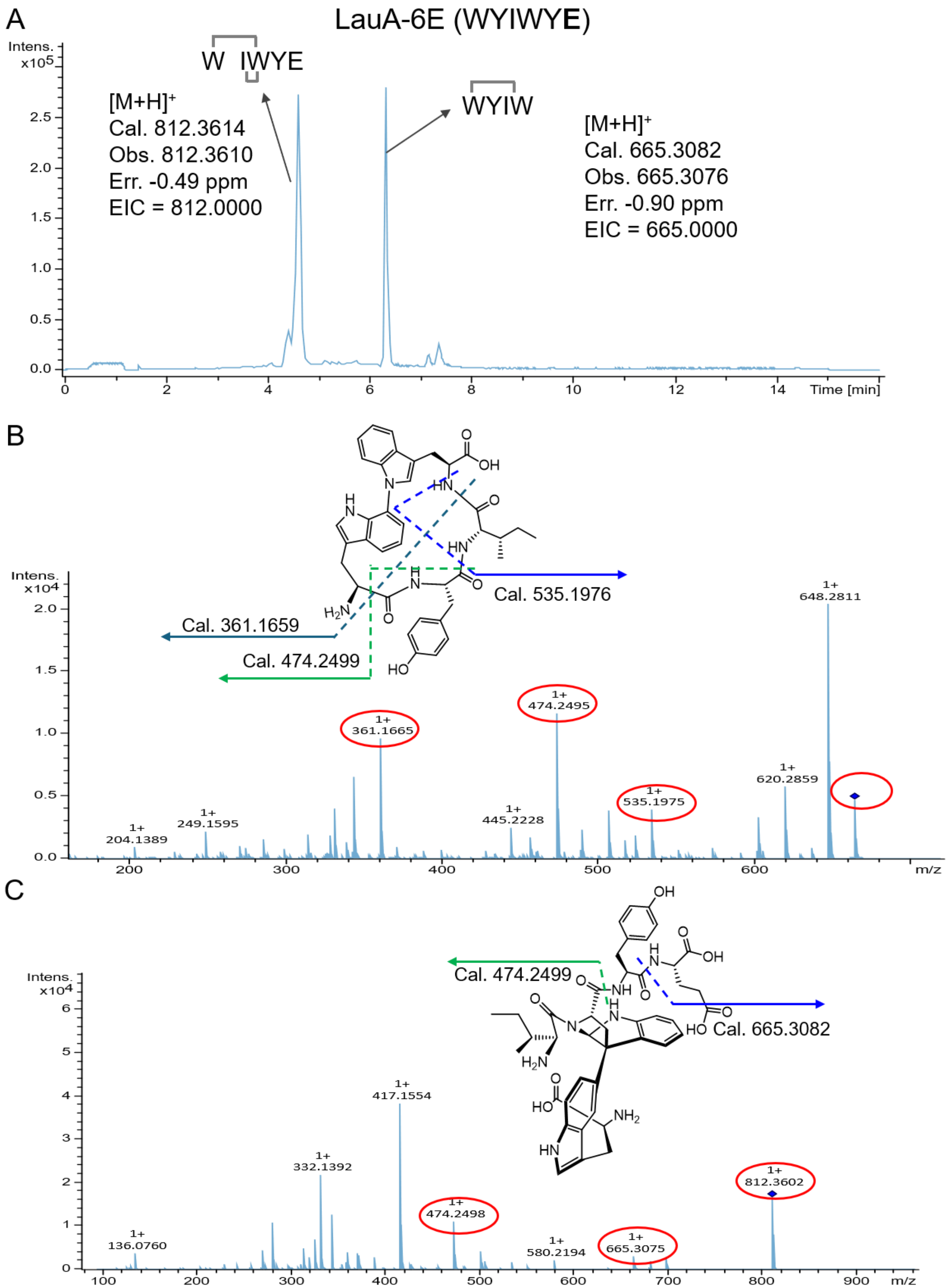

Figure S26. HRMS analysis of extracts of the recombinant *S. albus* strain harboring *lauA*-6E and *lauB1*. A) Extracted ion chromatogram of the product from the coexpression of *lauA*-6E with *lauB1*; B) MS/MS spectrum of the corresponding atropopeptide detected at *m/z* 665.3076 [M+H]+ with key fragments of the cyclic tetrapeptide highlighted; C) MS/MS spectrum of the corresponding atropopeptide detected at *m/z* 812.3610 [M+H]+ with key fragments indicating the loss of tyrosine at the second position of the core peptide highlighted.

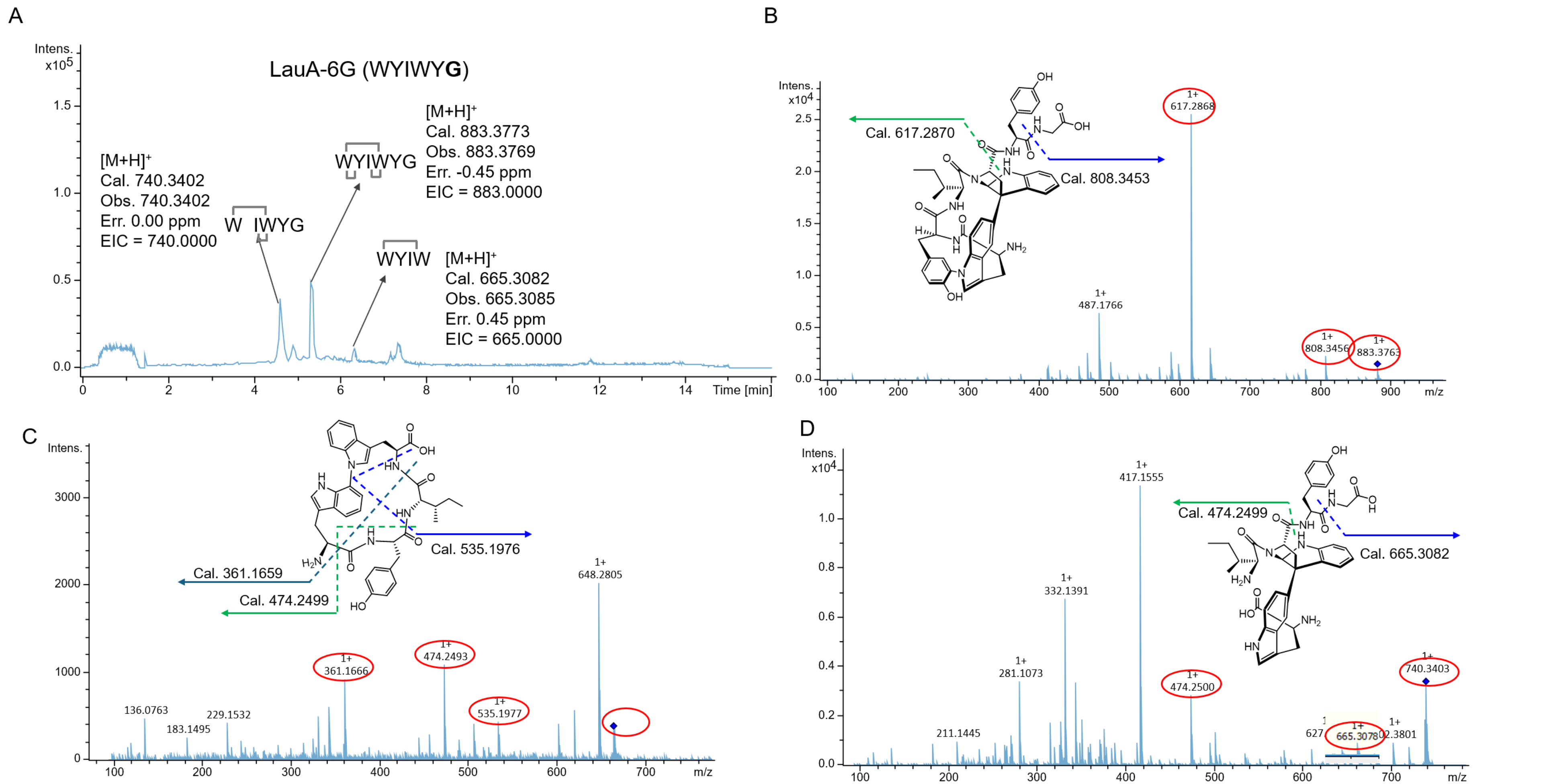

Figure S27. HRMS analysis of extracts of the recombinant *S. albus* strain harboring *lauA*-6G and *lauB1*. A) Extracted ion chromatogram of the product from the coexpression of *lauA*-6G with *lauB1*; B) MS/MS spectrum of the corresponding atropopeptide detected at *m*/*z* 883.3769 $[M+H]^+$; C) MS/MS spectrum of the corresponding atropopeptide detected at *m*/*z* 665.3085 $[M+H]^+$ with key fragments of the cyclic tetrapeptide highlighted; D) MS/MS spectrum of the corresponding atropopeptide detected at *m*/*z* 740.3402 $[M+H]^+$ with key fragments indicating the loss of tyrosine at the second position of the core peptide highlighted.

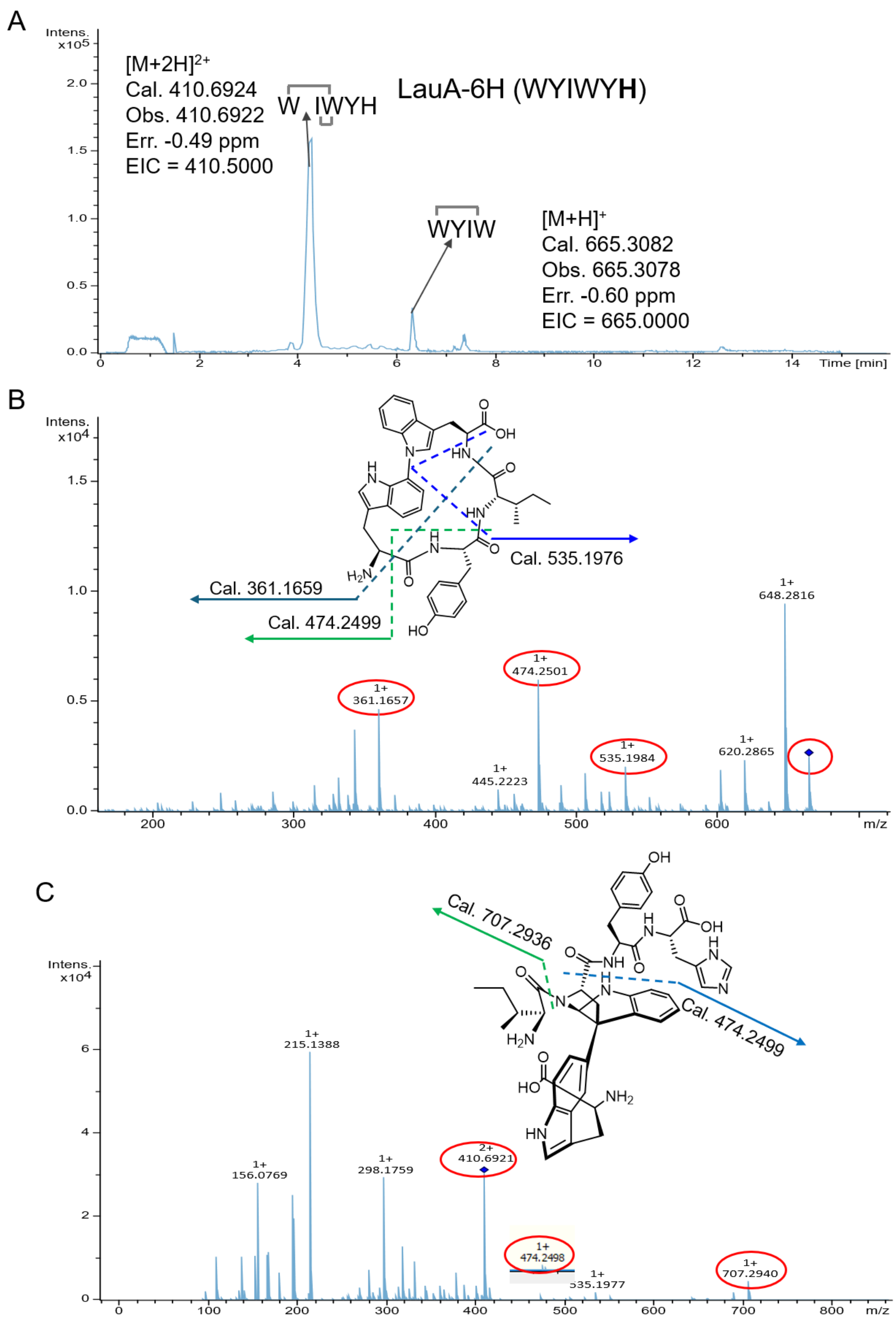

Figure S28. HRMS analysis of extracts of the recombinant *S. albus* strain harboring *lauA*-6H and *lauB1*. A) Extracted ion chromatogram of the product from the coexpression of *lauA*-6H with *lauB1*; B) MS/MS spectrum of the corresponding atropopeptide detected at *m/z* 665.3078 $[M+H]^+$ with key fragments of the cyclic tetrapeptide highlighted; C) MS/MS spectrum of the corresponding atropopeptide detected at *m/z* 410.6922 $[M+2H]^{2+}$ with key fragments indicating the loss of tyrosine at the second position of the core peptide highlighted.

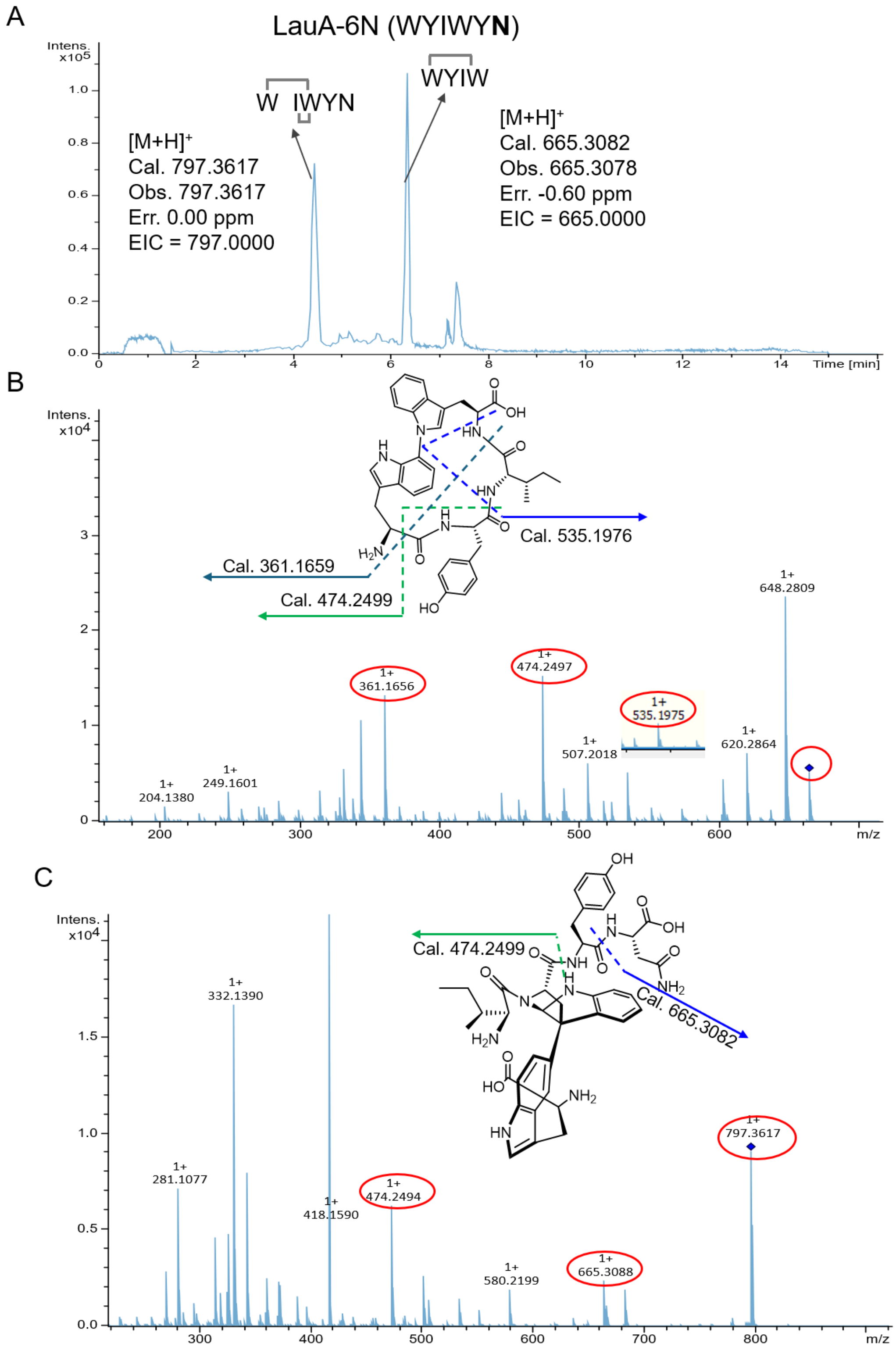

Figure S29. HRMS analysis of extracts of the recombinant *S. albus* strain harboring *lauA*-6N and *lauB1*. A) Extracted ion chromatogram of the product from the coexpression of *lauA*-6N with *lauB1*; B) MS/MS spectrum of the corresponding atropopeptide detected at *m/z* 665.3078 $[M+H]^+$ with key fragments of the cyclic tetrapeptide highlighted; C) MS/MS spectrum of the corresponding atropopeptide detected at *m/z* 797.3617 $[M+H]^+$ with key fragments indicating the loss of tyrosine at the second position of the core peptide highlighted.

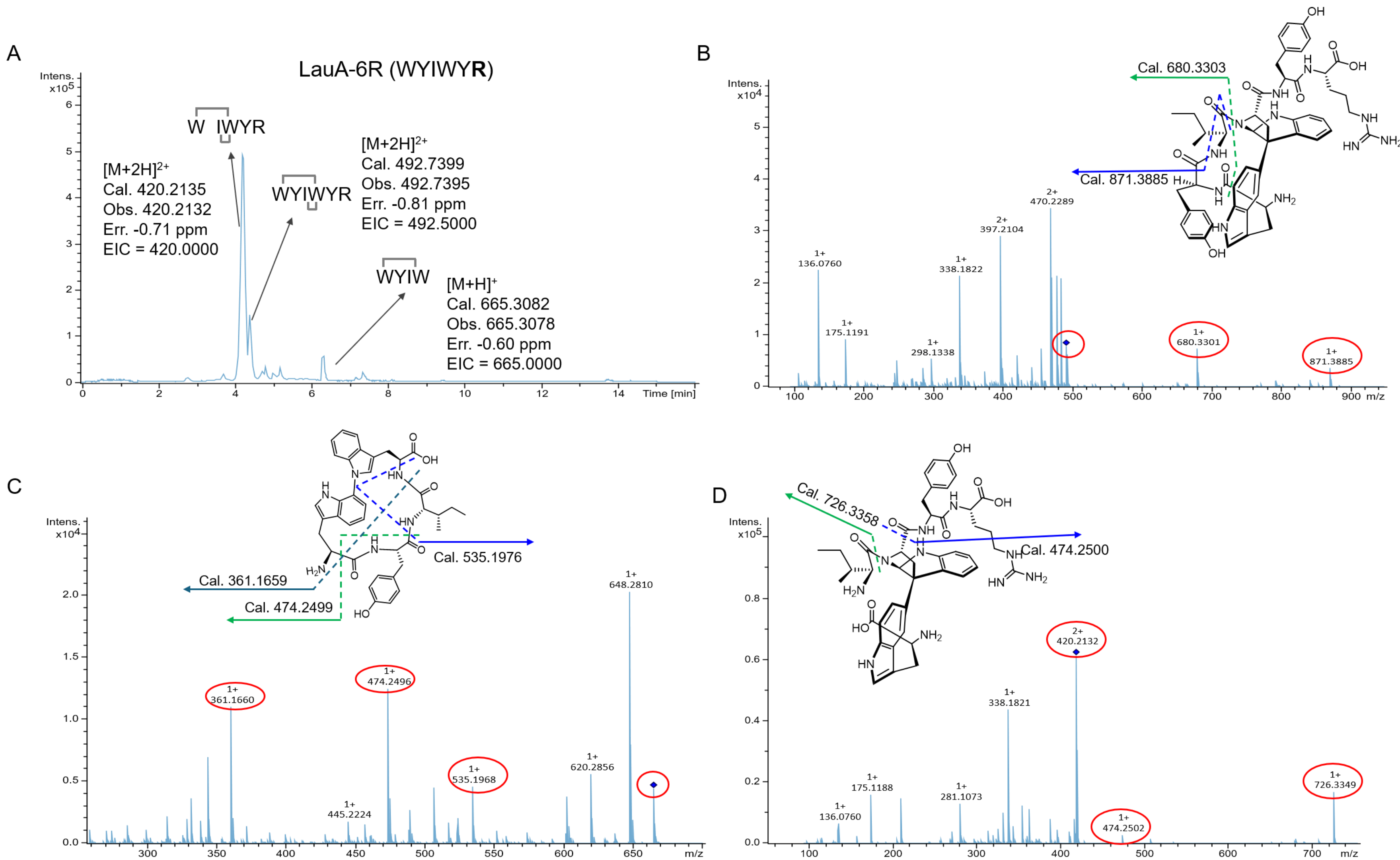

Figure S30. HRMS analysis of extracts of the recombinant *S. albus* strain harboring *lauA*-6R and *lauB1*. A) Extracted ion chromatogram of the product from the coexpression of *lauA*-6R with *lauB1*; B) MS/MS spectrum of the corresponding atropopeptide detected at *m*/*z* 492.7395 $[M+2H]^{2+}$; C) MS/MS spectrum of the corresponding atropopeptide detected at *m*/*z* 665.3078 $[M+H]^{+}$ with key fragments of the cyclic tetrapeptide highlighted; D) MS/MS spectrum of the corresponding atropopeptide detected at *m*/*z* 420.2132 $[M+2H]^{2+}$ with key fragments indicating the loss of tyrosine at the second position of the core highlighted.

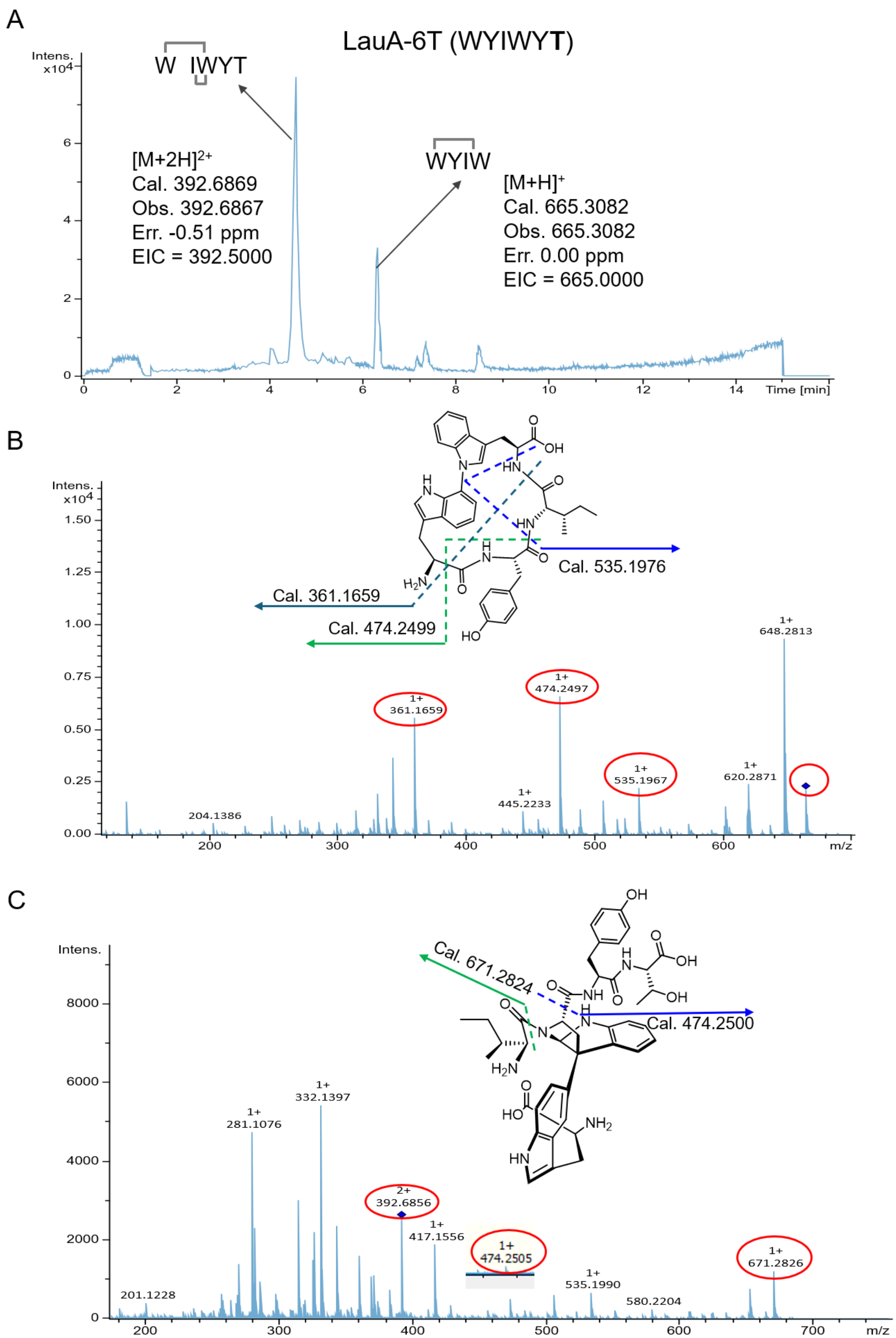

Figure S31. HRMS analysis of extracts of the recombinant *S. albus* strain harboring *lauA*-6T and *lauB1*. A) Extracted ion chromatogram of the product from the coexpression of *lauA*-6T with *lauB1*; B) MS/MS spectrum of the corresponding atropopeptide detected at *m*/*z* 665.3082 $[M+H]^+$ with key fragments of the cyclic tetrapeptide highlighted; C) MS/MS spectrum of the corresponding atropopeptide detected at *m*/*z* 392.6867 $[M+2H]^{2+}$ with key fragments indicating the loss of tyrosine at the second position of the core peptide highlighted.

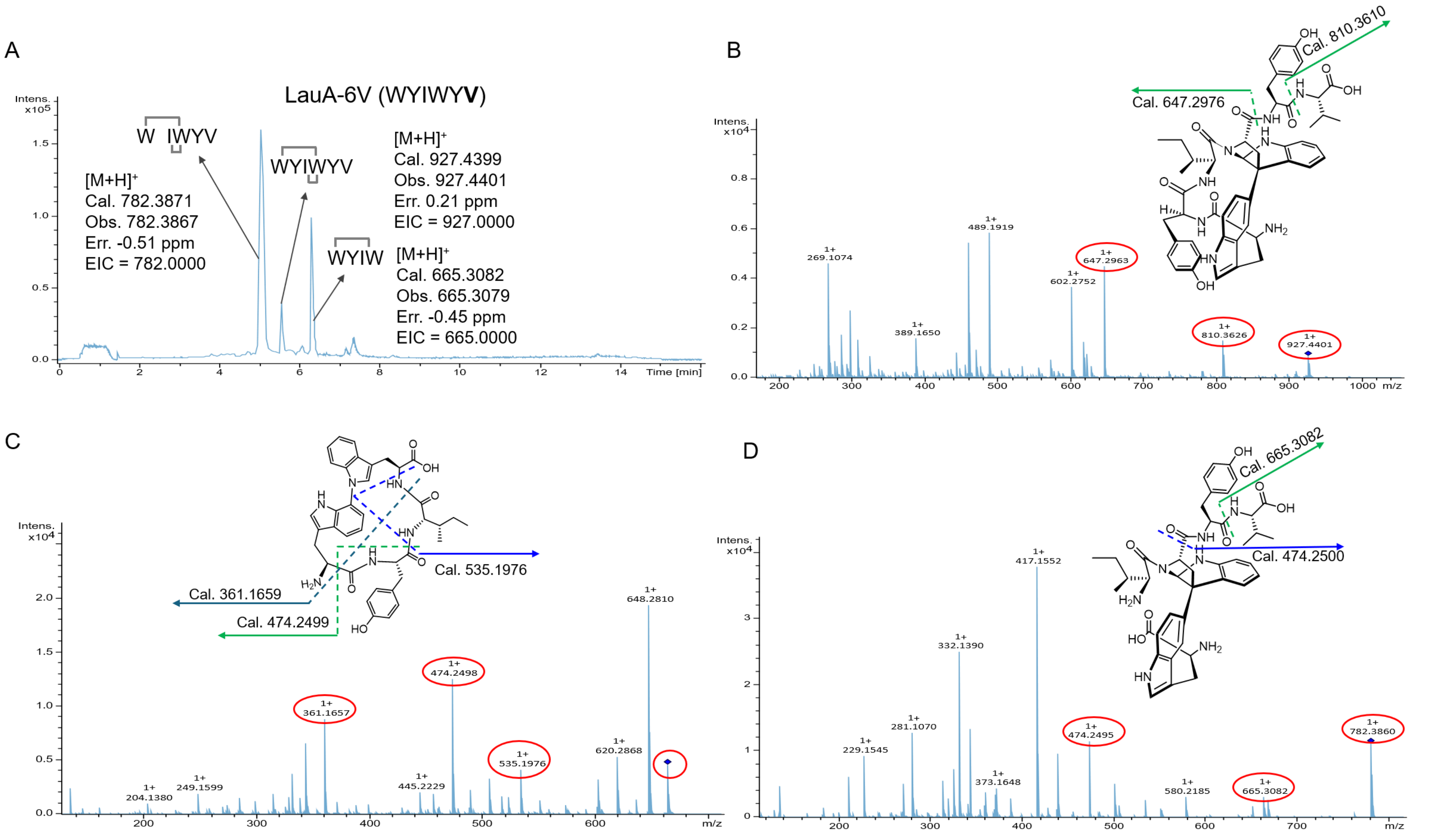

Figure S32. HRMS analysis of extracts of the recombinant *S. albus* strain harboring *lauA*-6V and *lauB1*. A) Extracted ion chromatogram of the product from the coexpression of *lauA*-6V with *lauB1*; B) MS/MS spectrum of the corresponding atropopeptide detected at *m*/*z* 927.4401 $[M+H]^+$; C) MS/MS spectrum of the corresponding atropopeptide detected at *m*/*z* 665.3079 $[M+H]^+$ with key fragments of the cyclic tetrapeptide highlighted; D) MS/MS spectrum of the corresponding atropopeptide detected at *m*/*z* 782.3867 $[M+H]^+$ with key fragments indicating the loss of tyrosine at the second position of the core peptide highlighted.

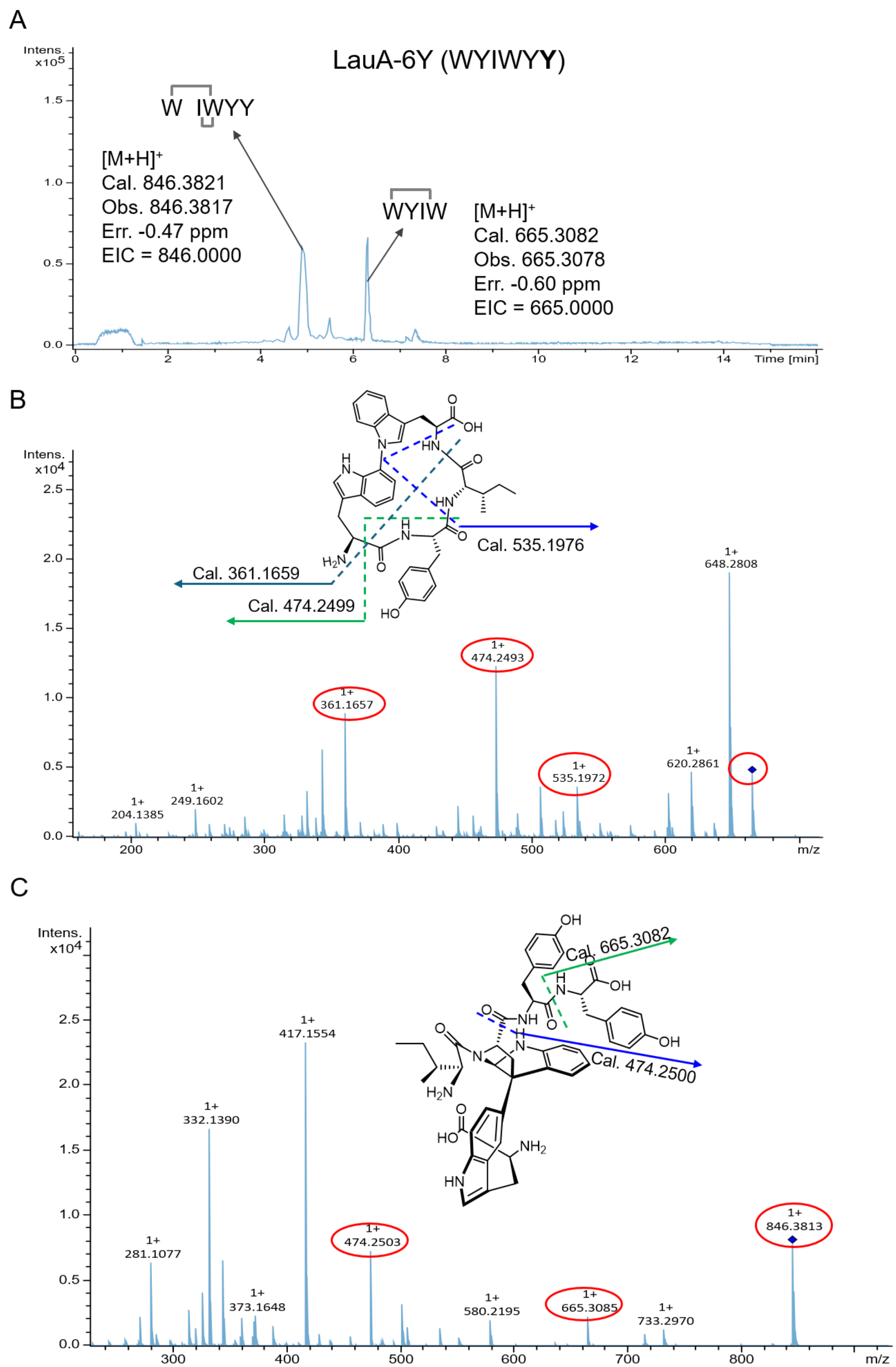

Figure S33. HRMS analysis of extracts of the recombinant *S. albus* strain harboring *lauA*-6Y and *lauB1*. A) Extracted ion chromatogram of the product from the coexpression of *lauA*-6Y with *lauB1*; B) MS/MS spectrum of the corresponding atropopeptide detected at *m/z* 665.3078 $[M+H]^+$ with key fragments of the cyclic tetrapeptide highlighted; C) MS/MS spectrum of the corresponding atropopeptide detected at *m/z* 846.3817 $[M+H]^+$ with key fragments indicating the loss of tyrosine at the second position of the core peptide highlighted.

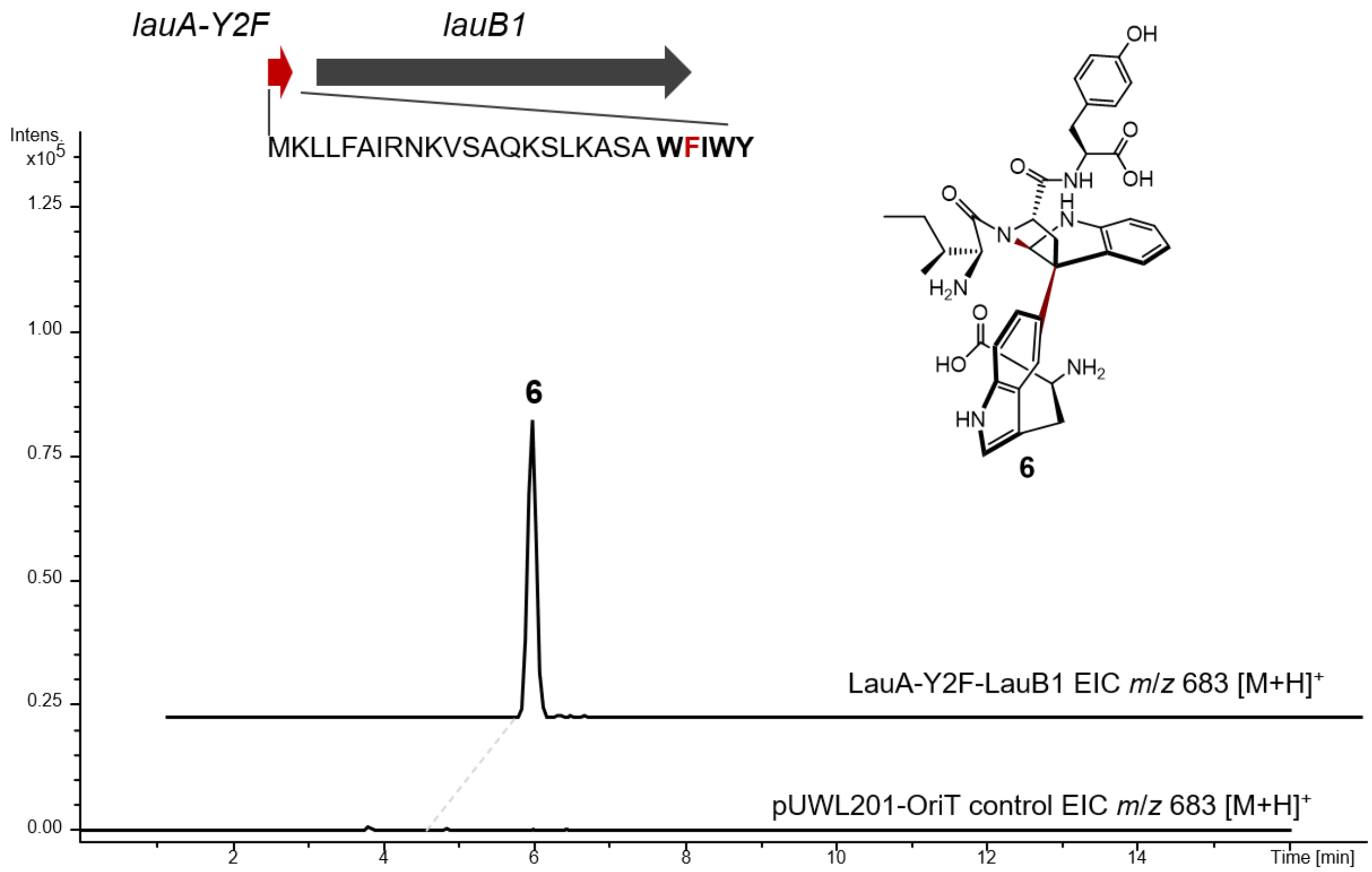

Figure S34. Coexpression of *lauA-Y2F* with *lauB1*. Extracted ion chromatogram of compound **6** from extracts of *S. albus* harboring pUWL201-OriT-LauA-Y2F + LauB1 and *S. albus* harboring pUWL201-OriT as a negative control.

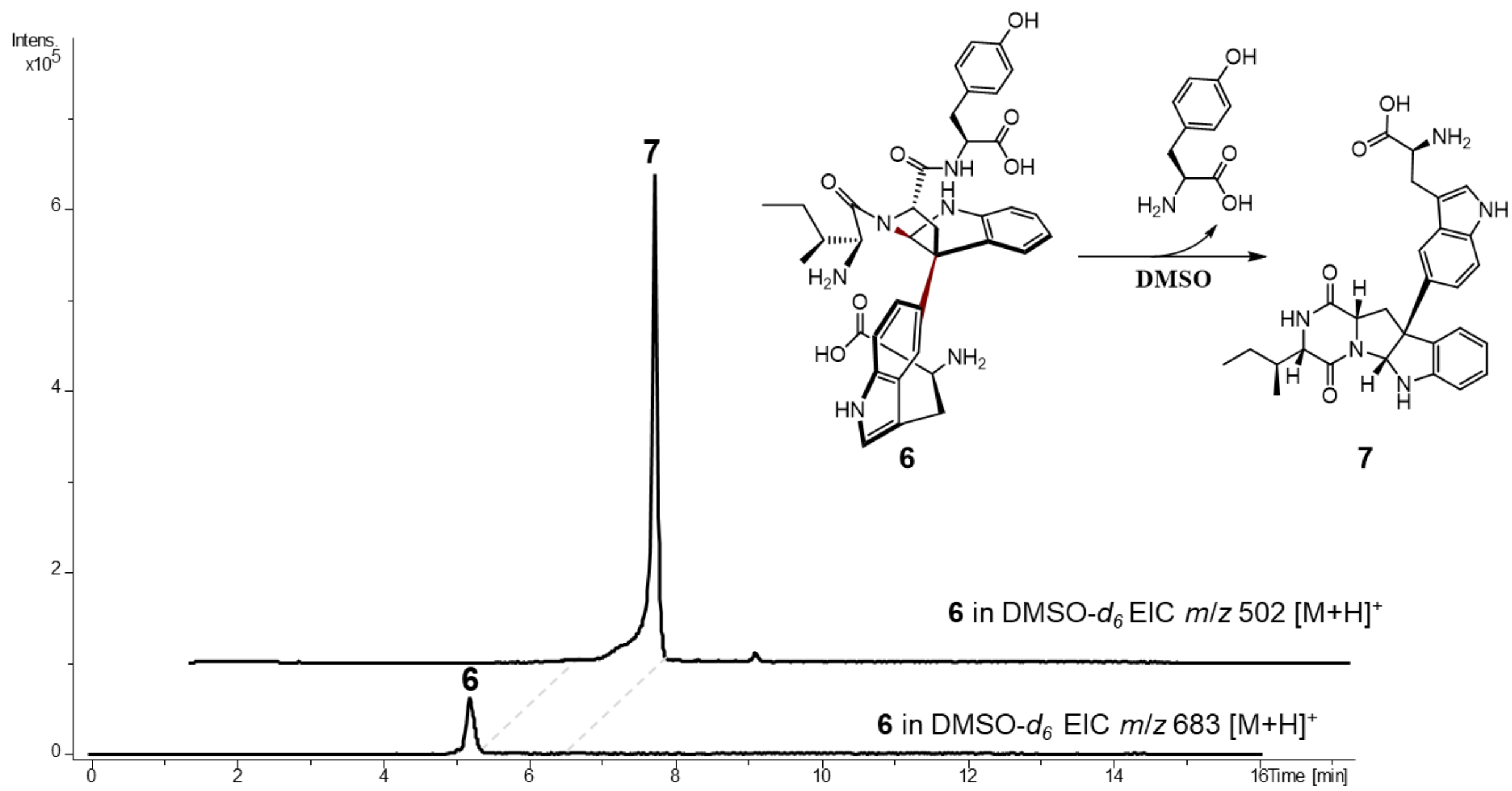

Figure S35. Spontaneous transformation of **6** into **7**. Extracted ion chromatograms of **6** and **7**. Compound **7** was generated from a sample of **6** dissolved in DMSO-d_6 and stored in the NMR tube for 15 days at room temperature.

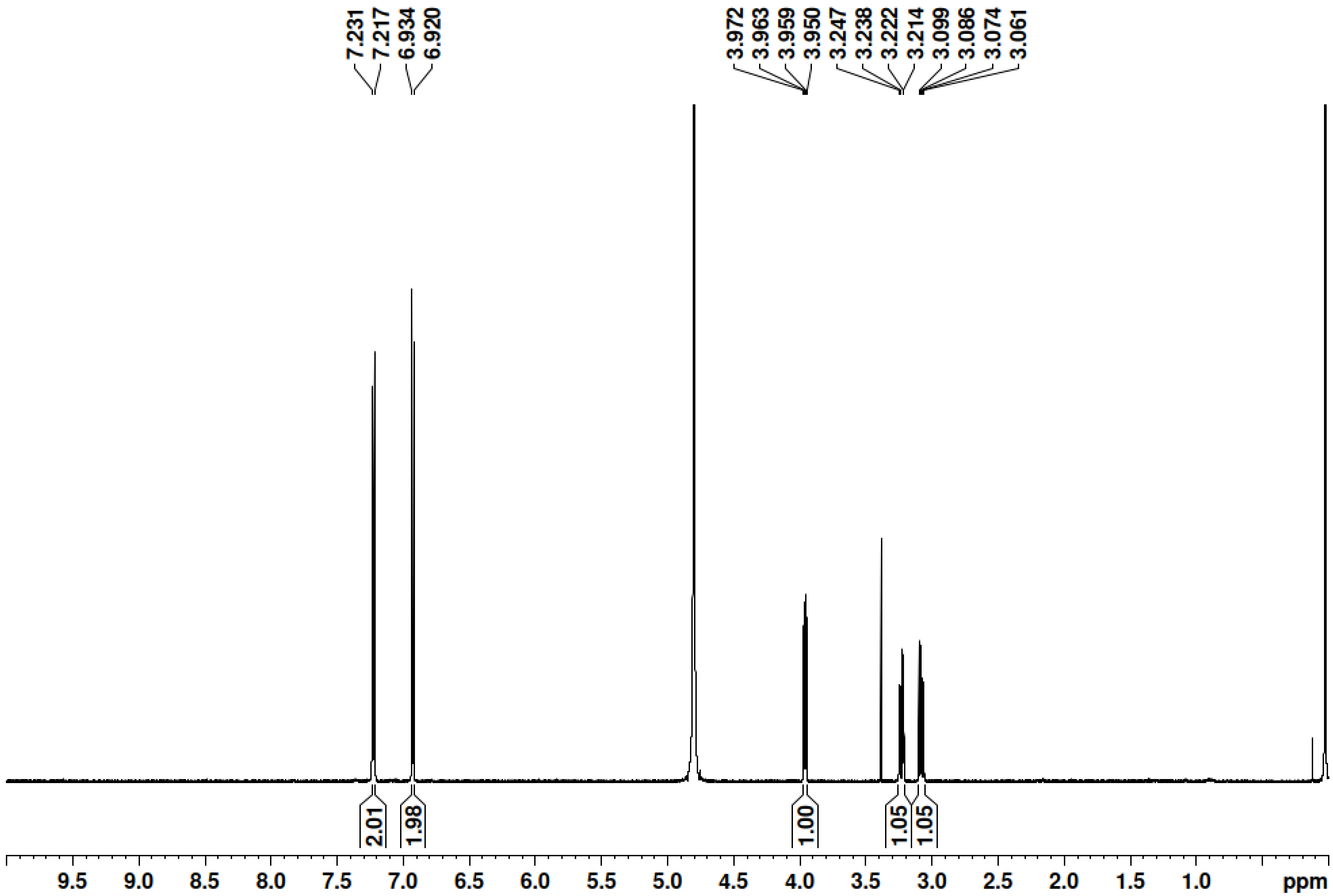

Figure S36. ^{1}H NMR spectrum (600 MHz) of Tyr isolated from NMR sample of **6** in D_2O.

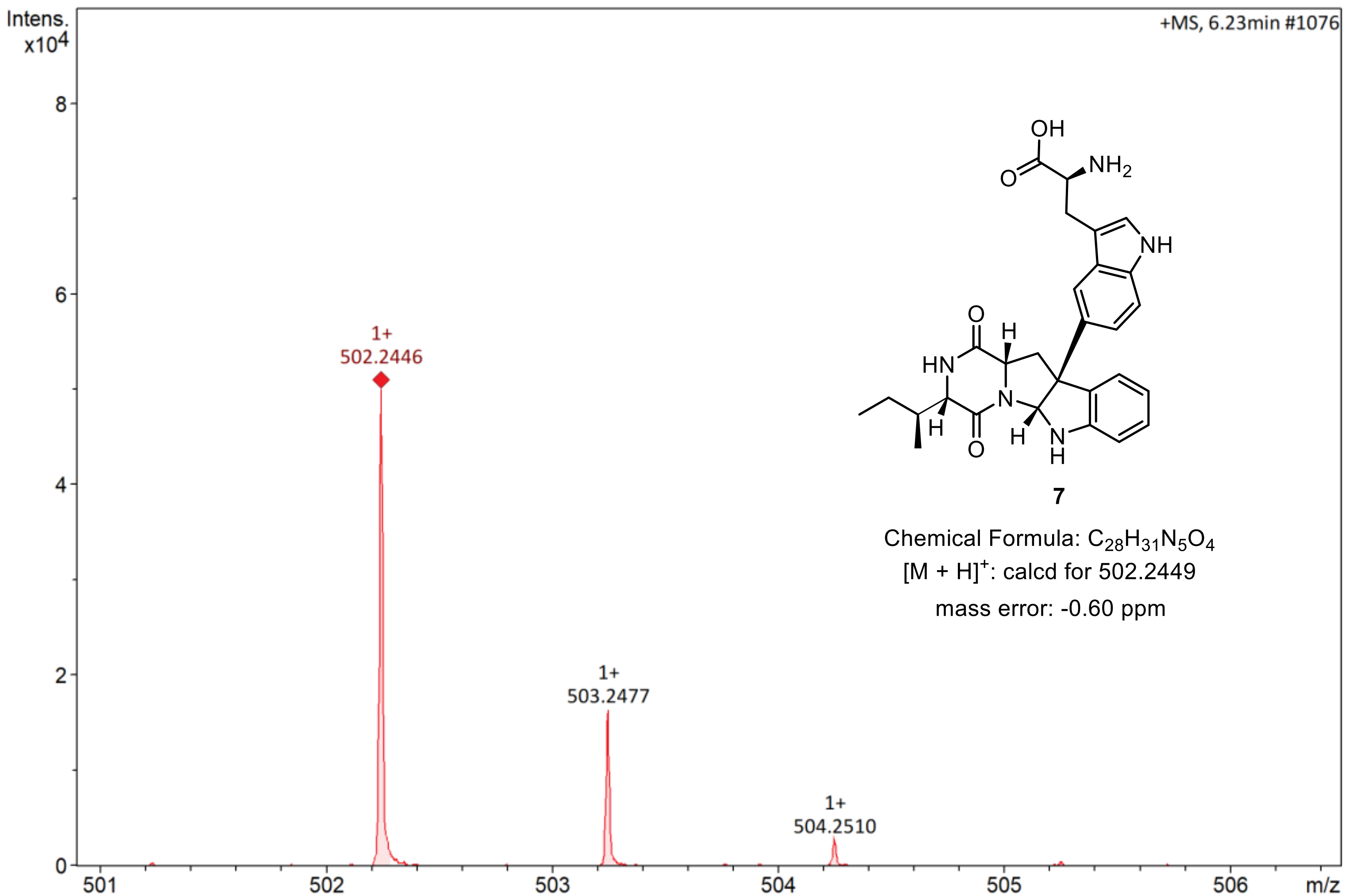

Figure S37. HPLC-ESI-QTOF-HRMS analysis of **7**.

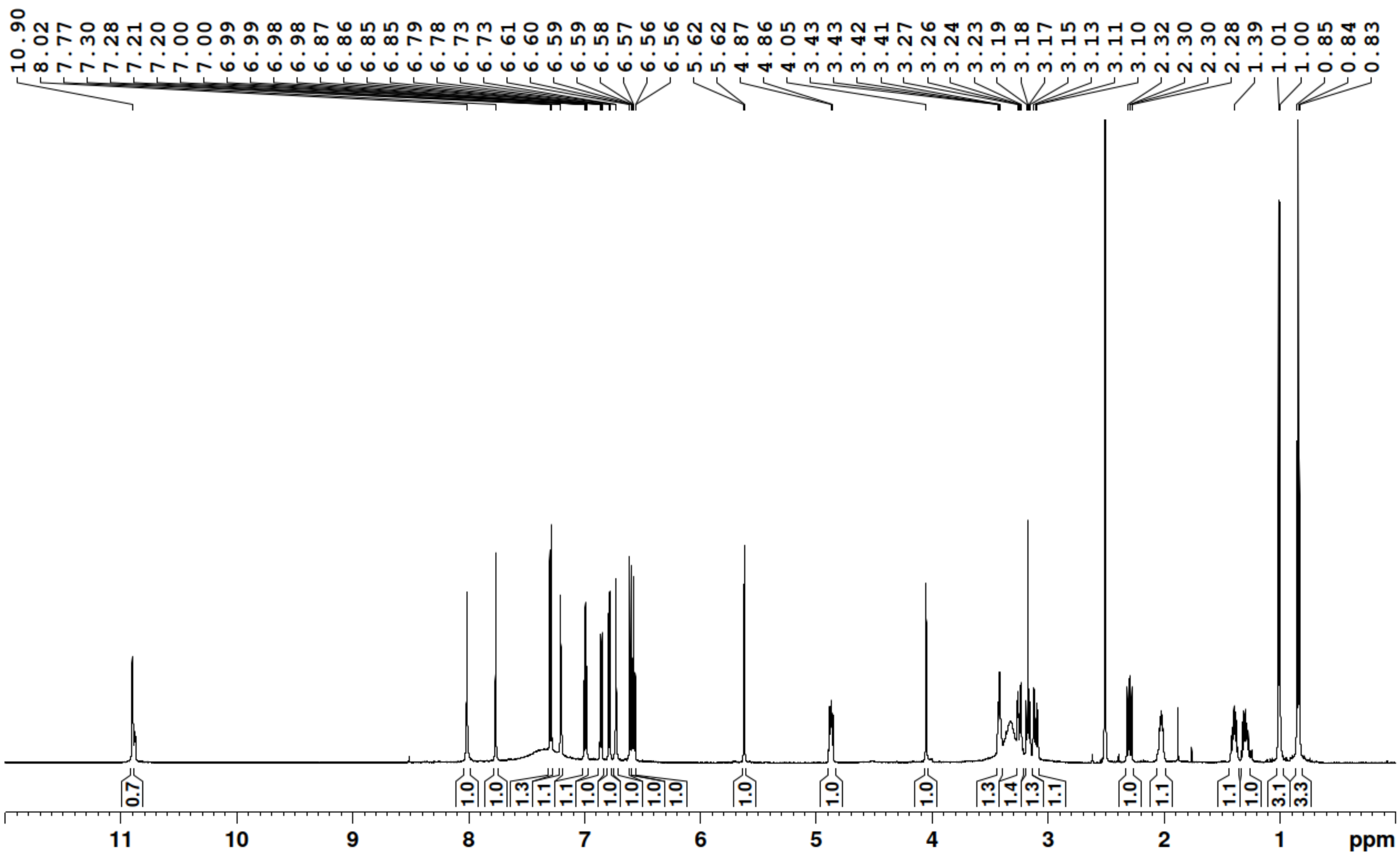

Figure S38. ^{1}H NMR spectrum (600 MHz) of **7** in DMSO-d_6.

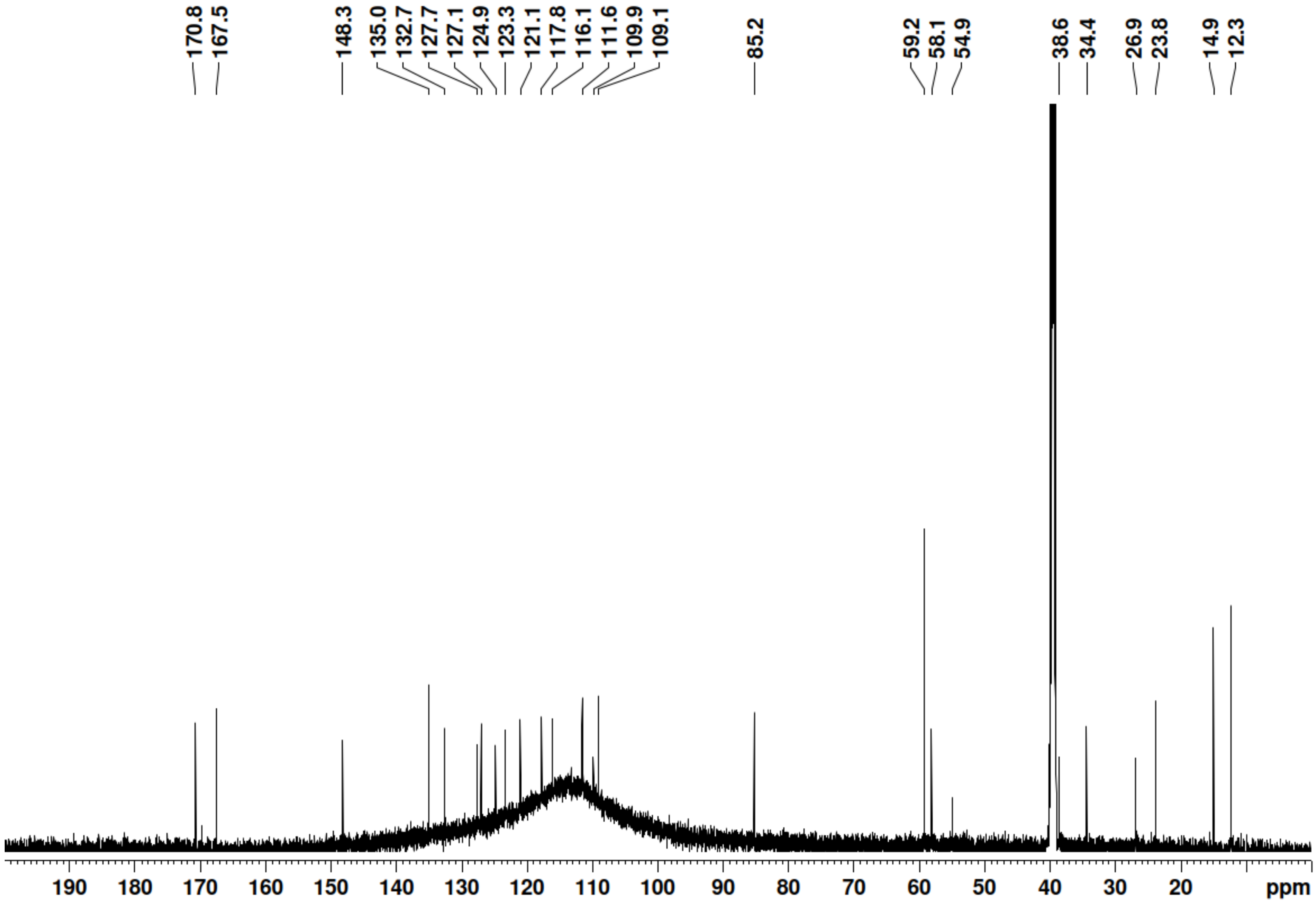

Figure S39. ^{13}C NMR spectrum (150 MHz) of **7** in DMSO-d_6.

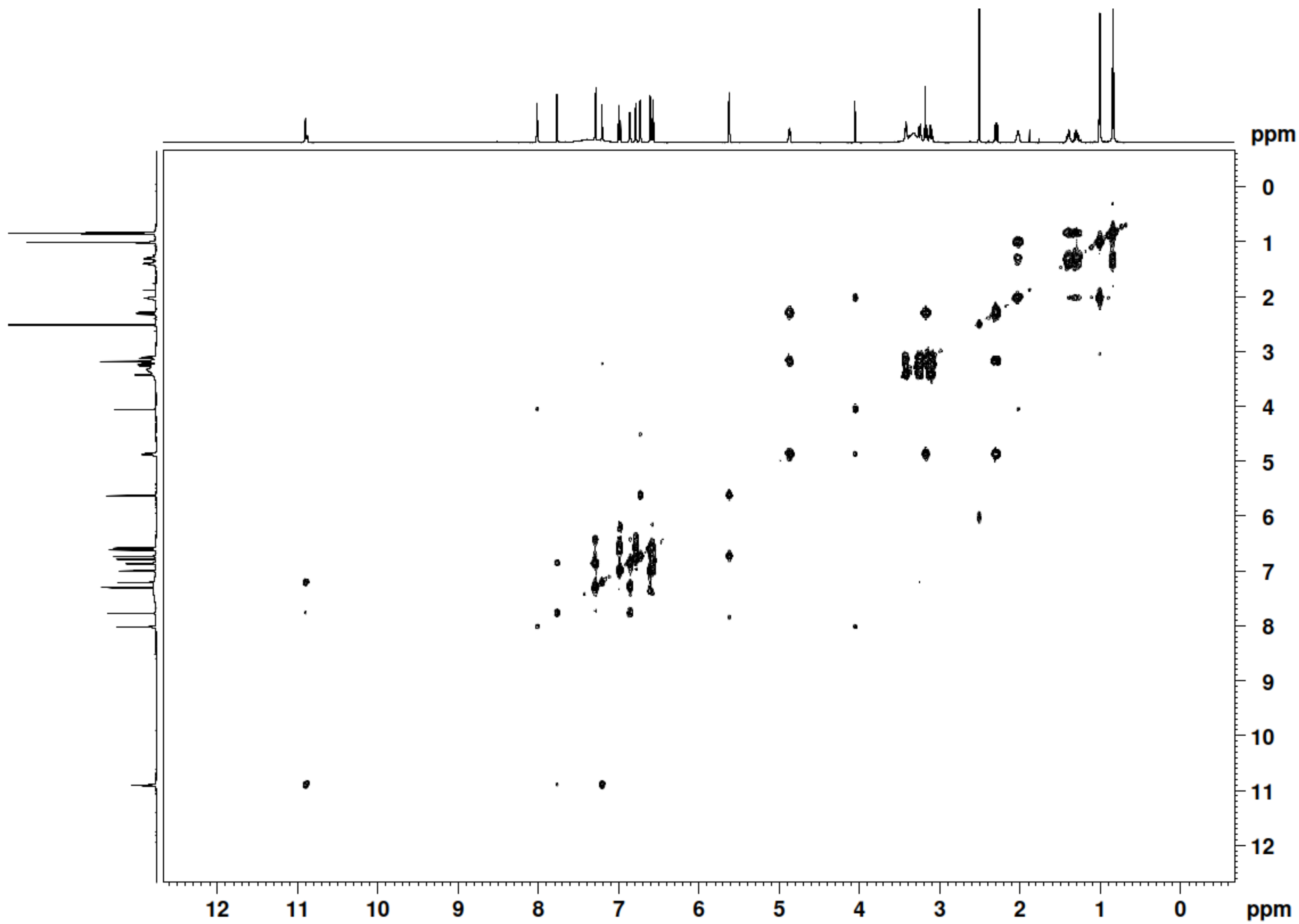

Figure S40. COSY NMR spectrum (600 MHz) of **7** in DMSO-d_6.

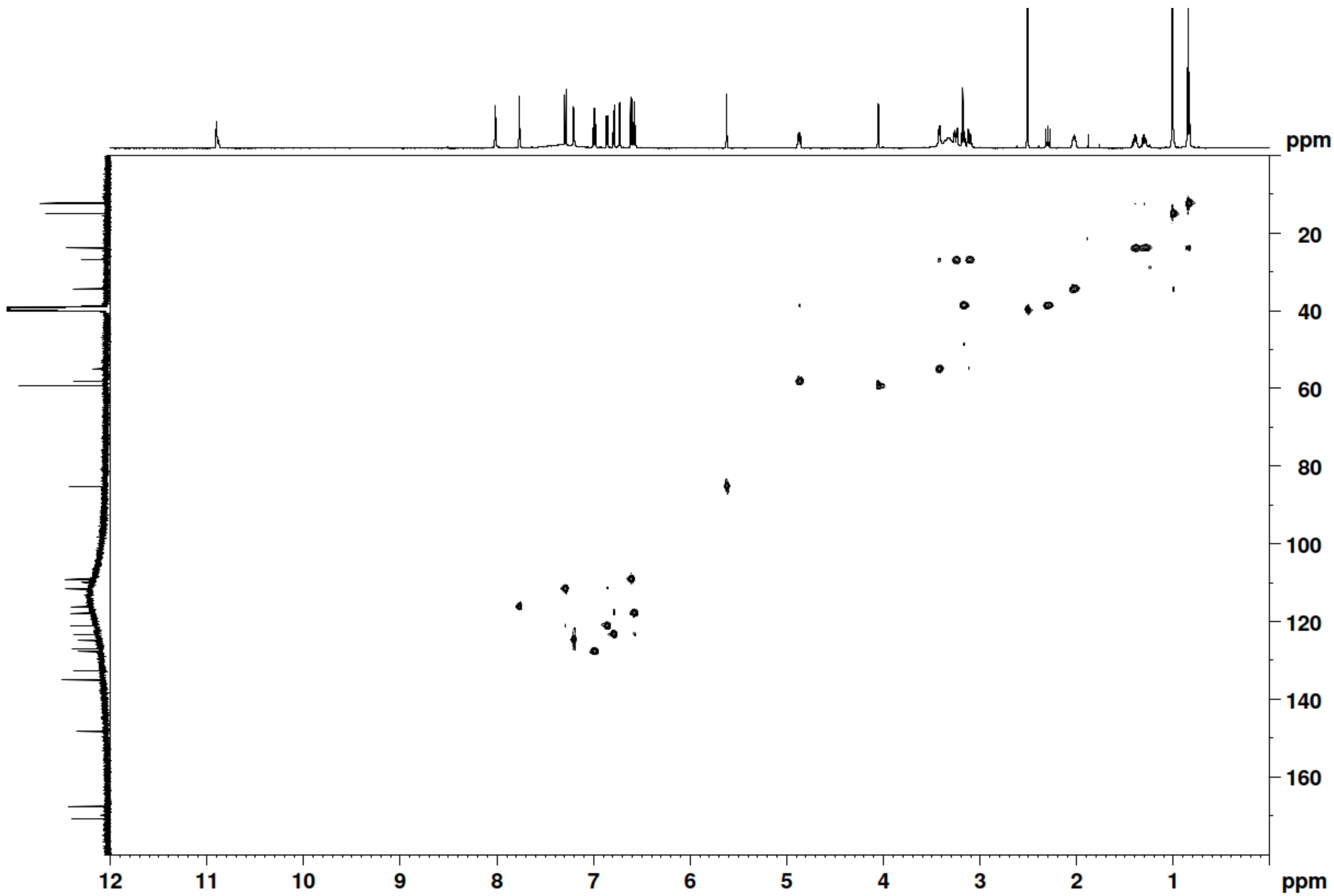

Figure S41. HSQC NMR spectrum (600 MHz) of **7** in DMSO-d_6.

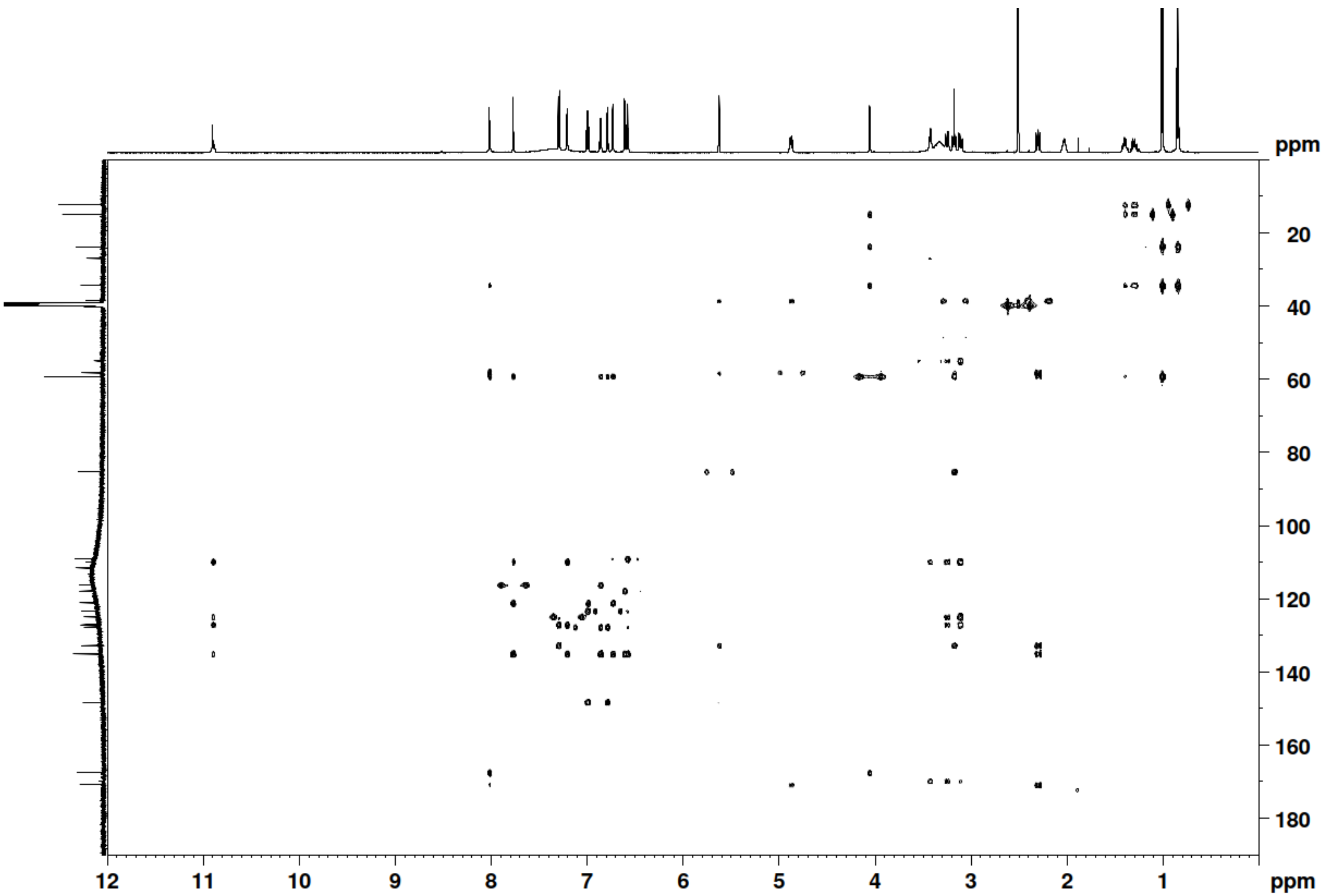

Figure S42. HMBC NMR spectrum (600 MHz) of **7** in DMSO-d_6.

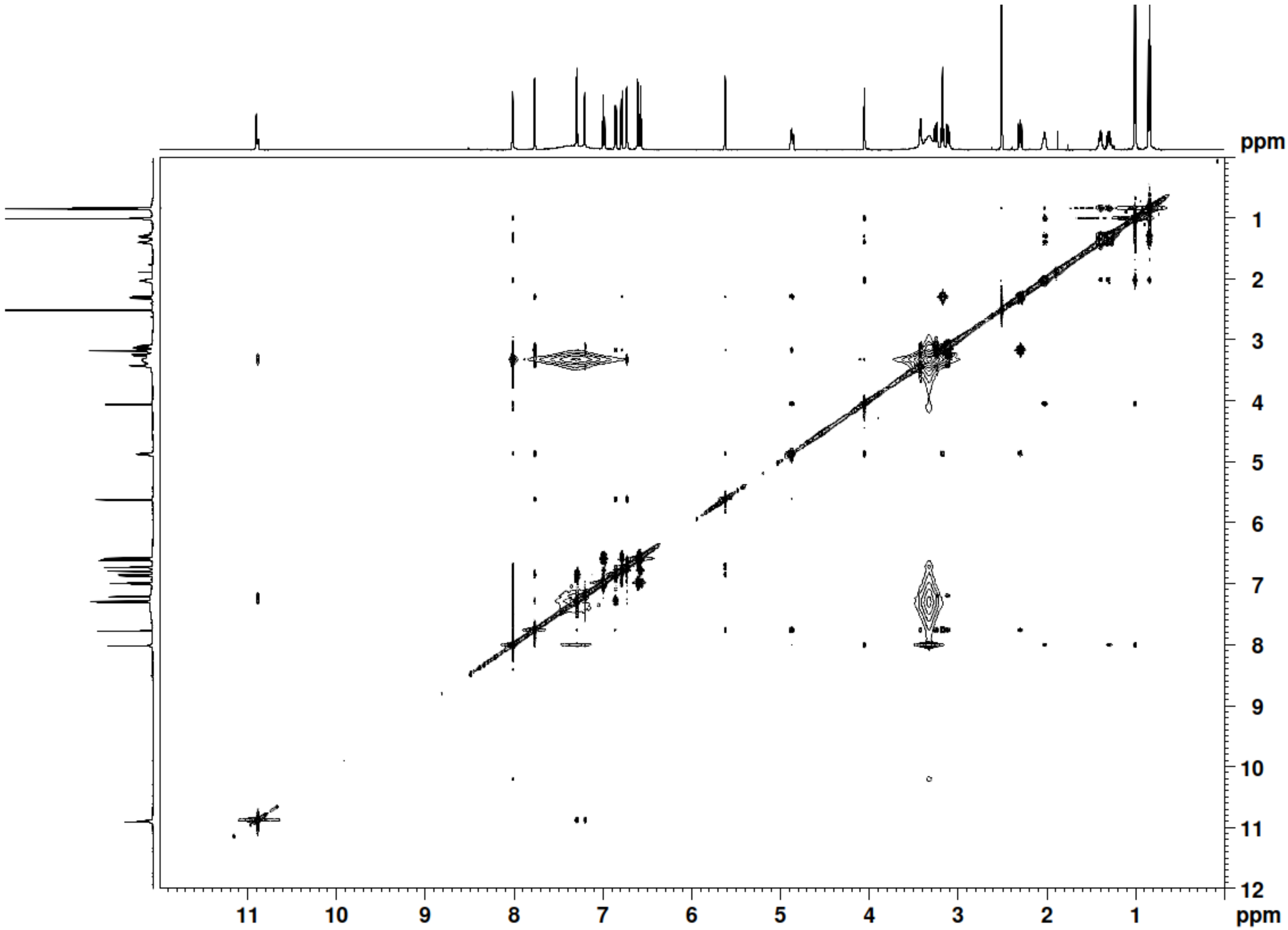

Figure S43. NOESY NMR spectrum (600 MHz) of **7** in DMSO-d_6.

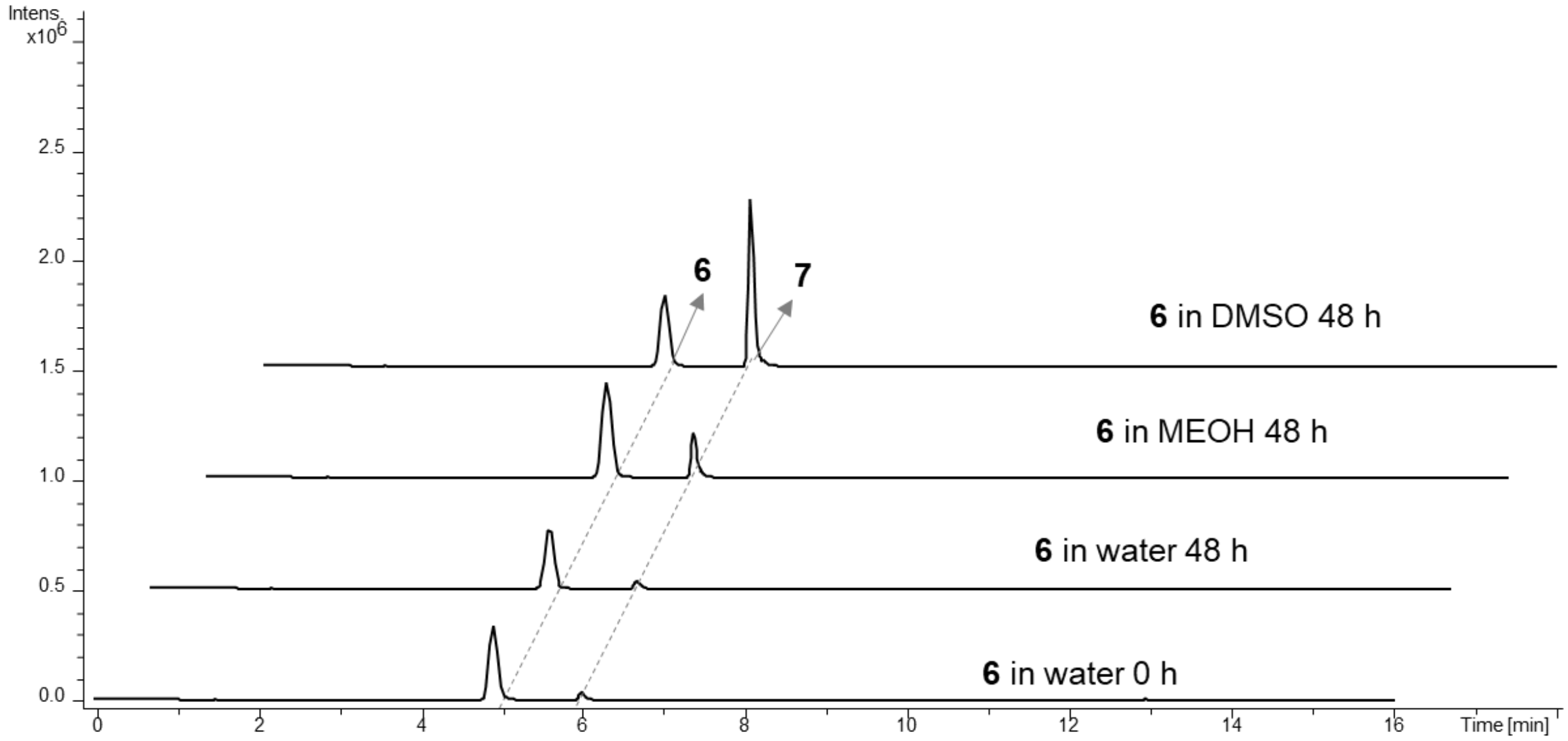

Figure S44. Stability test of **6** in different solvents. Extracted ion chromatogram of **6** and **7** from samples of **6** dissolved in different solvents (**6** in water, in methanol and in DMSO).

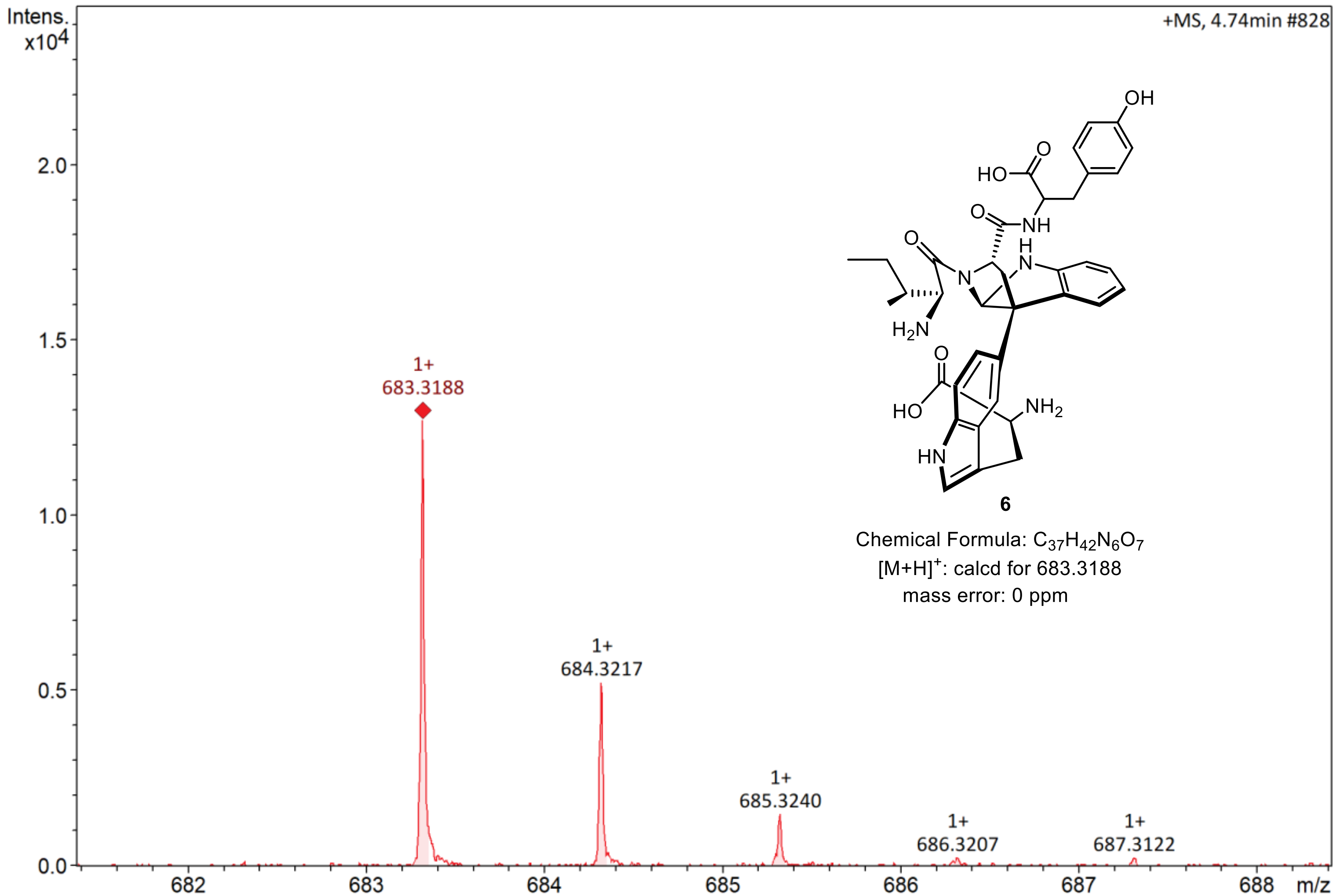

Figure S45. HPLC-ESI-QTOF-HRMS analysis of **6**.

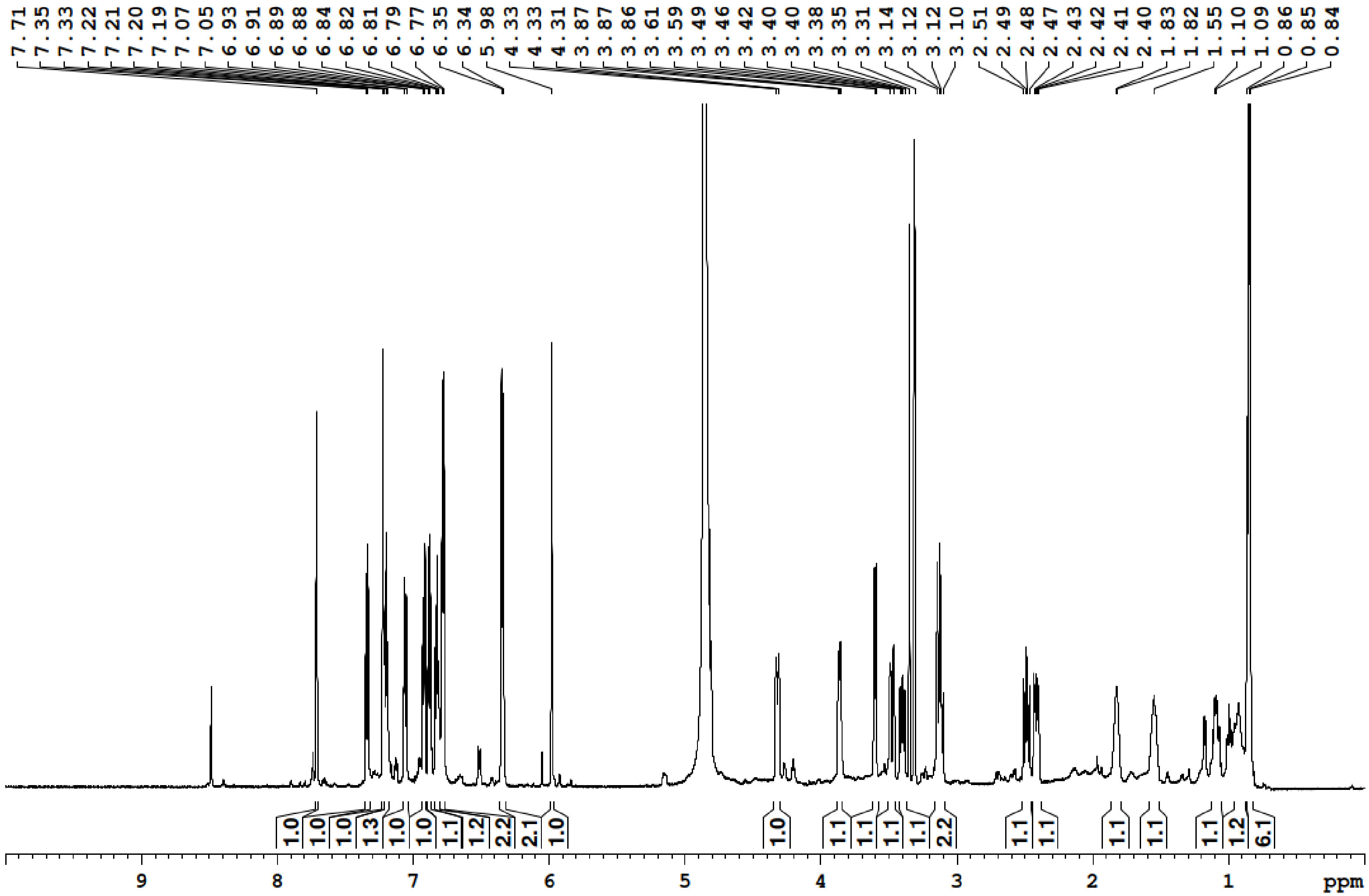

Figure S46. ^{1}H NMR spectrum (600 MHz) of **6** in MeOD.

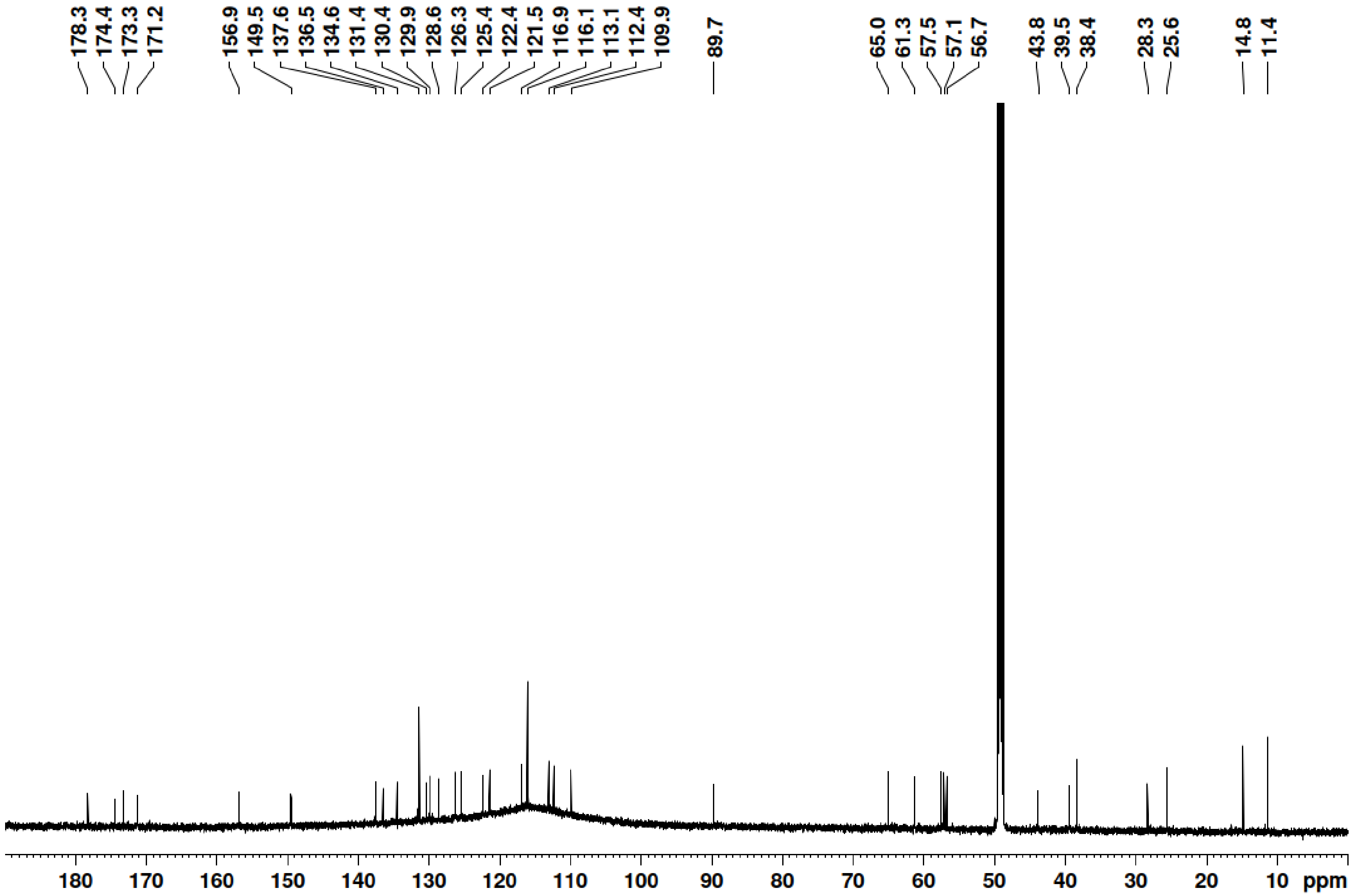

Figure S47. ^{13}C NMR spectrum (125 MHz) of **6** in MeOD.

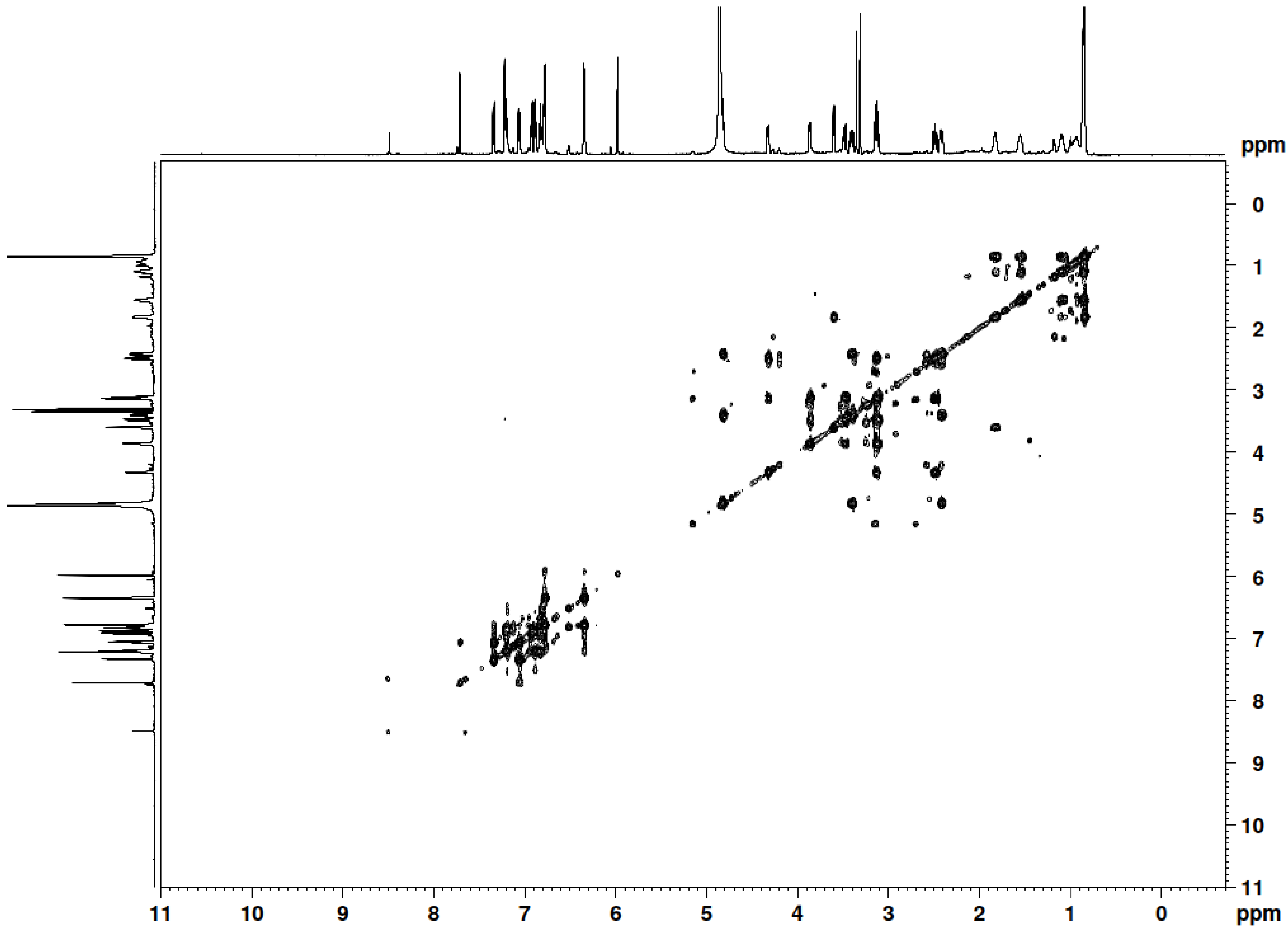

Figure S48. COSY NMR spectrum (500 MHz) of **6** in MeOD.

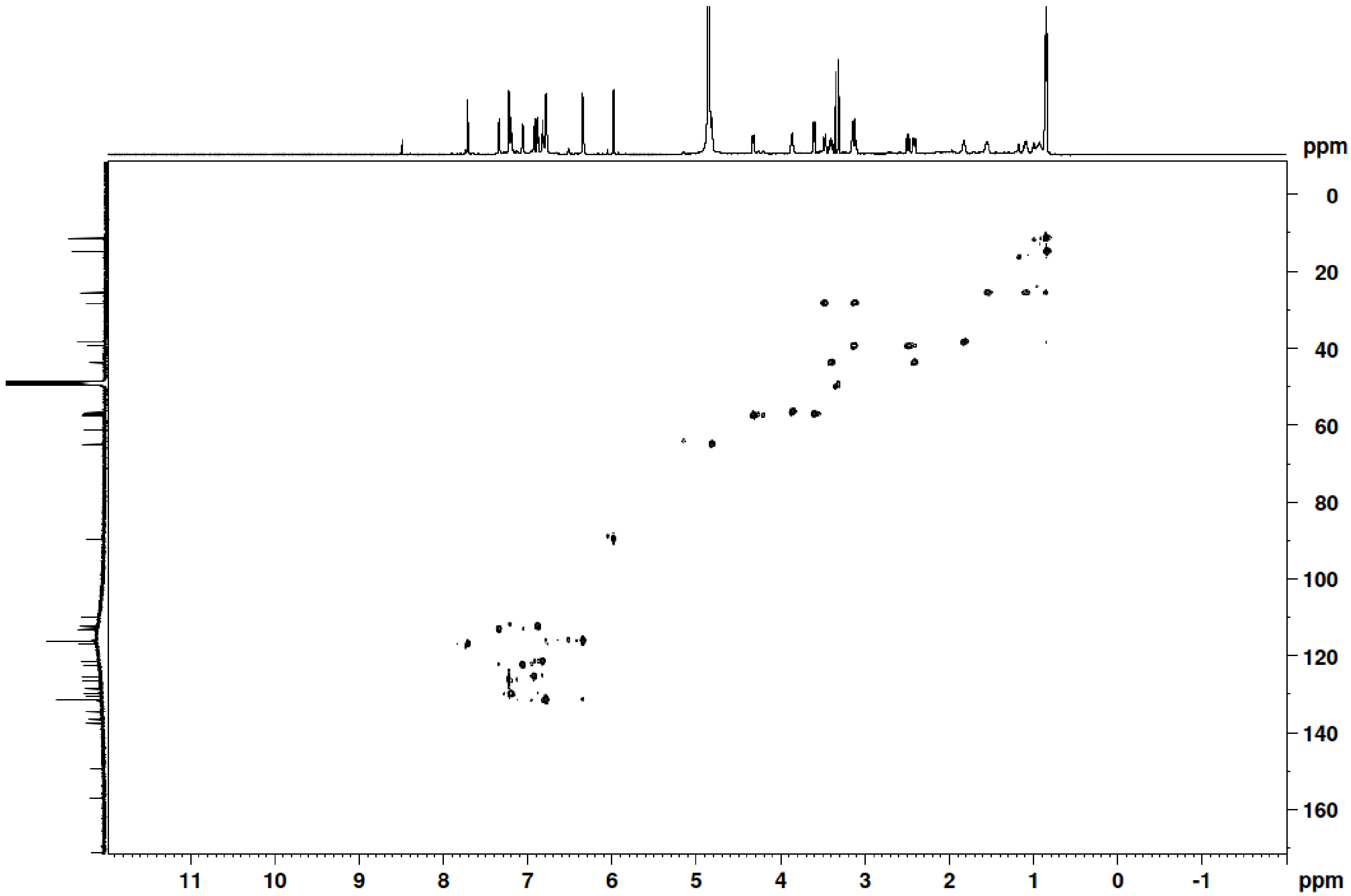

Figure S49. HSQC NMR spectrum (500 MHz) of **6** in MeOD.

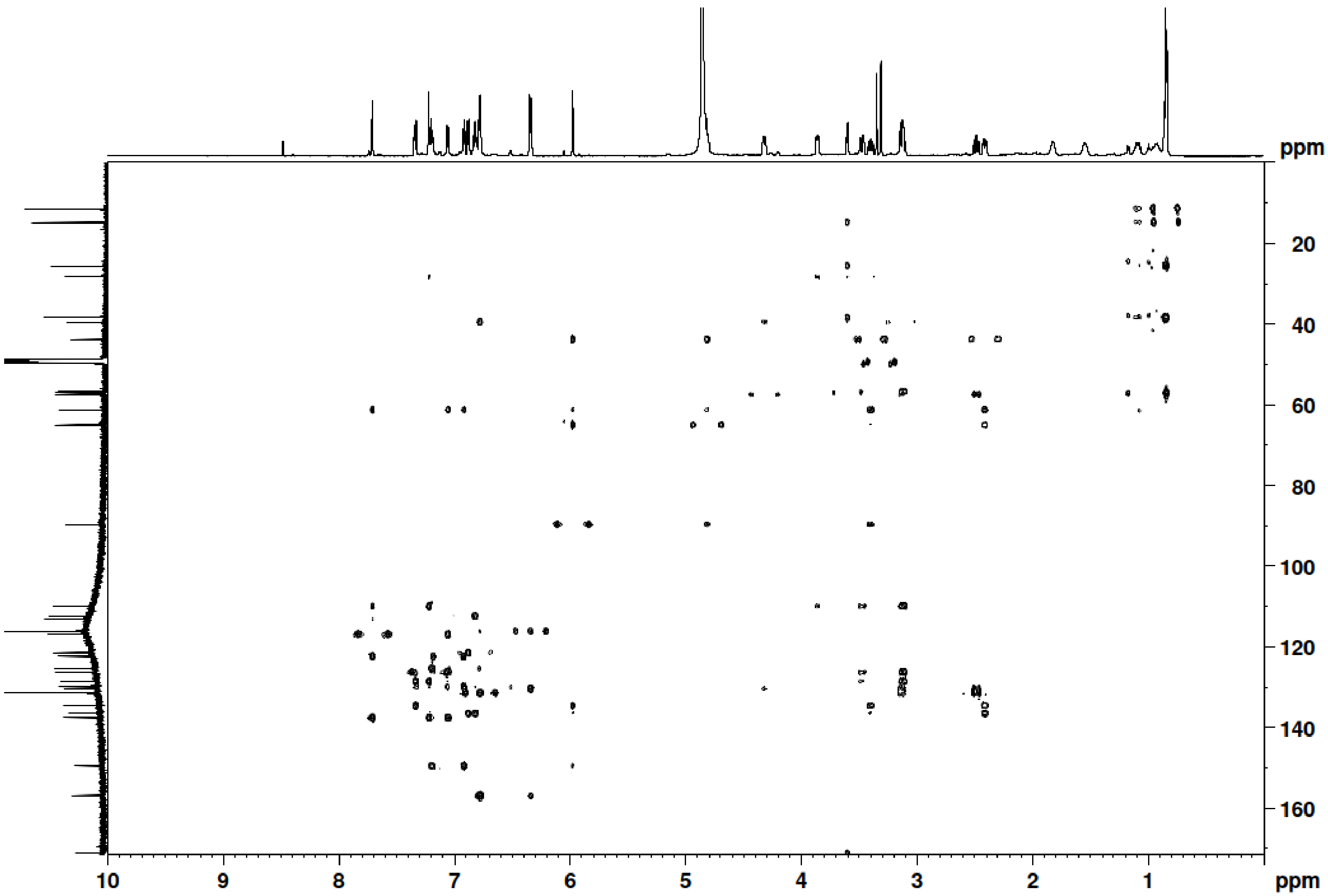

Figure S50. HMBC NMR spectrum (500 MHz) of **6** in MeOD.

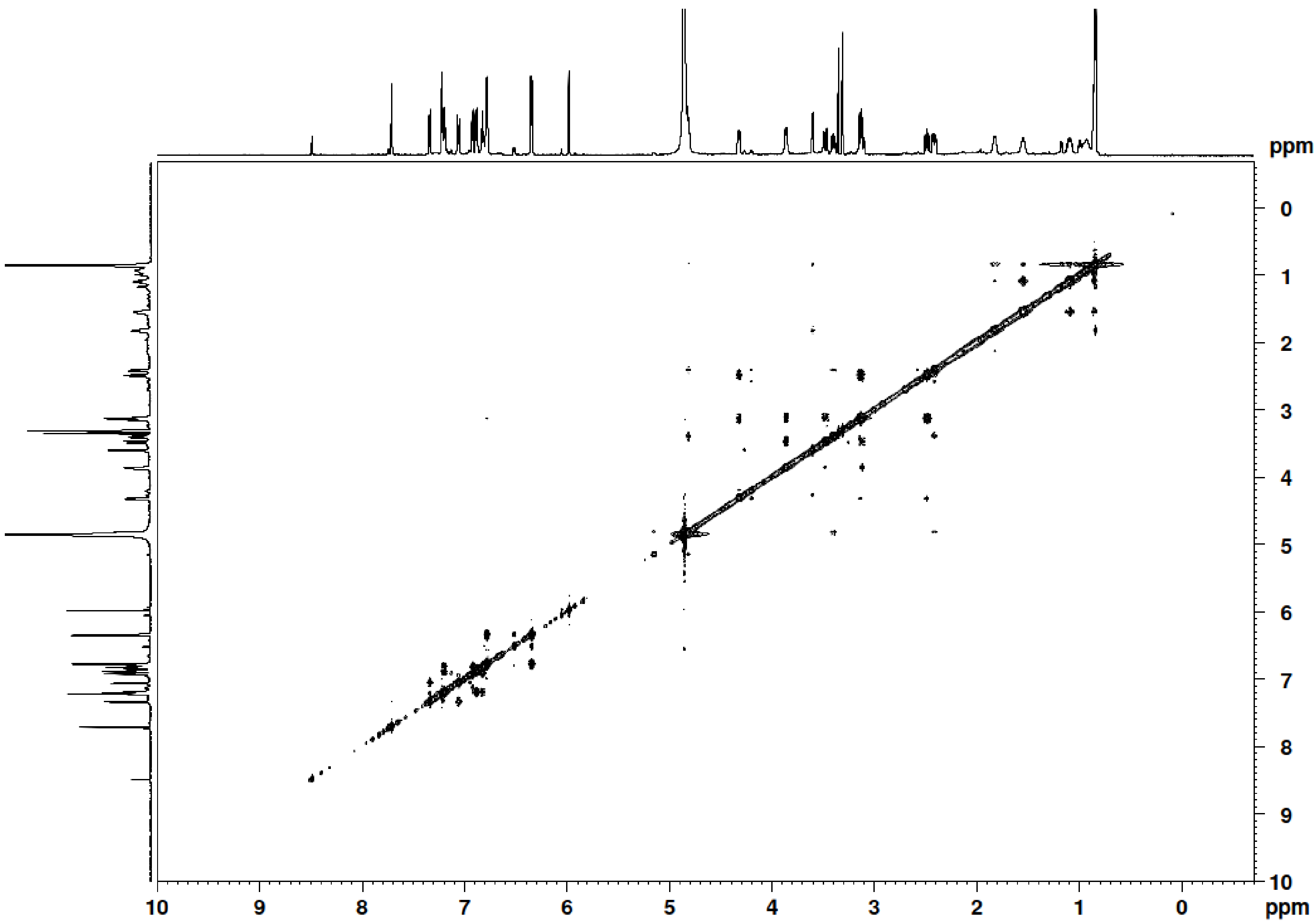

Figure S51. NOESY NMR spectrum (500 MHz) of **6** in MeOD.

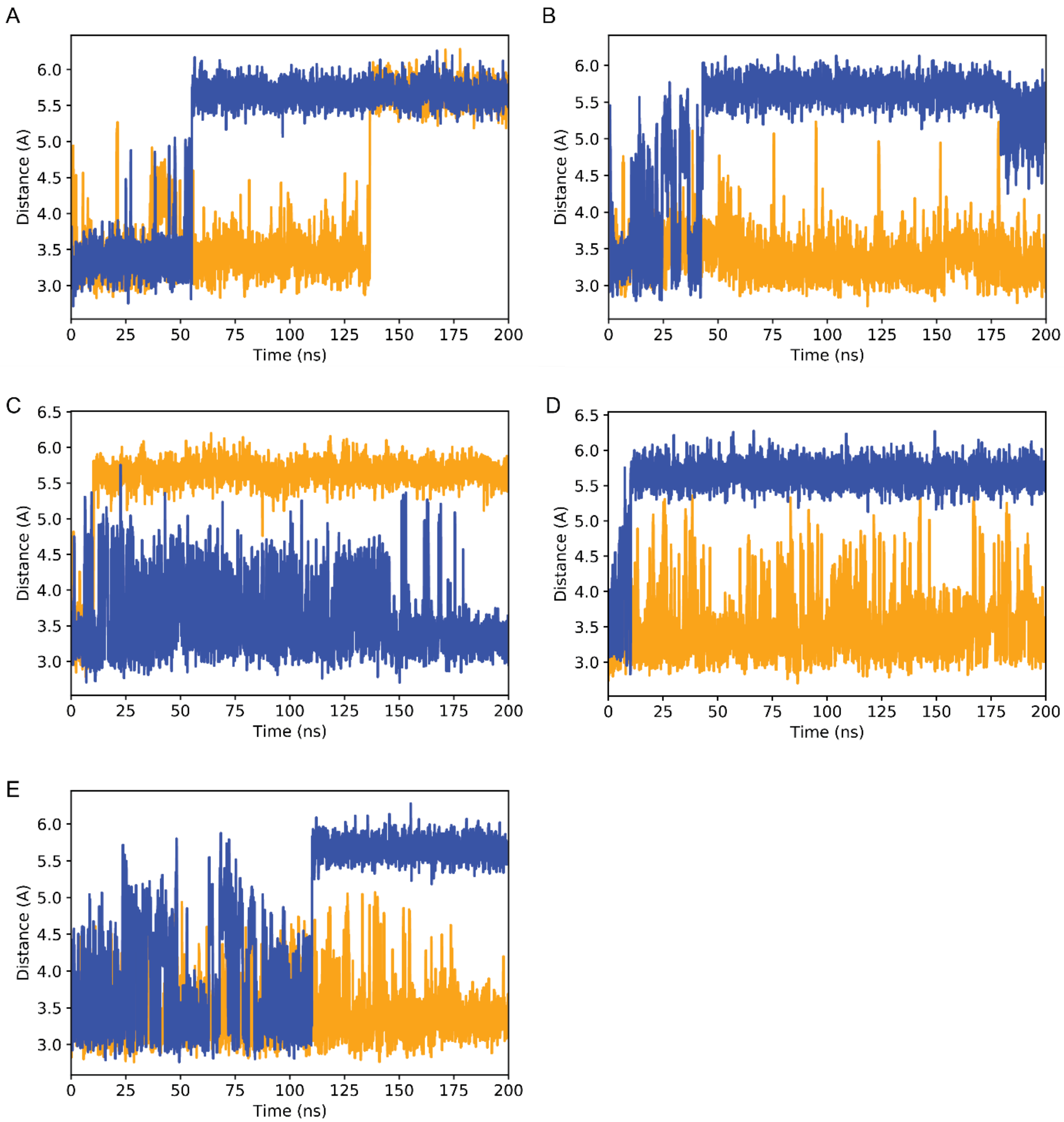

Figure S52. Molecular dynamics simulations of **6**. Each panel represents simulations from a different starting structure. Simulations in water are shown in blue and simulations in DMSO are shown in orange.

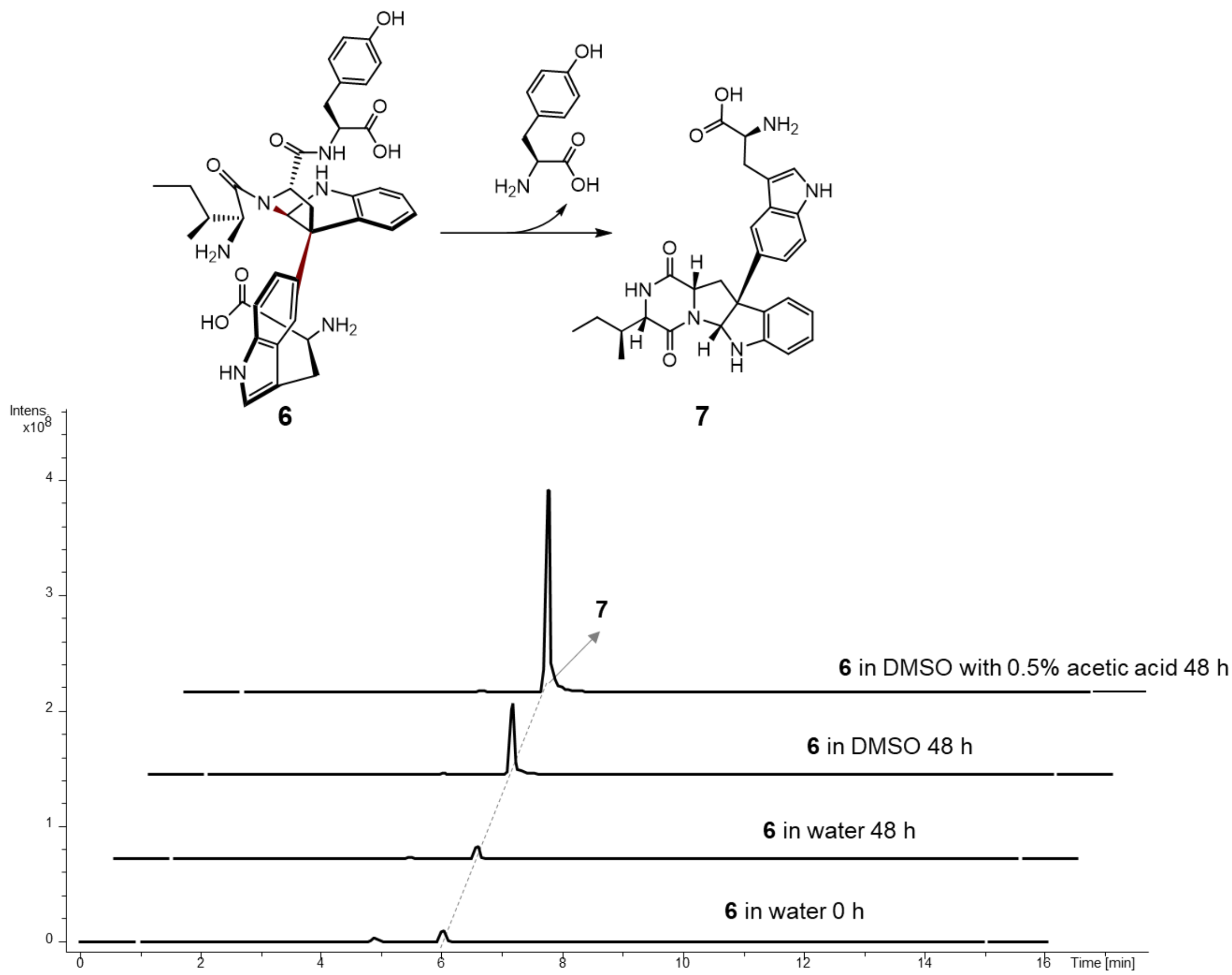

Figure S53. Spontaneous transformation of **6** into **7** in different conditions. Extracted ion chromatograms of **6** and **7** from samples of **6** dissolved in DMSO with or without acetic acid.

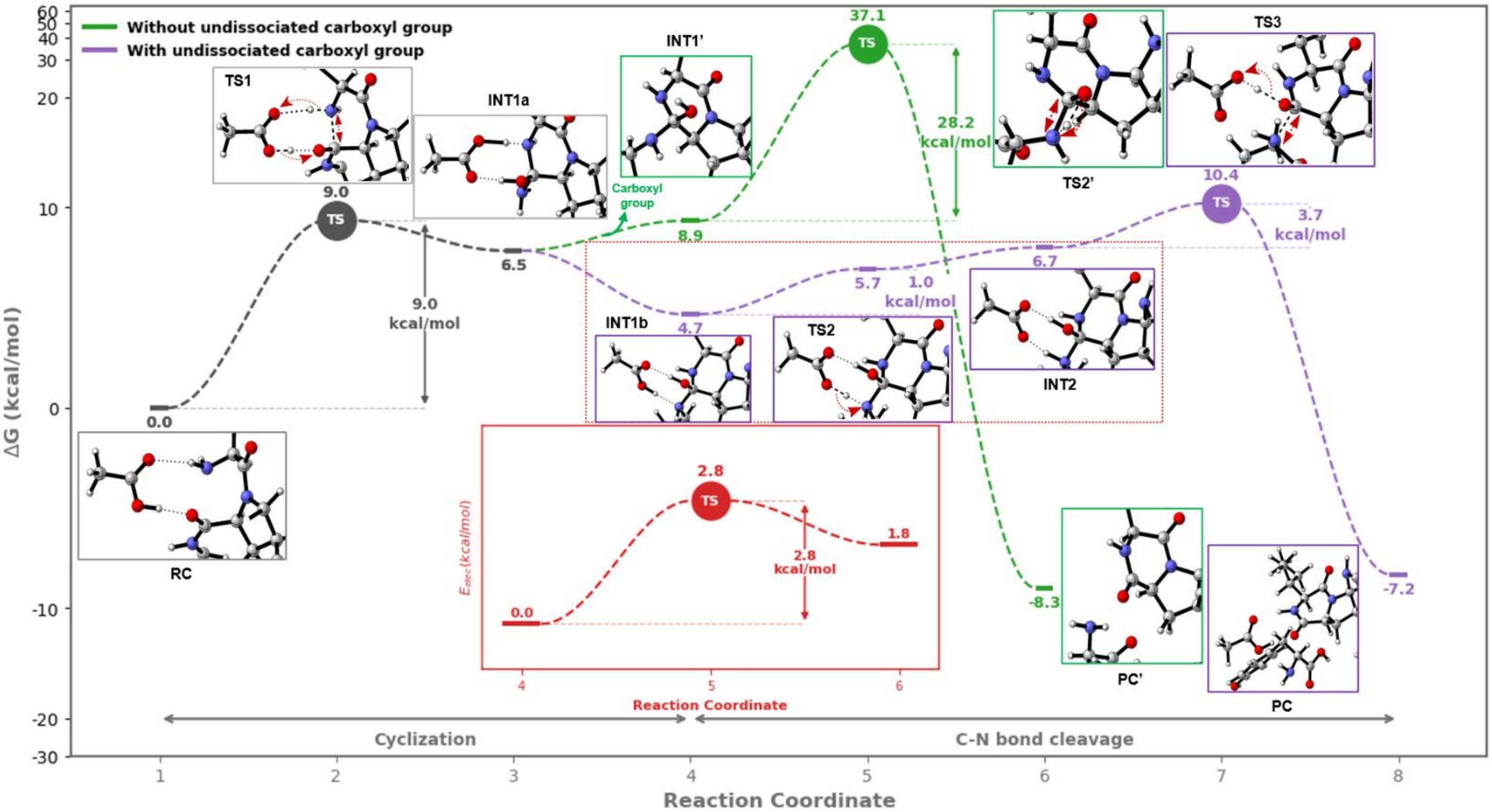

Figure S54. Mechanistic investigation of the proposed diketopiperazine formation. DFT-computed free-energy profile for the proposed diketopiperazine formation from the atropopeptide derivative. Two catalytic scenarios were investigated: with an undissociated carboxyl group (purple) and without an undissociated carboxyl group (green), together with the corresponding key intermediates and proposed reaction scheme. For a detailed description of the results of the DFT calculations see "**Detailed mechanistic insights of diketopiperazine formation"** on page 134.

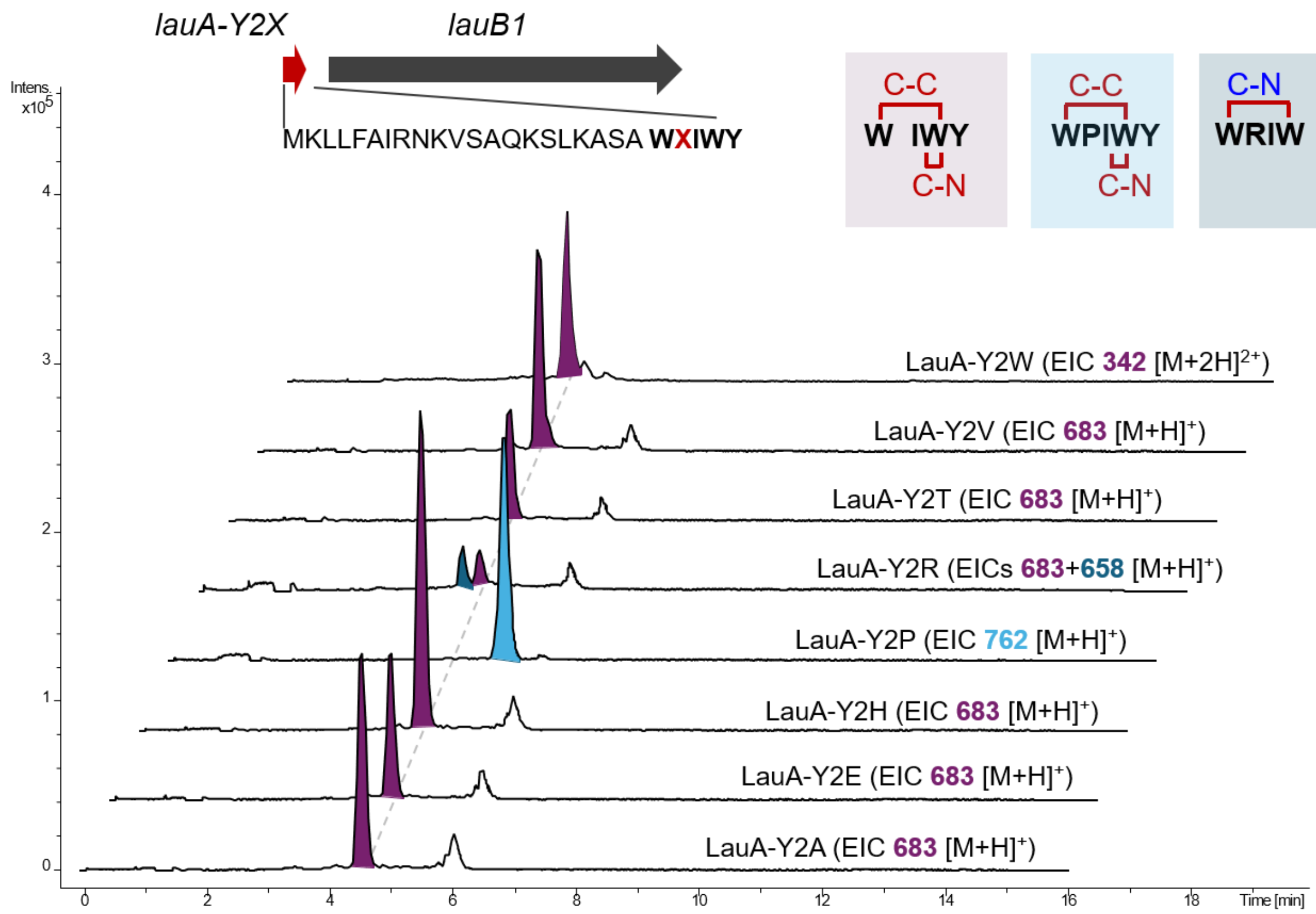

Figure S55. Coexpression of *lauA-Y2X* variants with *lauB1*. Extracted ion chromatogram of products generated from the coexpression of *lauA-Y2X* with *lauB1.*

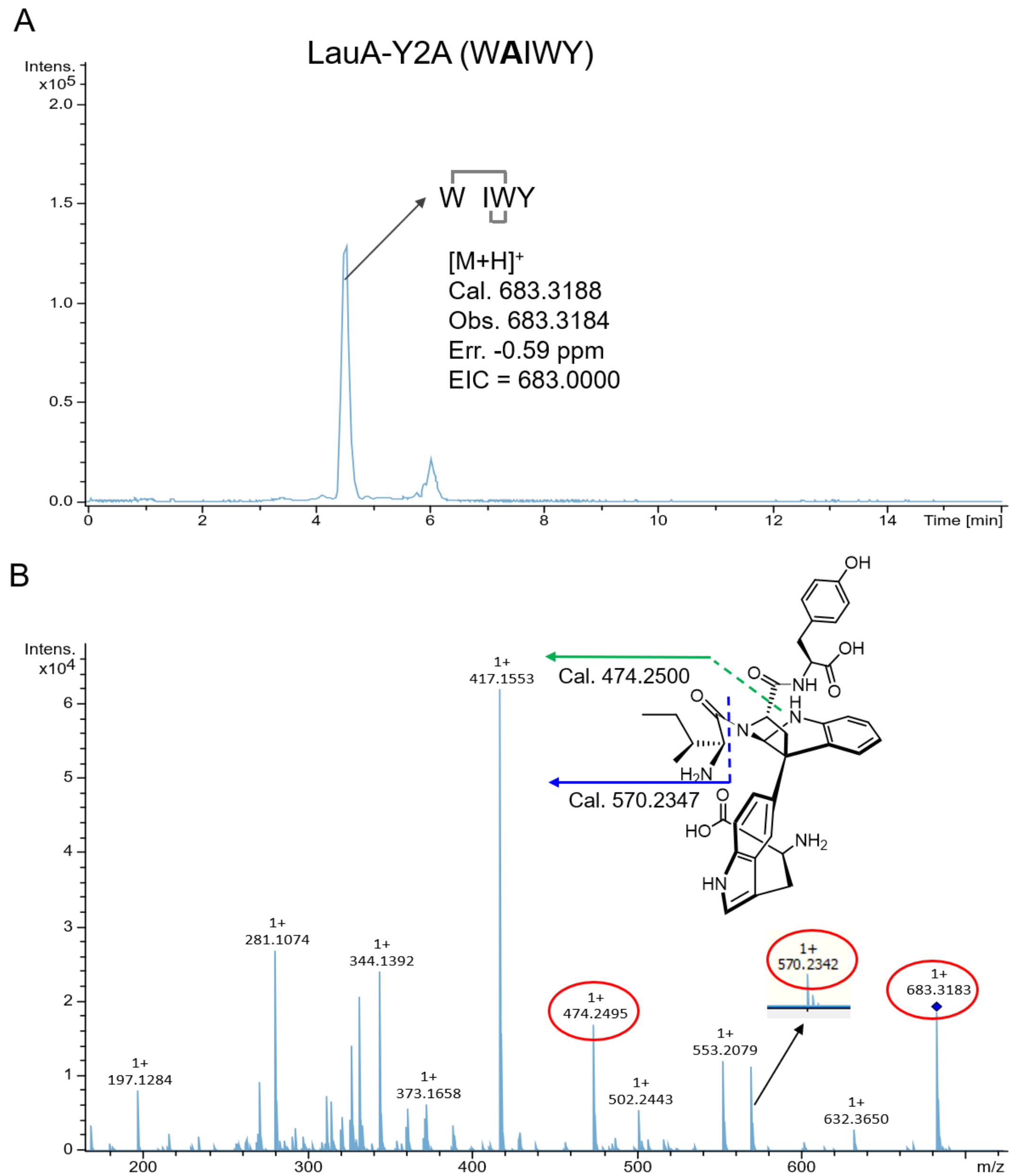

Figure S56. HRMS analysis of extracts of the recombinant *S. albus* strain harboring *lauA*-Y2A and *lauB1*. A) Extracted ion chromatogram of the product from the coexpression of *lauA*-Y2A with *lauB1*; B) MS/MS spectrum of the corresponding atropopeptide detected at *m*/*z* 683.3184 $[M+H]^+$ with key fragments indicating the loss of alanine at the second position of the core peptide highlighted.

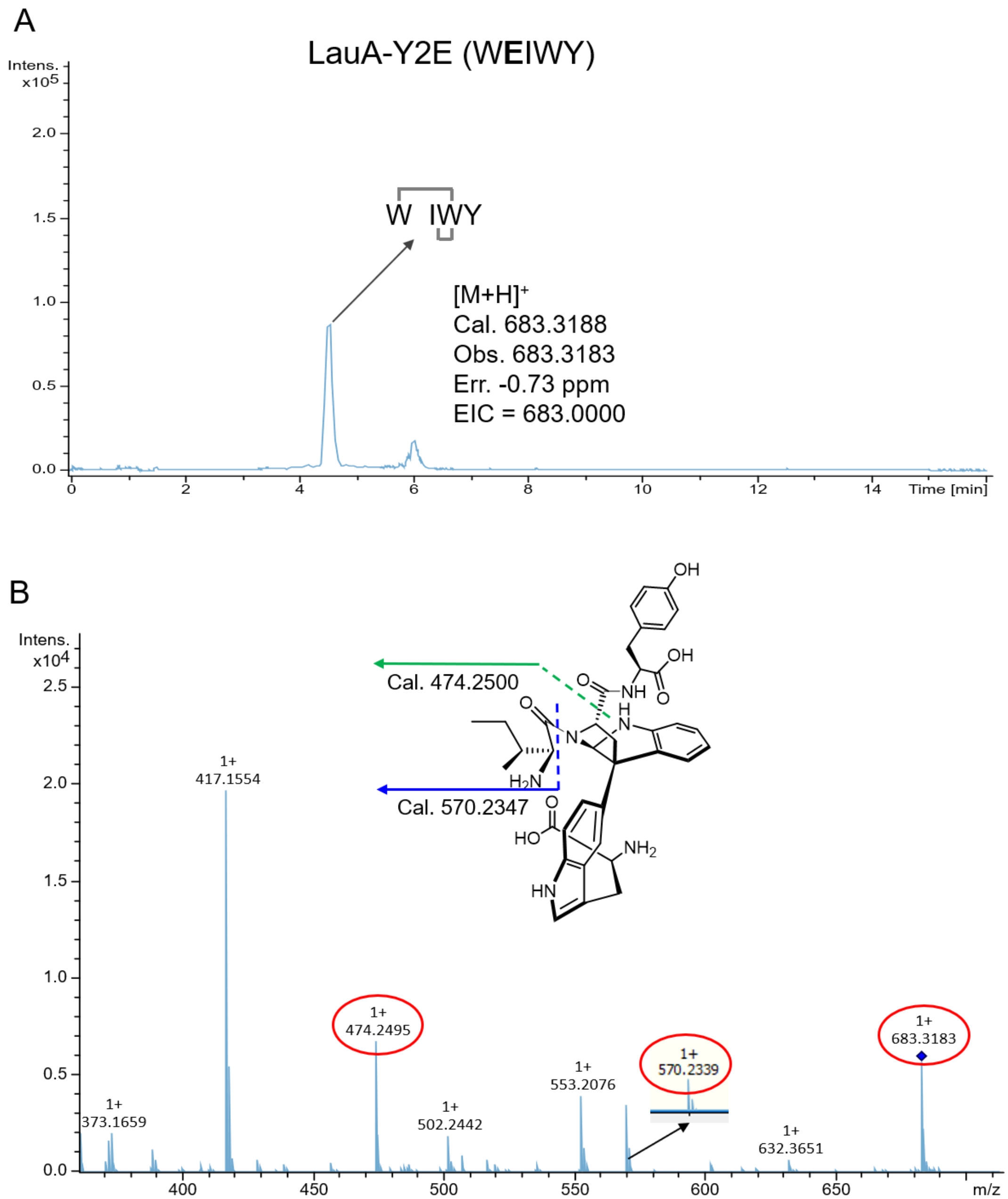

Figure S57. HRMS analysis of extracts of the recombinant *S. albus* strain harboring *lauA*-Y2E and *lauB1*. A) Extracted ion chromatogram of the product from the coexpression of *lauA*-Y2E with *lauB1*; B) MS/MS spectrum of the corresponding atropopeptide detected at *m*/*z* 683.3183 [M+H]+ with key fragments indicating the loss of glutamic acid at the second position of the core peptide highlighted.

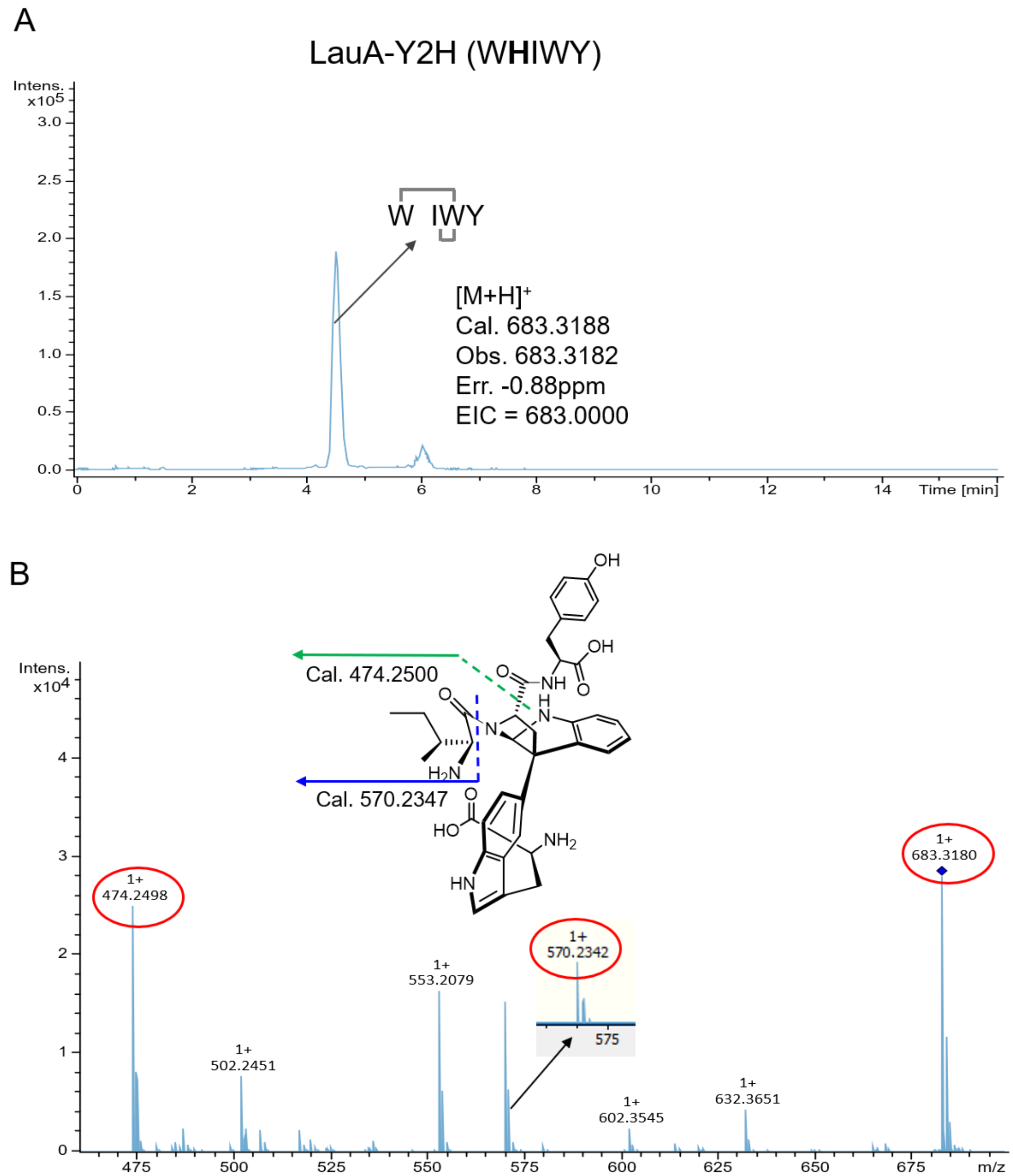

Figure S58. HRMS analysis of extracts of the recombinant *S. albus* strain harboring *lauA*-Y2H and *lauB1*. A) Extracted ion chromatogram of the product from the coexpression of *lauA*-Y2H with *lauB1*; B) MS/MS spectrum of the corresponding atropopeptide detected at *m*/*z* 683.3182 $[M+H]^+$ with key fragments indicating the loss of histidine at the second position of the core peptide highlighted.

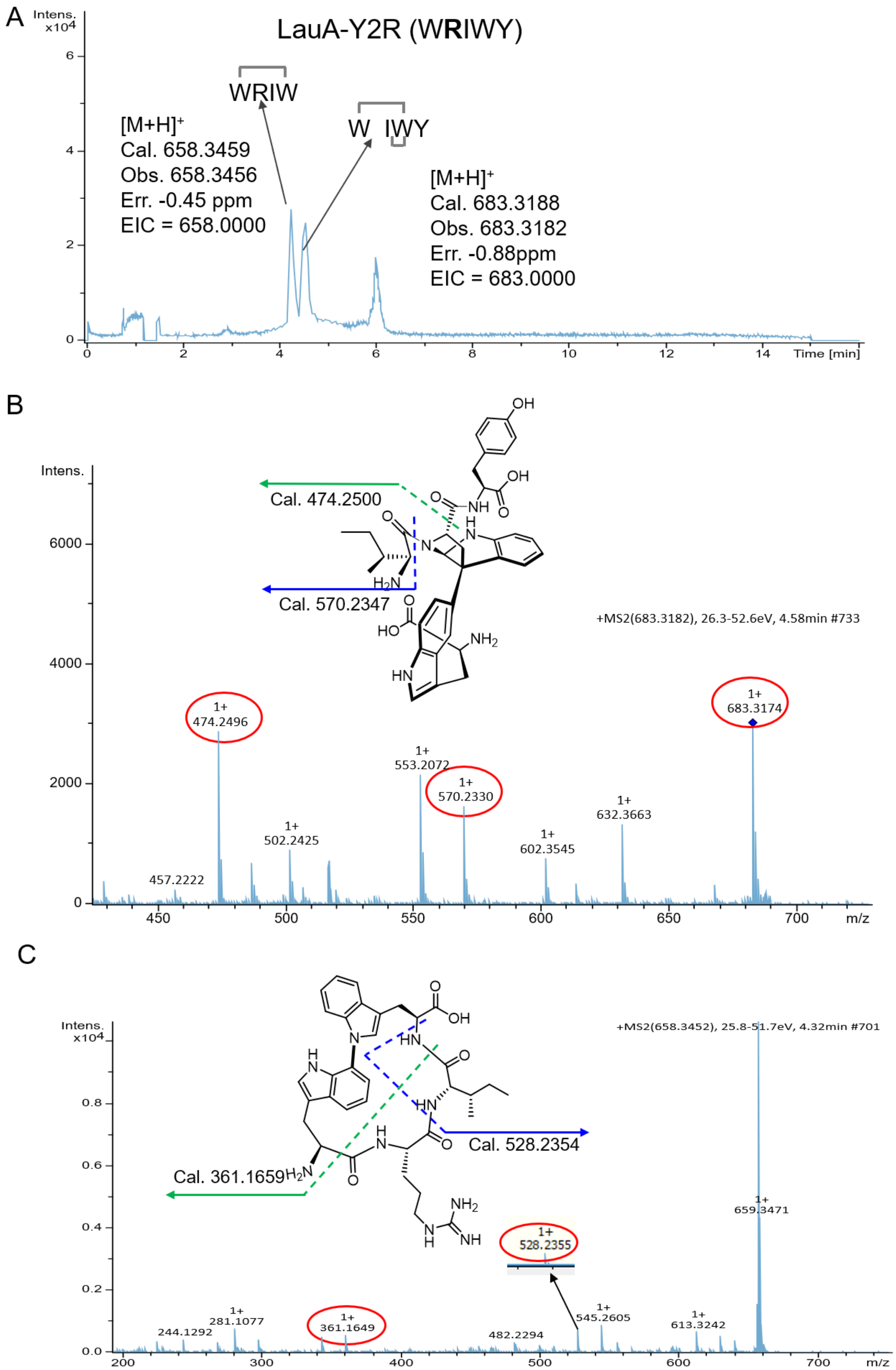

Figure S59. HRMS analysis of extracts of the recombinant *S. albus* strain harboring *lauA*-Y2R and *lauB1*. A) Extracted ion chromatogram of the product from the coexpression of *lauA*-Y2R with *lauB1*; B) MS/MS spectrum of the corresponding atropopeptide detected at *m*/*z* 683.3182 [M+H]$^+$ with key fragments indicating the loss of arginine at the second position of the core peptide highlighted; C) MS/MS spectrum of the corresponding atropopeptide detected at *m*/*z* 658.3456 [M+H]$^+$.

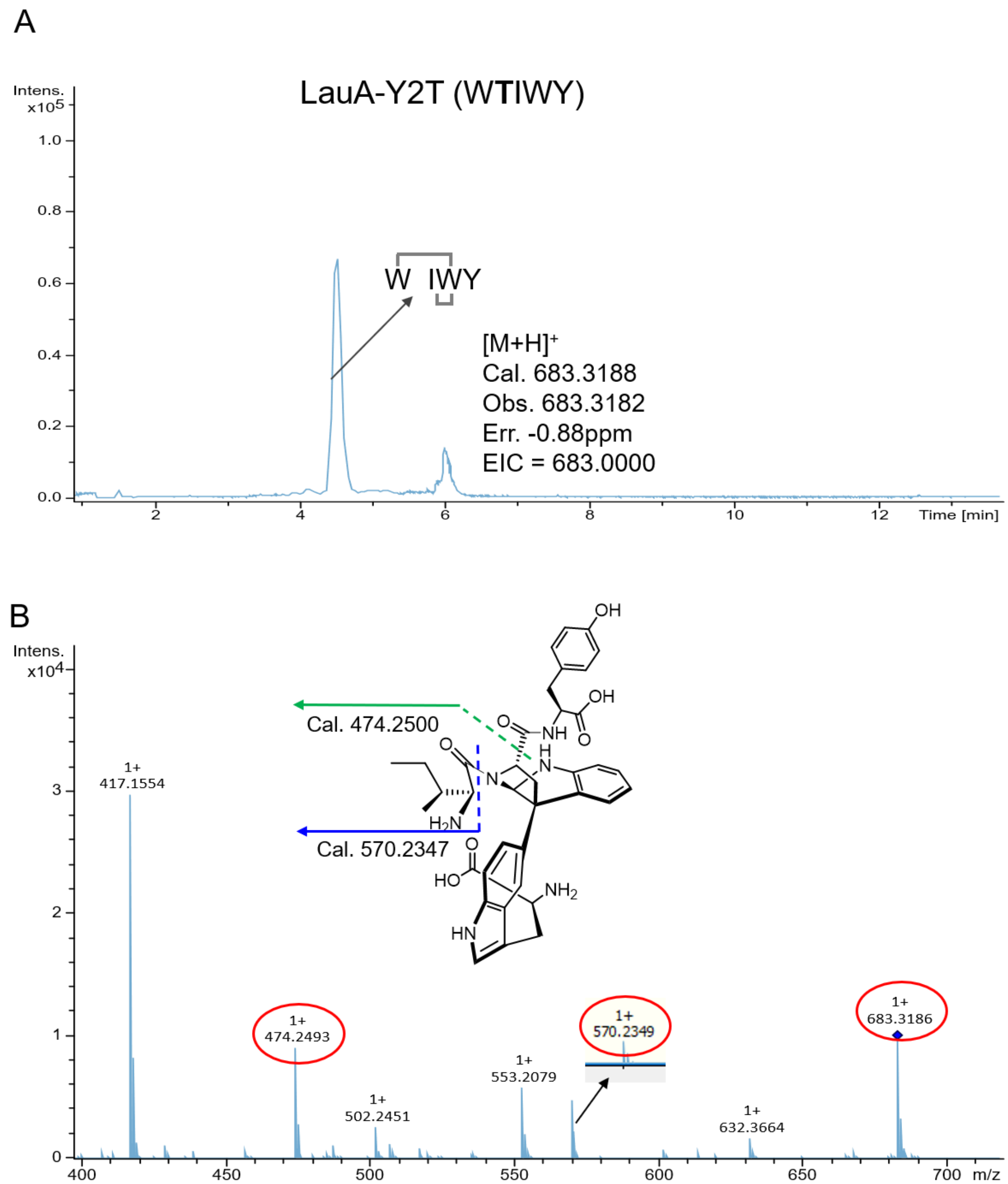

Figure S60. HRMS analysis of extracts of the recombinant *S. albus* strain harboring *lauA*-Y2T and *lauB1*. A) Extracted ion chromatogram of the product from the coexpression of *lauA*-Y2T with *lauB1*; B) MS/MS spectrum of the corresponding atropopeptide detected at *m*/*z* 683.3182 [M+H]+ with key fragments indicating the loss of threonine at the second position of the core peptide highlighted.

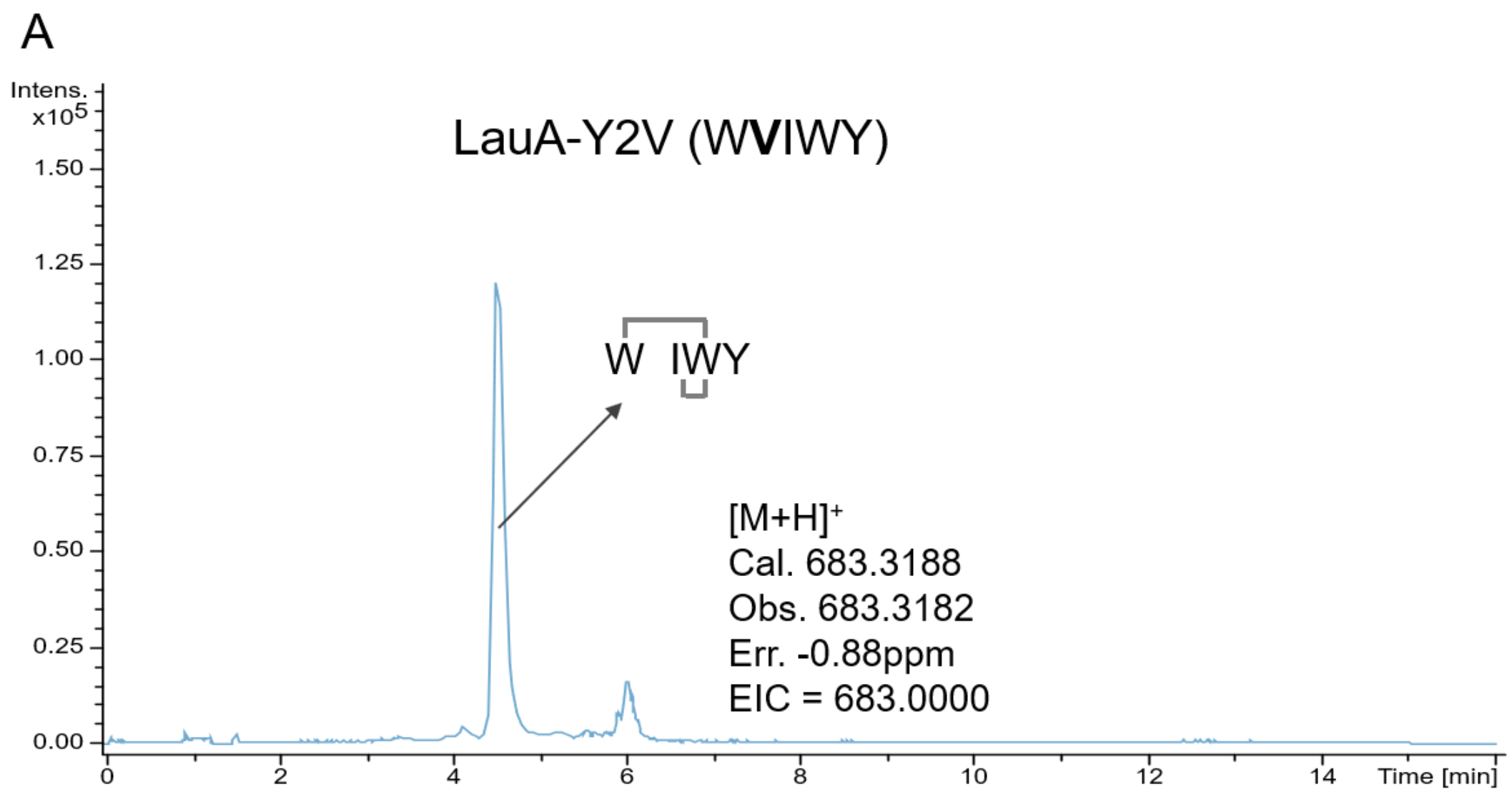

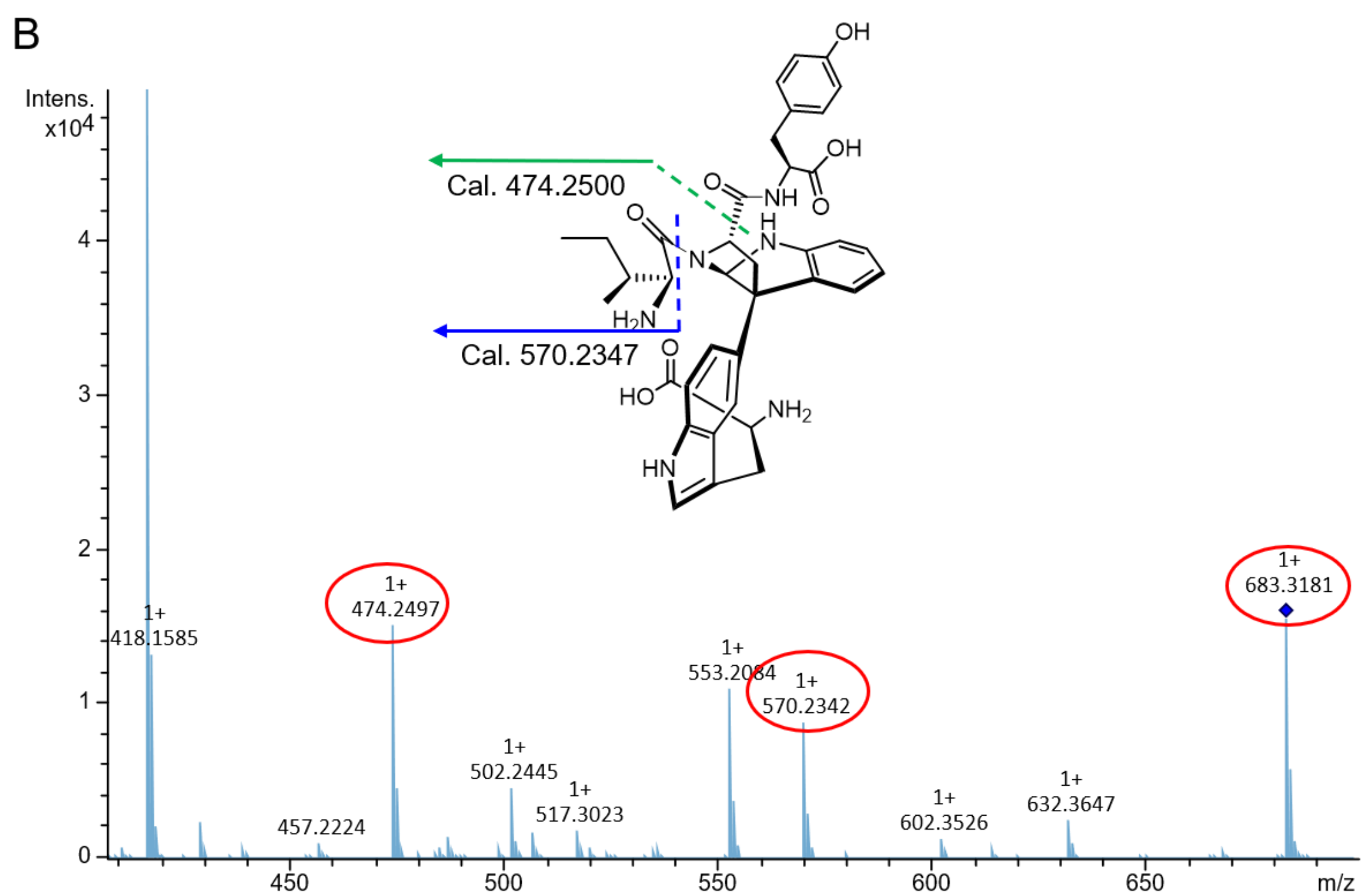

Figure S61. HRMS analysis of extracts of the recombinant *S. albus* strain harboring *lauA*-Y2V and *lauB1*. A) Extracted ion chromatogram of the product from the coexpression of *lauA*-Y2V with *lauB1*; B) MS/MS spectrum of the corresponding atropopeptide detected at *m/z* 683.3182 $[M+H]^+$ with key fragments indicating the loss of valine at the second position of the core peptide highlighted.

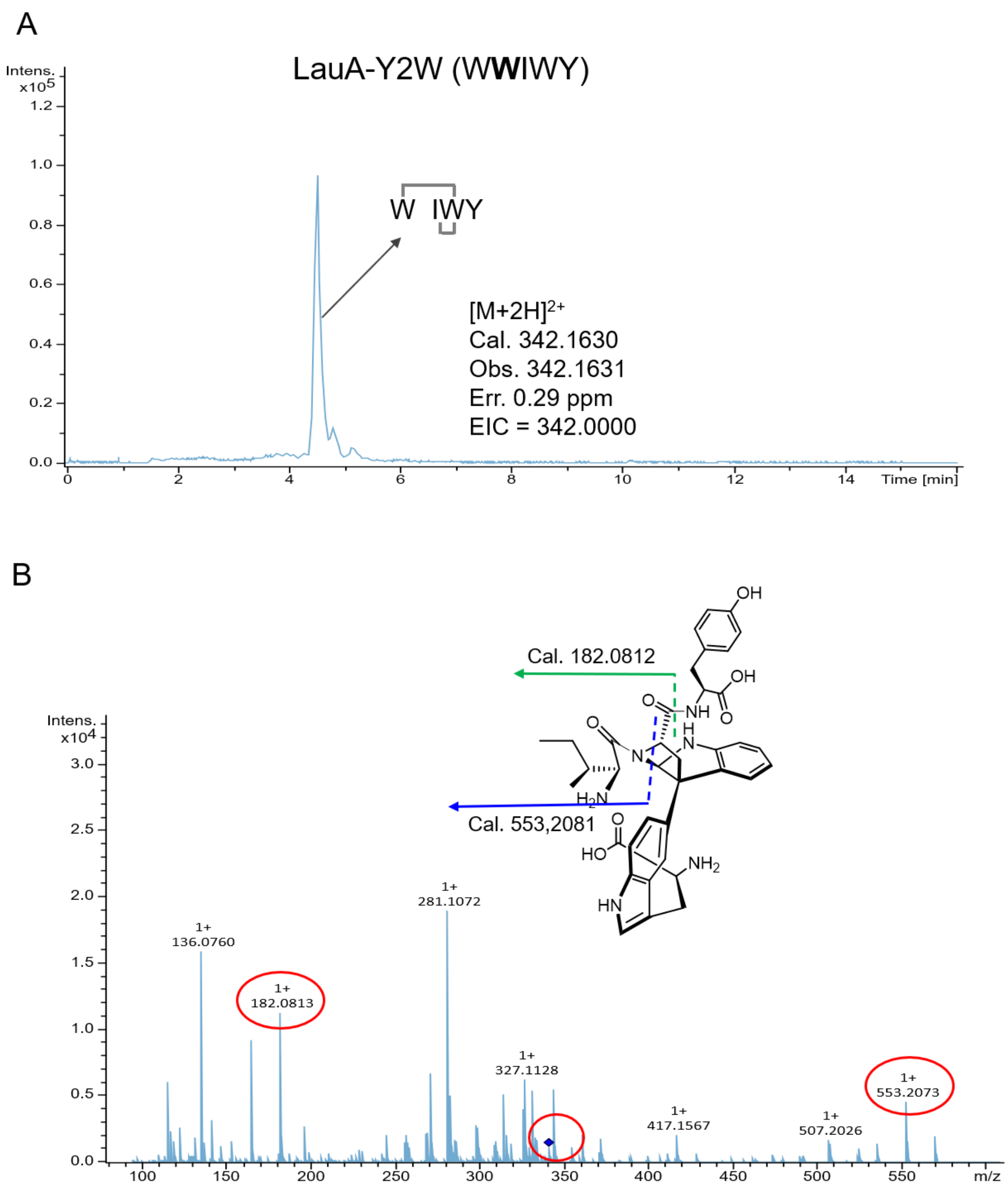

Figure S62. HRMS analysis of extracts of the recombinant *S. albus* strain harboring *lauA*-Y2W and *lauB1*. A) Extracted ion chromatogram of the product from the coexpression of *lauA*-Y2W with *lauB1*; B) MS/MS spectrum of the corresponding atropopeptide detected at *m*/*z* 342.1631 $[M+2H]^{2+}$ with key fragments indicating the loss of tryptophan at the second position of the core peptide highlighted.

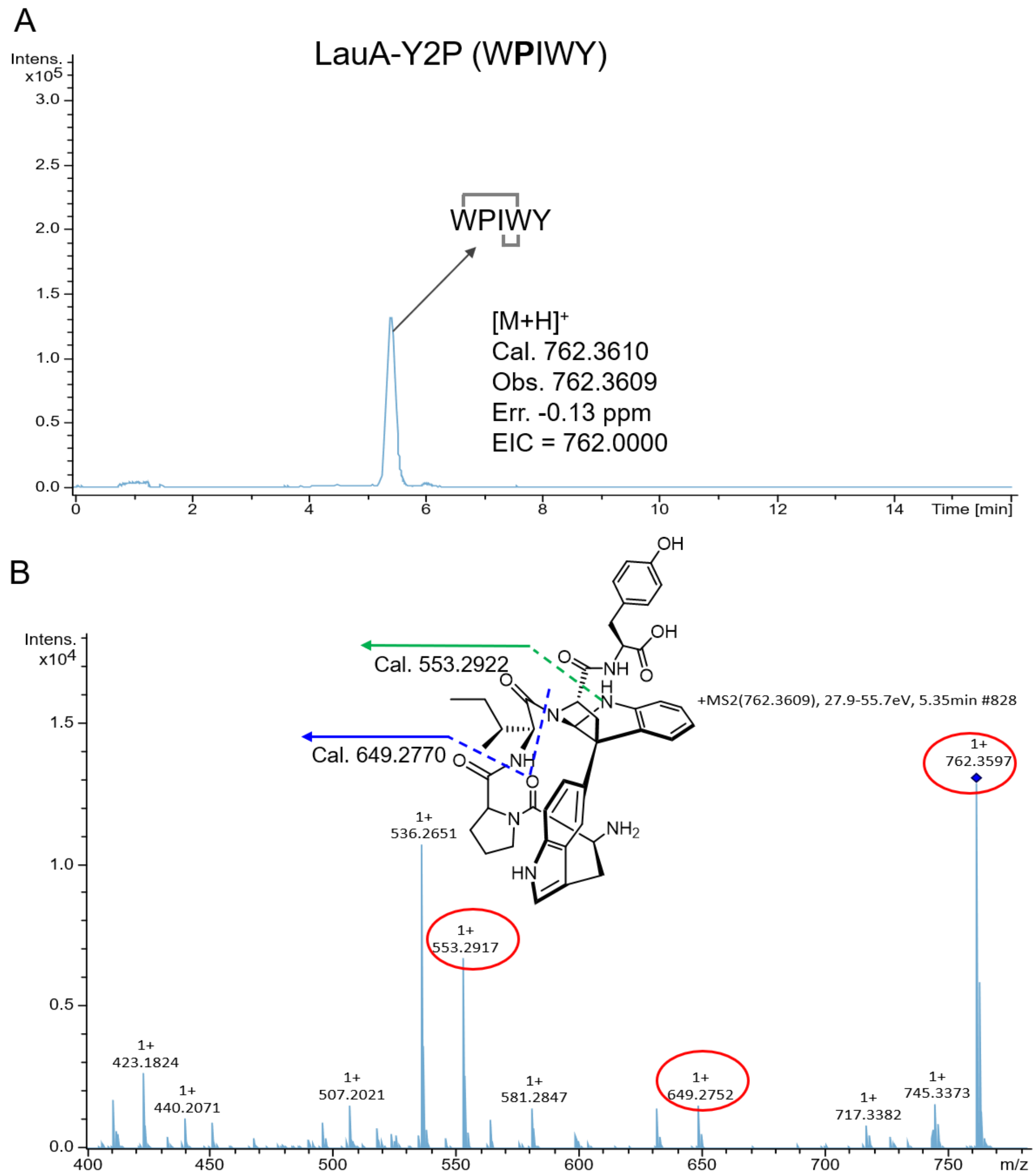

Figure S63. HRMS analysis of extracts of the recombinant *S. albus* strain harboring *lauA*-Y2P and *lauB1*. A) Extracted ion chromatogram of the product from the coexpression of *lauA*-Y2P with *lauB1*; B) MS/MS spectrum of the corresponding atropopeptide detected at *m*/*z* 762.3609 [M+H]$^+$ with key fragments indicating the presence of the second proline at the second position of the core peptide highlighted.

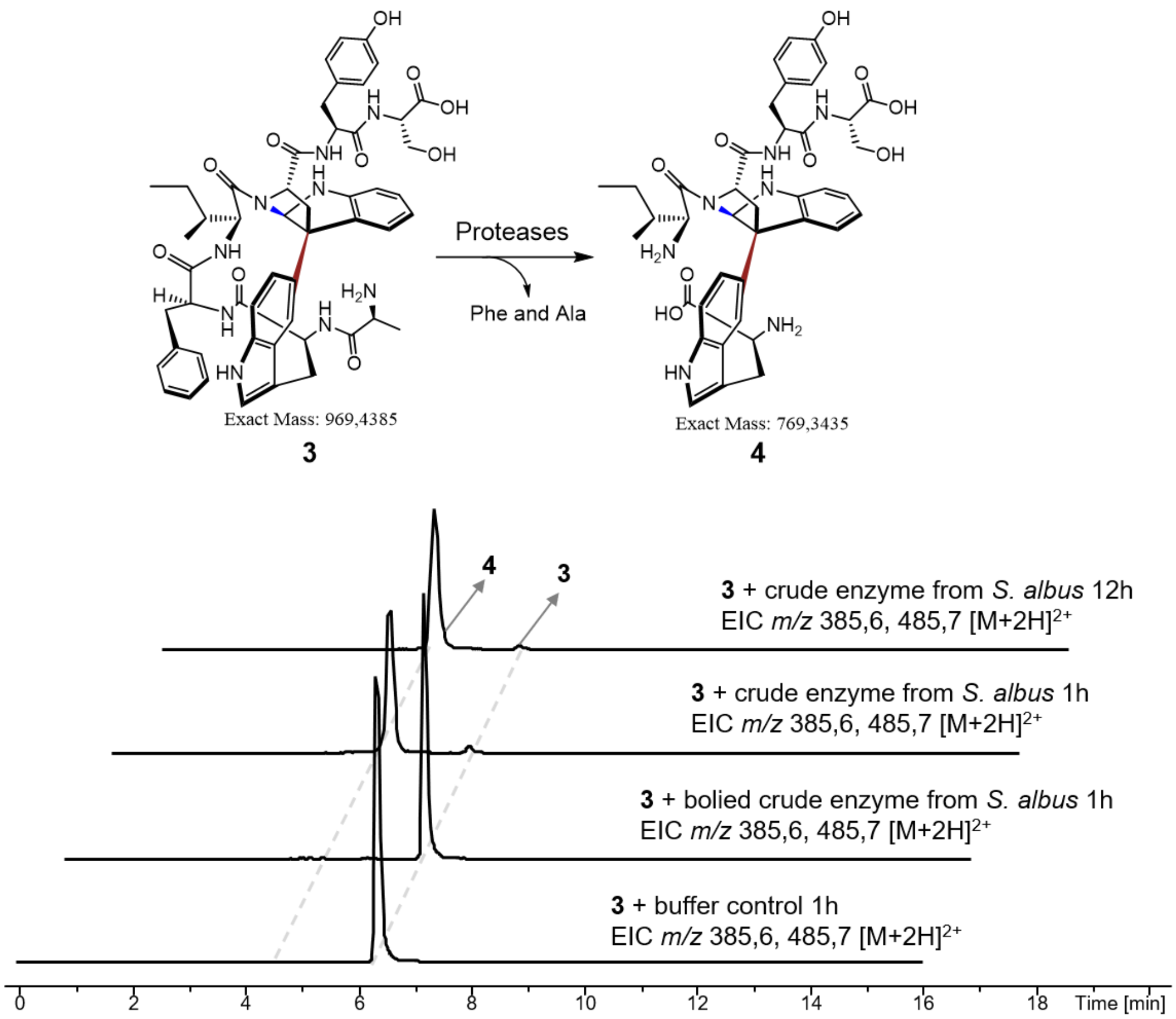

Figure S64. Bioconversion assays of **3** using crude enzyme extracts from *S. albus*. The crude enzyme extracts converted compound **3** into **4,** whereas no conversion was observed with boiled crude enzyme extracts or the buffer control. Each 50 µL reaction mixture contained 0.05 mM **3** and either buffer (50 mM Tris-HCl, 50 mM NaCl, pH 7.5) or crude enzymes prepared in the same buffer or the boiled crude enzymes. The reaction was performed at 30 °C for 1 h or 12 h.

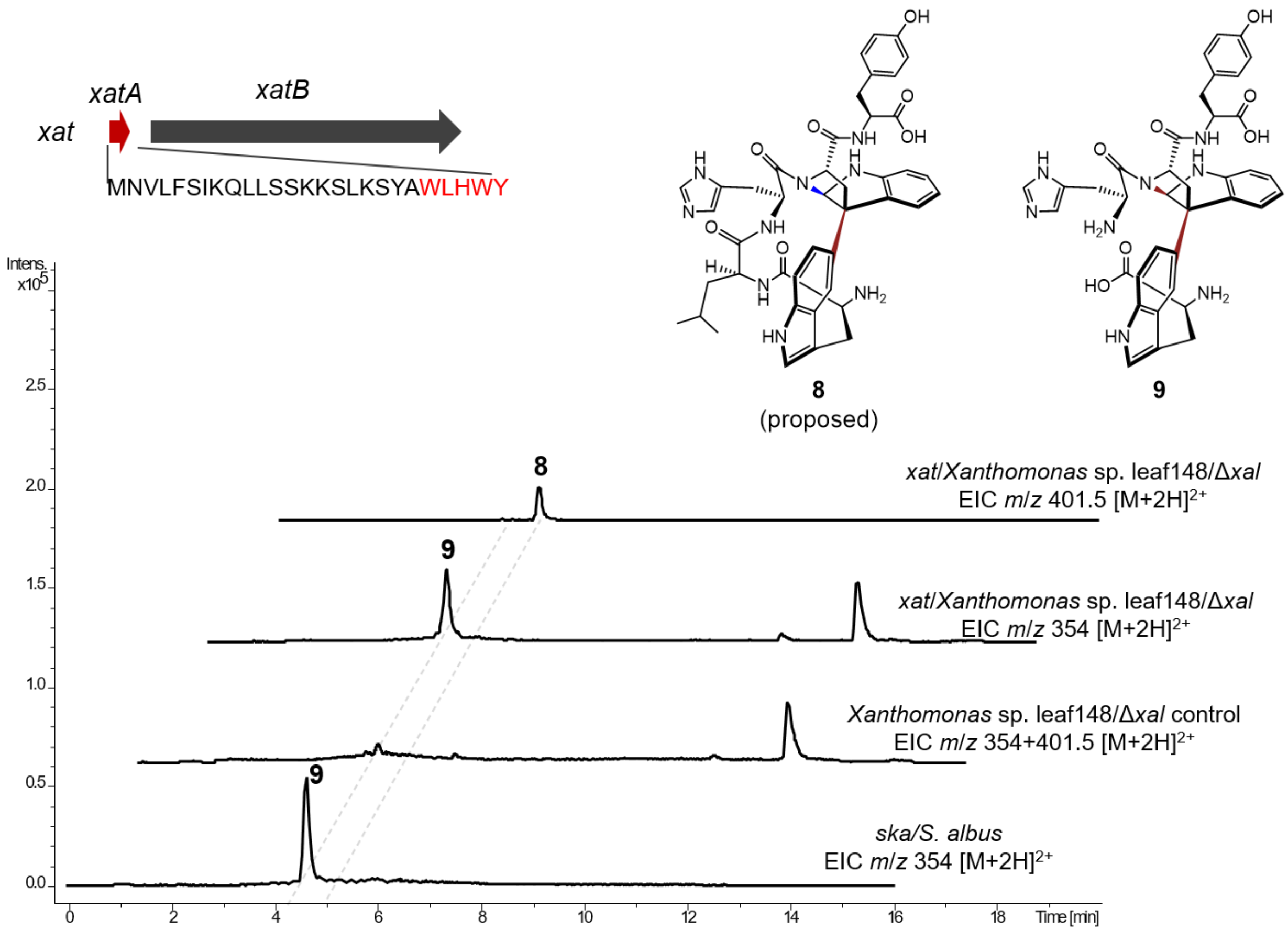

Figure S65. Heterologous expression of the *xat* BGC. Extracted ion chromatogram of two products from extracts of the control *Xanthomonas* sp. Leaf148/*Δxal* and *Xanthomonas* sp. Leaf148/*Δxal* harboring the *xat* BGC. Extract ion chromatogram of **9** from *S. albus* harboring pUWL201-OriT-ska as authentic standard.

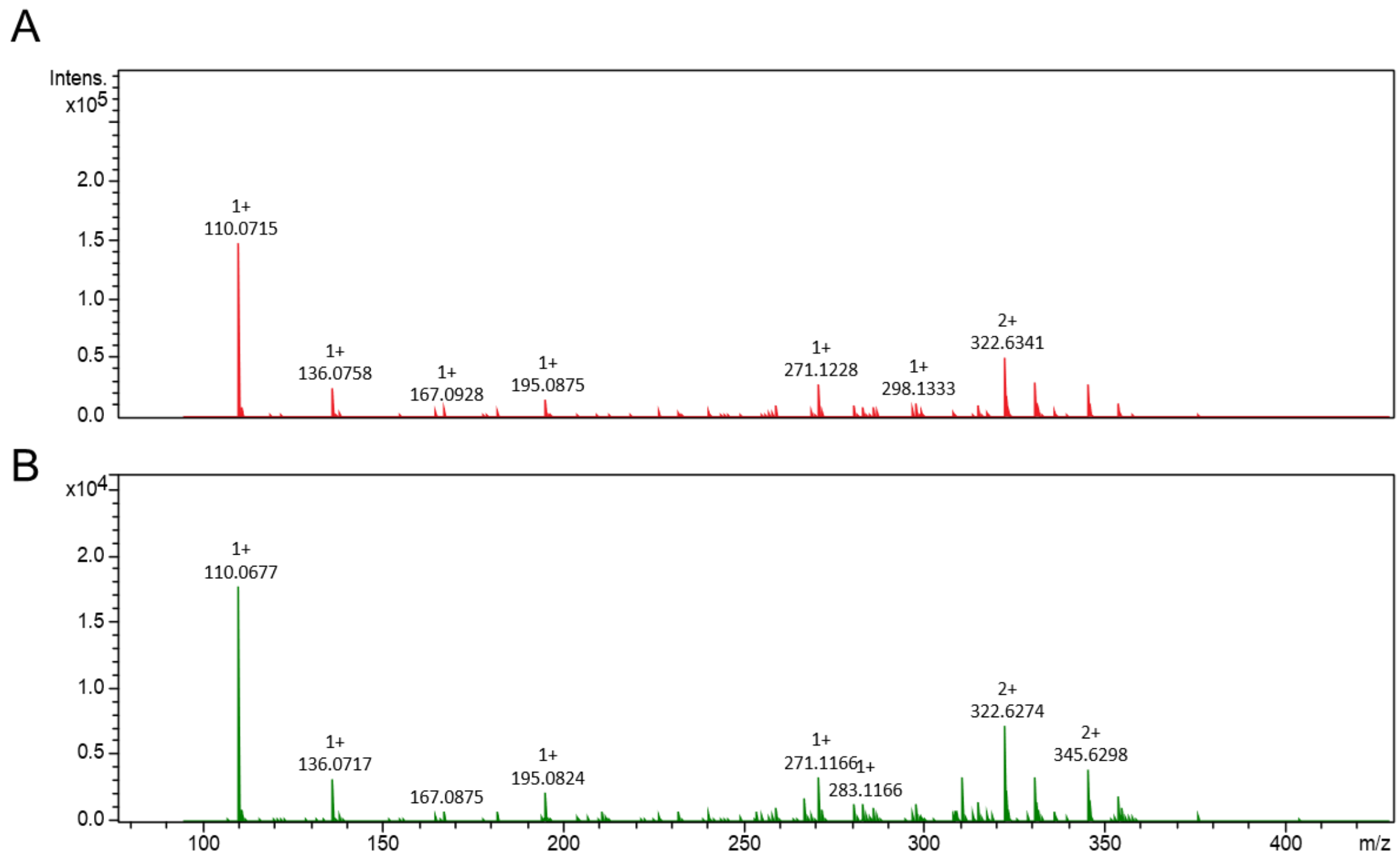

Figure S66. MS/MS fragmentation comparison of **9** and the major product from the *xat* BGC. A) MS/MS fragments of compound **9** from *S. albus* harboring pUWL201-OriT-ska; B) MS/MS fragments of the major product from *Xanthomonas* sp. Leaf148/*Δxal* harboring pME6012-xat.

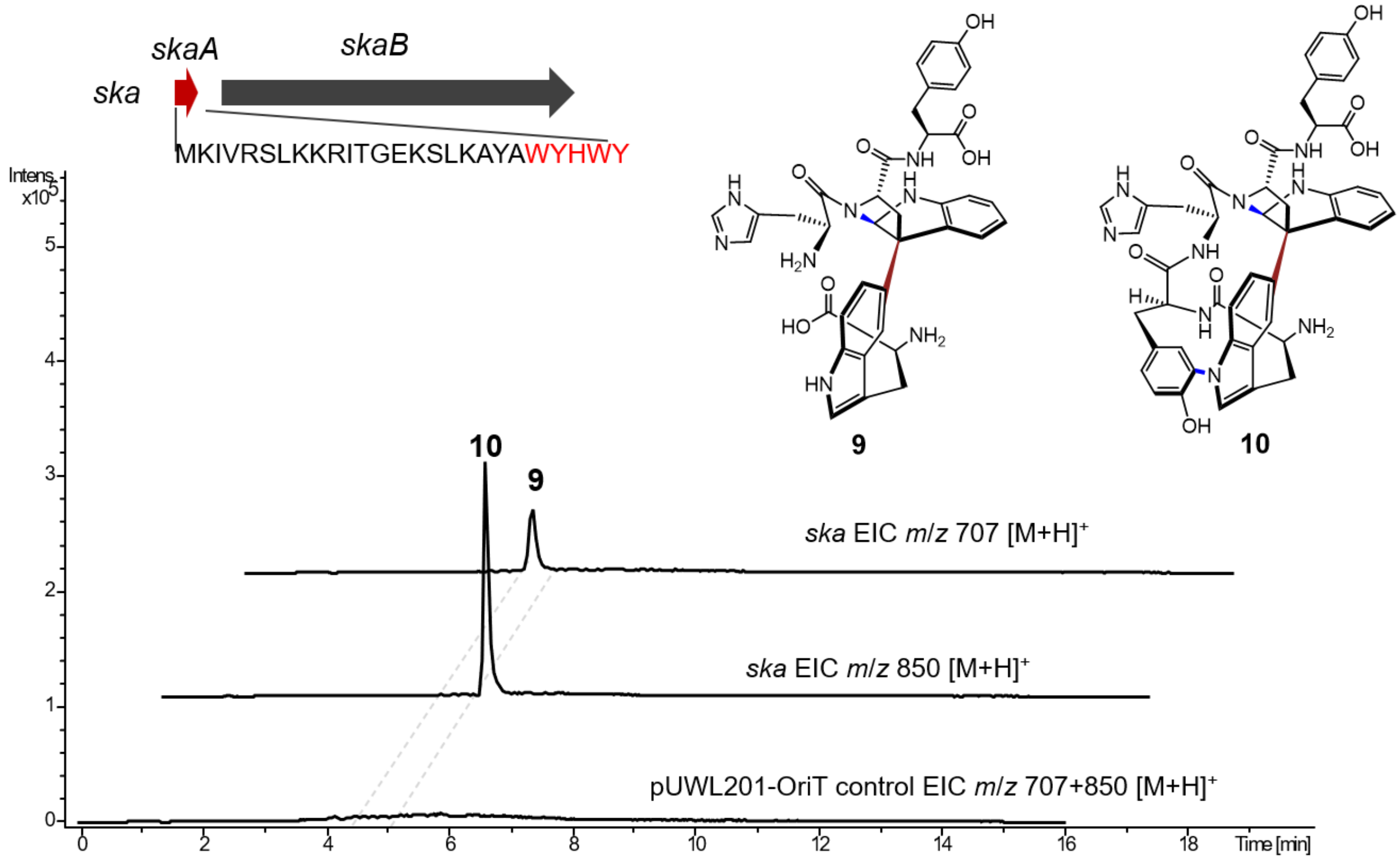

Figure S67. Heterologous expression of *ska* BGC. Extracted ion chromatogram of compounds **9** and **10** from extracts of *S. albus* harboring pUWL201-OriT-ska and *S. albus* harboring pUWL201-Ori as a negative control. Compound **9** is a new compound that was fully characterized (Figure S60-S72) and used in this study as an authentic standard for the major product generated from the *xat* BGC. Compound **10** is a known compound[1] (see the Structure Elucidation section).

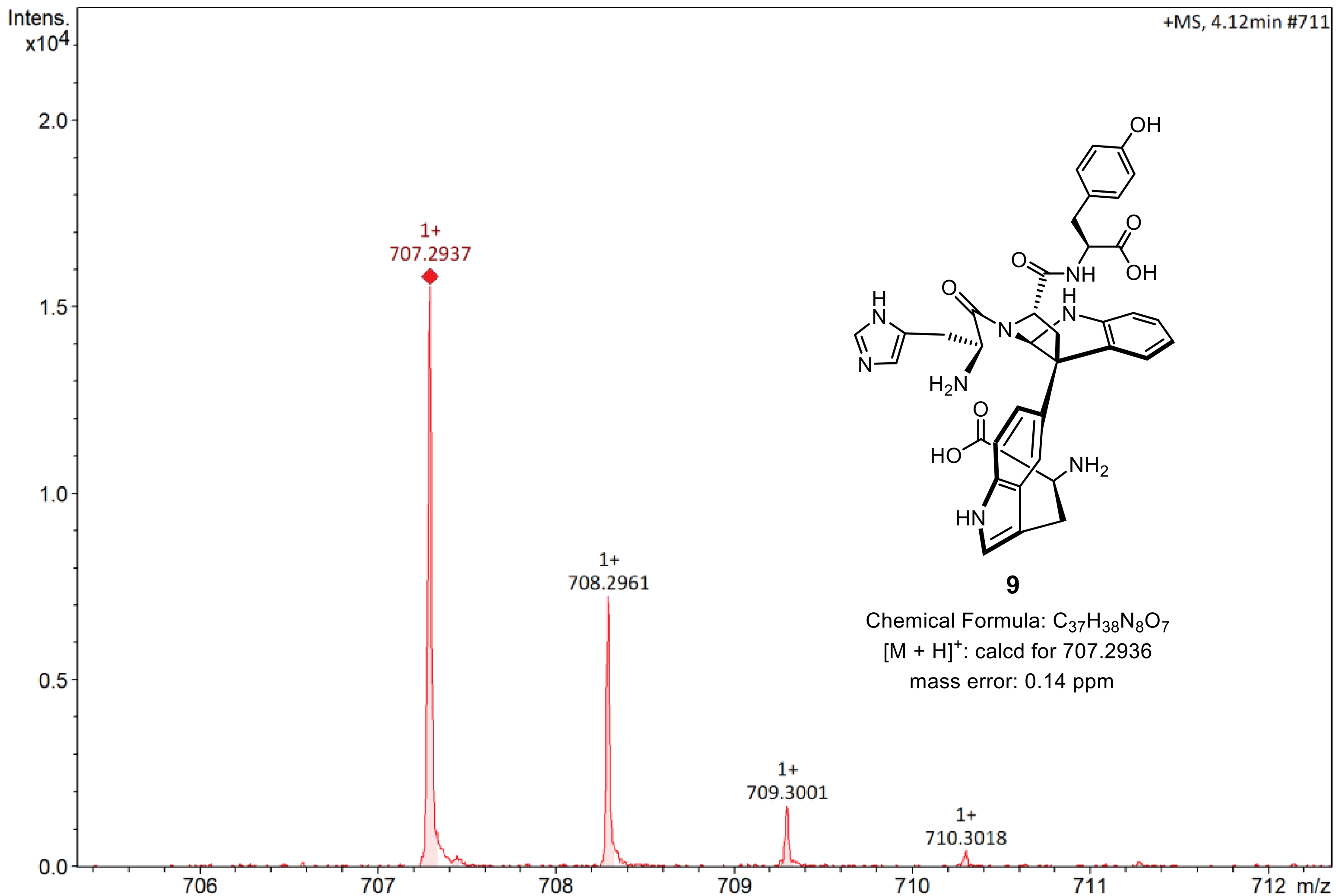

Figure S68. HPLC-ESI-QTOF-HRMS analysis of **9**.

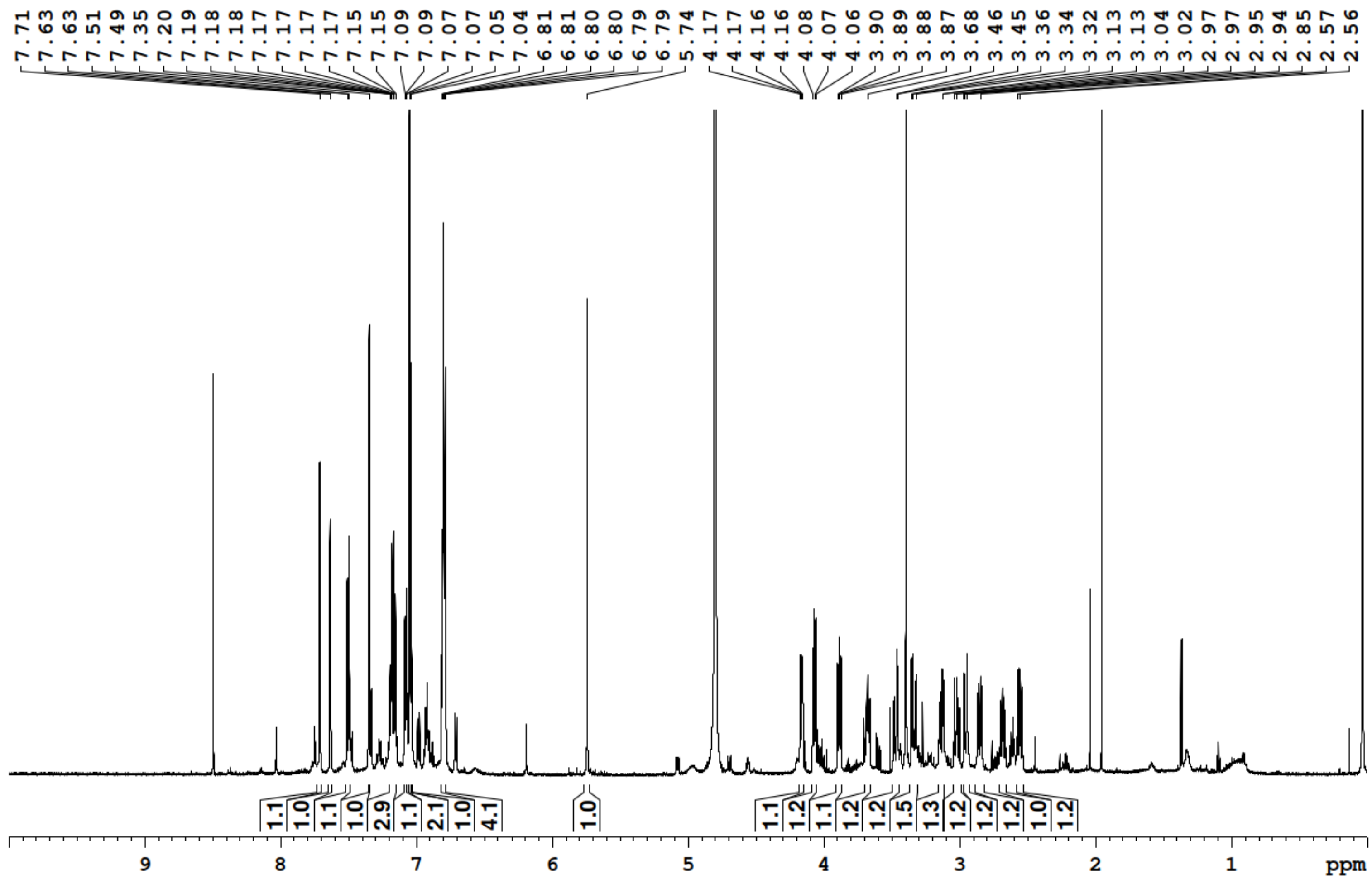

Figure S69. ^{1}H NMR spectrum (600 MHz) of **9** in D_2O.

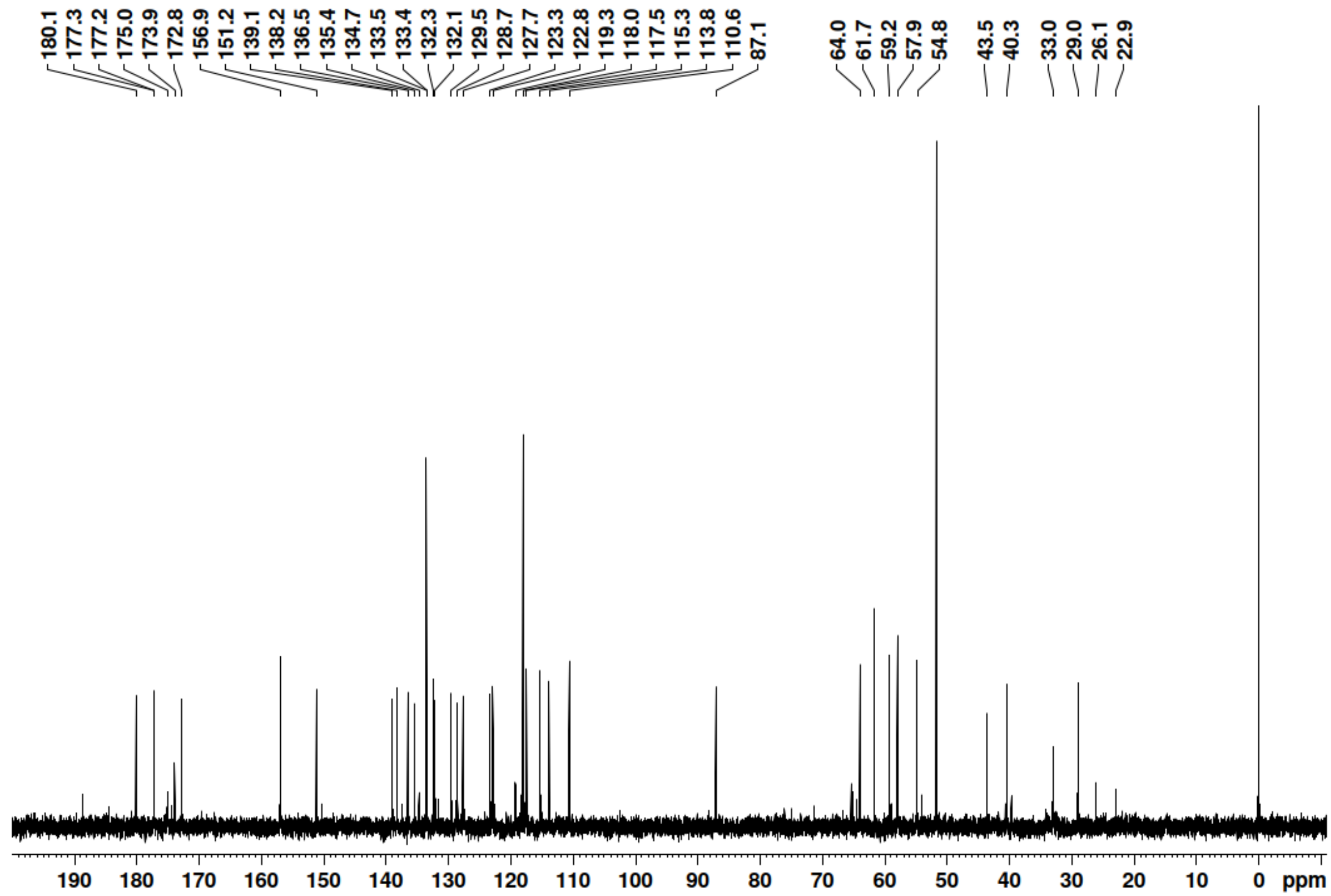

Figure S70. ^{13}C NMR spectrum (125 MHz) of **9** in D_2O.

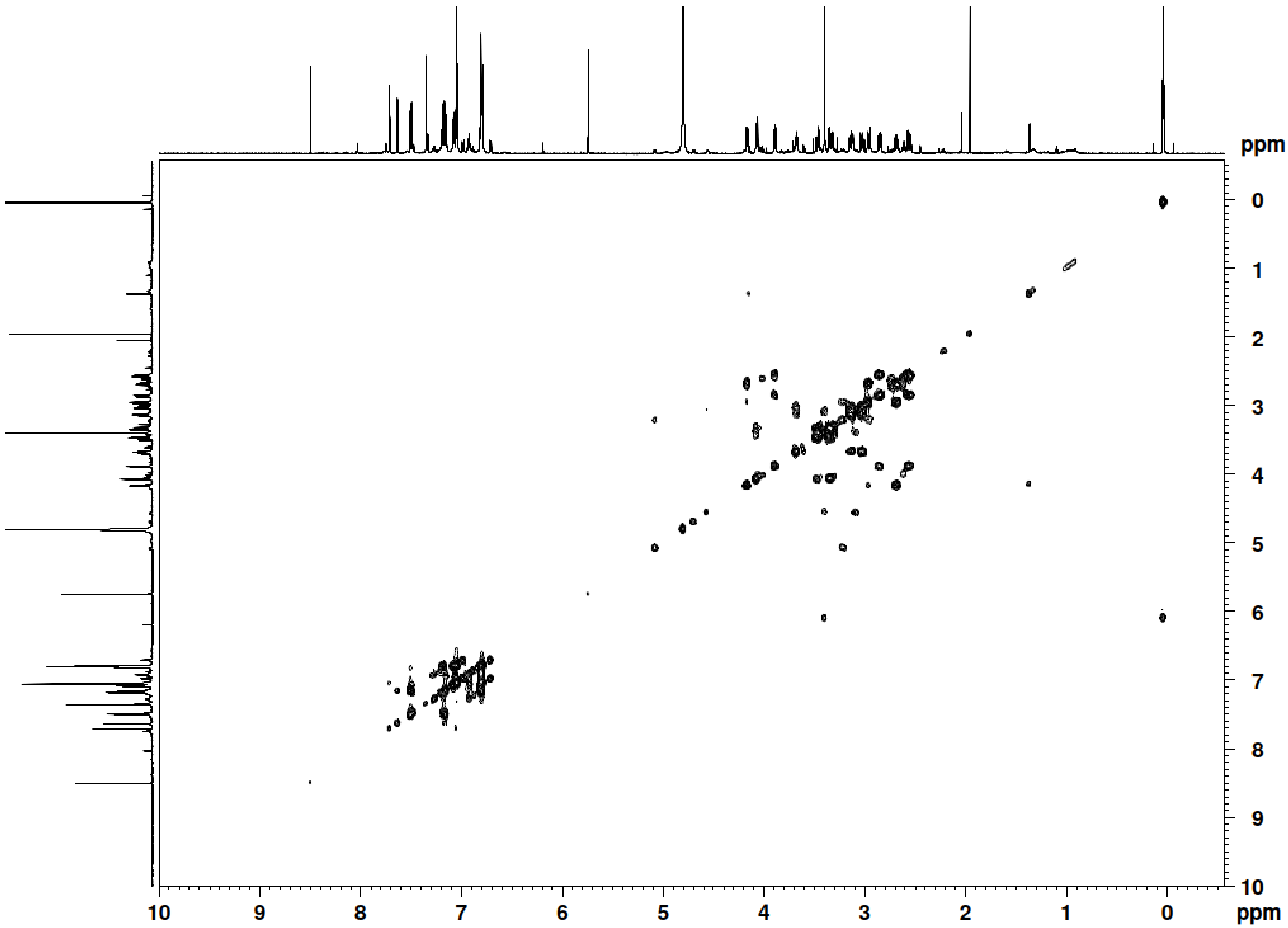

Figure S71. COSY spectrum (600 MHz) of **9** in D_2O.

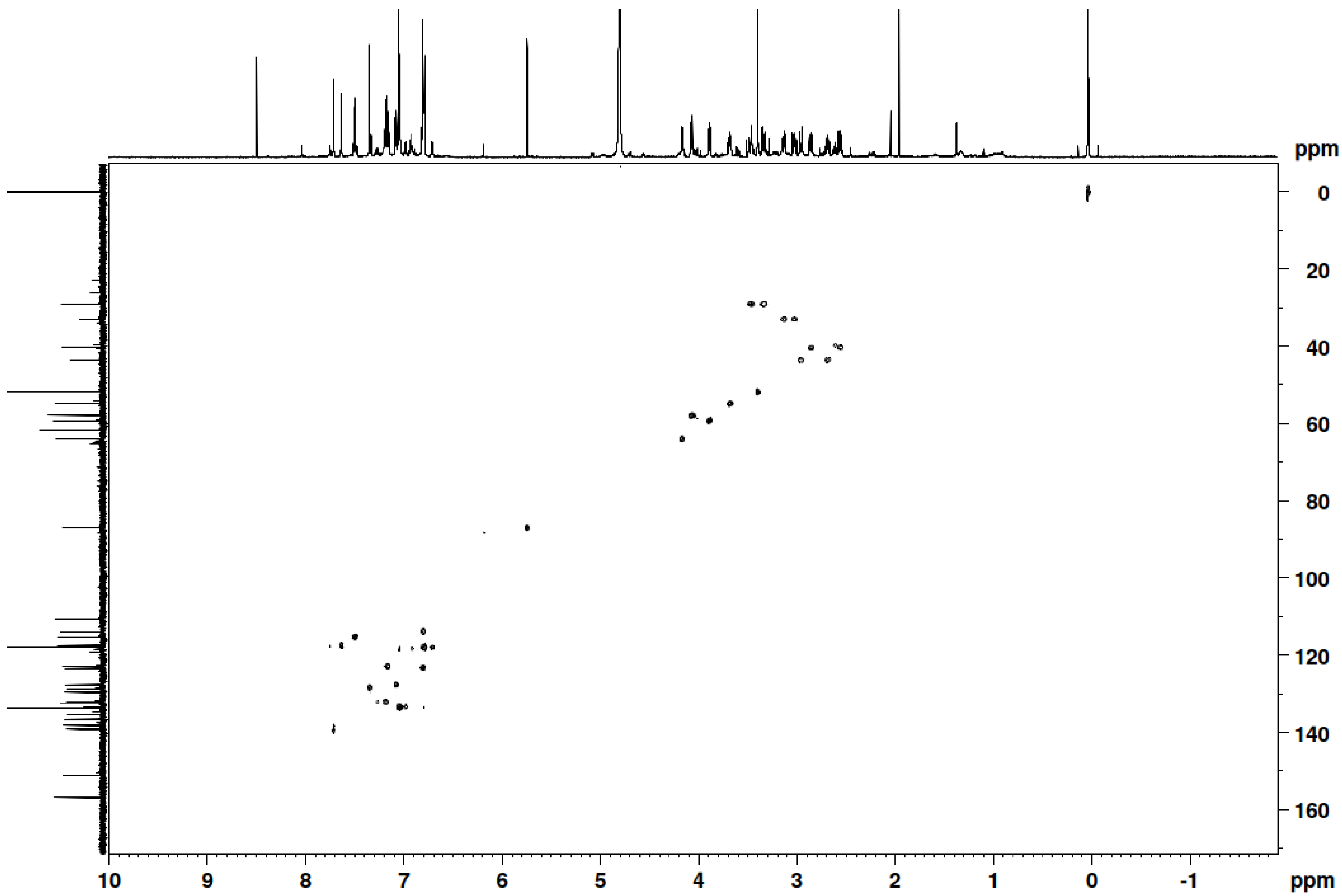

Figure S72. HSQC spectrum (500 MHz) of **9** in D_2O.

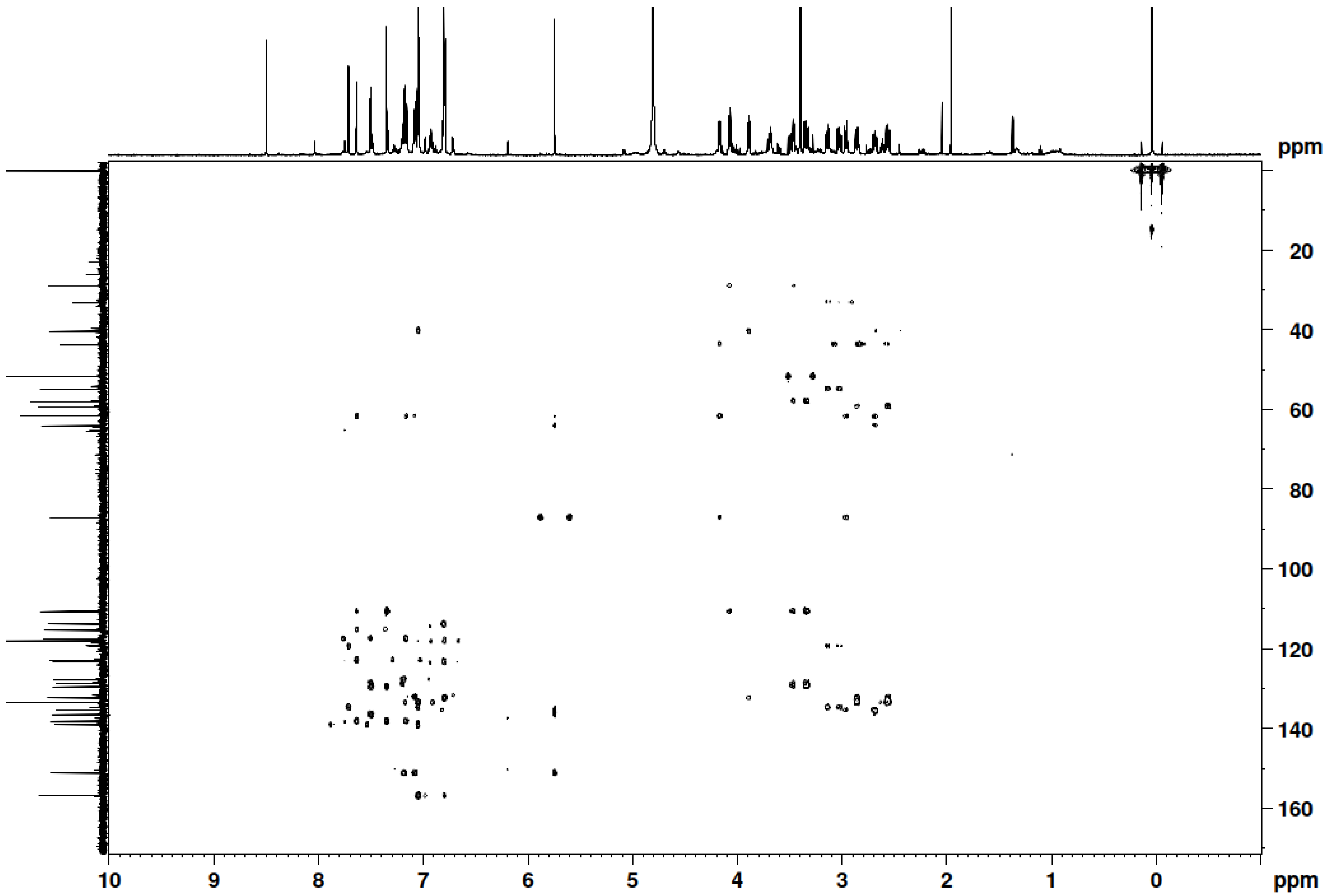

Figure S73. HMBC spectrum (500 MHz) of **9** in D_2O.

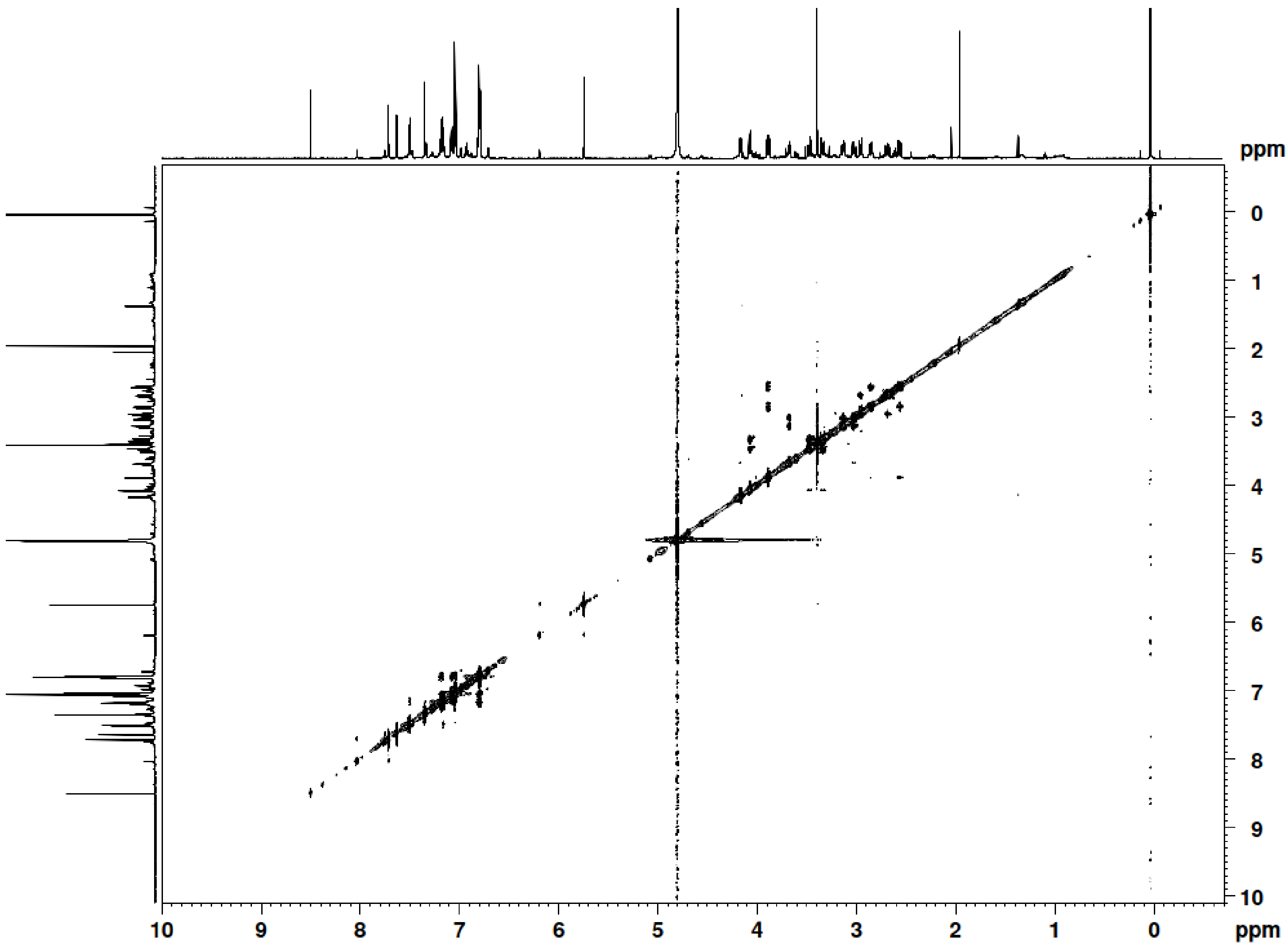

Figure S74. NOESY spectrum (500 MHz) of **9** in D_2O.

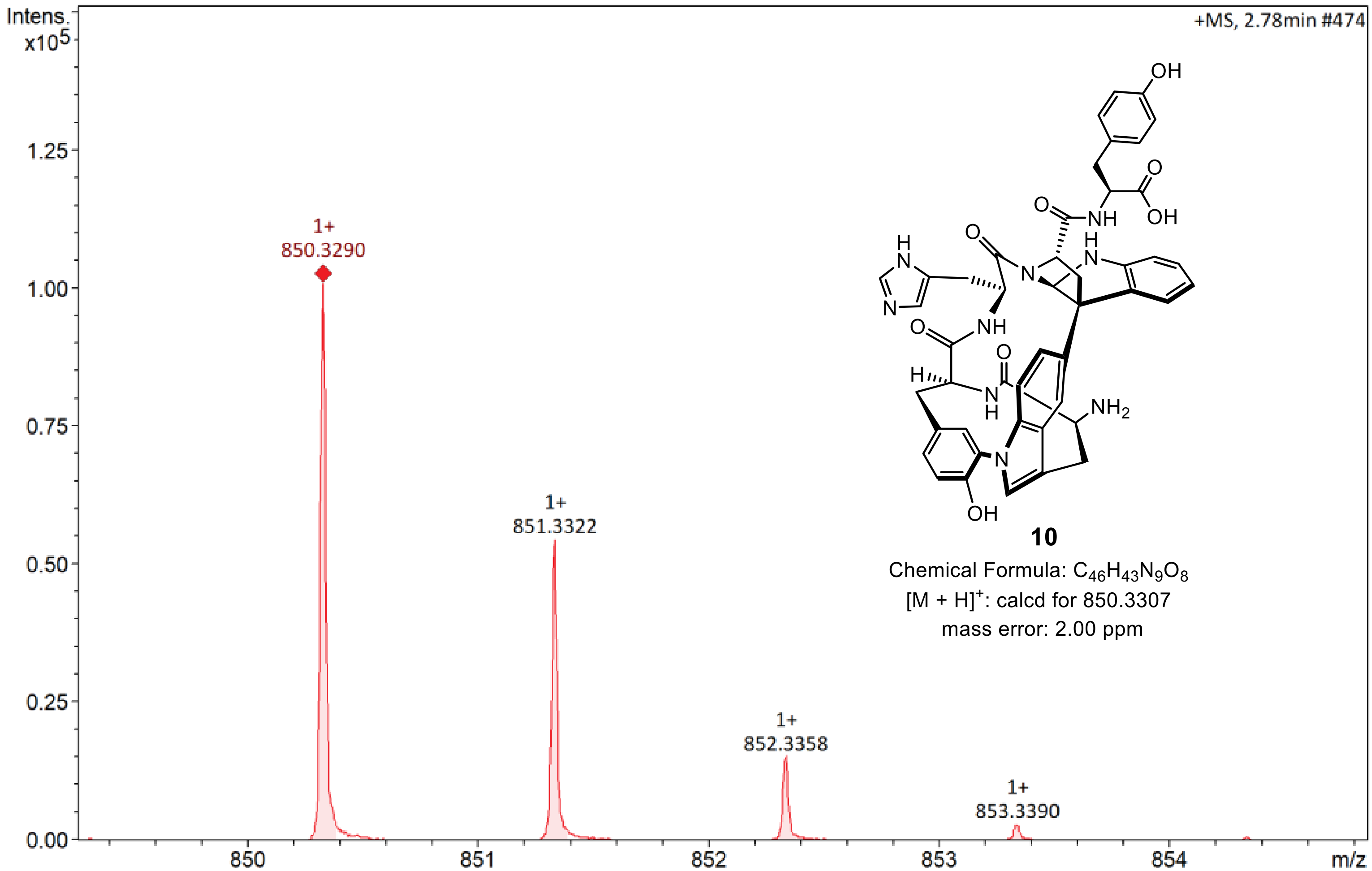

Figure S75. HPLC-ESI-QTOF-HRMS analysis of **10**.

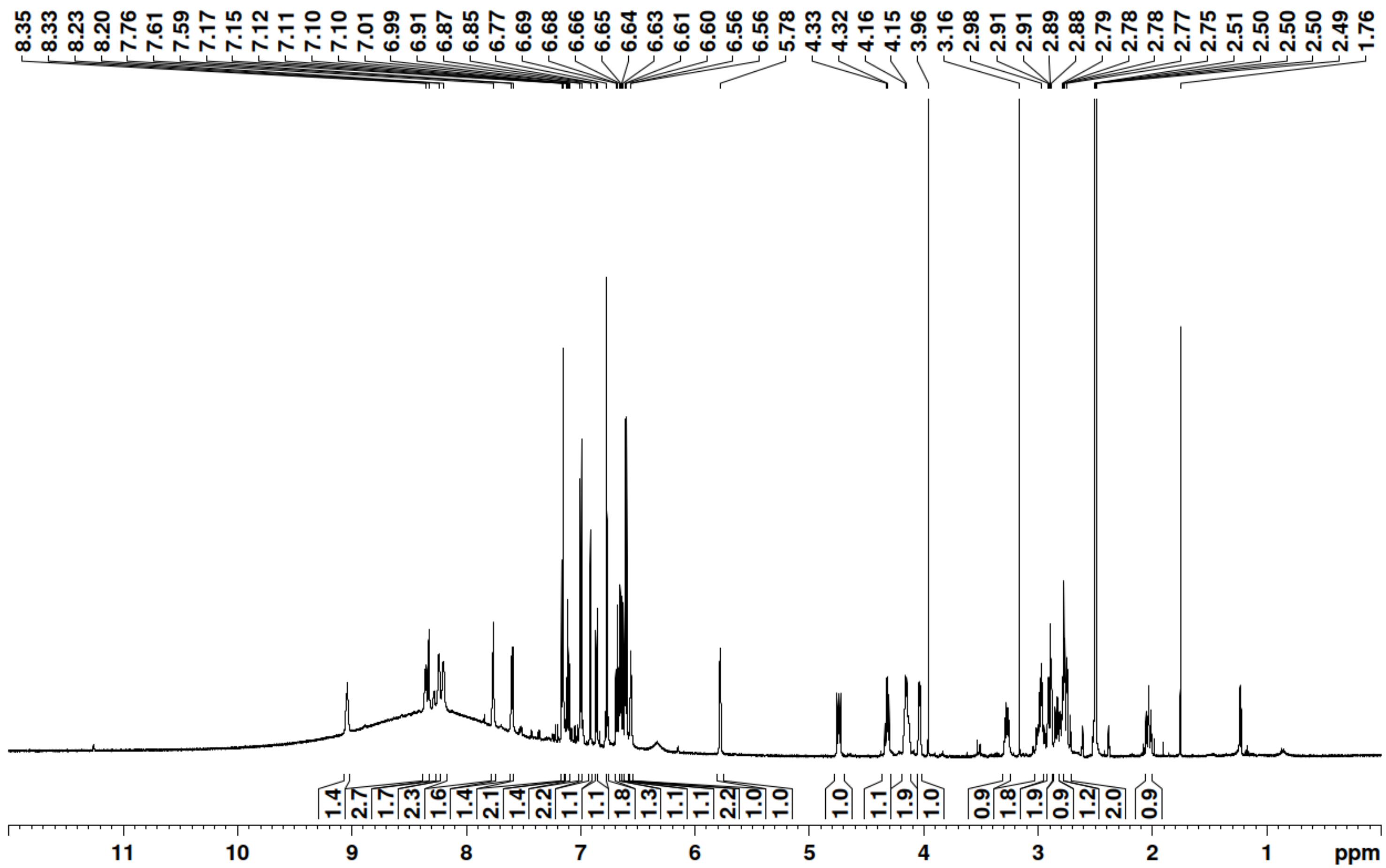

Figure S76. ^{1}H NMR spectrum (500 MHz) of **10** in DMSO-d_6.

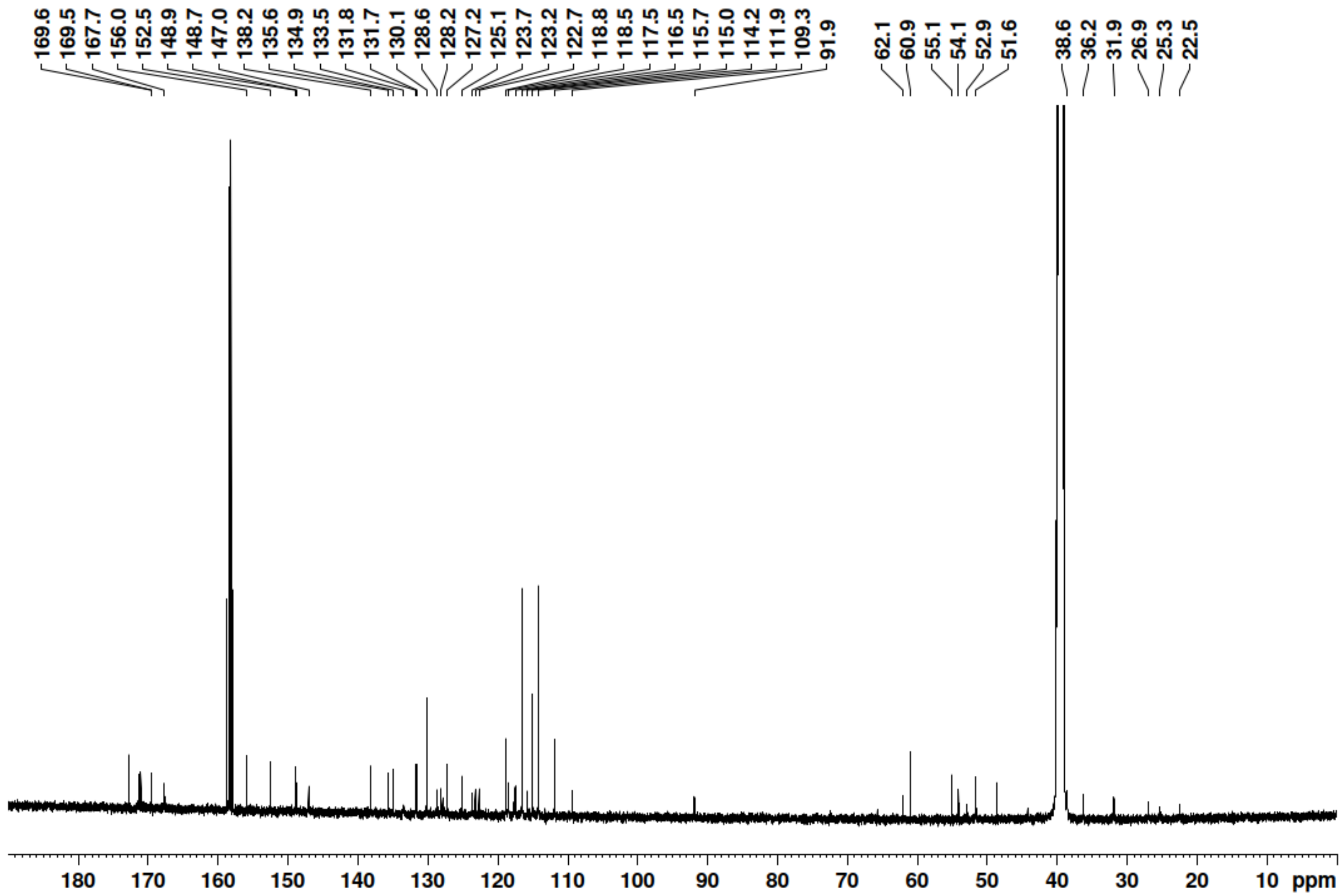

Figure S77. ^{13}C NMR spectrum (125 MHz) of **10** in DMSO-d_6.

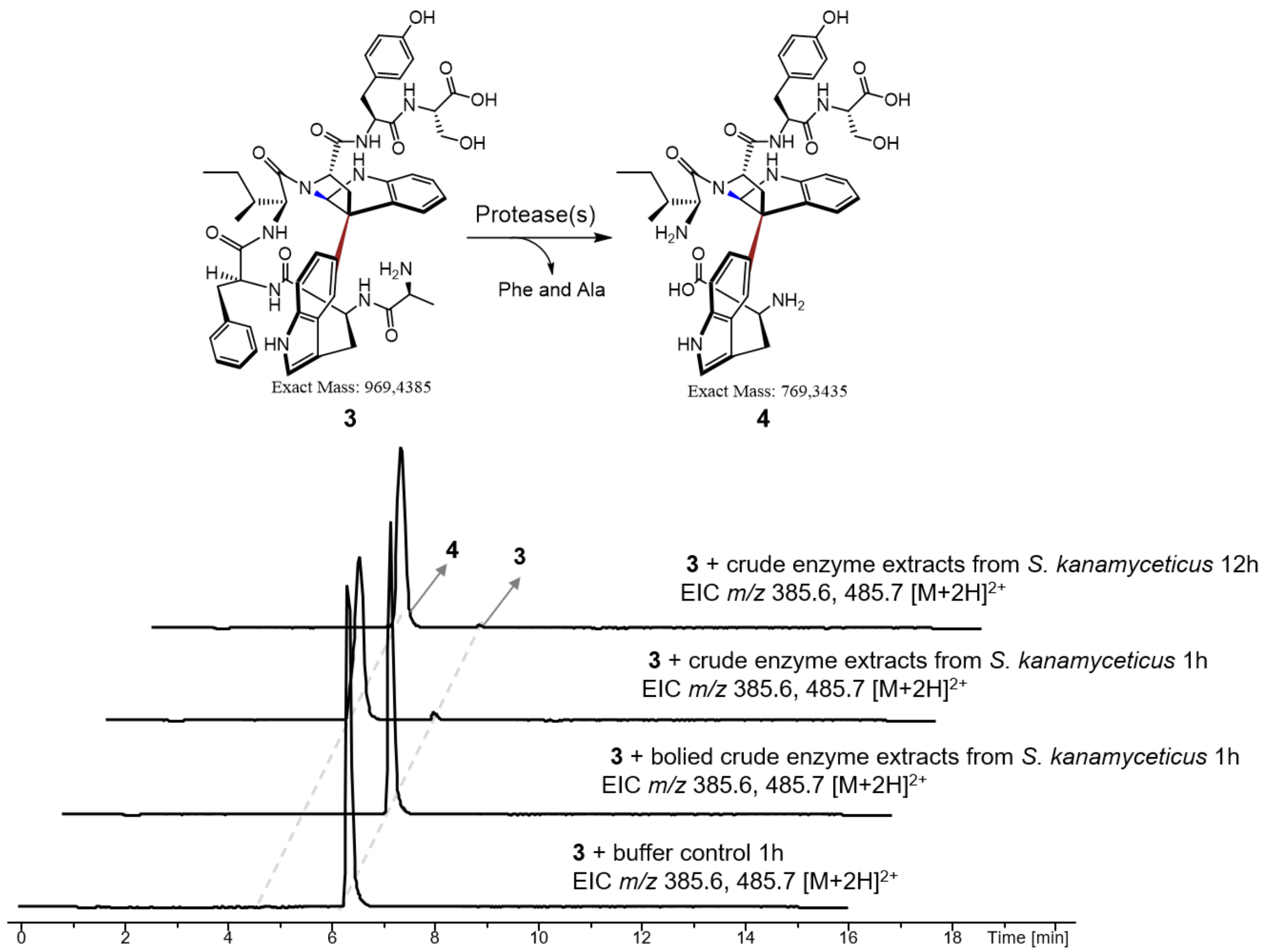

Figure S78. Bioconversion assays of **3** using crude enzyme extracts from *S. kanamyceticus*. Crude enzyme extracts from *S. kanamyceticus* converted compound **3** into **4,** whereas no product was observed with boiled crude enzyme extracts or the buffer control. Each 50 µL reaction mixture contained 0.05 mM **3** and either buffer (50 mM Tris-HCl, 50 mM NaCl, pH 7.5) or crude enzymes prepared in the same buffer or the boiled crude enzyme extracts. Reactions were performed at 30 °C for 1 h or 12 h.

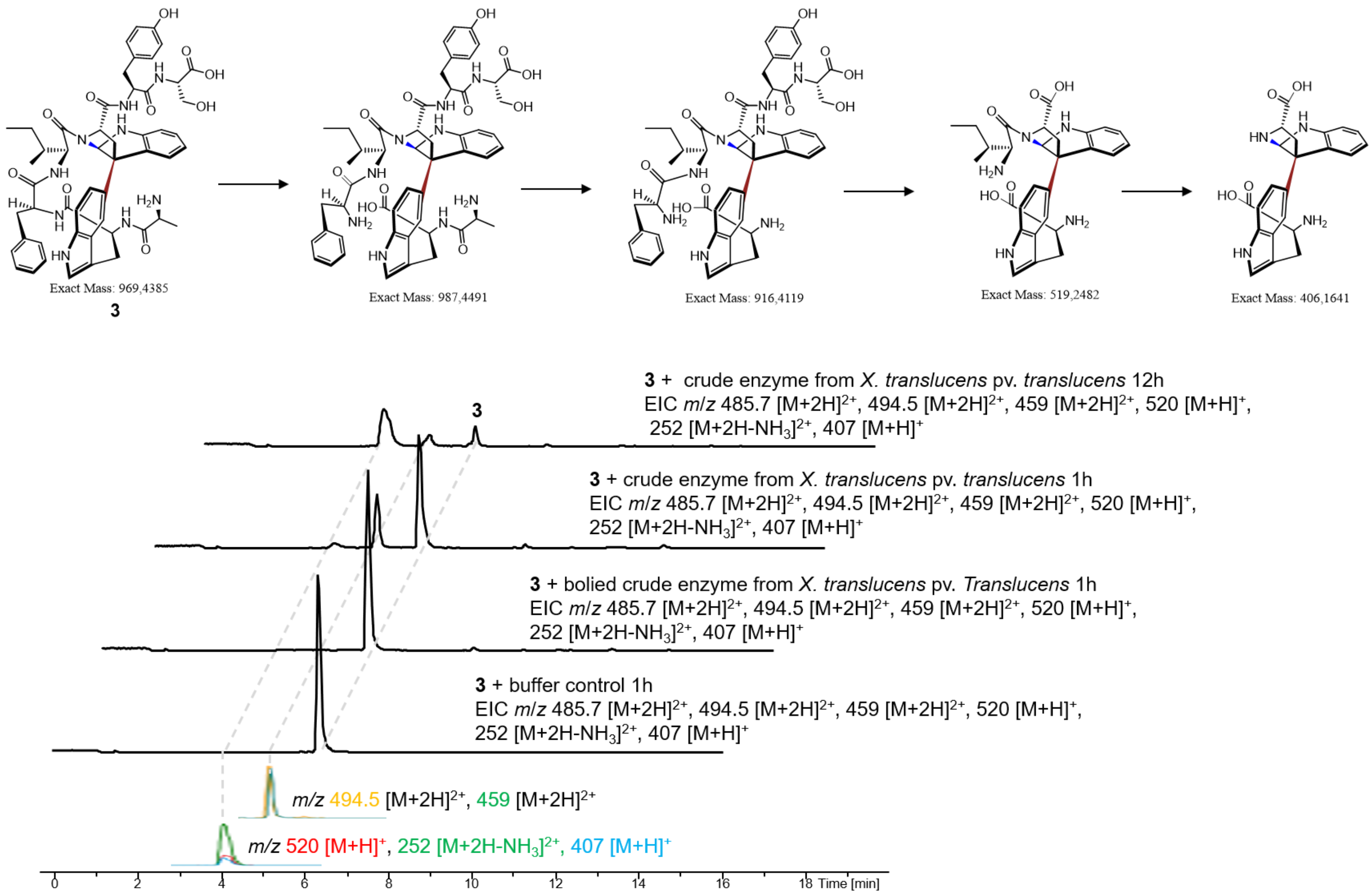

Figure S79. Bioconversion assays of **3** using crude enzyme extracts from *X. translucens* pv. translucens. Crude enzyme extracts from *X. translucens* pv. translucens converted compound **3** into **4,** whereas no product was observed with boiled crude enzyme extracts or the buffer control. Each 50 µL reaction mixture contained 0.05 mM **3** and either buffer (50 mM Tris-HCl, 50 mM NaCl, pH 7.5) or crude enzymes prepared in the same buffer or the boiled crude enzyme extracts. Reactions were performed at 30 °C for 1 h or 12.

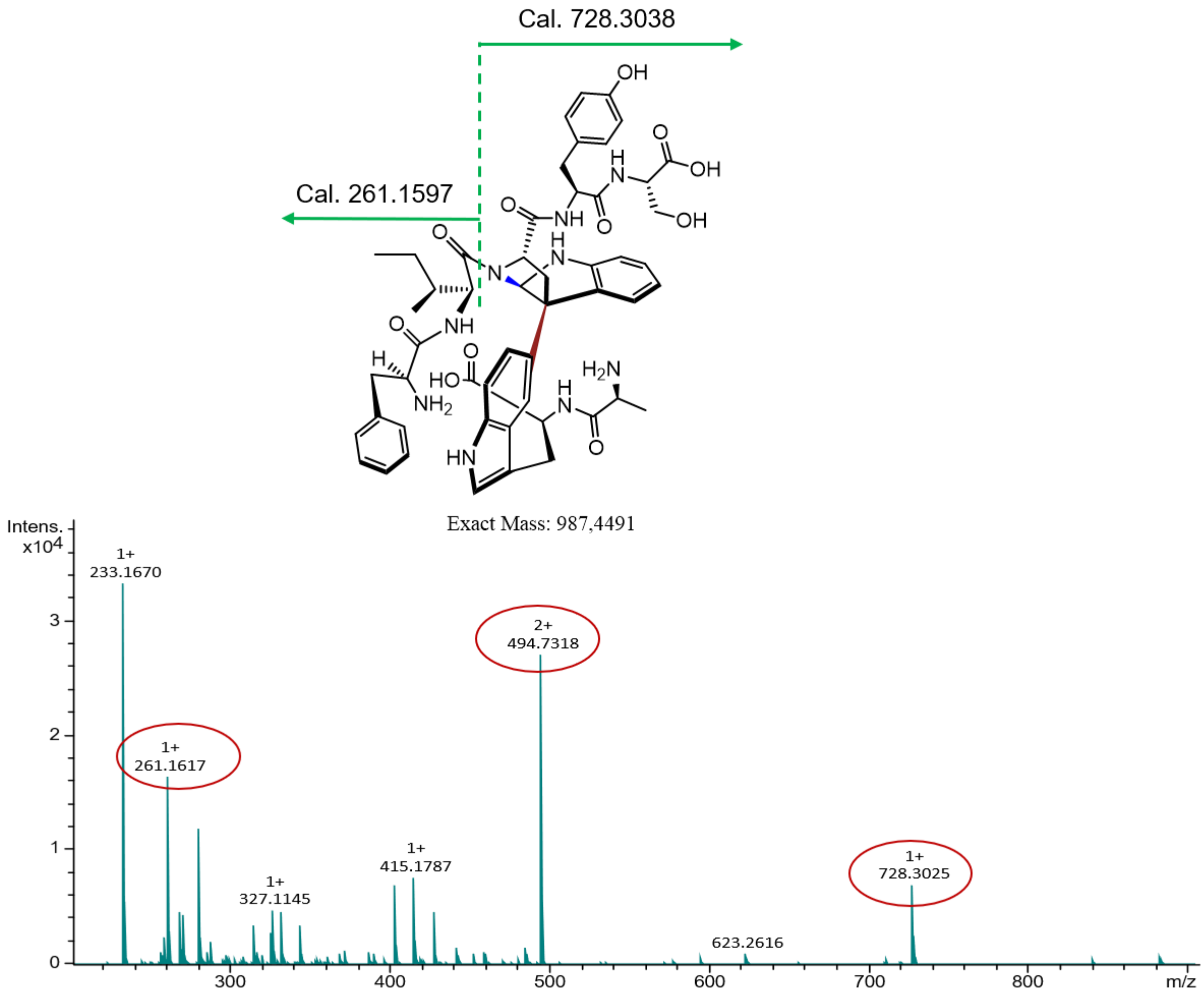

Figure S80. MS/MS spectrum of the atropopeptide detected at *m*/*z* 494.7318 $[M+2H]^{2+}$ with key fragments indicating the cleavage of the peptide bond between Trp and Phe highlighted.

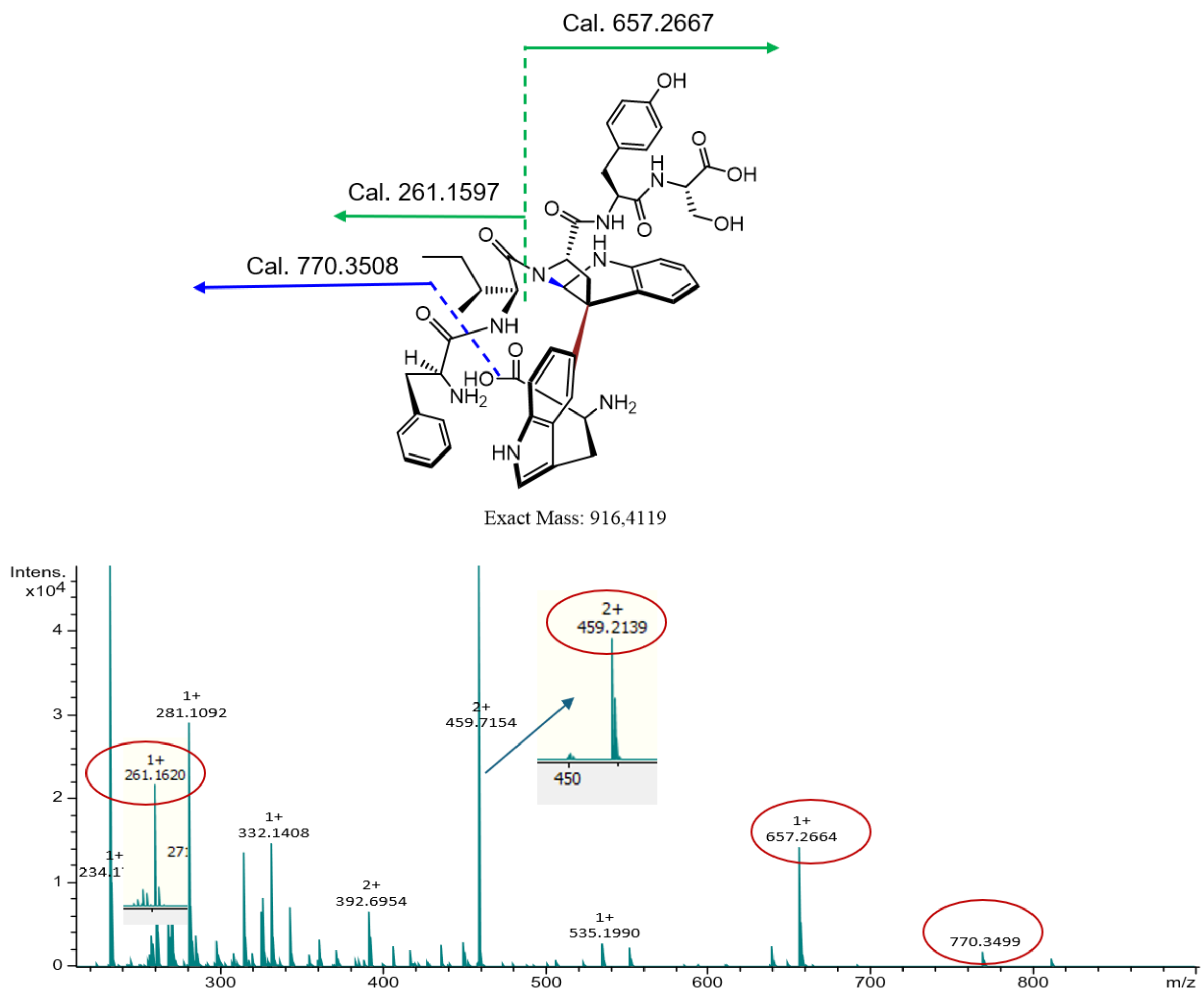

Figure S81. MS/MS spectrum of the atropopeptide detected at *m/z* 459.2139 $[M+2H]^{2+}$ with key fragments indicating the cleavage of the peptide bond between Trp and Phe highlighted.

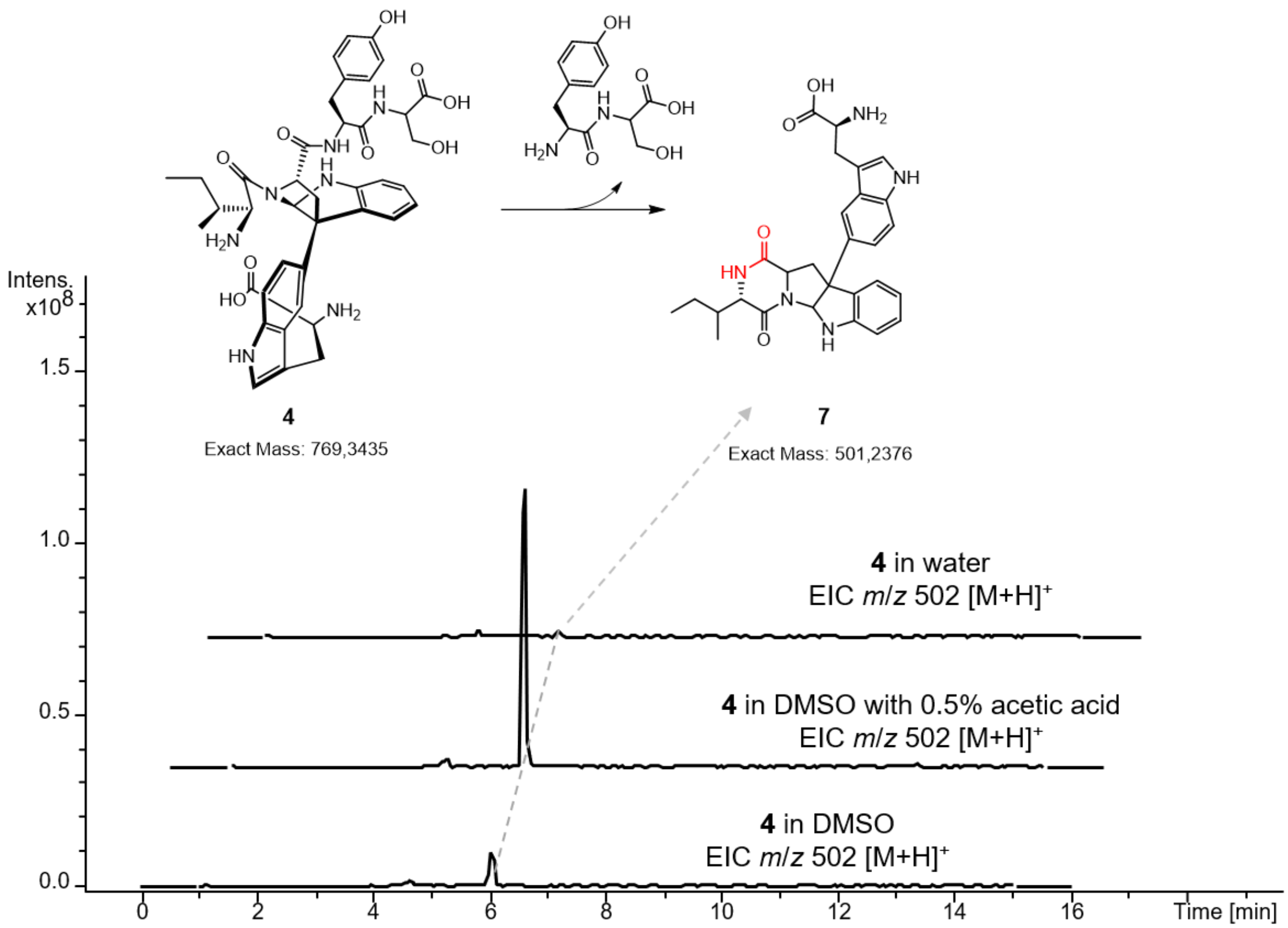

Figure S82. Spontaneous transformation of **4** into a diketopiperazine. Extracted ion chromatogram of the putative diketopiperazine (**7**) generated from **4** dissolved in different solvents (water, DMSO with 0.5% acetic acid, DMSO).

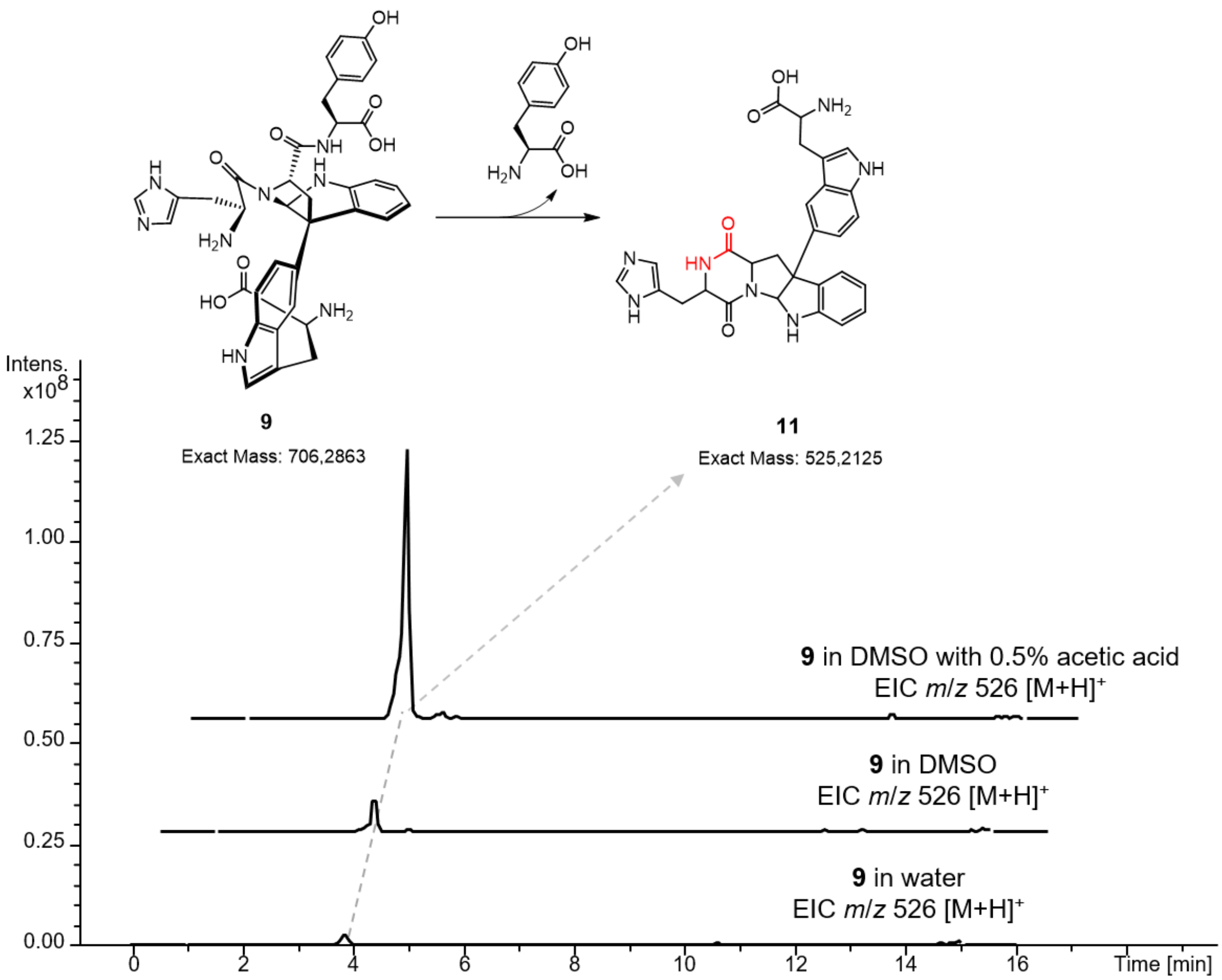

Figure S83. Spontaneous transformation of **9** into a diketopiperazine. Extracted ion chromatogram of the putative diketopiperazine (**11**) generated from **9** dissolved in different solvents (water, DMSO with 0.5% acetic acid, DMSO).

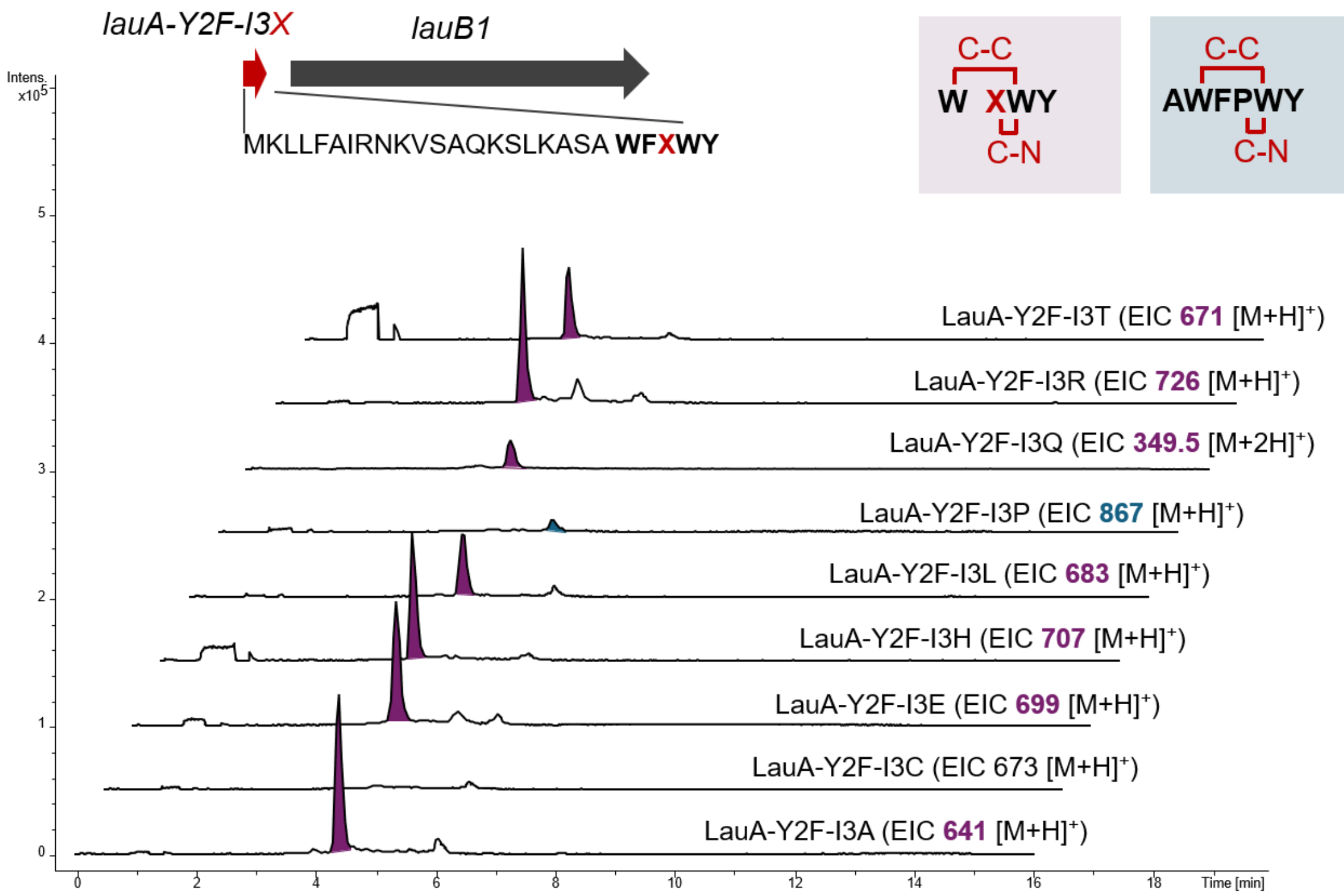

Figure S84. Coexpression of *lauA-Y2F-I3X* variants with *lauB1*. Extracted ion chromatogram of products generated from the coexpression of *lauA-Y2F-I3X* with *lauB1*.

A

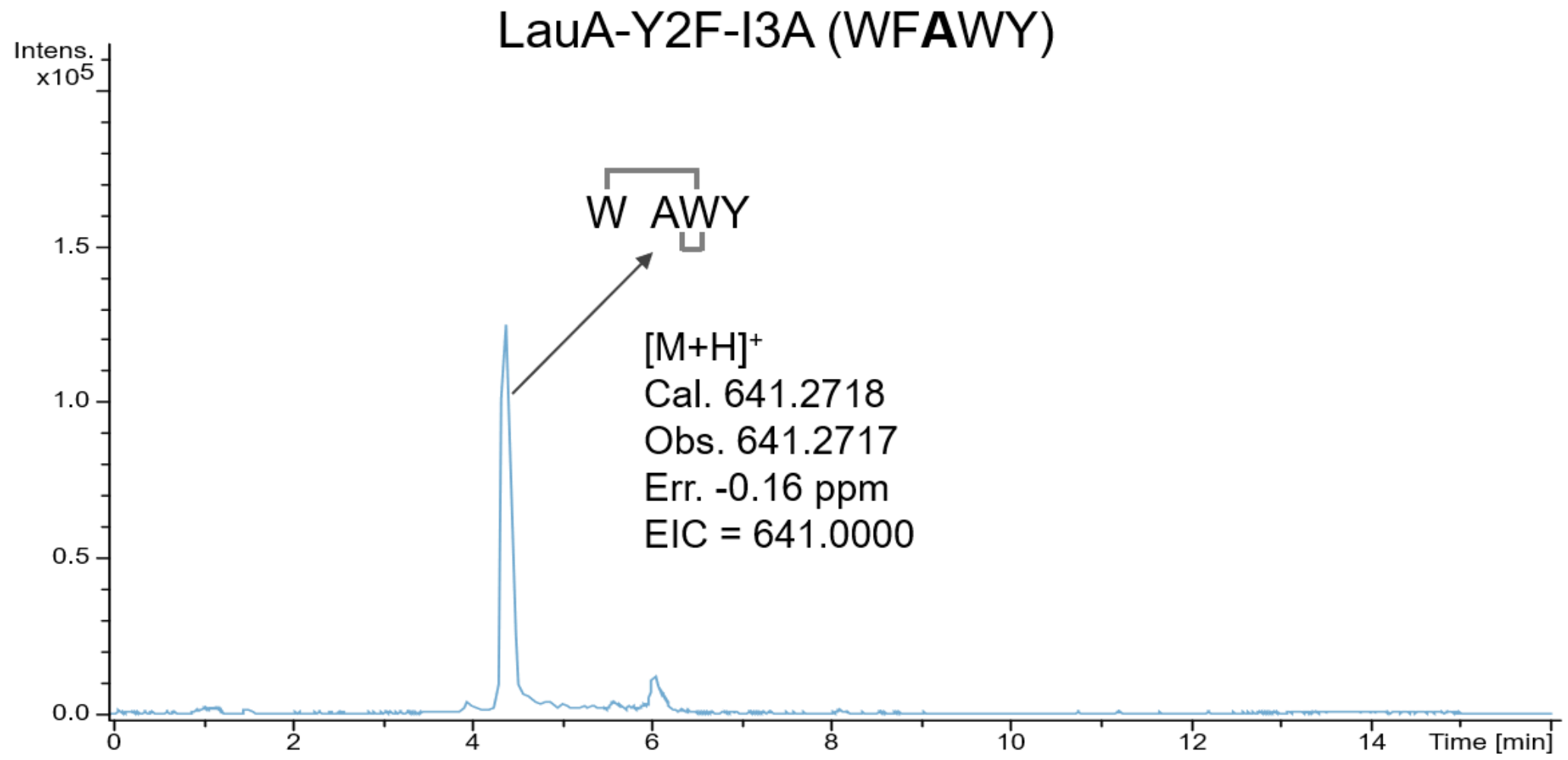

B

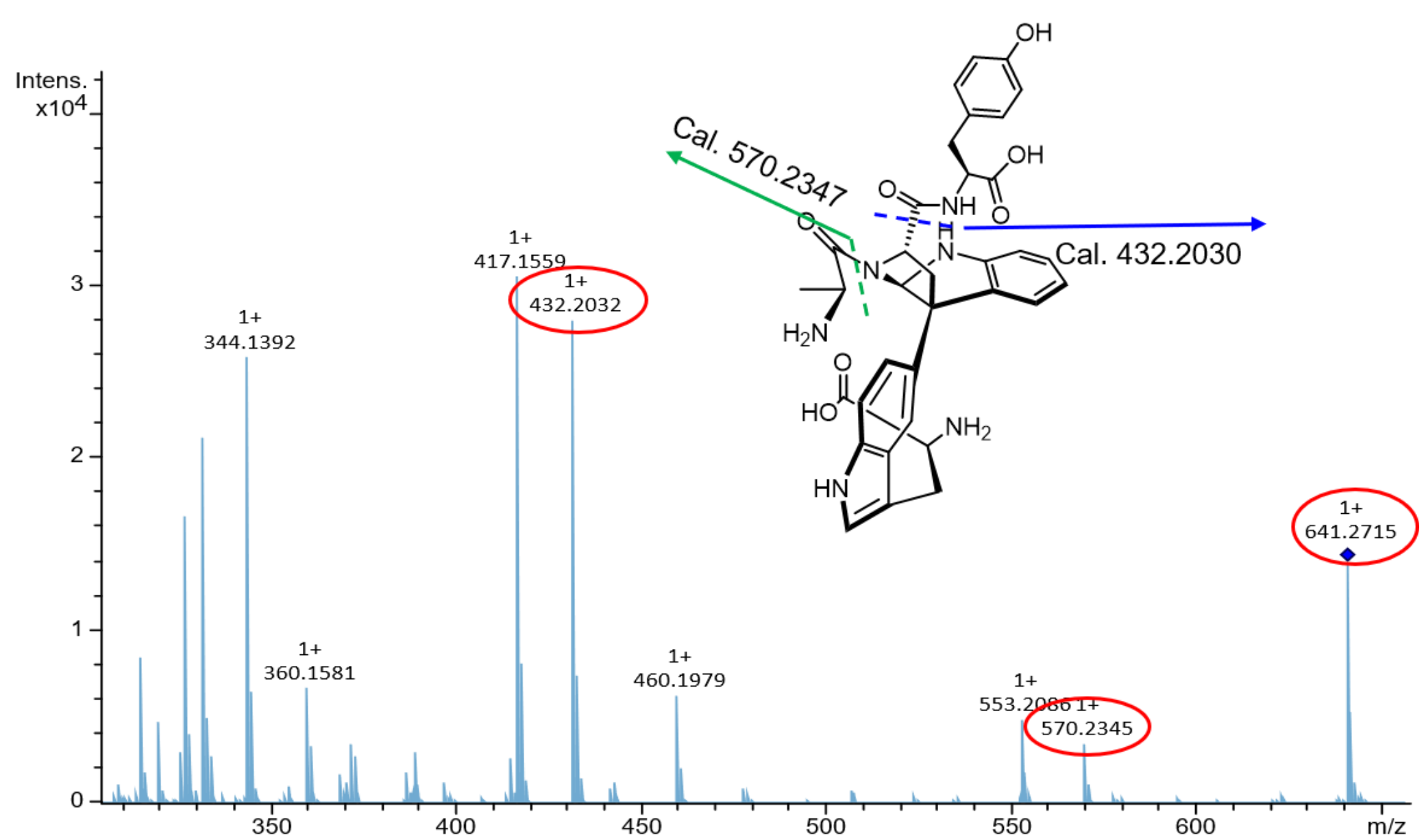

Figure S85. HRMS analysis of extracts of the recombinant *S. albus* strain harboring *lauA*-Y2F-I3A and *lauB1*. A) Extracted ion chromatogram of the product from the coexpression of *lauA*-Y2F-I3A with *lauB1*; B) MS/MS spectrum of the corresponding atropopeptide detected at *m*/*z* 641.2717 $[M+H]^+$ with key fragments indicating the loss of phenylalanine at the second position of the core peptide highlighted.

A

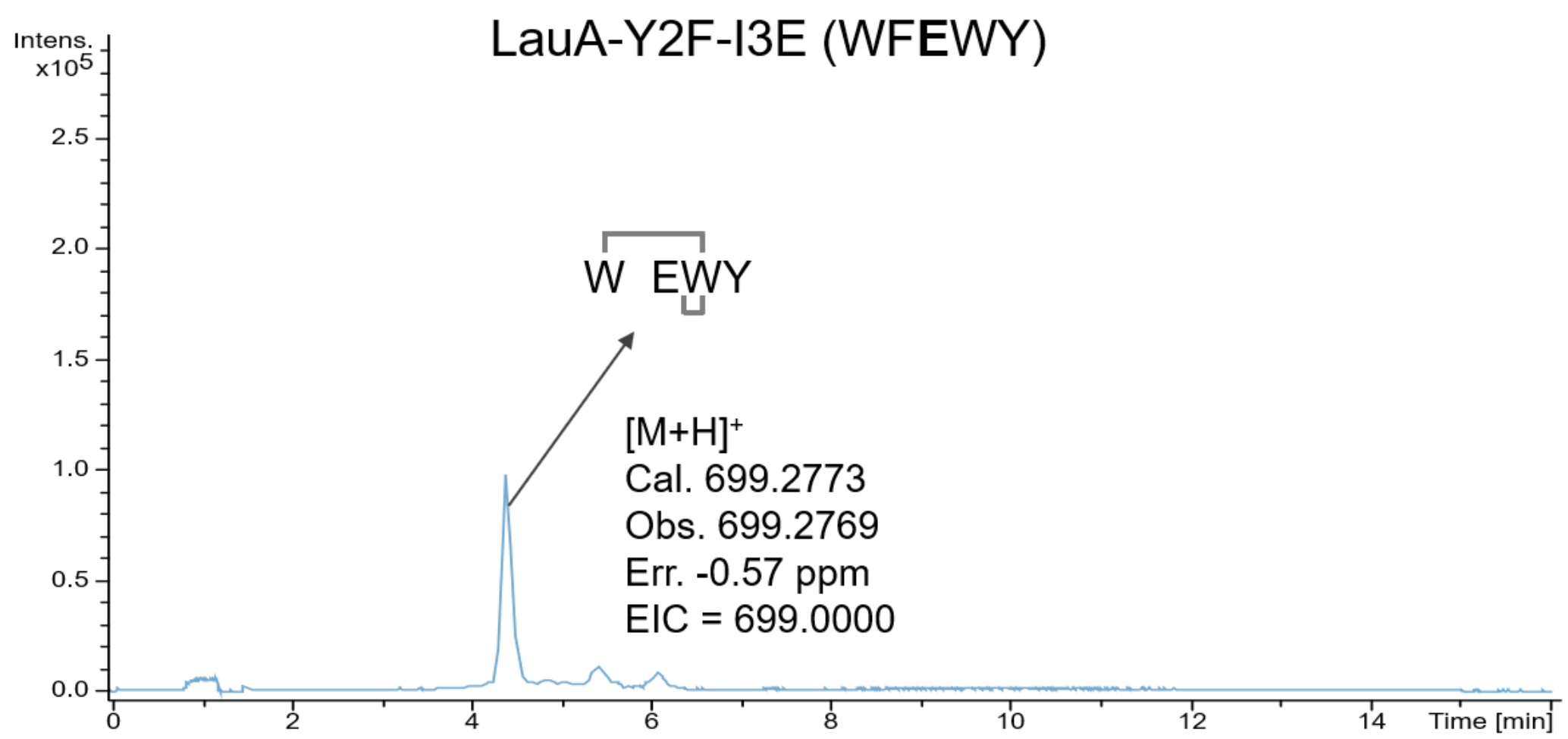

B

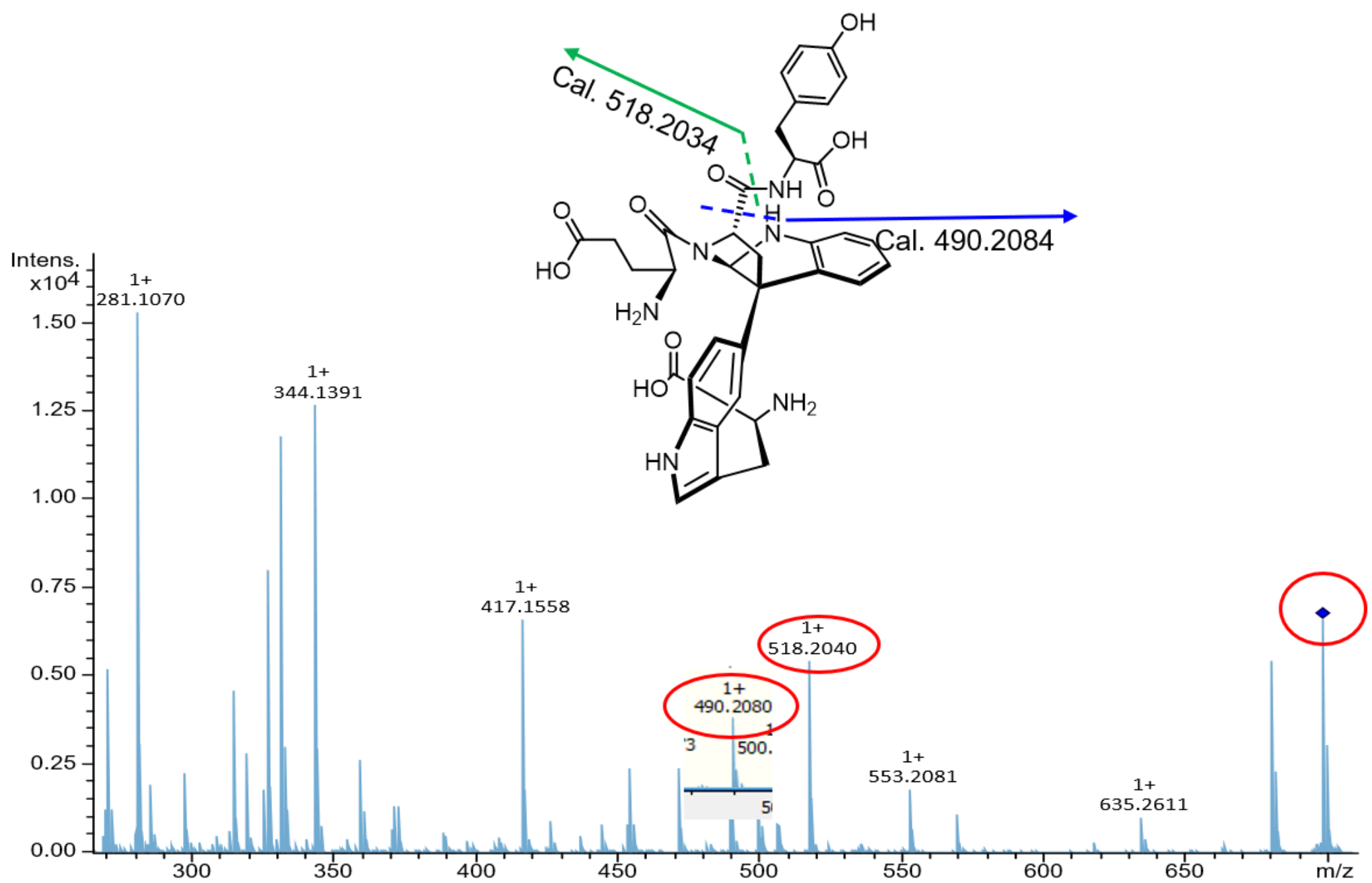

Figure S86. HRMS analysis of extracts of the recombinant *S. albus* strain harboring *lauA*-Y2F-I3E and *lauB1*. A) Extracted ion chromatogram of the product from the coexpression of *lauA*-Y2F-I3E with *lauB1*; B) MS/MS spectrum of the corresponding atropopeptide detected at *m*/*z* 699.2769 $[M+H]^+$ with key fragments indicating the loss of phenylalanine at the second position of the core peptide highlighted.

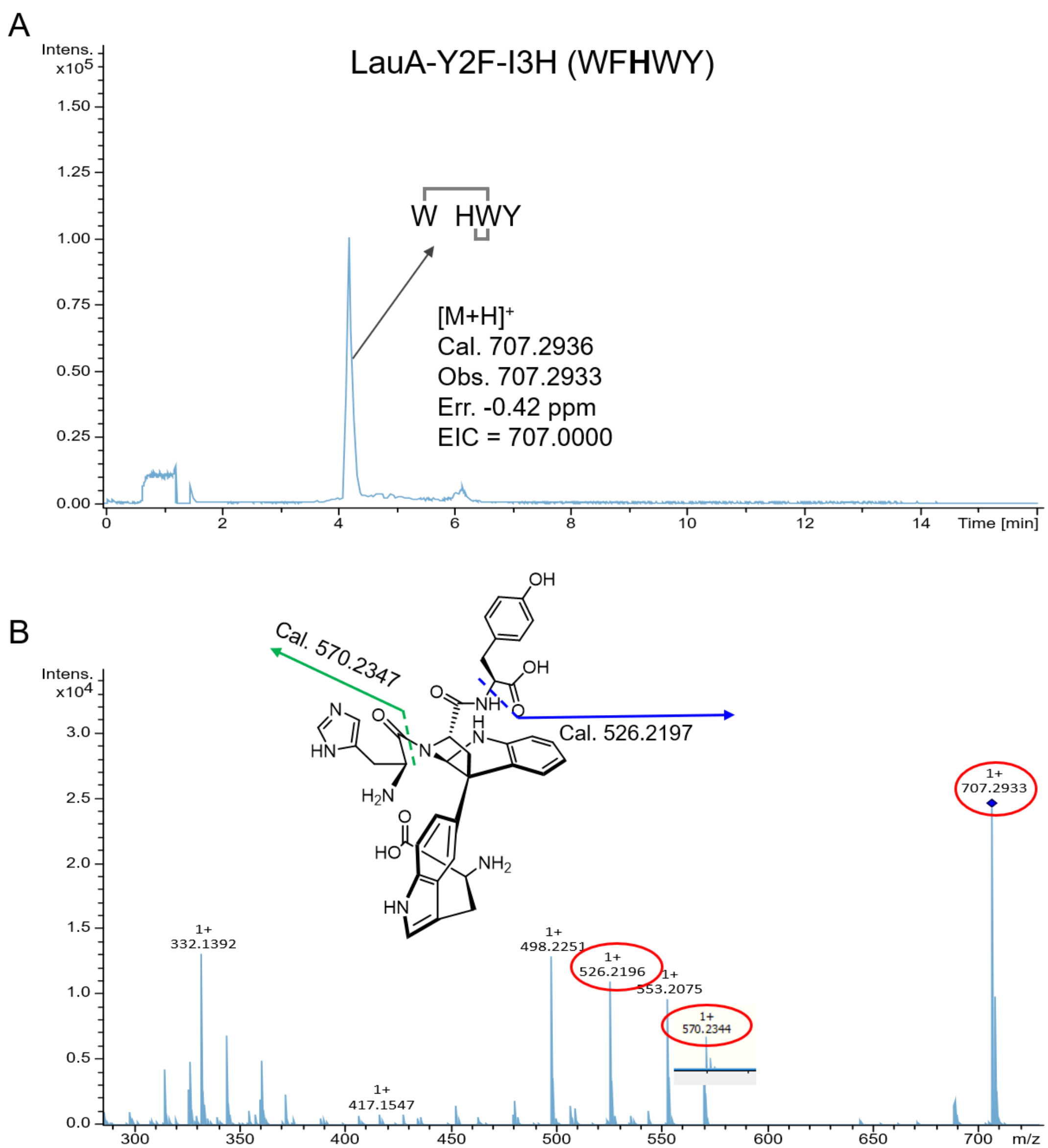

Figure S87. HRMS analysis of extracts of the recombinant *S. albus* strain harboring *lauA*-Y2F-I3H and *lauB1*. A) Extracted ion chromatogram of the product from the coexpression of *lauA*-Y2F-I3H with *lauB1*; B) MS/MS spectrum of the corresponding atropopeptide detected at *m*/*z* 707.2933 $[M+H]^+$ with key fragments indicating the loss of phenylalanine at the second position of the core peptide highlighted.

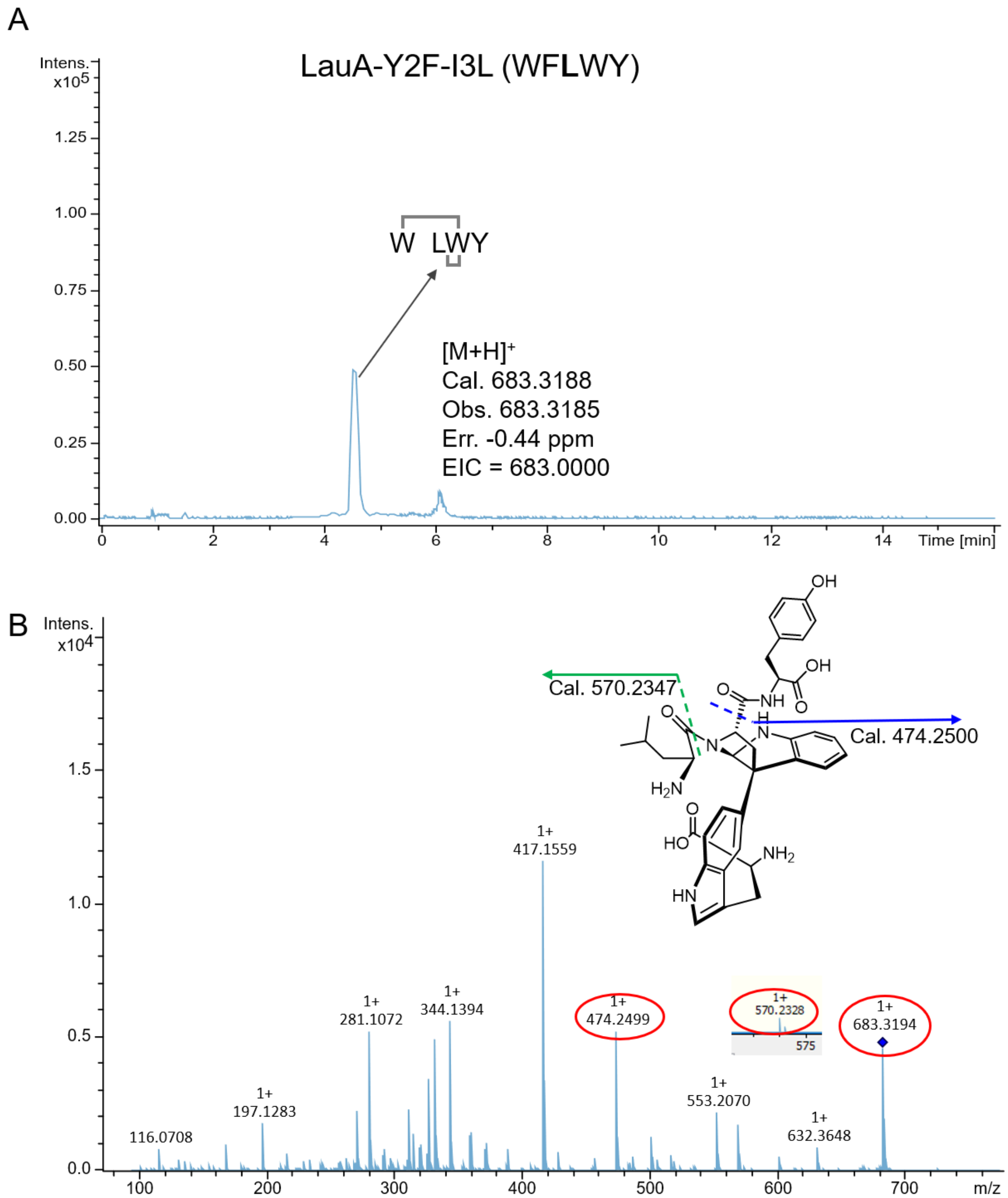

Figure S88. HRMS analysis of extracts of the recombinant *S. albus* strain harboring *lauA*-Y2F-I3L and *lauB1*. A) Extracted ion chromatogram of the product from the coexpression of *lauA*-Y2F-I3L with *lauB1*; B) MS/MS spectrum of the corresponding atropopeptide detected at *m*/*z* 683.3185 $[M+H]^+$ with key fragments indicating the loss of phenylalanine at the second position of the core peptide highlighted.

A

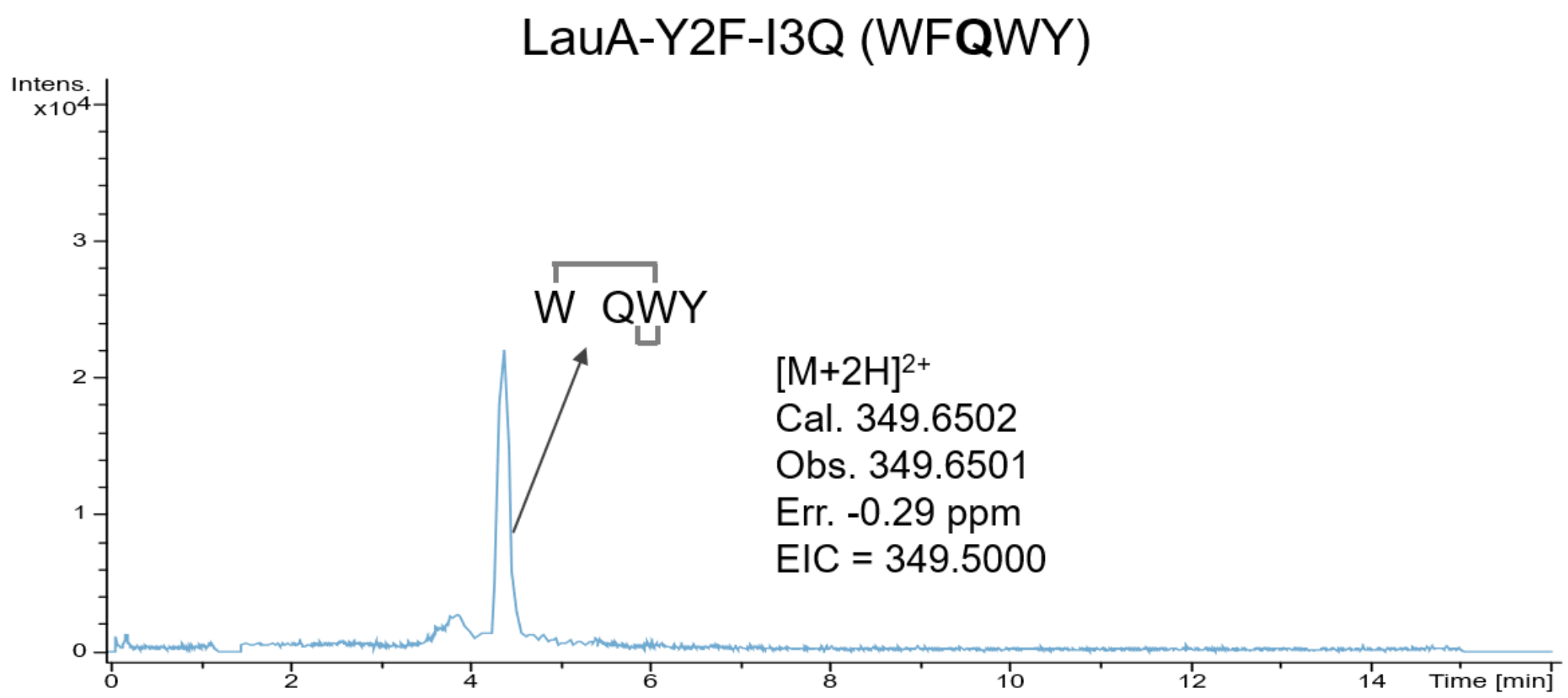

B

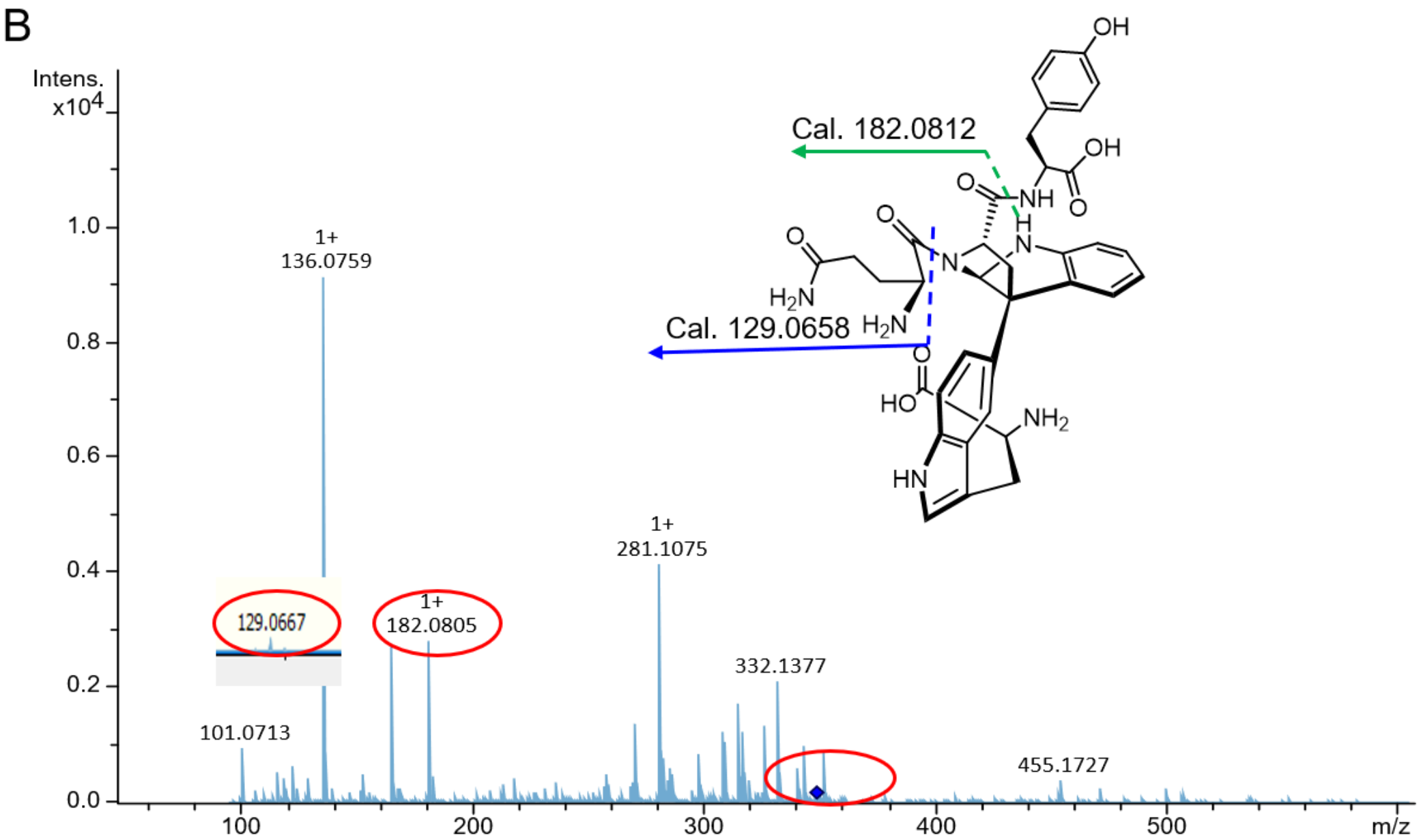

Figure S89. HRMS analysis of extracts of the recombinant *S. albus* strain harboring *lauA*-Y2F-I3Q and *lauB1*. A) Extracted ion chromatogram of the product from the coexpression of *lauA*-Y2F-I3Q with *lauB1*; B) MS/MS spectrum of the corresponding atropopeptide detected at *m*/*z* 349.6501 $[M+2H]^{2+}$ with key fragments indicating the loss of phenylalanine at the second position of the core peptide highlighted.

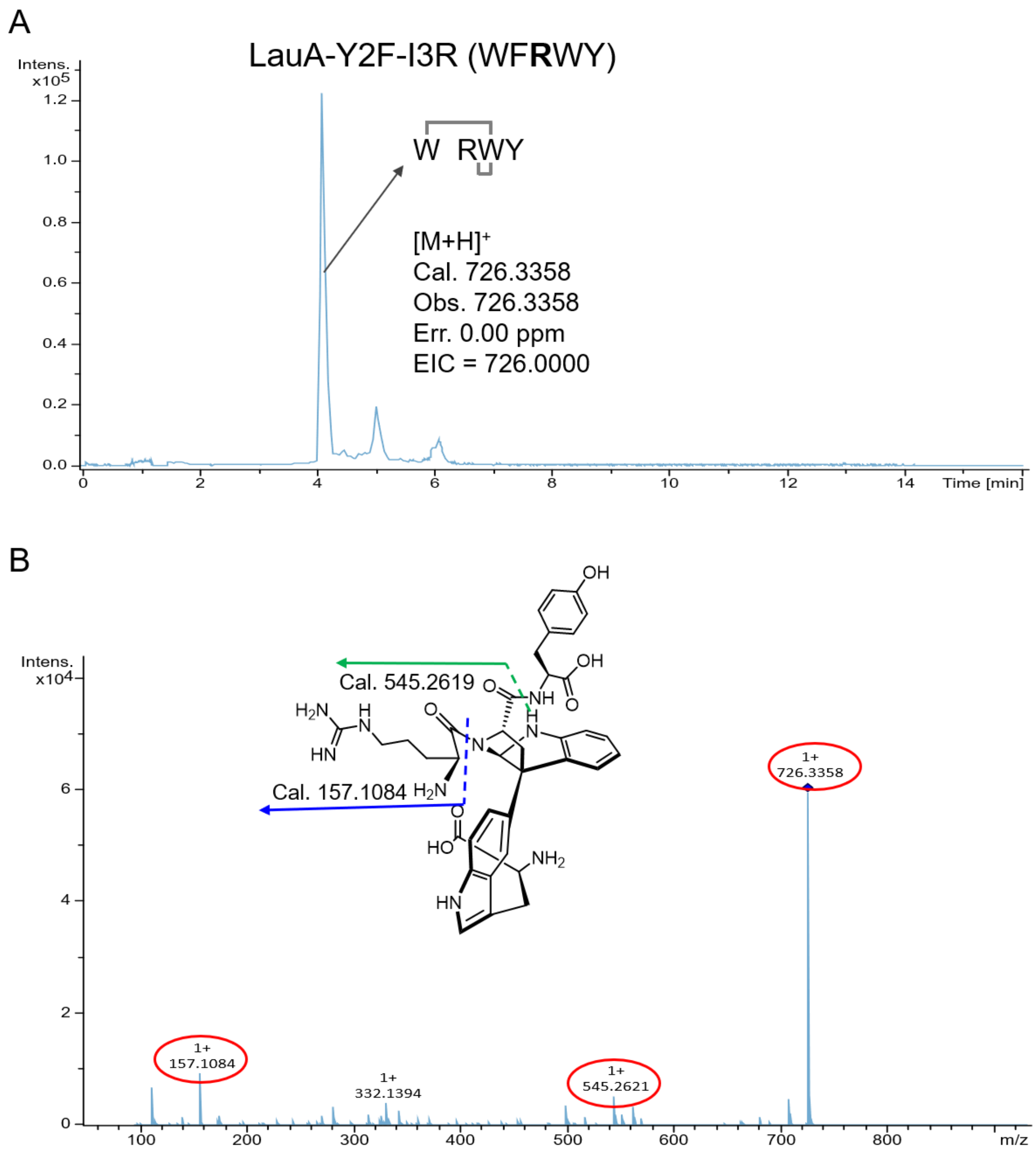

Figure S90. HRMS analysis of extracts of the recombinant *S. albus* strain harboring *lauA*-Y2F-I3R and *lauB1*. A) Extracted ion chromatogram of the product from the coexpression of *lauA*-Y2F-I3R with *lauB1*; B) MS/MS spectrum of the corresponding atropopeptide detected at *m*/*z* 726.3358 $[M+H]^+$ with key fragments indicating the loss of phenylalanine at the second position of the core peptide highlighted.

A

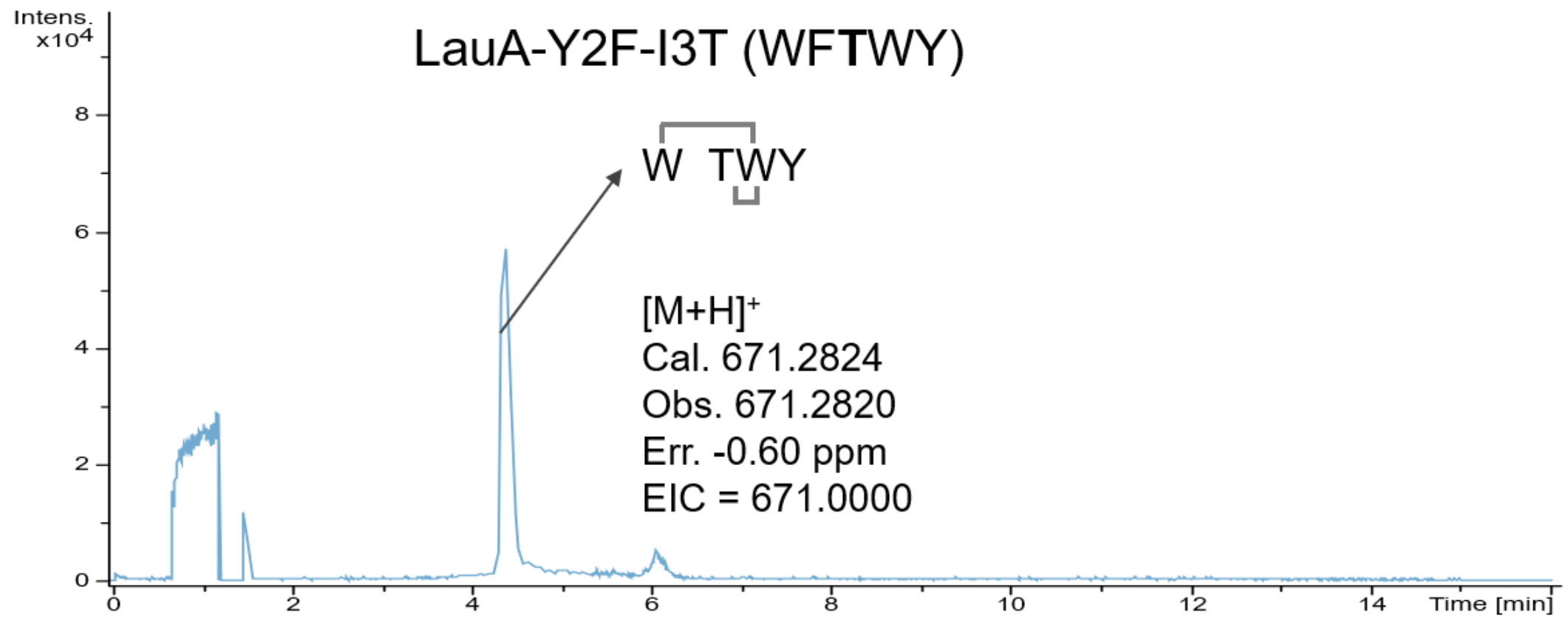

B

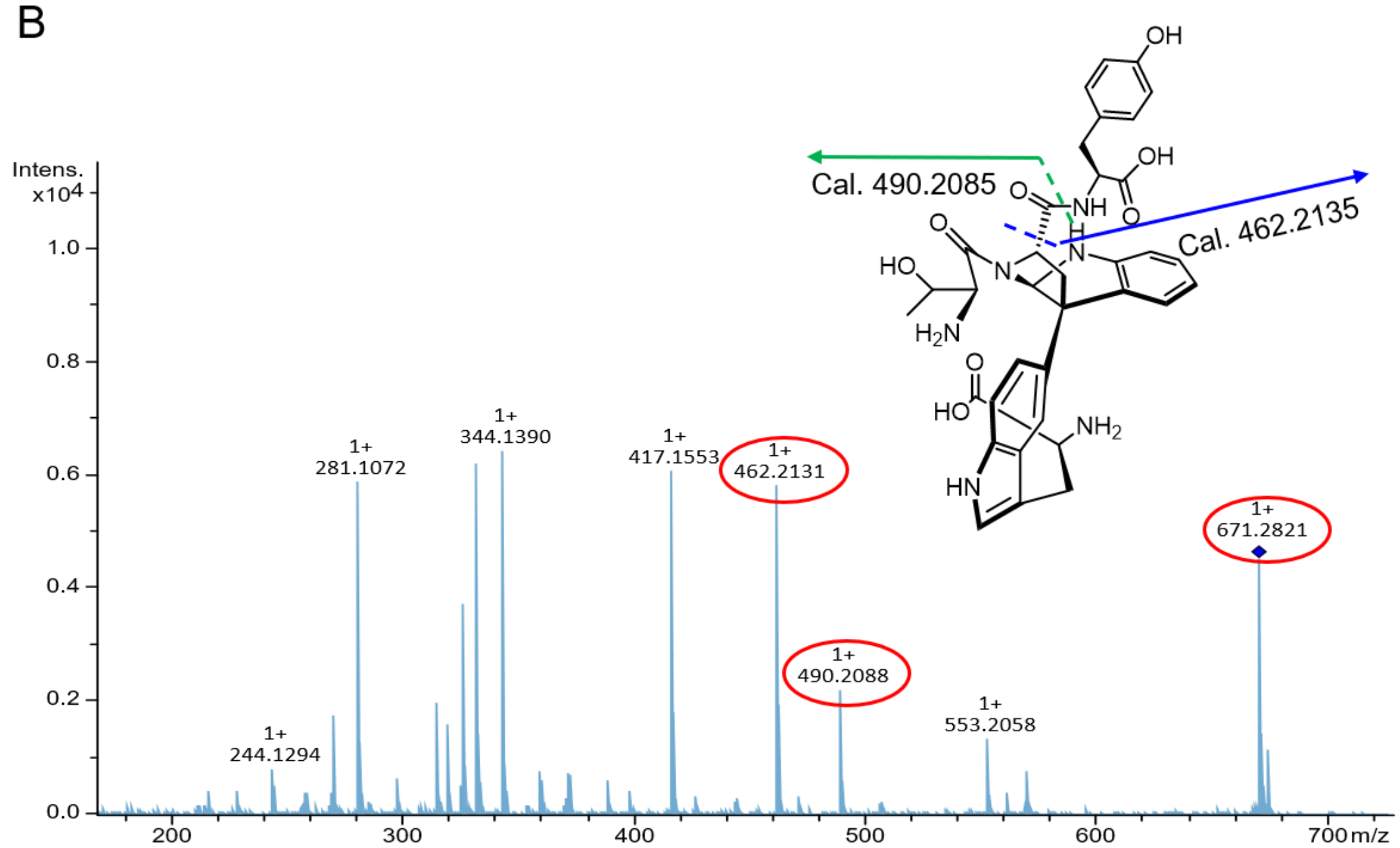

Figure S91. HRMS analysis of extracts of the recombinant *S. albus* strain harboring *lauA*-Y2F-I3T and *lauB1*. A) Extracted ion chromatogram of the product from the coexpression of *lauA*-Y2F-I3T with *lauB1*; B) MS/MS spectrum of the corresponding atropopeptide detected at *m*/*z* 671.2820 $[M+H]^+$ with key fragments indicating the loss of phenylalanine at the second position of the core peptide highlighted.

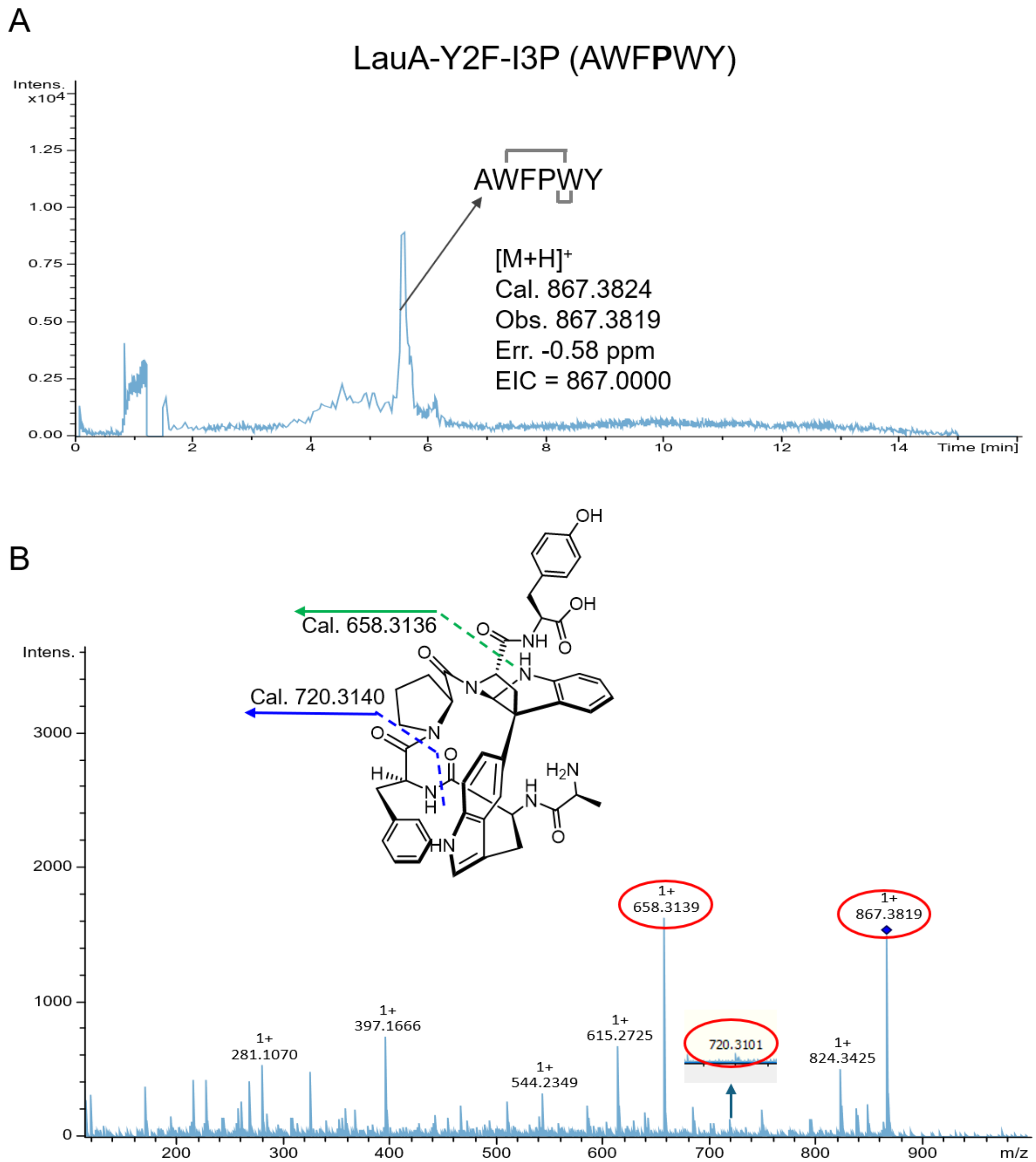

Figure S92. HRMS analysis of extracts of the recombinant *S. albus* strain harboring *lauA*-Y2F-I3P and *lauB1*. A) Extracted ion chromatogram of the product from the coexpression of *lauA*-Y2F-I3P with *lauB1*; B) MS/MS spectrum of the corresponding atropopeptide detected at *m*/*z* 867.3819 $[M+H]^+$ with key fragments indication the presence of the proline highlighted.

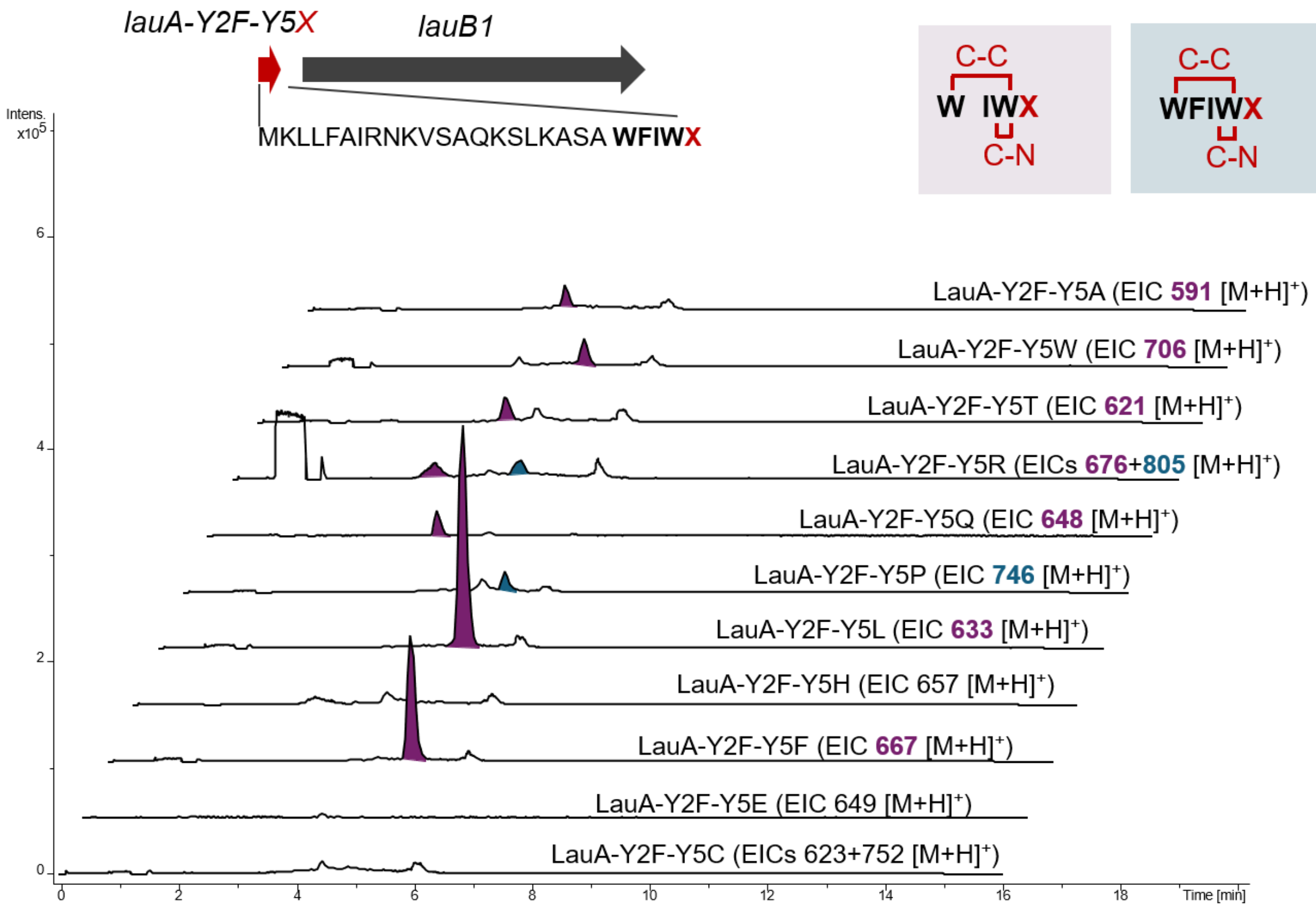

Figure S93. Coexpression of *lauA-Y2F-Y5X* variants with *lauB1*. Extracted ion chromatogram of products generated from the coexpression of *lauA-Y2F-Y5X* with *lauB1*.

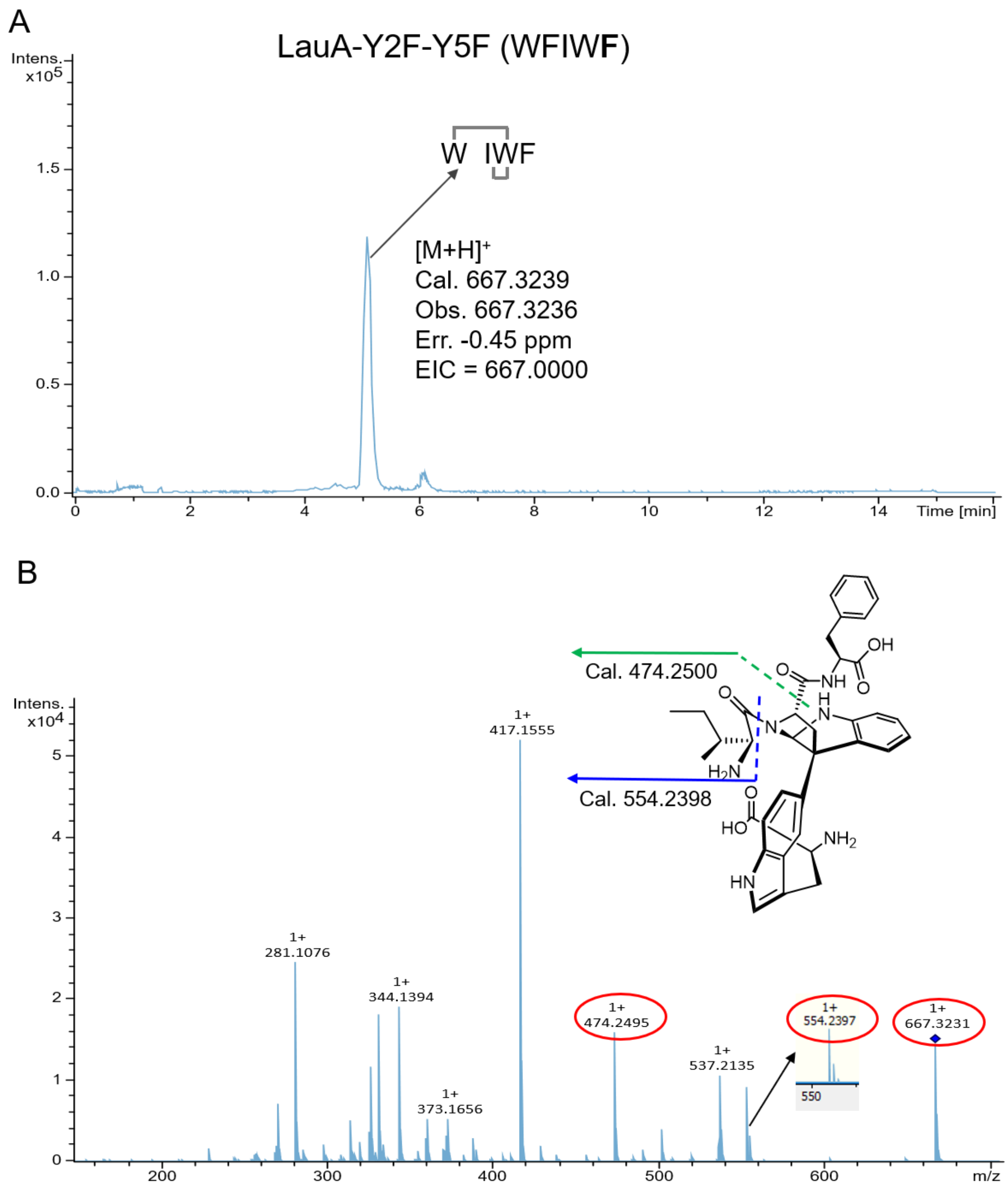

Figure S94. HRMS analysis of extracts of the recombinant *S. albus* strain harboring *lauA*-Y2F-Y5F and *lauB1*. A) Extracted ion chromatogram of the product from the coexpression of *lauA*-Y2F-Y5F with *lauB1*; B) MS/MS spectrum of the corresponding atropopeptide detected at *m*/*z* 667.3236 [M+H]+ with key fragments indicating the loss of phenylalanine at the second position of the core peptide highlighted.

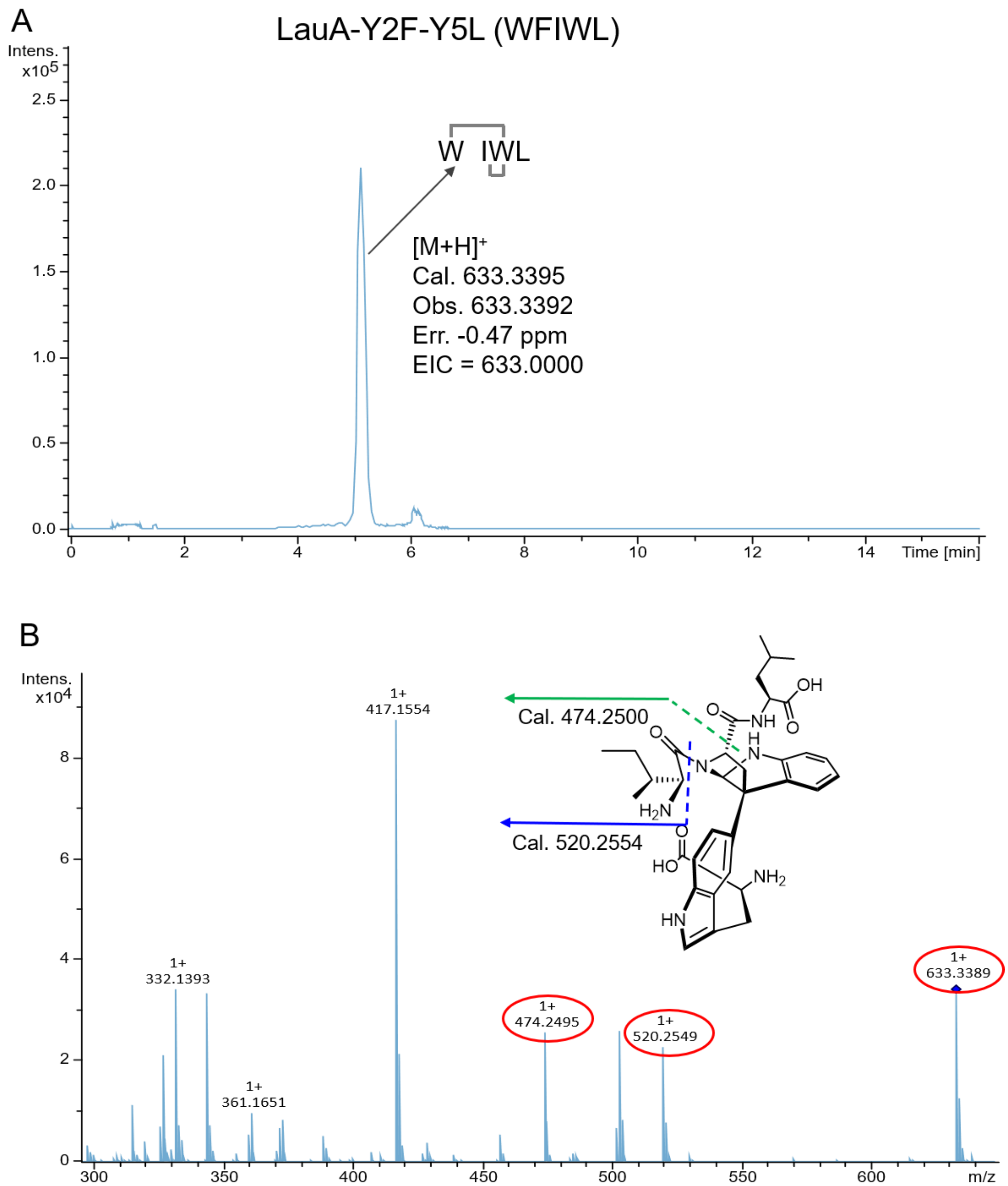

Figure S95. HRMS analysis of extracts of the recombinant *S. albus* strain harboring *lauA*-Y2F-Y5L and *lauB1*. A) Extracted ion chromatogram of the product from the coexpression of *lauA*-Y2F-Y5L with *lauB1*; B) MS/MS spectrum of the corresponding atropopeptide detected at *m*/*z* 633.3392 $[M+H]^+$ with key fragments indicating the loss of phenylalanine at the second position of the core peptide highlighted.

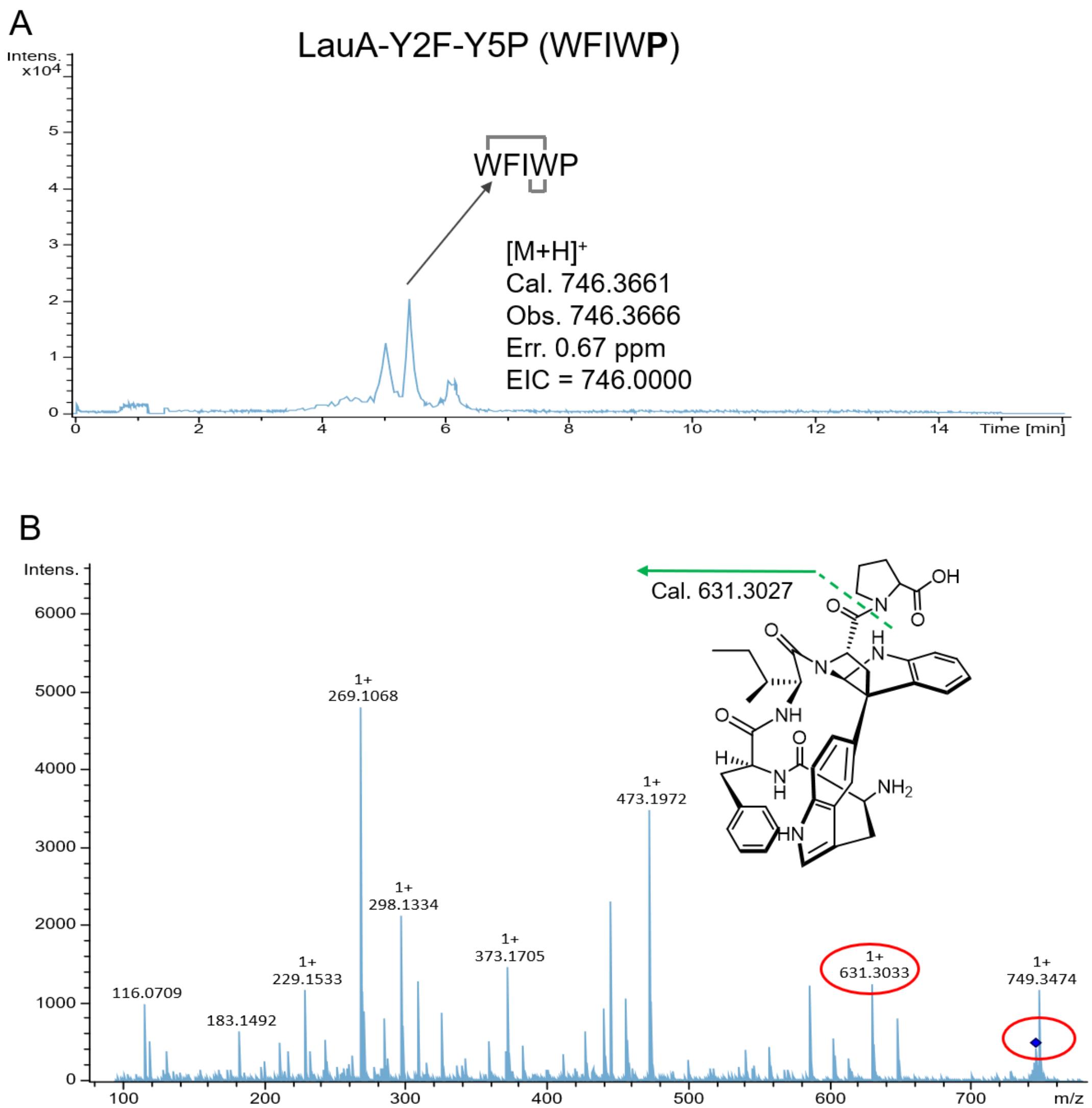

Figure S96. HRMS analysis of extracts of the recombinant *S. albus* strain harboring *lauA*-Y2F-Y5P and *lauB1*. A) Extracted ion chromatogram of the product from the coexpression of *lauA*-Y2F-Y5P with *lauB1*; B) MS/MS spectrum of the corresponding atropopeptide detected at *m*/*z* 746.3666 $[M+H]^+$ with key fragments that indicate the second residue was present highlighted.

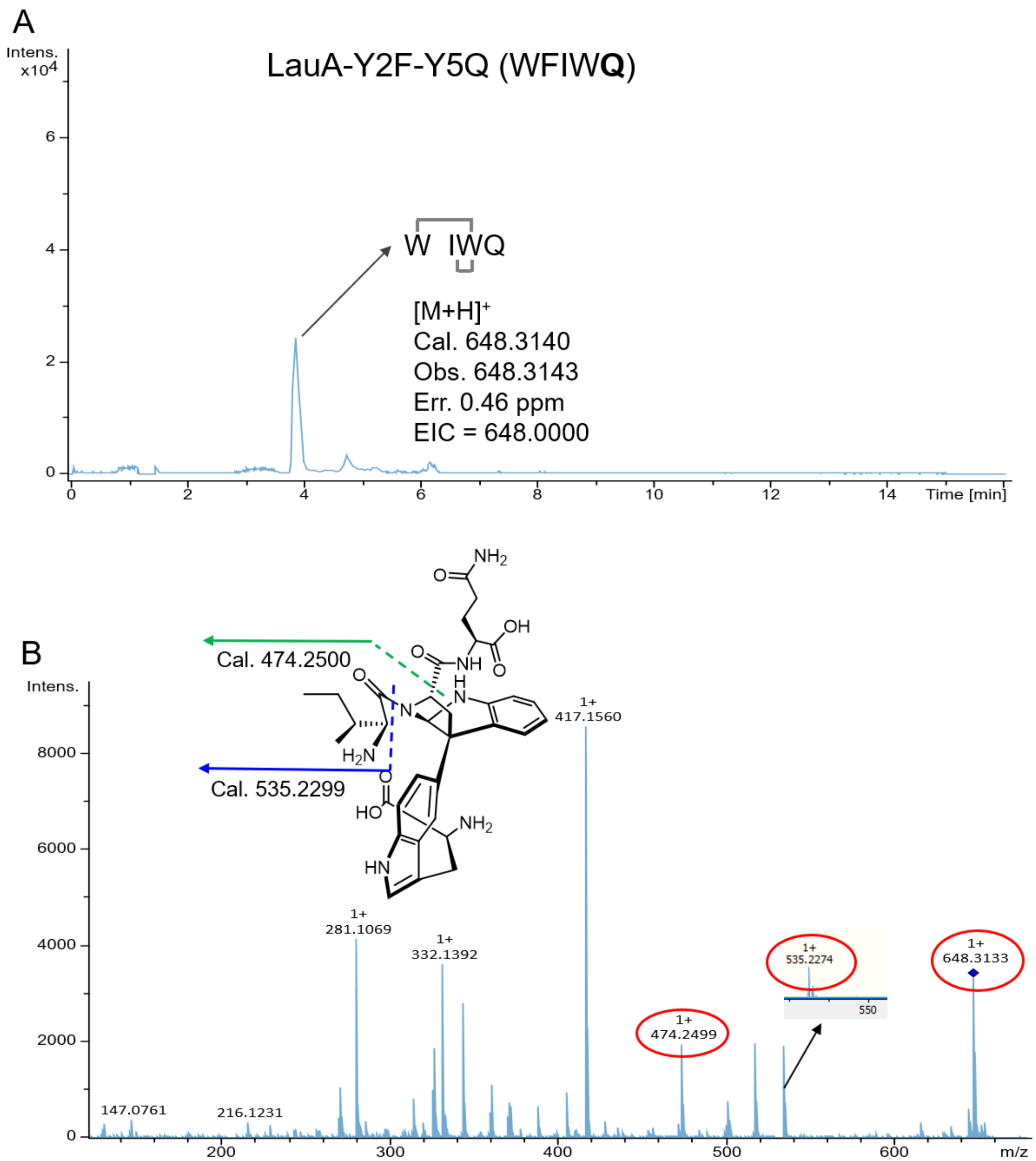

Figure S97. HRMS analysis of extracts of the recombinant *S. albus* strain harboring *lauA*-Y2F-Y5Q and *lauB1*. A) Extracted ion chromatogram of the product from the coexpression of *lauA*-Y2F-Y5Q with *lauB1*; B) MS/MS spectrum of the corresponding atropopeptide detected at *m*/*z* 648.3143 $[M+H]^+$ with key fragments indicating the loss of phenylalanine at the second position of the core peptide highlighted.

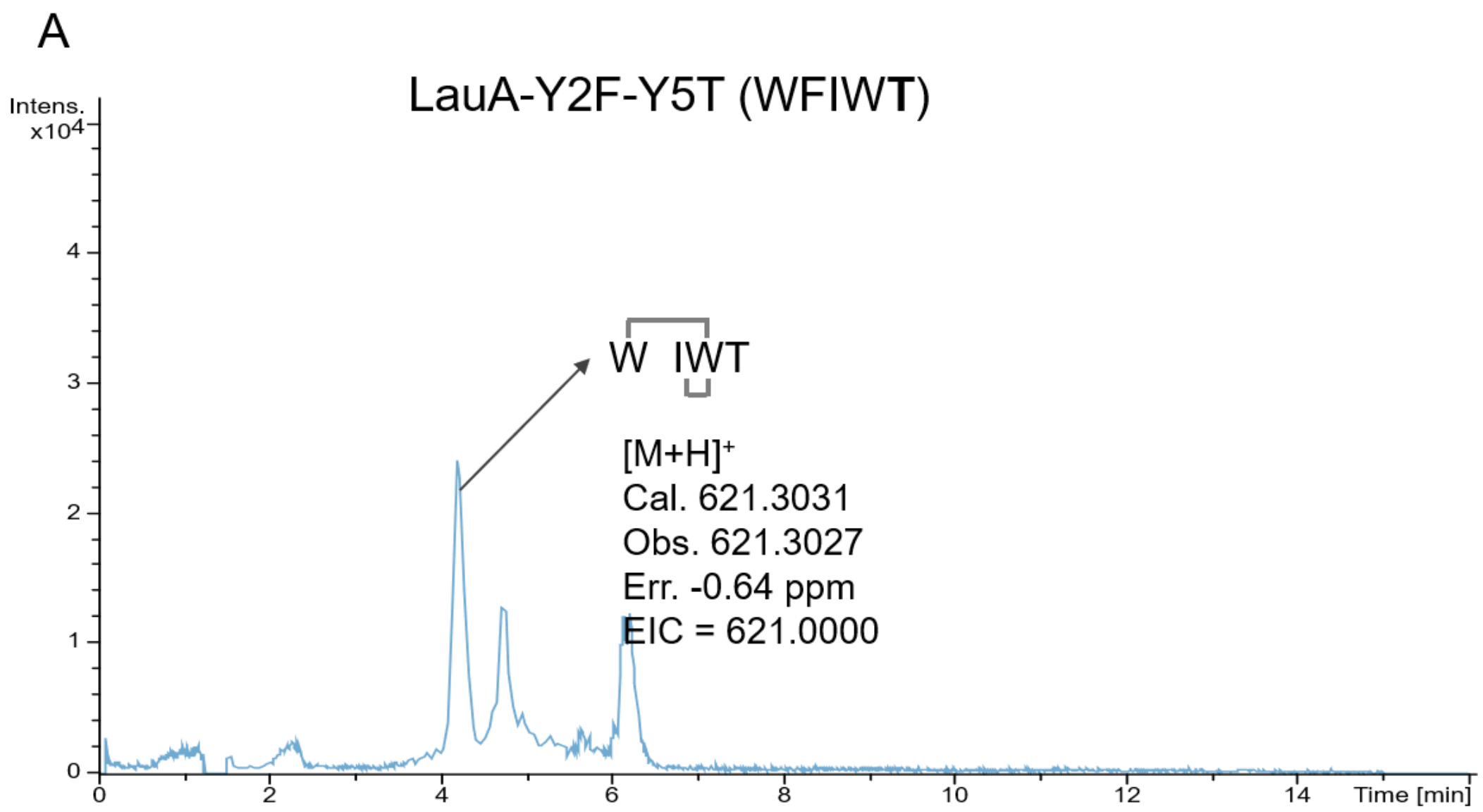

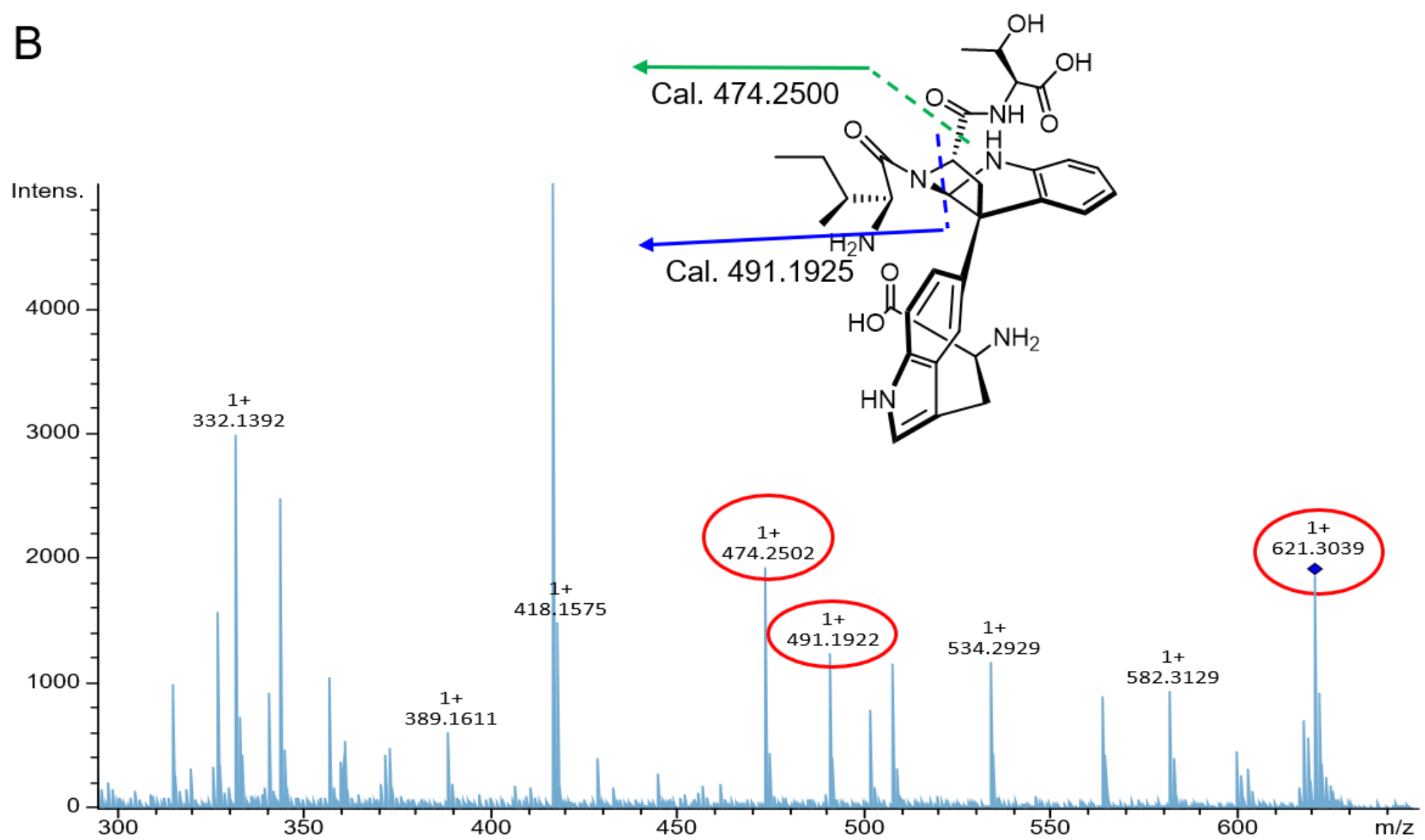

Figure S98. HRMS analysis of extracts of the recombinant *S. albus* strain harboring *lauA*-Y2F-Y5T and *lauB1*. A) Extracted ion chromatogram of the product from the coexpression of *lauA*-Y2F-Y5T with *lauB1*; B) MS/MS spectrum of the corresponding atropopeptide detected at *m*/*z* 621.3027 $[M+H]^+$ with key fragments indicating the loss of phenylalanine at the second position of the core peptide highlighted.

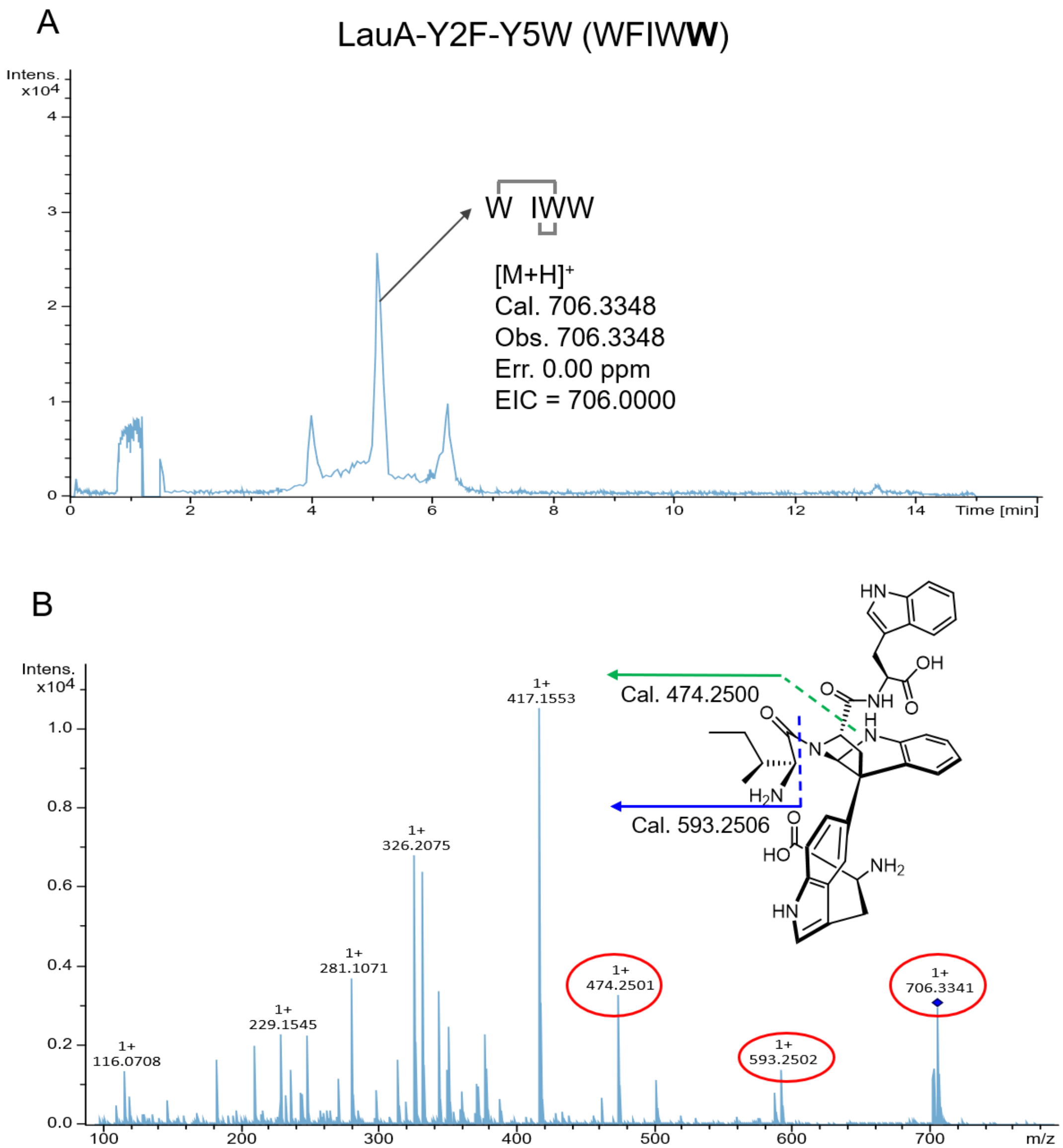

Figure S99. HRMS analysis of extracts of the recombinant *S. albus* strain harboring *lauA*-Y2F-Y5W and *lauB1*. A) Extracted ion chromatogram of the product from the coexpression of *lauA*-Y2F-Y5W with *lauB1*; B) MS/MS spectrum of the corresponding atropopeptide detected at *m*/*z* 706.3348 [M+H]+ with key fragments indicating the loss of phenylalanine at the second position of the core peptide highlighted.

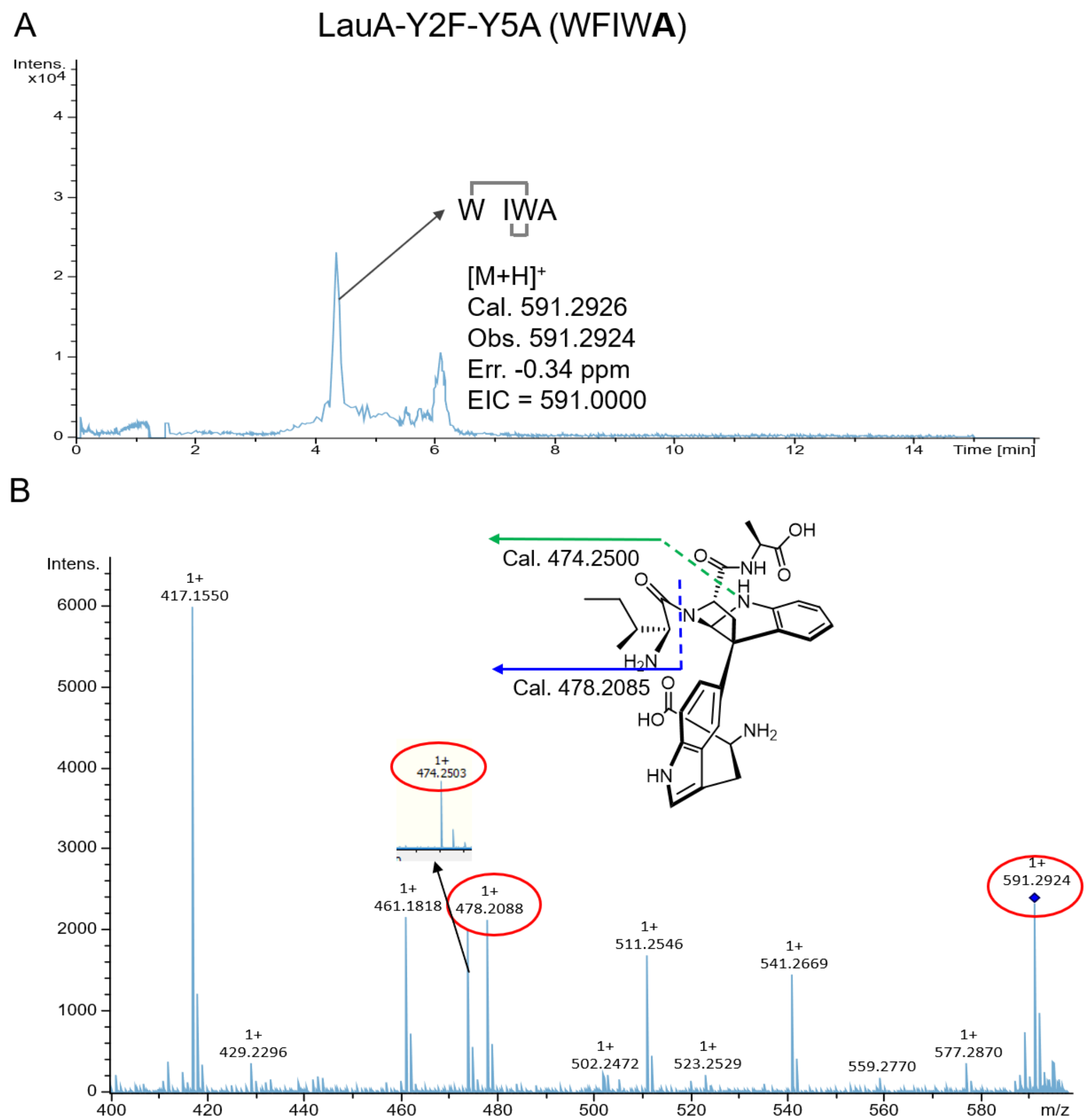

Figure S100. HRMS analysis of extracts of the recombinant *S. albus* strain harboring *lauA*-Y2F-Y5A and *lauB1*. A) Extracted ion chromatogram of the product from the coexpression of *lauA*-Y2F-Y5A with *lauB1*; B) MS/MS spectrum of the corresponding atropopeptide detected at *m*/*z* 591.2924 $[M+H]^+$ with key fragments indicating the loss of phenylalanine at the second position of the core peptide highlighted.

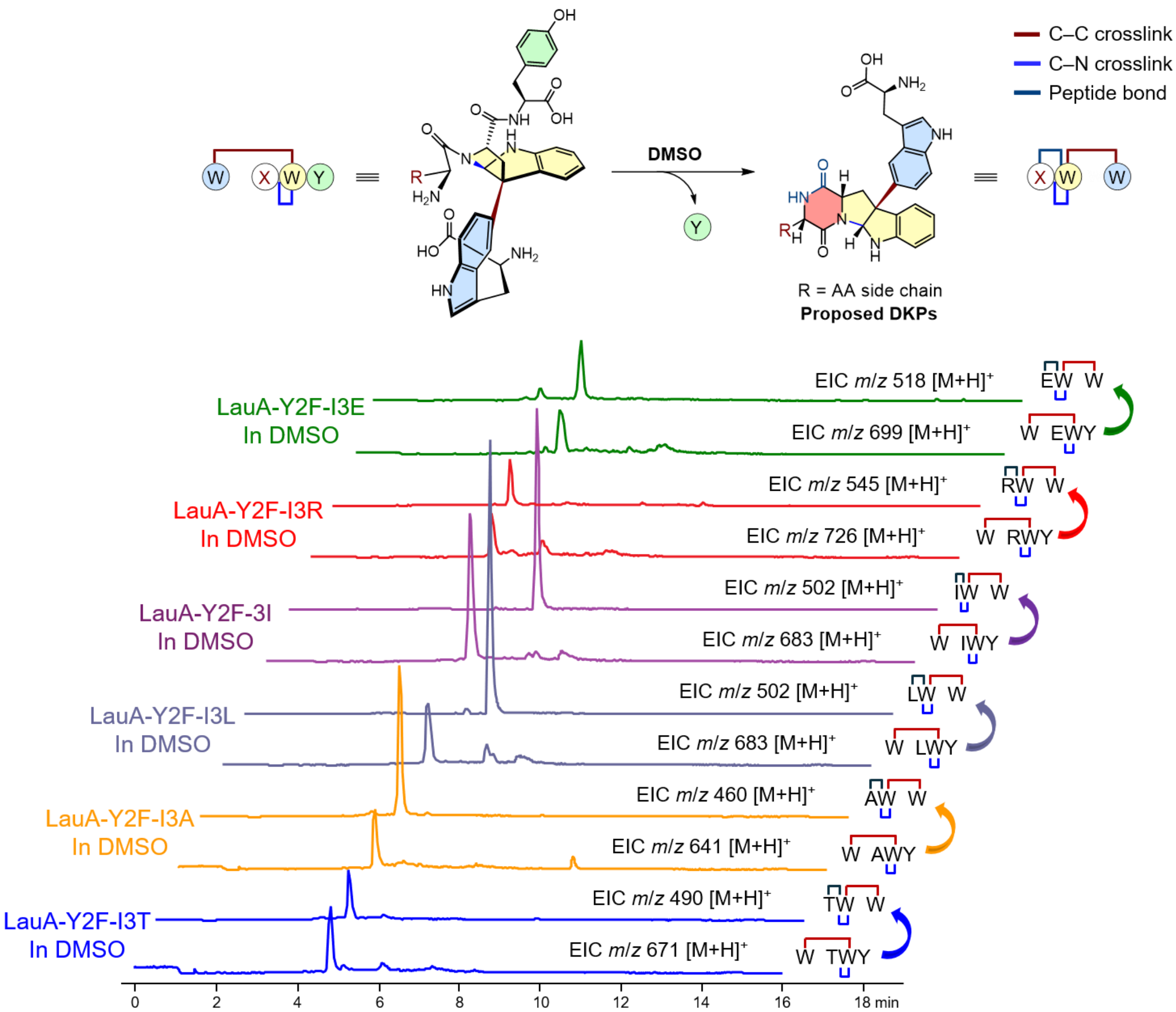

Figure S101. Investigation of DKP formation from crude extracts in DMSO. Crude extracts from LauA-Y2F-I3X mutant series were resuspended in DMSO without the addition of acid or base at room temperature. Extracted ion chromatograms of the remaining parent atropopeptide and the corresponding transformed diketopiperazine are shown.

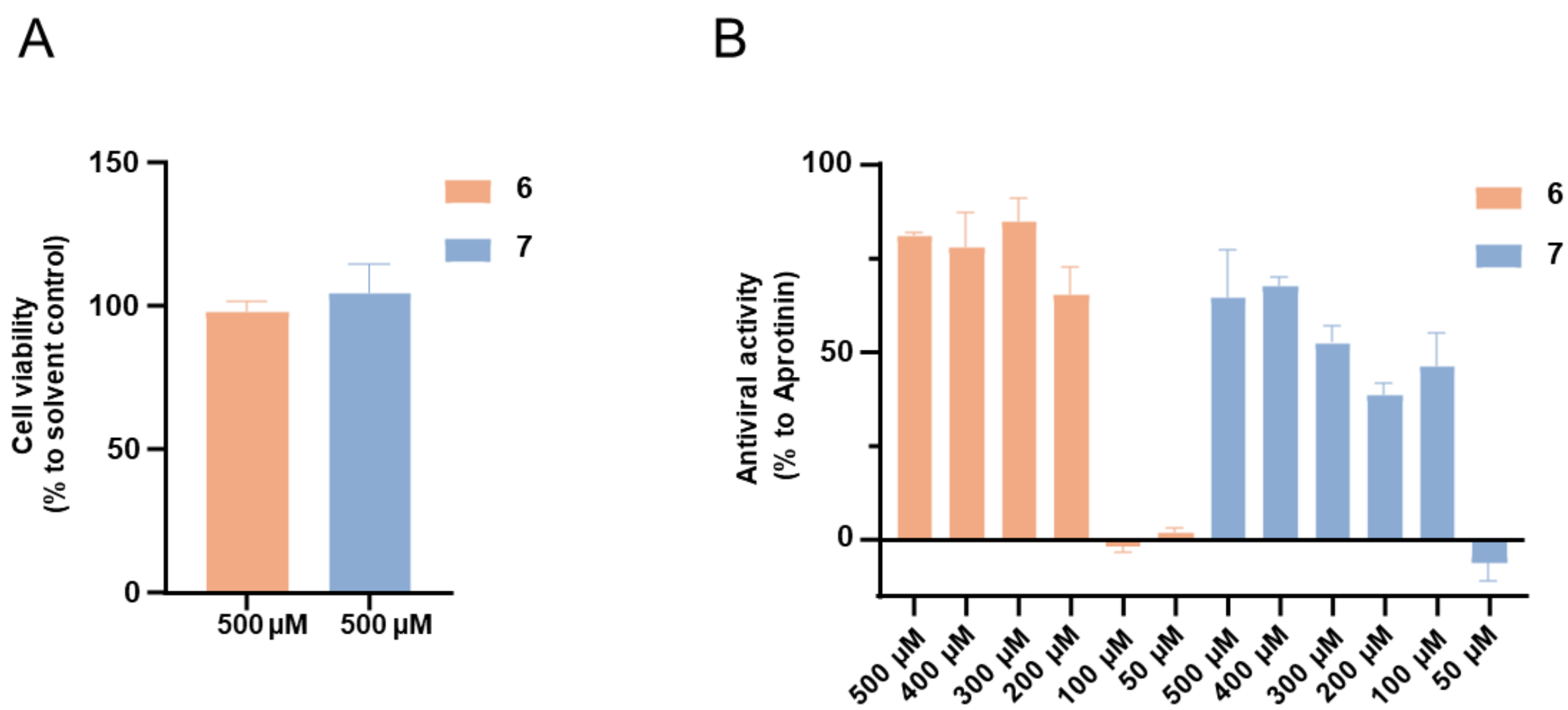

Figure S102. Antiviral activity of **6** and **7**. A) Effect of **6** and **7** on the viability of MDCK II cells. Cell viability was evaluated by measuring the intracellular ATP levels using CellTiter-Glo assay after incubation of MDCK II cells with compounds for 48 h. Cell viability was normalized to the corresponding solvent control, which was set to 100%. B) Antiviral effects of **6** and **7** against influenza virus H1N1pdm. MDCK II cells were infected with A/Hamburg/05/2009 (H1N1pdm). Aprotinin was used as positive control, which was normalized to 100%.

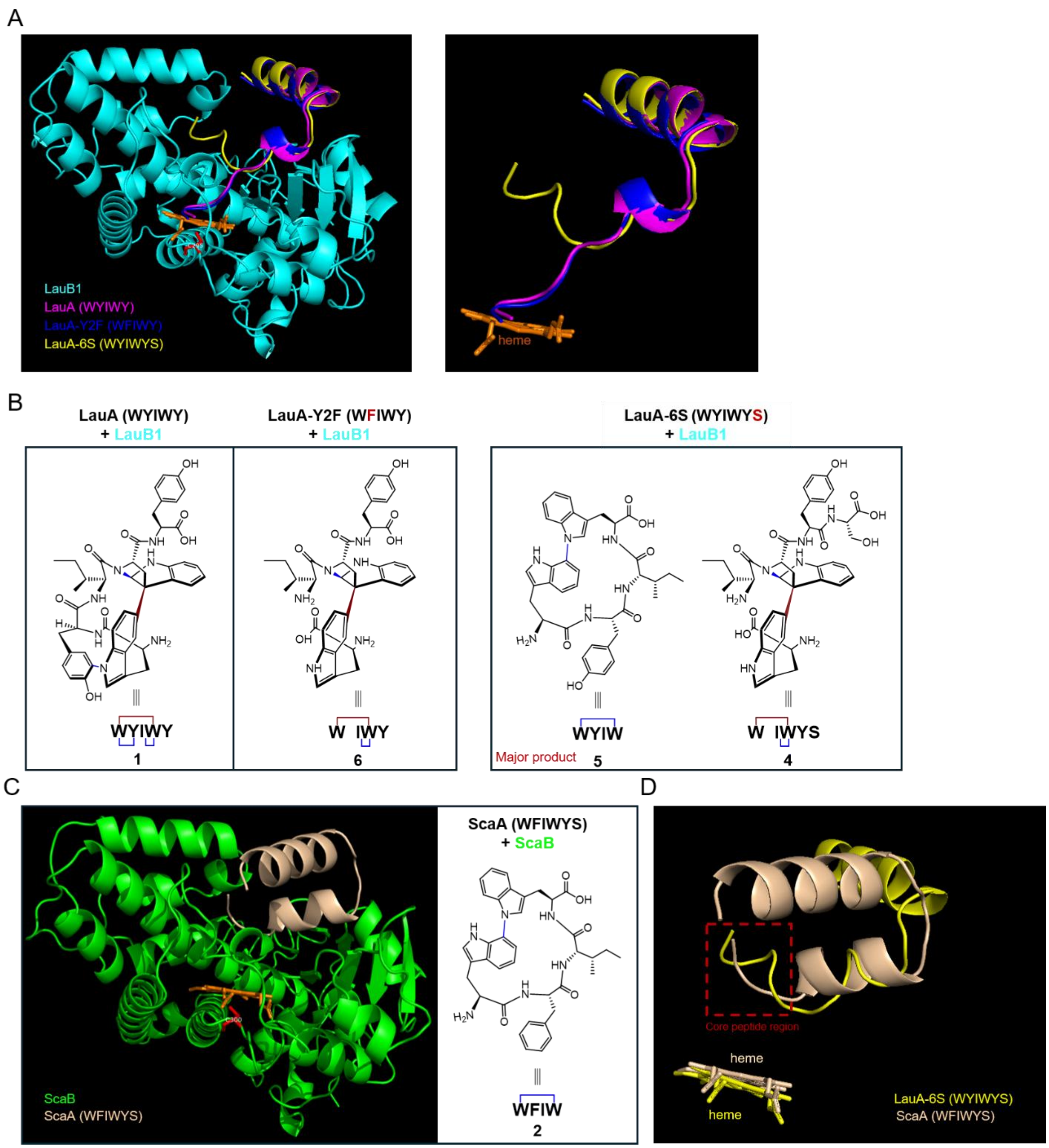

Figure S103. Predicted structural models of the LauB1-LauA, LauB1-LauA-Y2F, LauB1-LauA-6S and ScaB-ScaA complexes generated by AlphaFold2. (A) Models of LauB1 with different precursor peptides, and structure alignments of the three modelled precursors; (B) Combination of LauB1 with different precursors, and their corresponding products; (C) Model of ScaB-ScaA complex, and the corresponding product; (D) Structure alignment of LauA-6S with ScaA.

Table S1. ^{1}H (δ_H, 600 MHz) and ^{13}C NMR (δ_C, 125 MHz) NMR data for **3** in DMSO-d_6

position	δ_H, (mult, J in Hz)	δ_C, type
1		171.4, C
2	3.71, br	53.5, CH
2-NH	7.39, s	
3	3.23, ovlp.[i]	61.5, CH_2
	3.53, dd (5.0, 9.5)	
4		169.3, C
5	4.13, dd (7.5, 14.5)	53.6, CH
5-NH	7.44, d (7.5)	
6	2.27, dd (7.0, 14.5)	36.3, CH_2
	2.43, dd (7.0, 14.5)	
7		127.0, C
8/8’	6.82, d (8.5)	129.4, CH
9/9’	6.53, d (8.5)	114.3, CH
10		155.0, C
11		169.3, C
12	4.57, dd (6.5, 8.5)	60.7, CH
13	2.54, ovlp.[ii]	43.6, CH_2
	2.81, dd (8.5, 13.0)	
14		60.5, C
15	5.98, d (3.0)	84.4, CH
15-NH	6.93, d (3.0)	
16		133.1, C
17	7.23, ovlp.[iii]	124.5, CH
18	6.72, t ovlp.[iv]	117.5, CH
19	7.08, t (7.5)	127.7, CH
20	6.71, d ovlp.[iv]	109.2, CH
21		148.5, C
22		169.6, C
23	4.31, dd (7.0, 8.0)	55.0, CH
23-NH	8.17, d (7.0)	
24	1.89, m	35.9, CH
25	1.02, m	23.6, CH_2
	1.53, m	
26	0.87, t (7.5)	10.8, CH_3
27	0.91, d (6.5)	14.8, CH_3
28		169.9, C
29	4.46, m	53.7, CH
29-NH	8.25, d (9.0)	
30	2.71, dd (10.0, 14.0)	37.9, CH_2
	3.00, dd (5.0, 14.0)	
31		136.7, C
32/32’	7.17, d (7.5)	128.5, CH
33/33’	7.22, ovlp.[iii]	127.5, CH
34	7.16, t (9.0)	125.8, CH
35		169.3, C
36	4.49, m	51.1, CH

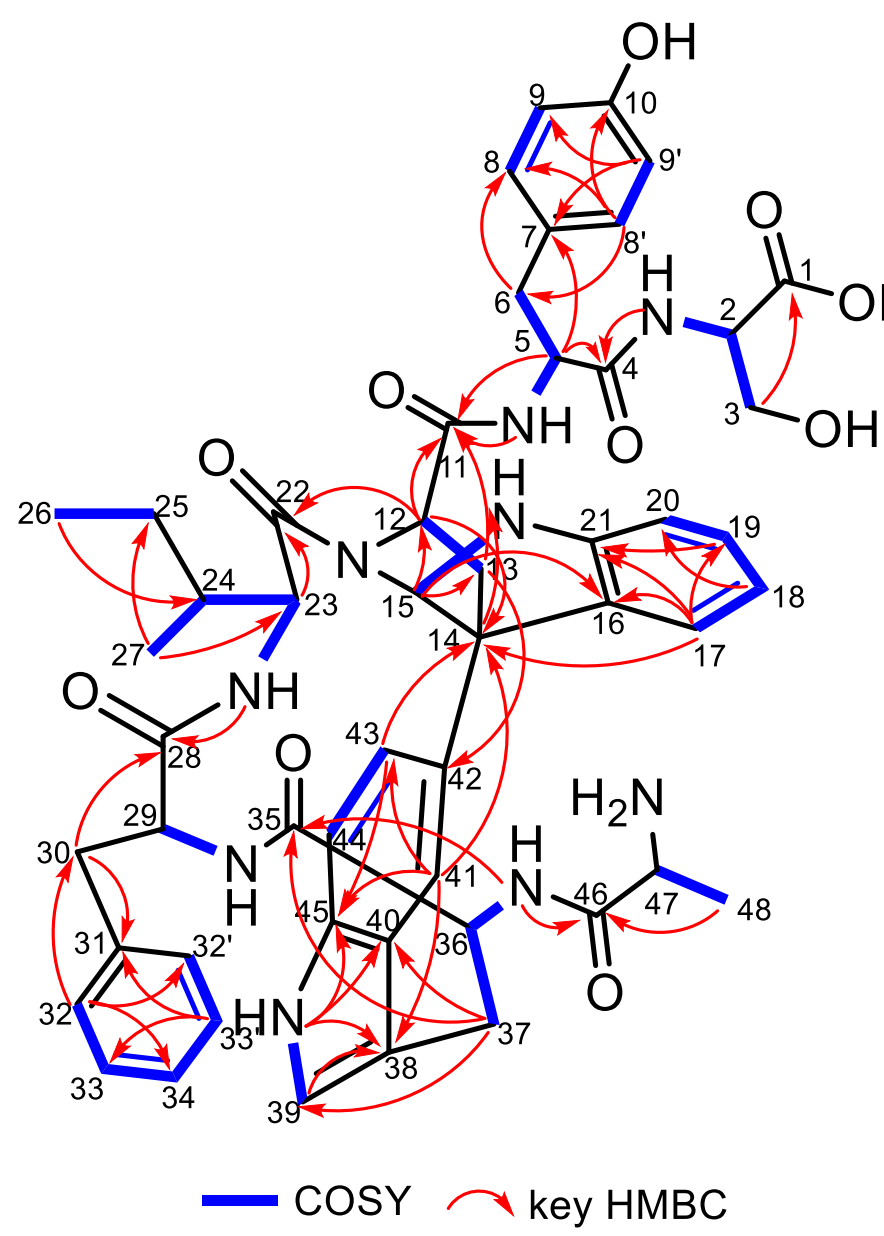

3

36-NH	8.02, d (7.5)	
37	3.14, m ovlp.[v]	26.7, CH_2
	3.18, m ovlp.[v]	
38		107.8, C
39	7.06, br s	124.6, CH
39-NH	10.86, s	
40		127.9, C
41	7.42, s	114.7, CH
42		131.5, C
43	6.91, d (8.0)	119.4, CH
44	7.21, d (8.0)	109.8, CH
45		134.1, C
46		173.0, C
47	3.43, m	49.2, CH
48	1.22, d (6.5)	20.2, CH_3

i: overlapped with water
ii: overlapped with solvent
iii: overlapped with H-17 and H-33/33'
iv: overlapped with H-18 and H-20
v: overlapped with H-37

Table S2. ^{1}H (δ_H, 600 MHz) and ^{13}C NMR (δ_C, 125 MHz) NMR data for **4** in D_2O

position	δ_H, (mult, *J* in Hz)	δ_C, type
1		178.5, C
2	4.25, m	60.1, CH
3	3.91, m	65.0, CH_2
4		174.7, C
5	4.28, m	57.8, CH
6	3.04, dd (5.4, 13.8)	40.0, CH_2
	2.65, dd (10.2, 13.8)	
7		131.3, C
8/8'	7.11, d (8.4)	133.5, CH
9/9'	6.82, d (8.4)	118.2, CH
10		157.1, C
11		173.4, C
12	4.73, t (4.8)	63.9, CH
13	3.27, ovlp.[i]	43.9, CH_2
14		61.5, C
15	5.84, s	87.1, CH
16		135.4, C
17	7.14, d (8.4)	127.5, CH
18	6.75, ovlp.[ii]	123.1, CH
19	7.15, t (8.4)	132.1, CH
20	6.75, ovlp.[ii]	113.6, CH
21		151.3, C
22		172.5, C
23	3.19, d (5.4)	59.3, CH
24	1.93, m	39.0, CH
25	1.51, m	26.6, CH_2
	1.15, m	
26	0.91, t (7.2)	13.5, CH_3
27	0.99, d (6.6)	17.1, CH_3
28		177.2, C
29	4.05, dd (4.8, 7.8)	58.0, CH
30	3.44, dd (4.8, 15.6)	29.0, CH_2
	3.27, ovlp.[i]	
31		110.6, C
32	7.302, s	128.6, CH
33		129.6, C
34	7.70, s	117.3, CH
35		136.7, C
36	7.21, d (8.4)	122.5, CH
37	7.44, d (8.4)	115.3, CH
38		138.2, C

i: overlapped with H-13 and H-30
ii: overlapped with H-18 and H-20

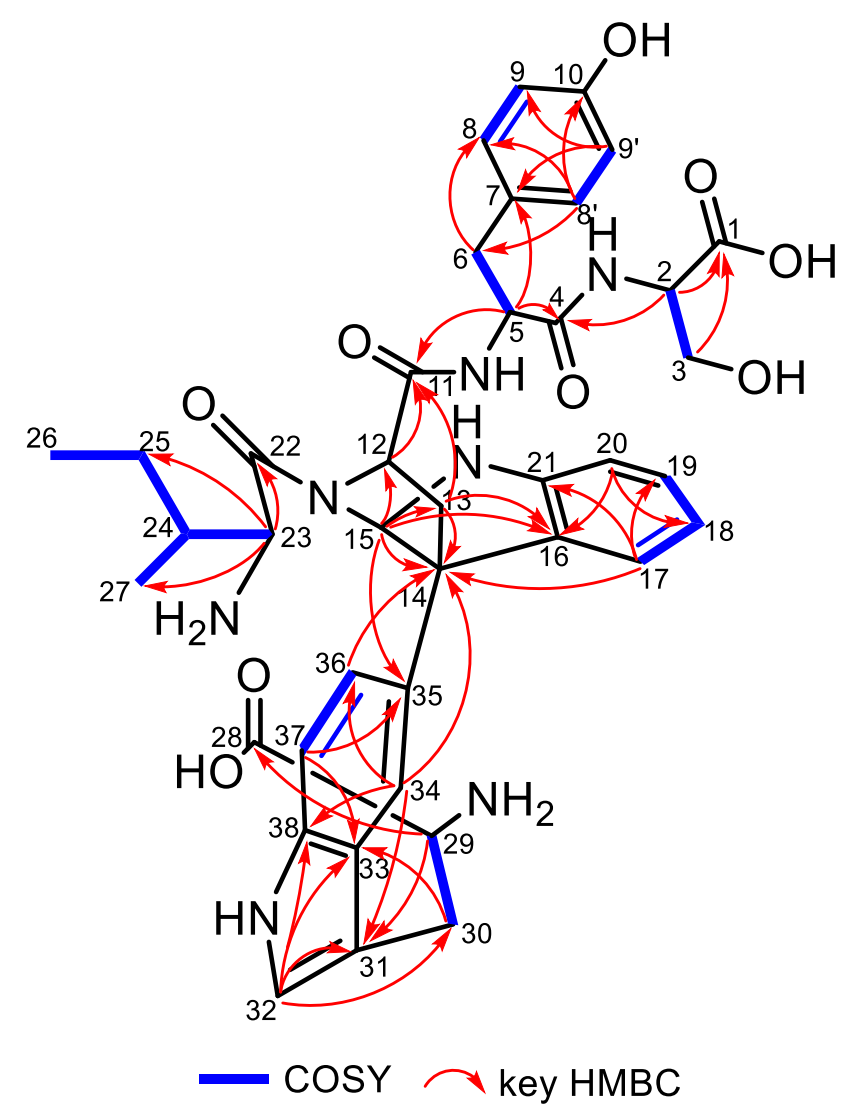

4

Table S3. ^1H (δ_H, 600 MHz) and ^{13}C NMR (δ_C, 125 MHz) NMR data for **6** in MeOD

position	δ_H, (mult, *J* in Hz)	δ_C, type
1		178.3, C
2	4.32, m	57.5, CH
3	3.13, ovlp.[i]	39.5, CH_2
	2.49, dd (10.8, 13.8)	
4		130.4, C
5/5'	6.78, d (8.4)	131.4, CH
6/6'	6.34, d (8.4)	116.1, CH
7		156.9, C
8		173.3, C
9	4.82, ovlp.[ii]	65.0, CH
10	3.40, dd (9.0, 13.8)	43.8, CH_2
	2.41, dd (6.6, 13.8)	
11		61.3, C
12	5.98, s	89.7, CH
13		136.5, C
14	6.92, d (7.8)	125.4, CH
15	6.82, t (7.8)	121.5, CH
16	7.20, t (7.8)	129.9, CH
17	6.88, d (7.8)	112.4, CH
18		149.5, C
19		171.2, C
20	3.60, d (7.2)	57.1, CH
21	1.82, m	38.4, CH
22	1.55, m	25.6, CH_2
	1.09, m	
23	0.86, d (7.8)	11.4, CH_3
24	0.84, d (7.8)	14.8, CH_3
25		174.4, C
26	3.87, m	56.7, CH
27	3.48, br d (15.0)	28.3, CH_2
	3.12, ovlp.[i]	
28		109.4, C
29	7.22, s	126.3, CH
30		128.6, C
31	7.71, s	116.9, CH
32		134.6, C
33	7.06, d (8.4)	122.4, CH
34	7.34, d (8.4)	113.1, CH
35		137.6, C

i: overlapped with H-3 and H-27
ii: overlapped with water

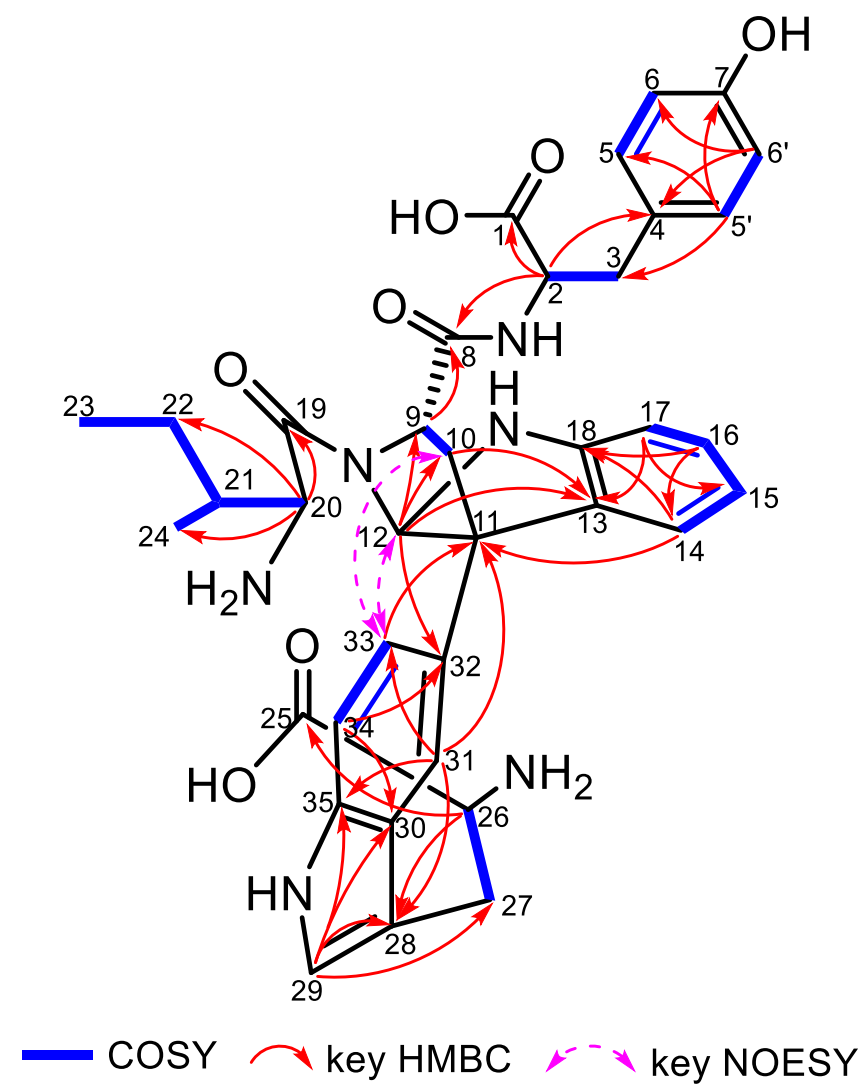

6

Table S4. ^{1}H (δ_H, 600 MHz) and ^{13}C NMR (δ_C, 150 MHz) NMR data for **7** in DMSO-d_6

position	δ_H, (mult, *J* in Hz)	δ_C, type
1		169.8, C
2	3.42, dd (4.2, 7.8)	54.9, CH
3	3.11, dd (7.8, 15.0)	34.4 CH_2
	3.25, dd (4.2, 15.0)	
4		127.1, C
5	7.21, d (2.4)	124.9, CH
5-NH	10.90, s	
6		109.9, C
7	7.77, s	116.1, CH
8		132.7, C
9	6.86, dd (1.8, 8.4)	121.1, CH
10	7.29, d (8.4)	111.6, CH
11		135.0, C
12		170.8, C
12-NH	8.02, s	
13	4.87, dd (7.2, 10.2)	58.1, CH
14	2.30, dd (10.2, 13.2)	38.6, CH_2
	3.17, dd (7.2, 13.2)	
15		59.2, C
16	5.62, d (3.6)	85.2, CH
16-NH	6.73, d (3.6)	
17		135.0, C
18	6.79, d (7.2)	123.3, CH
19	6.58, td (7.2, 0.6)	117.8, CH
20	6.99, td (7.8, 1.2)	127.7, CH
21	6.60, d (7.2)	109.1, CH
22		148.3, C
23		167.5, C
24	4.05, br s	59.2, CH
25	2.02, m	34.4, CH
26	1.30, m	23.8, CH_2
	1.39, m	
27	0.84, d (7.2)	12.3, CH_3
28	1.01, t (7.8)	14.9, CH_3

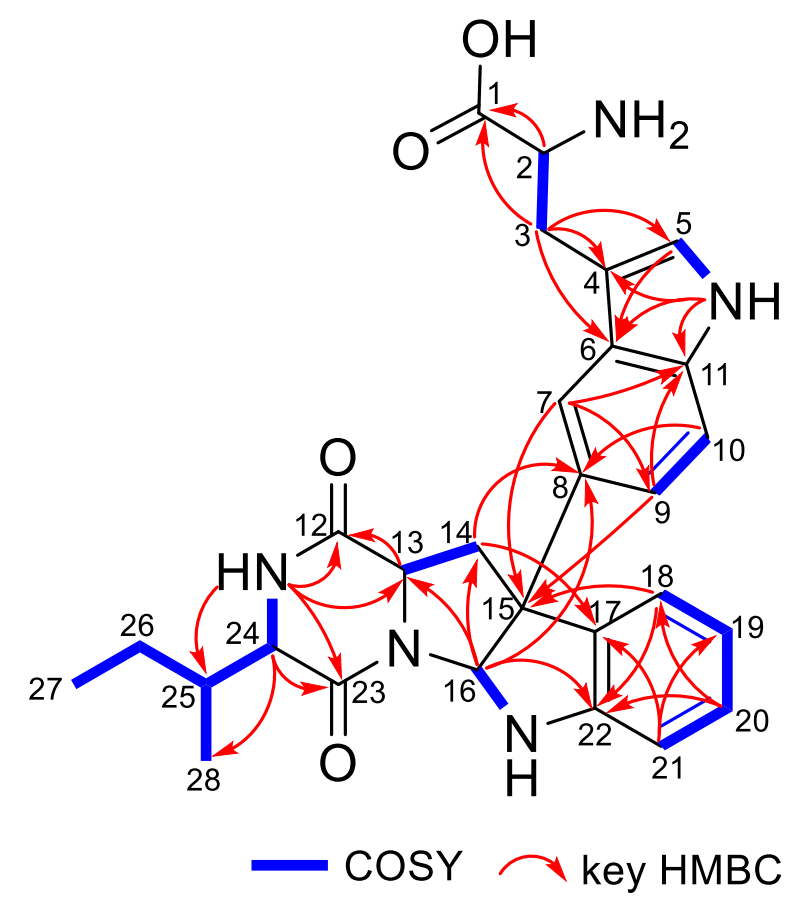

7

Table S5. ^1H (δ_H, 600 MHz) and ^{13}C NMR (δ_C, 125 MHz) NMR data for **9** in D_2O

position	δ_H, (mult, J in Hz)	δ_C, type
1		180.1, C
2	3.89, dd (6.0, 8.4)	59.2, CH
3	2.85, dd (6.0, 13.8)	40.3, CH_2
	2.56, dd (8.4, 13.8)	
4		132.3, C
5/5’	7.04, d (8.4)	133.5, CH
6/6’	6.79, ovlp.[i]	118.0, CH
7		156.9, C
8		172.8, C
9	4.17, dd (1.2, 8.4)	64.0, CH
10	2.96, dd (1.2,13.8)	43.5, CH_2
	2.68, dd (8.4, 13.8)	
11		61.7, C
12	5.74, s	87.1, CH
13		135.4, C
14	7.08, dd (0.6, 7.8)	127.7, CH
15	6.81, ovlp.[i]	123.3, CH
16	7.18, td (7.8, 1.2)	132.1, CH
17	6.80, ovlp.[i]	113.8, CH
18		151.2, C
19		175.0, C
20	3.68, m	54.8, CH
21	3.13, dd (5.4, 13.8)	33.0, CH_2
	3.02, dd (9.6, 13.8)	
22		134.5, C
23	7.71, br s	139.1, CH
24	7.05, s	119.3, CH
25		177.3, C
26	4.07, dd (4.8, 7.8)	57.9, CH
27	3.46, dd (4.8, 15.0)	29.0, CH_2
	3.34, dd (7.8, 15.0)	
28		110.6, C
29	7.35, s	128.7, CH
30		129.5, C
31	7.64, d (1.8)	117.5, CH
32		136.5, C
33	7.16, dd (1.8, 9.0)	122.8, CH
34	7.50, d (9.0)	115.3, CH
35		138.2, C

i: overlapped with H-6/6’, H-15, H-17

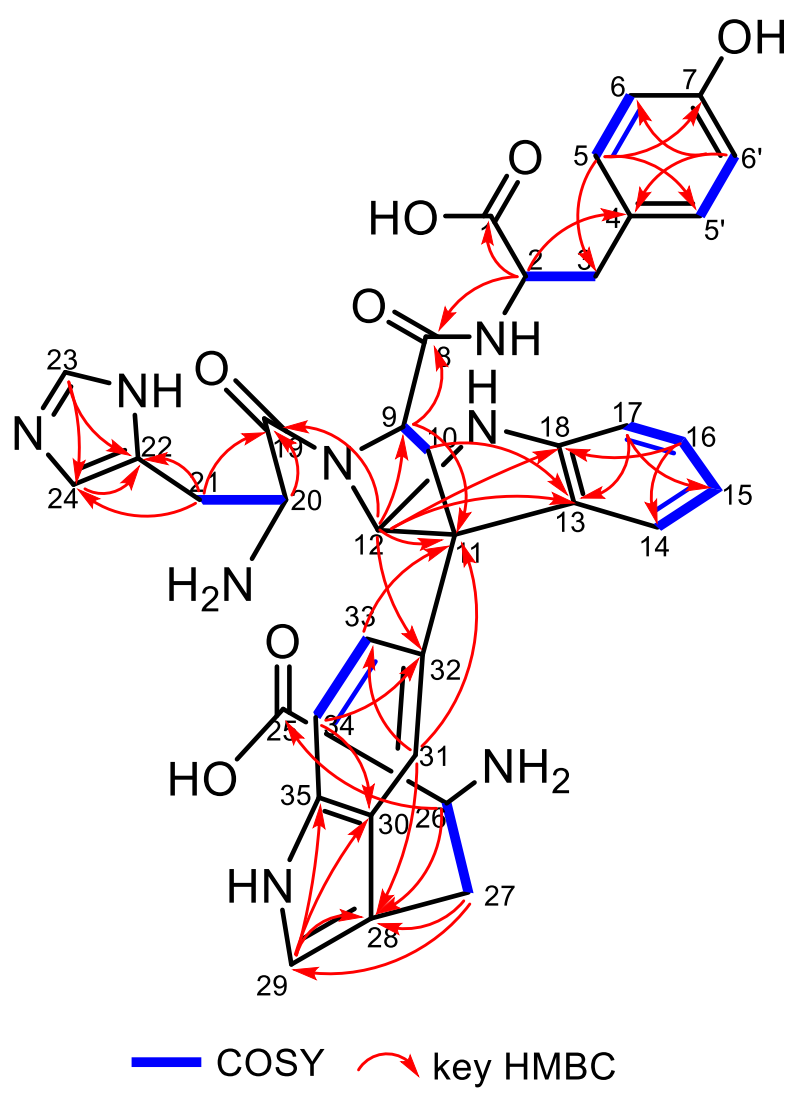

9

Table S6. Strains and plasmids used in this study

Strains/plasmids	Characteristic(s)	Sources
E. coli		
DH5a	Host strain for cloning	[2]
ET12567/pUZ8002	Donor strain for conjugation	[3]
Xanthomonas		
Xanthomonas translucens pv. translucens DSM 18974	Wild type strain containing *xat*	DSMZ
Xanthomonas sp. Leaf148	Wild type strain containing *xal* BGC	[4]
Xanthomonas sp. Leaf148/Δ*xal*	*Xanthomonas* sp. leaf 148 derivative where the atropopeptide BGC *xal* has been deleted	This study
Xanthomonas sp. Leaf148/Δ*xal/xat*	*Xanthomonas* sp. Leaf148/Δ*xal* derivative where *xat* BGC was introduced	This study
Streptomyces		
S. albus J1074	Heterologous host	[5]
Streptomyces kanamyceticus DSM 40500	Wild type strain containing *ska* BGC	DSMZ
S. albus J1074/*$lauA_L$-$scaA_C$ +lauB1*	*S.albus* J1074 harboring *$lauA_L$-$scaA_C$* and *lauB1*	This study
S. albus J1074/*lauA*-6S+*lauB1*	*S.albus* J1074 harboring *lauA-6S* and *lauB1*	This study
S. albus J1074/*lauA-Y2F+lauB1*	*S.albus* J1074 harboring *lauA-Y2F* and *lauB1*	This study
S. albus J1074/*lauA-6X+lauB1* *(X =A/C/E/G/H/N/R/S/T/V/Y)*	*S.albus* J1074 harboring *lauA-6X* and *lauB1*	This study
S. albus J1074/*lauA-Y2F-I3X+lauB1* *(X = A/C/E/H/L/P/Q/R/T)*	*S.albus* J1074 harboring *lauA-Y2F-I3X* and *lauB1*	This study
S. albus J1074/*lauA-Y2X+lauB1* *(X = A/E/H/P/R/T/V/W)*	*S.albus* J1074 harboring *lauA-Y2X* and *lauB1*	This study
S. albus J1074/*lauA-Y2F-Y5X+lauB1* *(X = A/C/E/F/H/L/P/Q/R/T/W)*	*S.albus* J1074 harboring *lauA-Y2F-Y5X* and *lauB1*	This study
S. albus J1074/lauB1	*S.albus* J1074 harboring *lauB1*	This study
S. albus J1074/*ska*	*S.albus* J1074 harboring *ska* BGC	This study
**Plasmids**		
pUWL201-OriT	Apr^r, *ermE**p, replicative expression vector in *Streptomyces*	[6]
pME6012	Tet^r, *Kan**p, pVS1 replicon.	[7]
pME6012-*xat*	pME6012 derivative containing *xat* BGC	This study
pUWL201-OriT-*lauA+lauB1*	pUWL201 derivative containing *lauA* and *lauB1*	[8]

pUWL201-OriT-His	pUWL201 derivative with 7 his tag	This study
pUWL201-OriT-His-*lauB1*	pUWL201-OriT-His derivative containing *lauB1*	This study
pK18mobsacB	Gene knockout vector, *sacB*, Km^r, *ori*T	[9]
pL148_xal_KO	pK18mobsacB derivative used for deletion of *xal* BGC	This study
pUWL201-OriT-*ska*	pUWL201 derivative containing *ska* BGC	This study
pME6012-*xat*	pME6012 derivative containing *xat* BGC	This study
pUWL201-OriT- *lauA*$_L$*-scaA*$_C$*+lauB1*	pUWL201 derivative containing *lauA*$_L$*-scaA*$_C$ and *lauB1*	This study
pUWL201-OriT- *lauA-6S+lauB1*	pUWL201 derivative containing *lauA-6S* and *lauB1*	This study
pUWL201-OriT-*lauA-Y2F+lauB1*	pUWL201 derivative containing *lauA-Y2F* and *lauB1*	This study
pUWL201-OriT-*lauA-6A+lauB1*	pUWL201 derivative containing *lauA-6A* and *lauB1*	This study
pUWL201-OriT-*lauA-6C+lauB1*	pUWL201 derivative containing *lauA-6C* and *lauB1*	This study
pUWL201-OriT-*lauA-6E+lauB1*	pUWL201 derivative containing *lauA-6E* and *lauB1*	This study
pUWL201-OriT-*lauA-6G+lauB1*	pUWL201 derivative containing *lauA-6G* and *lauB1*	This study
pUWL201-OriT-*lauA-6H+lauB1*	pUWL201 derivative containing *lauA-6H* and *lauB1*	This study
pUWL201-OriT-*lauA-6N+lauB1*	pUWL201 derivative containing *lauA-6N* and *lauB1*	This study
pUWL201-OriT-*lauA-6R+lauB1*	pUWL201 derivative containing *lauA-6R* and *lauB1*	This study
pUWL201-OriT-*lauA-6S+lauB1*	pUWL201 derivative containing *lauA-6S* and *lauB1*	This study
pUWL201-OriT-*lauA-6T+lauB1*	pUWL201 derivative containing *lauA-6T* and *lauB1*	This study
pUWL201-OriT-*lauA-6V+lauB1*	pUWL201 derivative containing *lauA-6V* and *lauB1*	This study
pUWL201-OriT-*lauA-6Y+lauB1*	pUWL201 derivative containing *lauA-6Y* and *lauB1*	This study
pUWL201-OriT-*lauA-Y2F-I3A+lauB1*	pUWL201 derivative containing *lauA-Y2F-I3A* and *lauB1*	This study
pUWL201-OriT-*lauA-Y2F-I3C+lauB1*	pUWL201 derivative containing *lauA-Y2F-I3C* and *lauB1*	This study
pUWL201-OriT-*lauA-Y2F-I3E+lauB1*	pUWL201 derivative containing *lauA-Y2F-I3E* and *lauB1*	This study
pUWL201-OriT-*lauA-Y2F-I3H+lauB1*	pUWL201 derivative containing *lauA-Y2F-I3H* and *lauB1*	This study
pUWL201-OriT-*lauA-Y2F-I3L+lauB1*	pUWL201 derivative containing *lauA-Y2F-I3L* and *lauB1*	This study
pUWL201-OriT-*lauA-Y2F-I3P+lauB1*	pUWL201 derivative containing *lauA-Y2F-I3P* and *lauB1*	This study
pUWL201-OriT-*lauA-Y2F-I3Q+lauB1*	pUWL201 derivative containing *lauA-Y2F-I3Q* and *lauB1*	This study
pUWL201-OriT-*lauA-Y2F-I3R+lauB1*	pUWL201 derivative containing *lauA-Y2F-I3R* and *lauB1*	This study
pUWL201-OriT-*lauA-Y2F-I3T+lauB1*	pUWL201 derivative containing *lauA-Y2F-I3T* and *lauB1*	This study
pUWL201-OriT-*lauA-Y2A+lauB1*	pUWL201 derivative containing *lauA-Y2A* and *lauB1*	This study
pUWL201-OriT-*lauA-Y2E+lauB1*	pUWL201 derivative containing *lauA-Y2E* and *lauB1*	This study
pUWL201-OriT-*lauA-Y2H+lauB1*	pUWL201 derivative containing *lauA-Y2H* and *lauB1*	This study
pUWL201-OriT-*lauA-Y2P+lauB1*	pUWL201 derivative containing *lauA-Y2P* and *lauB1*	This study
pUWL201-OriT-*lauA-Y2R+lauB1*	pUWL201 derivative containing *lauA-Y2R* and *lauB1*	This study
pUWL201-OriT-*lauA-Y2T+lauB1*	pUWL201 derivative containing *lauA-Y2T* and *lauB1*	This study

pUWL201-OriT-*lauA-Y2V+lauB1*	pUWL201 derivative containing *lauA-Y2V* and *lauB1*	This study
pUWL201-OriT-*lauA-Y2W+lauB1*	pUWL201 derivative containing *lauA-Y2W* and *lauB1*	This study
pUWL201-OriT-*lauA-Y2F-Y5A+lauB1*	pUWL201 derivative containing *lauA-Y2F-Y5A* and *lauB1*	This study
pUWL201-OriT-*lauA-Y2F-Y5C+lauB1*	pUWL201 derivative containing *lauA-Y2F-Y5C* and *lauB1*	This study
pUWL201-OriT-*lauA-Y2F-Y5E+lauB1*	pUWL201 derivative containing *lauA-Y2F-Y5E* and *lauB1*	This study
pUWL201-OriT-*lauA-Y2F-Y5F+lauB1*	pUWL201 derivative containing *lauA-Y2F-Y5F* and *lauB1*	This study
pUWL201-OriT-*lauA-Y2F-Y5H+lauB1*	pUWL201 derivative containing *lauA-Y2F-Y5H* and *lauB1*	This study
pUWL201-OriT-*lauA-Y2F-Y5L+lauB1*	pUWL201 derivative containing *lauA-Y2F-Y5L* and *lauB1*	This study
pUWL201-OriT-*lauA-Y2F-Y5P+lauB1*	pUWL201 derivative containing *lauA-Y2F-Y5P* and *lauB1*	This study
pUWL201-OriT-*lauA-Y2F-Y5Q+lauB1*	pUWL201 derivative containing *lauA-Y2F-Y5Q* and *lauB1*	This study
pUWL201-OriT-*lauA-Y2F-Y5R+lauB1*	pUWL201 derivative containing *lauA-Y2F-Y5R* and *lauB1*	This study
pUWL201-OriT-*lauA-Y2F-Y5T+lauB1*	pUWL201 derivative containing *lauA-Y2F-Y5T* and *lauB1*	This study
pUWL201-OriT-*lauA-Y2F-Y5W+lauB1*	pUWL201 derivative containing *lauA-Y2F-Y5W* and *lauB1*	This study

Table S7. Primers used in this study

**Primers**	**Fragment**	**Sequence (5' to 3')**
Construction of pUWL201-OriT/$lauA_L$-$scaA_C$+*lauB1*		
LL-EC1-F	LL-EC1	CTGGTTCATCTGGTACAGCTAGAACGCCTAGTCGTCCCCG
LL-EC1-R		GCTGTACCAGATGAACCAGGCACTGGCCTTCAGGCTCTTC
Construction of pUWL201-OriT/*lauA-6S*+lauB1		
LA-6S-F	LauA-6S	GCCTGGTACATCTGGTACAGCTAGAACGCCTAGTCGTCC
LA-6S-R		GCTGTACCAGATGTACCAGGCACTGGCCTTCAGGCTCTTC
Construction of pUWL201-OriT/*lauA-Y2F*+lauB1		
LL-EC2-F	LL-EC2	CTGAAGGCCAGTGCCTGGTTCATCTGGTACTAG
LL-EC2-R		GAACCAGGCACTGGCCTTCAGGCTCTTCTGCGCGGAC
Construction of pL148_xal_KO		
148_xal_upF	Up homologous arm	ATTGAATTCTCGGCGACAGCCGGGTCAAG
148_xal_upR		AGATCTAGACATGAGCGACATCCTGTTTG
148_xal_downF	Down homologous arm	ATTTCTAGAGATGTCGTGCGTTGCTAGCG
148_xal_downR		ATTAAGCTTCTATCTCGACAGTTTGCAGGCAG
For screening of the *xal* deleted mutant		
L148_xal_OF		CCGCGGTGGCTACCTTTG
L148_xal_OR		CCGCATCGCGCTGATCAAG
L148_xal_AF		CAAACTGATGCAGGCAATCG
L148_xal_AR		TGGCGACCAAGGTGGAAATG
Construction of pME6012/*xat*		
Xat-F	*xat* BGC containing	ctatcgatgcatgccatggtaccGTGACTCTCCCCGATAAGG
Xat-R		gaatggcaaaagcttcgaattcTCAGTGGCGGACGACGTCGAC
pME6012-F	pME6012 backbone	gaattcgaagcttttgcc
pME6012-R		ccatggcatgcatcgatag
Construction of pUWL201-His		
pUWL-His-F	pUWL201-His	CATCATCATCATCATCATCACAGCAGCGGCCATaagcttgatatcgaattcc
pUWL-His-R		GTGATGATGATGATGATGATGATGATGATGGCCCATatggggcctcctgttctag
Construction of pUWL201-His-lauB1		
lauB1-puwl-F	pUWL201-His-lauB1	CACAGCAGCGGCCATaagcttATGCGCGCCACGGTGTTCC
lauB1-puwl-R		gcttctagaactagtggatccTCAGACAGTGGCGGCACG
Construction of pUWL201-OriT/*ska*		
ska-F	*ska* BGC containing	gtctagaacaggaggccccatATGAAGATCGTTCGCTCG
ska-R		caggaattcgatatcaagcttGTGTTACTCACACCCCTAC
pUWL-oriT-F	pUWL backbone	aagcttgatatcgaattcc
pUWL-oriT-R		atggggcctcctgttctag
For mutation of lauA-6X		
LA-6A-F	LauA-6A	GCCTGGTACATCTGGTACgcCTAGAACGCCTAGTCGTCC
LA-6A-R		gcGTACCAGATGTACCAGGCACTGGCCTTCAGGCTCTTC
LA-6C-F	LauA-6C	GCCTGGTACATCTGGTACtGCTAGAACGCCTAGTCGTCC
LA-6C-R		aGTACCAGATGTACCAGGCACTGGCCTTCAGGCTCTTC
LA-6E-F	LauA-6E	GCCTGGTACATCTGGTACgagTAGAACGCCTAGTCGTCC
LA-6E-R		ctcGTACCAGATGTACCAGGCACTGGCCTTCAGGCTCTTC
LA-6G-F	LauA-6G	GCCTGGTACATCTGGTACgGCTAGAACGCCTAGTCGTCC
LA-6G-R		cGTACCAGATGTACCAGGCACTGGCCTTCAGGCTCTTC
LA-6H-F	LauA-6H	GCCTGGTACATCTGGTACcaCTAGAACGCCTAGTCGTCC
LA-6H-R		tgGTACCAGATGTACCAGGCACTGGCCTTCAGGCTCTTC
LA-6N-F	LauA-6N	GCCTGGTACATCTGGTACAaCTAGAACGCCTAGTCGTCC
LA-6N-R		tTGTACCAGATGTACCAGGCACTGGCCTTCAGGCTCTTC
LA-6R-F	LauA-6R	GCCTGGTACATCTGGTACAGgTAGAACGCCTAGTCGTCC
LA-6R-R		cCTGTACCAGATGTACCAGGCACTGGCCTTCAGGCTCTTC
LA-6S-F	LauA-6S	GCCTGGTACATCTGGTACAGCTAGAACGCCTAGTCGTCC
LA-6S-R		GCTGTACCAGATGTACCAGGCACTGGCCTTCAGGCTCTTC
LA-6T-F	LauA-6T	GCCTGGTACATCTGGTACAcCTAGAACGCCTAGTCGTCC

LA-6T-R		gTGTACCAGATGTACCAGGCACTGGCCTTCAGGCTCTTC
LA-6V-F	LauA-6V	GCCTGGTACATCTGGTACgtCTAGAACGCCTAGTCGTCC
LA-6V-R		acGTACCAGATGTACCAGGCACTGGCCTTCAGGCTCTTC
LA-6Y-F	LauA-6Y	GCCTGGTACATCTGGTACtaCTAGAACGCCTAGTCGTCC
LA-6Y-R		taGTACCAGATGTACCAGGCACTGGCCTTCAGGCTCTTC
For mutation of lauA-Y2F-I3X		
LA-Y2F-I3A-F	LauA-Y2F-I3A	GAAGGCCAGTGCCTGGTTCgcCTGGTACTAGAACGCC
LA-Y2F-I3A-R		gcGAACCAGGCACTGGCCTTCAGGCTCTTCTGCGCGG
LA-Y2F-I3C-F	LauA-Y2F-I3C	GAAGGCCAGTGCCTGGTTCtgCTGGTACTAGAACGC
LA-Y2F-I3C-R		caGAACCAGGCACTGGCCTTCAGGCTCTTCTGCGCGG
LA-Y2F-I3E-F	LauA-Y2F-I3E	GAAGGCCAGTGCCTGGTTCgagTGGTACTAGAACGCC
LA-Y2F-I3E-R		ctcGAACCAGGCACTGGCCTTCAGGCTCTTCTGCGCGG
LA-Y2F-I3H-F	LauA-Y2F-I3H	GAAGGCCAGTGCCTGGTTCcaCTGGTACTAGAACGCC
LA-Y2F-I3H-R		tgGAACCAGGCACTGGCCTTCAGGCTCTTCTGCGCGG
LA-Y2F-I3L-F	LauA-Y2F-I3L	GAAGGCCAGTGCCTGGTTCcTCTGGTACTAGAACGCC
LA-Y2F-I3L-R		gGAACCAGGCACTGGCCTTCAGGCTCTTCTGCGCGGAC
LA-Y2F-I3P-F	LauA-Y2F-I3P	GAAGGCCAGTGCCTGGTTCccCTGGTACTAGAACGCC
LA-Y2F-I3P-R		ggGAACCAGGCACTGGCCTTCAGGCTCTTCTGCGCGG
LA-Y2F-I3Q-F	LauA-Y2F-I3Q	GAAGGCCAGTGCCTGGTTCcagTGGTACTAGAACGCC
LA-Y2F-I3Q-R		ctgGAACCAGGCACTGGCCTTCAGGCTCTTCTGCGCGG
LA-Y2F-I3R-F	LauA-Y2F-I3R	GAAGGCCAGTGCCTGGTTCcgCTGGTACTAGAACGCC
LA-Y2F-I3R-R		cgGAACCAGGCACTGGCCTTCAGGCTCTTCTGCGCGG
LA-Y2F-I3T-F	LauA-Y2F-I3T	GAAGGCCAGTGCCTGGTTCAcCTGGTACTAGAACGCC
LA-Y2F-I3T-R		gTGAACCAGGCACTGGCCTTCAGGCTCTTCTGCGCGGAC
For mutation of lauA-Y2X		
LA-Y2A-F	LauA-Y2A	CTGAAGGCCAGTGCCTGGgcCATCTGGTACTAGAACG
LA-Y2A-R		gcCCAGGCACTGGCCTTCAGGCTCTTCTGCGCGGAC
LA-Y2E-F	LauA-Y2E	CTGAAGGCCAGTGCCTGGgagATCTGGTACTAGAACGC
LA-Y2E-R		ctcCCAGGCACTGGCCTTCAGGCTCTTCTGCGCGGAC
LA-Y2H-F	LauA-Y2H	CTGAAGGCCAGTGCCTGGcaCATCTGGTACTAGAAC
LA-Y2H-R		tgCCAGGCACTGGCCTTCAGGCTCTTCTGCGCGGAC
LA-Y2P-F	LauA-Y2P	CTGAAGGCCAGTGCCTGGccCATCTGGTACTAGAACG
LA-Y2P-R		ggCCAGGCACTGGCCTTCAGGCTCTTCTGCGCGGAC
LA-Y2R-F	LauA-Y2R	CTGAAGGCCAGTGCCTGGcgCATCTGGTACTAGAACG
LA-Y2R-R		cgCCAGGCACTGGCCTTCAGGCTCTTCTGCGCGGACAC
LA-Y2T-F	LauA-Y2T	CTGAAGGCCAGTGCCTGGacCATCTGGTACTAGAACG
LA-Y2T-R		gtCCAGGCACTGGCCTTCAGGCTCTTCTGCGCGGACAC
LA-Y2V-F	LauA-Y2V	CTGAAGGCCAGTGCCTGGgTCATCTGGTACTAGAACG
LA-Y2V-R		cCCAGGCACTGGCCTTCAGGCTCTTCTGCGCGGACAC
LA-Y2W-F	LauA-Y2W	CTGAAGGCCAGTGCCTGGTggATCTGGTACTAGAACGC
LA-Y2W-R		ccACCAGGCACTGGCCTTCAGGCTCTTCTGCGCGGAC
For mutation of lauA-Y2F-Y5X		
LA-Y2F-Y5A-F	LauA-Y2F-Y5A	CAGTGCCTGGTTCATCTGGgcCTAGAACGCCTAGTC
LA-Y2F-Y5A-R		gcCCAGATGAACCAGGCACTGGCCTTCAGGCTCTTC
LA-Y2F-Y5C-F	LauA-Y2F-Y5C	CAGTGCCTGGTTCATCTGGtgCTAGAACGCCTAGTC
LA-Y2F-Y5C-R		caCCAGATGAACCAGGCACTGGCCTTCAGGCTCTTC
LA-Y2F-Y5E-F	LauA-Y2F-Y5E	CAGTGCCTGGTTCATCTGGgagTAGAACGCCTAGTCGTC
LA-Y2F-Y5E-R		ctcCCAGATGAACCAGGCACTGGCCTTCAGGCTCTTCTG
LA-Y2F-Y5F-F	LauA-Y2F-Y5F	GTGCCTGGTTCATCTGGTtCTAGAACGCCTAGTC
LA-Y2F-Y5F-R		aACCAGATGAACCAGGCACTGGCCTTCAGGCTC
LA-Y2F-Y5H-F	LauA-Y2F-Y5H	GTGCCTGGTTCATCTGGcACTAGAACGCCTAGTC
LA-Y2F-Y5H-R		gCCAGATGAACCAGGCACTGGCCTTCAGGCTCTTC
LA-Y2F-Y5L-F	LauA-Y2F-Y5L	GTGCCTGGTTCATCTGGctCTAGAACGCCTAGTC
LA-Y2F-Y5L-R		agCCAGATGAACCAGGCACTGGCCTTCAGGCTCTTC
LA-Y2F-Y5P-F	LauA-Y2F-Y5P	CAGTGCCTGGTTCATCTGGccCTAGAACGCCTAGTC
LA-Y2F-Y5P-R		ggCCAGATGAACCAGGCACTGGCCTTCAGGCTCTTC
LA-Y2F-Y5Q-F	LauA-Y2F-Y5Q	GTGCCTGGTTCATCTGGcAgTAGAACGCCTAGTCG

LA-Y2F-Y5Q-R		cTgCCAGATGAACCAGGCACTGGCCTTCAGGCTCTTC
LA-Y2F-Y5R-F	LauA-Y2F-Y5R	CAGTGCCTGGTTCATCTGGcgCTAGAACGCCTAGTC
LA-Y2F-Y5R-R		cgCCAGATGAACCAGGCACTGGCCTTCAGGCTCTTC
LA-Y2F-Y5T-F	LauA-Y2F-Y5T	CAGTGCCTGGTTCATCTGGacCTAGAACGCCTAGTC
LA-Y2F-Y5T-R		gtCCAGATGAACCAGGCACTGGCCTTCAGGCTCTTC
LA-Y2F-Y5W-F	LauA-Y2F-Y5W	GTGCCTGGTTCATCTGGTggTAGAACGCCTAGTCG
LA-Y2F-Y5W-R		ccACCAGATGAACCAGGCACTGGCCTTCAGGCTCTTC

Materials and methods

General materials

All chemicals, reagents and solvents were purchased from Sigma-Aldrich or Carl Roth (Germany). Primer synthesis and plasmids sequencing were performed by Microsynth AG (Germany). Q5 High Fidelity DNA polymerase, deoxynucleotides (dNTPs), restriction enzymes and HiFi DNA Assembly master mix were purchased from New England Biolabs (UK). Monarch® Genomic DNA Purification kit, Monarch® DNA gel extraction kit, Monarch® PCR Clean-up kit and Monarch® Plasmid miniprep kit were purchased from New England Biolabs (UK).

Strains and culture conditions

Bacterial strains and plasmids used in this study are summarized in Table S6. *Escherichia coli* strains were cultured in Luria-Bertani (LB) medium or on LB agar (10 g/L tryptone, 5 g/L yeast extract, 10 g/L NaCl, 20 g/L agar) overnight at 37 °C. *Streptomyces albus* J1074 and its derivatives were cultured on MS agar (20 g/L mannitol, 20 g/L soybean flour, 20 g/L agar) at 30 °C for growth, sporulation and conjugation (MS medium with 20 mM $MgCl_2$). *Xanthomonas translucens* DSM 18974 (medium 1) and *Streptomyces kanamyceticus* DSM 40500 (ISP2) were cultured at 30 °C in the corresponding medium for growth. Small-scale or large-scale fermentations of heterologous recombinant strains were performed in Tryptic soy broth medium (TSB) at 30 °C for 6 to 7 days. For the cultivation of strains harboring plasmids, the medium was supplementary with the corresponding antibiotics at a final concentration of 50 µg/mL for kanamycin, 50 µg/mL for chloramphenicol, 50 µg/mL for apramycin, and 12.5 µg/mL for tetracycline.

DNA isolation, manipulation and sequencing

The DNA sequence of the *xat* and *ska* BGCs are described in the last section of materials and methods. DNA isolation and manipulation in *Escherichia coli*, *Xanthomonas* and *Streptomyces* were performed using the Monarch® Genomic DNA Purification kit according to the manufacturer's instructions. Primers used in this study are listed in Table S7. PCR reactions using Q5 High Fidelity DNA polymerase were prepared according to the manufacturer's protocol. DNA fragments obtained by PCR were purified by agarose gel electrophoresis and isolated using the Monarch gel extraction kit according to the manufacturer's instructions. The amplified plasmid backbone was purified using a PCR Clean-up kit followed by *Dpn*I digestion according to the manufacturer's instructions.

Construction of recombinant plasmids

A) Plasmids used for combinatorial co-expression of chimeric precursor with *lauB1*

The primers used for constructing chimeric precursor-encoding genes (*lauA*$_L$-*scaA*$_C$) with *lauB1* are lists in Table S7. The plasmid pUWL201-OriT-*lauA*+*lauB1*[8] was reversely amplified. The reverse-amplified plasmid fragments were purified, digested with *Dpn*I, and cyclized by HiFi assembly. The assembled product was transformed into *E. coli* DH5a. The recombinant plasmids were confirmed by sequencing.

B) Plasmids used for site-directed mutagenesis

The primers used for site-directed mutagenesis are listed in Table S7. The plasmid pUWL201-OriT-*lauA*+*lauB1* were reversely amplified using the respective primer pairs bearing the introduced mutations, digested by *Dpn*I and then transformed into *E. coli* DH5a. The recombinant plasmids were confirmed by sequencing.

C) Plasmids used for biotransformation

The primers used for constructing plasmid pUWL201-OriT-*lauB1* are listed in Table S7. The plasmid pUWL201-OriT was reversely amplified to by primers, digested by *Dpn*I and then assembled by HiFi assembly kit to afford pUWL201-OriT with His tag. The sequenced plasmid pUWL201-His was linearized by digestion with *Hin*dIII and *Bam*HI. *lauB1* was amplified using pUWL201-OriT-*lauA*+*lauB1* as template. The linearized vector and amplified *lauB1* fragment were assembled by HiFi assembly kit and transformed into *E. coli* DH5a. The recombinant plasmids were confirmed by sequencing.

D) Plasmids used for deletion of *xal* gene cluster in *Xanthomonas* sp. leaf148

The primers used for constructing plasmid pL148_xal_KO are listed in Table S7. Approx. 600 bp regions up- and downstream of the *xal* BGC locus were PCR amplified from genomic DNA of *Xanthomonas* sp. Leaf148[10, 11] (*xal* BGC is located in region 1107068-1108315 of *Xanthomonas* Leaf148 contig_2, NCBI access number: LMOP01000008) and cloned into the mutagenesis vector pK18mobsacB (Table S6) via classical restriction enzyme cloning. The recombinant plasmids were confirmed by sequencing.

E) Plasmids constructed for the heterologous expression of the *xat* and *ska* BGCs

The primers used for constructing the plasmid pUWL201-OriT-*ska* and pME6012-*xat* are listed in Table S7. The *ska* and *xat* BGCs were amplified using the genomic DNA of *Streptomyces*

kanamyceticus DSM 40500 and *Xanthomonas translucens* DSM 18974 as template, respectively. The pUWL201-OriT and pME6012 were reversely amplified to afford the linearized backbone. After *Dpn*I digestion, the linearized pUWL201-OriT and pME6012 backbone were assembled with the amplified *ska* BGC or *xat* BGC-containing fragment, respectively, using HiFi assembly. The assembled plasmids were transformed into *E. coli* DH5a. The recombinant plasmids were confirmed by sequencing.

Generation of *Xanthomonas* sp. Leaf148/Δ*xal*

The in-frame deletion of the *xal* BGC in *Xanthomonas* sp. Leaf148 was performed as previously described.[10] Briefly, the plasmid pL148_xal_KO was transformed into *Xanthomonas* sp. Leaf148 by electroporation as described before.[12] Electroporated cells were regenerated in 1 mL of LB-Lennox for 4 h at 28°C with agitation and merodiploids having undergone one homologous recombination event were selected on LB supplemented with 50 μg/mL kanamycin and restreaked once on the same medium. Cell material was resuspended in 450 μL LB-Lennox, incubated for 2 h at 28°C with agitation and dilutions plated onto LB supplemented with 5.5% (w/v) sucrose to select for cells having undergone a second homologous recombination event. Recovered kanamycin sensitive and sucrose resistant colonies were subsequently screened for genomic loss of the *xal* locus by PCR. Mutants were confirmed using primers L148_xal_OF/ L148_xal_OR that bind outside the flanking regions used for pL148_xal_KO and primers L148_xal_AF/ L148_xal_AR that amplify a 324 bp product inside the deleted region.

Heterologous expression of engineered or native BGCs

Recombinant plasmids were introduced into *S. albus* J1074 by conjugation as described before.[12] After conjugation, ten exconjugants were picked and re-streaked on MS agar plates with apramycin (50 μg/mL) and trimethoprim (100 μg/mL) and incubated at 30 °C for three to four days. Three clones were randomly selected for small-scale fermentation.

Alternatively, the recombinant plasmids were introduced into *Xanthomonas* sp. leaf148/Δ*xal* by electroporation. The electrocompetent *Xanthomonas* sp. leaf148/Δ*xal* cell suspension was prepared as follows: *Xanthomonas* sp. leaf148/Δ*xal* was streaked on LB agar plates. The plate was incubated at 30 °C overnight. A single clone from the plate was transferred to 50 mL of LB liquid medium and incubated at 30 °C, 180 rpm to an OD_{600} 0.6 – 0.8. The cells were harvested, washed twice with 20 mL 10% pre-cooled glycerol and resuspended in 700 μL 10% pre-cooled

glycerol. 50 µL of resuspended cells were aliquoted into pre-cooled Eppendorf tubes, flash frozen, and stored at -80 °C. For electroporation, a tube containing 50 µL of electrocompetent cells was carefully thawed on ice for 10 min and then mixed with 5 µL of plasmid DNA (300 ng). The cell-plasmid mixture was transferred to a 0.1-cm pre-cooled electroporation cuvette and electroporated at 1.25 kV, 25 µF, 200 Ω. After electroporation, 400 µL of pre-cooled LB medium was immediately added to the electroporation cuvette and the cell suspension was transferred into a 1.5 mL EP tube and incubated at 30 °C, shaking for 3 to 4 h. After recovery, the cell suspension was plated on LB agar plates with 12.5 µg/mL tetracycline and incubated at 30 °C for 48 to 72 h.

Small-scale fermentation and LC-MS analysis

For small-scale fermentations of *Streptomyces*-derived recombinants, the spores of exconjugants were collected and transferred into 50 mL of TSB (250 mL Erlenmeyer flask) with 50 µg/mL apramycin and incubated at 30 °C, 180 rpm shaking for 6 days. On the fourth day, the culture was supplemented with 5% (w/v) Diaion HP-20 resin and cultured for another two days. The resin was collected by centrifugation (3214 x g, 10 min) and extracted with 15 mL acetone under sonication for 15 min. 800 µL of the extract was dried under reduced pressure and dissolved in 1 mL of methanol. After centrifugation (13222 x g, 10 min), 50 µL of the crude extract was mixed with 100 µL of methanol and subjected to LC-MS analysis.

For small-scale fermentations of *Xanthomonas*-derived recombinants, the recombinant *Xanthomonas* were streaked on the LB agar plates with 12.5 µg/mL tetracycline and incubated in 30 °C for two to three days. The recombinant strains were then transferred into ISP2 medium with 12.5 µg/mL tetracycline and incubated at 30 °C for five days. On the third day, the culture was supplemented with 5% (w/v) Diaion HP-20 resin and cultured for another two days. The resin was collected by centrifugation (3214 x g, 10 min) and extracted with 10 mL acetone under sonication for 15 min. 800 µL of the extract was dried under reduced pressure and dissolved in 1 mL of methanol. After centrifugation (13222 x g, 10 min), 50 µL of the crude extract was mixed with 50 µL of methanol, which was then subjected to LC-MS analysis.

High-performance liquid chromatography-electrospray ionization–quadrupole time of flight-high resolution mass spectrometry (HPLC-ESI-QTOF-HRMS) measurements were conducted on an Ultimate 3000 LC system (Thermo Fisher) coupled to an Impact II QTOF mass spectrometer

(Bruker) with an ACQUITY UPLC BEH C18 column (130 Å, 2.1 mm × 100 mm, 1.7 μm particle size, Waters) at a flow rate of 0.4 mL/min at 40 °C (5%- 95% acetonitrile/Milli-Q water both supplemented with 0.1 % (v/v) formic acid for 14 min). Data was acquired in positive mode at a scan range between 100 to 1200 *m*/*z*. A sample volume of 5 μL was injected for each analysis. The software DataAnalysis 4.3 (Bruker) and Metabolite Detect (Bruker) were used to analyze the data.

Large-scale fermentation and purification of compounds

The recombinant strains were streaked on MS agar plates with 50 μg/mL apramycin and 100 μg/mL trimethoprim and incubated at 30 °C for 7 days. The spores were collected from one MS agar plate and transferred into five 1 L Erlenmeyer flask with 200 mL of TSB medium with 50 μg/mL apramycin. A total of 10 L of culture was prepared for each recombinant strain. The cultures were incubated at 30 °C, 180 rpm shaking for 7 days, then supplemented with 5% (w/v) sterilized Diaion HP-20 resin at day four and incubated for three additional days. The resin was collected by filtration through a metal sieve (40 mesh). The harvested resin was washed with water and extracted with around 5 L of acetone. The extract was dried under reduced pressure. The obtained crude extract was subjected to normal phase silica gel column chromatography (230 to 400 mesh) and eluted with $CHCl_3$/CH_3OH (1:0, 4:1, 2:1, 0:1 (4 times), v/v, 600 mL) to yield 7 fractions (Fr. 1 to Fr. 7).

Compounds **2**, **3**, and **4** were purified from the crude extract of the recombinant strains *S. albus*/*lauA*$_L$-*scaA*$_C$ +*lauB1*. Compound **2** was first observed in Fr. 3 after normal phase silica gel column chromatography. This fraction was subjected to semi-preparative HPLC using a cholester C18 column (10ID × 250 mm, COSMOSIL) to afford compound **2** (2.9 mg, t_R = 9.5 min) with an isocratic elution of acetonitrile/water 35% (0-20min) at a flow rate of 3 mL/min. Compounds **3** and **4** were observed in Fr. 5 and Fr. 6 after normal phase silica gel column chromatography. These two fractions were subjected to semi-preparative HPLC using a cholester C18 column (10ID × 250 mm, COSMOSIL) to afford Fr.1.1 (t_R = 20.6 min, containing compound **2**) and Fr.1.2 (t_R = 13.0 min, containing compound **3**) with a gradient elution of acetonitrile/water 10% to 40% (0-20 min) at a flow rate of 3 mL/min. Fr.1.1 was subject to another round of semi-preparative HPLC-based purification using a XBridge® Peptide BEH C18 OBD Prep column (10 mm × 250 mm, 5 μm particle size, Waters) to afford compound **3** (1.2 mg, t_R = 17.0 min) with an isocratic elution of acetonitrile/water 26% (0-20min) at a flow rate of 3 mL/min. Fr.1.2 was further subject to another

round of semi-preparative HPLC-based separation using a XBridge® BEH Amide OBD Prep column (10 mm × 250 mm, 5 µm particle size, Waters) to afford compound **4** (0.9 mg, t_R = 13.0 min) with an isocratic elution of acetonitrile/water 70 % (0-30min) at a flow rate of 3 mL/min.

Compounds **4** and compound **5** were purified from the crude extract of the recombinant strains *S. albus*/*lauA*-6S+*lauB1*. Compound **5** was first observed in Fr. 3 and Fr. 4 after normal phase silica gel column chromatography. These two fractions were subjected to preparative HPLC using a cholester C18 column (20ID × 250 mm, COSMOSIL) to afford Fr.1.1 (t_R = 13.0 min, containing compound **5**) with a gradient elution of acetonitrile/water 20% to 60% (0-20 min) at a flow rate of 10 mL/min. Fr.1.1 was further subjected to semi-preparative HPLC using a cholester C18 column (10ID × 250 mm, COSMOSIL) to afford compound **5** (3.5 mg, t_R = 14.0 min) with an isocratic elution of acetonitrile/water 28 % (0-20min) at a flow rate of 3 mL/min. Compound **4** was observed in Fr. 5 and Fr. 6 after normal phase silica gel column chromatography. These two fractions were subjected to preparative HPLC using an Edipse XDB C18 column (21.2 × 250 mm, 7 µm particle size, Aglilent) to afford Fr.1.1 (t_R = 14.8 min, containing compound **4**) with a gradient elution of acetonitrile/water 15% to 30% (0-20min) at a flow rate of 10 mL/min. Fr.1.1 was further subjected to semi-preparative HPLC using a XBridge® Peptide BEH C18 OBD Prep column (10 mm × 250 mm, 5 µm particle size, Waters) to afford compound **4** (8.3 mg, t_R = 16.5 min) with a gradient elution of acetonitrile/water 18% to 20% (0-20min) at a flow rate of 3 mL/min.

Compound **6** was purified from the crude extract of the recombinant strains *S. albus*/*lauA*-Y2F+*lauB1*. Compound **6** was first observed in Fr. 6 and Fr. 7 after normal phase silica gel column chromatography. These two fractions were subjected to preparative HPLC using an Eclipse XDB C18 column (21.2 × 250 mm, 7 µm particle size, Aglilent) to afford Fr.1.1 (t_R = 15.0 min, containing compound **5**) with a gradient elution of acetonitrile/water 15% to 30% (0-20min) at a flow rate of 10 mL/min. Fr.1.1 was further subjected to semi-preparative HPLC (Altantis™ Premier BEH C18 A × 5uM, OBD™ 10 mm X 250mm) to afford compound **6** (6.3 mg, t_R = 18.0 min) with an isocratic elution of acetonitrile/water 20 % (0-20min) at a flow rate of 3 mL/min.

Compound **7** was isolated from the NMR sample of compound **6** in DMSO-d_6. The NMR sample was subjected to semi-preparative HPLC with a XBridge® BEH Amide OBD Prep column (10 mm × 250 mm, 5 µm particle size, Waters) to afford compound **7** (5.0 mg, t_R = 6.7 min) with an isocratic elution of acetonitrile/water 78 % (0-20 min) at a flow rate of 3 mL/min.

Compounds **9** and compound **10** were purified from the crude extract of the recombinant strain *S. albus*/*ska*. Compounds **9** and **10** were mainly observed in Fr. 7 after normal phase silica gel column chromatograph. Fraction Fr. 7 was subjected to preparative HPLC using a phenyl-hexyl column (21.2 mm× 250 mm, 5μm particle size, phenomenex) with a gradient elution of acetonitrile/water 15% to 30% (0-20min), 30% to 50% (20.1 to 32 min) to afford Fr.1.1 (t_R = 20.0 min, containing compound **9**) and Fr.1.2 (t_R = 30.0 min, containing compound **10**). Fr.1.1 was subjected to semi-preparative HPLC using an Eclipse XDB-C18 column (9.4 mm× 250 mm, 5μm particle size, Agilent) to afford Fr.2.1 (t_R = 14.0 min, containing compound **9**) with an isocratic elution of acetonitrile/water 17% (0-20min) at a flow rate of 3 mL/min. Fr.2.1 was further subject to semi-preparative HPLC with a XBridge® Peptide BEH C18 OBD Prep column (10 mm × 250 mm, 5 μm particle size, Waters) to afford compound **9**(2.0 mg, t_R = 21.5 min) with an isocratic elution of acetonitrile/water 17 % (0-30min) at a flow rate of 3 mL/min. Fr.1.2 was subjected to semi-preparative HPLC using a XBridge® Peptide BEH C18 OBD Prep column (10 mm × 250 mm, 5 μm particle size, Waters) to afford compound **10** (2.90 mg, t_R = 19.5 min) with an isocratic elution of 35% acetonitrile/water supplemented with 0.1% TFA (0-20min) at a flow rate of 3 mL/min.

Physical and spectroscopic data for the isolated compounds

Compound **2**: yellow powder; ^{1}H and ^{13}C NMR data, see Figures S2 and S3; HRMS (ESI-QTOF) *m*/*z*: 649.3129 $[M + H]^+$ (calcd for $C_{37}H_{41}N_6O_5^+$, 649.3133).

Compound **3**: yellow powder; ^{1}H and ^{13}C NMR data, see Table S1; HRMS (ESI-QTOF) *m*/*z*: 970.4457 $[M + H]^+$ (calcd for $C_{52}H_{60}N_9O_{10}^+$, 970.4458).

Compound **4**: white powder; ^{1}H and ^{13}C NMR data, see Table S2; HRMS (ESI-QTOF) *m*/*z*: 770.3507 $[M + H]^+$ (calcd for $C_{40}H_{48}N_7O_9^+$, 770.3508).

Compound **5**: yellow powder; ^{1}H and ^{13}C NMR data, see Figures S21 and S22; HRMS (ESI-QTOF) *m*/*z*: 665.3080 $[M + H]^+$ (calcd for $C_{37}H_{41}N_6O_6^+$, 665.3082).

Compound **6**: white powder; ^{1}H and ^{13}C NMR data, see Table S3; HRMS (ESI-QTOF) *m*/*z*: 683.3188 $[M + H]^+$ (calcd for $C_{37}H_{43}N_6O_7^+$, 683.3188).

Compound **7**: yellow powder; ^{1}H and ^{13}C NMR data, see Table S4; HRMS (ESI-QTOF) *m*/*z*: 502.2446 $[M + H]^+$ (calcd for $C_{28}H_{32}N_5O_4^+$, 502.2449).

Compound **9**: white powder; ^{1}H and ^{13}C NMR data, see Table S5; HRMS (ESI-QTOF) *m*/*z*: 707.2937 $[M + H]^+$ (calcd for $C_{37}H_{39}N_8O_7^+$, 707.2936).

Compound **10**: yellow powder; ^1H and ^{13}C NMR data, see Figures S76 and S77; HRMS (ESI-QTOF) *m/z:* 850.3290 $[M + H]^+$ (calcd for $C_{46}H_{44}N_9O_8^+$, 850.3307).

NMR analysis

^1H, ^{13}C, ^1H-^1H COSY, HMQC, HMBC and NOESY NMR spectra were recorded on Bruker AV500 and DRX600 spectrometers. Chemical shifts (δ) were acquired in ppm with reference to the residual solvents of DMSO-d_6 (δ 2.50 for ^1H NMR; δ 39.51 for ^{13}C NMR); MeOD (δ 3.31 for ^1H NMR; δ 49.15 for ^{13}C NMR), D_2O with TMS (δ 4.80 for ^1H NMR; δ 0 for ^{13}C NMR). Spectra were processed using TOPSPIN 4.3.0 (Bruker).

Structure elucidation

Compound **2** was isolated as a yellow powder. Its molecular formula was determined to be $C_{37}H_{40}N_6O_5$ based on a protonated ion at *m/z* 649.3129 (calcd for $C_{37}H_{41}N_6O_5^+$, 649.3133, Δ -0.62 ppm) in HR-ESI-QTOF-MS data (Figure S2). The ^1H and ^{13}C NMR spectra (Figure S3 and S4) of **2** were identical to those of the known compound scabrirubin.[8]

Compound **3** was isolated as a yellow powder. Its molecular formula was determined to be $C_{52}H_{59}N_9O_{10}$ based on a protonated ion at *m/z* 970.4457 (calcd for $C_{52}H_{60}N_9O_{10}^+$, 970.4458, Δ -0.10 ppm) in HR-ESI-QTOF-MS spectrum (Figure S5). Analysis of the ^1H and ^{13}C NMR spectra (Figure S6 and S7, Table S1), with the aid of the HSQC spectrum (Figure S9), revealed the presence of 52 carbons including seven carbonyl carbons, nine nonprotonated sp^2 carbons, 17 sp^2 methines, a quaternary carbon, nine sp^3 methines, six sp^3 methylenes, and three methyls. These signals and amide proton signals (δ_H 10.86, 8.25, 8.17, 8.02, 7.44, 7.39, 6.93) are characteristic for peptides (Table S1). The presence of a Phe residue was determined based on the ^1H-^1H COSY correlations between H-32/32' with H-33/33', H-33/33' with H-34 and the HMBC correlations from H-32 to C-30 and from H-30 to C-31 (Figures S8 and S10). The ^1H-^1H COSY correlations between H-27/H-24, H-24/H-23, H-23/23-NH, and H-26/H-25 along with the HMBC correlations from H-26 to C-24 and from H-27 to C-23 and C-25 suggested the presence of an Ile residue. A typical AA'XX' spin system (δ_H 6.82, 6.53) and benzyl methylene signals (δ_H 2.27, 2.43) in the ^1H NMR spectrum indicate the presence of a Tyr residue, which is supported by the HMBC correlations of H-5/C-7, H-6/C-8, H-8'/C-6, H-8'/C-10, H-9'/C-7 (Figure S10). The C-42-substitued Trp (Trp-1) was determined based on COSY correlations of H-39/39-NH, H-44/H-45, H-36/H-37 and H-36/36-NH (Figure S8) as well as HMBC correlations from 39-NH to C-38, C-40 and C-45, from H-39 to C-38, from H-37 to C-39 and C-40, from H-41 to C-43 and C45, and from H-43 to C-

45 (Figure S10). The ^{1}H-^{1}H COSY correlation of H-17/H-18, H-18/H-19, H-19/H-20, H-12/H-13 and H-15/15-NH (Figure S8) along with HMBC correlations from H-12 to C-14 and C-16, from H-15 to C-12, C-13 and C-16, from H-17 to C-14, C-16, C-19 and C-21, and from H-19 to C-21 (Figure S10) indicate the presence of a modified Trp residue (Trp-2). C-12 and C-15 were connected via 12-N, which was determined by HMBC correlation from H-15 to C-12 (Figure S10). The ^{1}H-^{1}H COSY correlations of H-47/H-48 as well as the HMBC correlation from H-48 to C-46 suggest the presence of an alanine residue (Figure S8 and S10). The COSY correlation of H-3/H-2 and H-2/2-NH as well as HMBC correlation from H-3 to C-1 indicate the presence of a Ser residue (Figure S8 and S10). The amino acid sequence of **3** was determined to be NH_2-Ala-Trp1-Phe-Ile-Trp2-Tyr-Ser-CO_2H based on the COSY and HMBC correlations (Figure S8 and S10), which is consistent with the core peptide sequence AWFIWYS of the chimeric precursor $LauA_L$-$ScaA_C$. The C-C bond between C-42 of Trp-1 and C-14 of Trp-2 was determined by the HMBC correlations from H-43 and H-41 to C-14, from H-13 to C-42 (Figure S10). The stereochemistry of the α-carbons for each amino acid was deduced as L-configured based on the ribosomal biosynthetic origin.

Compound **4** was isolated as a white powder. Its molecular formula was determined to be $C_{40}H_{47}N_7O_9$ based on a protonated ion at *m/z* 770.3507 (calcd for $C_{40}H_{48}N_7O_9^+$, 770.3508, Δ -0.12 ppm) in HR-ESI-QTOF-MS data (Figure S12). Analysis of the ^{1}H and ^{13}C NMR spectra (Figure S13 and S14, Table S2), with the aid of the HSQC spectrum (Figure S16), revealed the presence of 40 carbons including five carbonyl carbons, eight nonprotonated sp^2 carbons, 12 sp^2 methines, a quaternary carbon, seven sp^3 methines, five sp^3 methylenes, and two methyls. A Tyr residue was confirmed based on a typical AA'XX' spin system (δ_H 6.82, 7.11) and benzyl methylene signals (δ_H 2.65, 3.04) in the ^{1}H NMR spectrum, which is supported by the HMBC correlations from H-5 to C-4, C-7 and C-11, from H-6 to C-8, from H-8' to C-6 and C-10, from H-9' to C-7 and C-9' (Figure S17). A constituted spin system between two methyls (δ_H 0.91, 0.99), two methines (δ_H 1.93, 3.19), one methylene (δ_H 1.51, 1.15), and HMBC correlations from H-23 to C-27, C-25 and C-23 indicate the presence of an Ile residue (Figure S17). The C-35-substitued Trp (Trp-1) was determined based on COSY correlations of H-36/H-37 and H-29/H-30 as well as HMBC correlations from H-32 to C-30. C-31, C33 and C-38, from H-30 to C-33, from H-29 to C-28 and C-31, from H-34 to C-38, C-36 and from H-37 to C-33 and C-35 (Figure S15 and S17). The ^{1}H-^{1}H COSY correlation of H-17/H-18 and H-18/H-19 along with HMBC correlation from H-13 to C-

11, C-14 and C-16, from H-15 to C-12, C-13 and C-14, from H-17 to C-14, C-19 and C-21, and from H-20 to C-16 and C-18 suggested the presence of a modified Trp-2 residue (Figure S15 and S17). C-12 and C-15 were connected via 12-N, which was determined by HMBC correlation from H-15 to C-12 (Figure S17). The COSY correlation of H-3/H-2 and H-2/2-NH as well as HMBC correlations from H-3 to C-1 and from H-2 to C-1 and C-4 indicate the presence of a Ser residue (Figure S15 and S17). The C-C bond between C-35 of Trp-1 and C-14 of Trp-2 was determined by the HMBC correlations from H-36 and H-34 to C-14, from H-15 to C-35 (Figure S17). Compared with the core peptide sequence WFIWYS of the chimeric precursor of $LauA_L$-$ScaA_C$, the second amino acid Phe is absent in compound **4**. The stereochemistry of the α-carbons for each amino acid was deduced as L-configured based on the ribosomal biosynthetic origin.

Compound **5** was isolated as a yellow powder. Its molecular formula was determined to be $C_{37}H_{40}N_6O_6$ based on a protonated ion at *m*/*z* 665.3080 (calcd for $C_{37}H_{41}N_6O_6^+$, 665.3082, Δ -0.30 ppm) in HR-ESI-QTOF-MS data (Figure S20). The ^1H and ^{13}C NMR spectra of **5** (Figure S21 and S22) were identical to those of known compound scabrirubin CB-1.[13]

Compound **6** was isolated as a white powder. Its molecular formula was determined to be $C_{37}H_{42}N_6O_7$ based on a protonated ion at *m*/*z* 683.3188 (calcd for $C_{37}H_{42}N_6O_7^+$, 683.3188, Δ 0.00 ppm) in HR-ESI-QTOF-MS data (Figure S45). Analysis of the ^1H and ^{13}C NMR spectra (Figure S46 and S47, Table S3), with the aid of the HSQC spectrum (Figure S49), revealed the presence of 37 carbons including four carbonyl carbons, eight nonprotonated sp^2 carbons, 12 sp^2 methines, a quaternary carbon, six sp^3 methines, four sp^3 methylenes, and two methyls. The ^1H and ^{13}C NMR spectra of **6** closely resemble those of **4,** except for the absence of the signals for the serine residue (Figure S46 and S47, Table S3). This suggests that **6** is a congener of **4** lacking the C-terminal serine. The HMBC correlation of H-33/C-11, H-31/C-11 and H-12/C-32 and the NOESY correlation of H-12/H-33 and H-10/H-33 confirmed the presence of a C-C bond between C-32 of Trp1 and C-11 of Trp2 (Figure S50 and S51). The stereochemistry of the α-carbons for each amino acid was deduced as L-configured based on the ribosomal biosynthetic origin.

Compound **7** was isolated as a yellow powder. Its molecular formula was determined to be $C_{28}H_{31}N_5O_4$ based on a protonated ion at *m*/*z* 502.2446 (calcd for $C_{28}H_{32}N_5O_4^+$, 502.2449, Δ -0.60 ppm) in HR-ESI-QTOF-MS data (Figure S37). Analysis of the ^1H and ^{13}C NMR spectra (Figure S38 and S39, Table S4), with the aid of the HSQC spectrum (Figure S41), revealed the presence of 28 carbons including three carbonyl carbons, six nonprotonated sp^2 carbons, eight sp^2

methines, a quaternary carbon, five sp^3 methines, three sp^3 methylenes, and two methyls. The COSY correlation showed a constituted spin system between two methyls (δ_H 0.84, 1.01), two methines (δ_H 2.02, 4.05), one methylene (δ_H 1.30, 1.39) (Figure S40), indicating the presence of an Ile residue, which was supported by the HMBC correlations from 12-NH to C-25, and from H-24 to C-23 and C-28 (Figure S42). The C-8-substitued Trp was determined based on COSY correlations of H-5/5-NH, H-9/H-10 and H-2/H-3 as well as HMBC correlations from H-2 and H-3 to C-1, from H-3 to C-4, C-5 and C-6, from H-5 to C-6, from 5-NH to C-4, C-6 and C-11, from H-7 to C-11 and C-9, from H-10 to C-8 and from H-9 to C-11 (Figure S40 and S42). The ^1H-^1H COSY correlation of 16-NH/H-16, H-13/H-14, H-18/H-19, H-19/H-20 and H-20/H-21 along with HMBC correlations from H-16 to C-13, C-14 and C-22, from H-21 to C-17 and C-19, from H-20 to C-18 and C-22 and from H-18 to C-22 indicated the presence of another Trp-2 residue (Figure S40 and S42). C-13 and C-16 were connected via 13-N, which was determined by HMBC correlation from H-16 to C-13 (Figure S42). The C-C bond between C-8 of Trp-1 and C-15 of Trp-2 was determined by the HMBC correlations from H-7 and H-9 to C-15 and from H-14 and H-16 to C-8 (Figure S42). The HMBC correlation from 12-NH to C-12, C-23 and C-13 indicate a new amide bond formed between the amino group of Ile and the carboxyl acid of Trp-2 (Figure S42). Therefore, **7** was identified as a diketopiperazine.

Compound **9** was isolated as a white powder. Its molecular formula was determined to be $C_{37}H_{38}N_8O_7$ based on a protonated ion at *m/z* 707.2937 (calcd for $C_{37}H_{39}N_8O_7^+$, 707.2936, Δ -0.14 ppm) in HR-ESI-QTOF-MS data (Figure S68). Analysis of the ^1H and ^{13}C NMR spectra (Figure S69 and S70, Table S5), with the aid of the HSQC spectrum (Figure S72), revealed the presence of 37 carbons including four carbonyl carbons, nine nonprotonated sp^2 carbons, 14 sp^2 methines, a quaternary carbon, five sp^3 methines, four sp^3 methylenes. A typical AA'XX' spin system (δ_H 7.04, 6.79) and benzyl methylene signals (δ_H 2.56, 2.85) in the ^1H NMR spectrum indicate the presence of a Tyrosine residue, which is supported by HMBC correlations from H-2 to C-1 and C-4, from H-5 to C-5', C-7 and C-3, and from H-6' to C-4 and C-6 (Figure S73). The COSY correlation of H-20/H-21 as well as HMBC correlations from H-20 to C-19, from H-21 to C-19, C-22, and C-14, from C-24 to C-22, and from H-23 to C-22 and C-24 suggest the presence of a His residue (Figure S71 and S73). The C-32-substituted Trp was determined based on COSY correlations of H-33/H-34 and H-26/H-27 as well as HMBC correlations from H-27 to C-28 and C-29, from H-26 to C-25 and C-28, from H-29 to C-28, C-30 and C-35, from H-34 to C-30 and C-32, and from H-31 to C-28 and C-33 (Figure S71 and S73). The ^1H-^1H COSY correlation of H-14/H-

15, H-15/H-16, H-16/H-17 and H-9/H-10 along with HMBC correlation from H-9 to C-8 and C-11, from H-10 to C-13, from H-12 to C-9, C-11, C-13 and C-18, from H-16 to C-14 and C-18, and from H-17 to C-13 and C-15 indicate the presence of another Trp residue (Figure S71 and S73). C-9 and C-12 were connected via 9-N, which was determined by HMBC correlation from H-12 to C-9 (Figure S73). The C-C bond between C-32 of Trp-1 and C-11 of Trp-2 was determined by HMBC correlations from H-31 and H-33 to C-11 and from H-12 to C-32 (Figure S73). Compared with the core peptide sequence WYHWY of SkaA, the second amino acid Tyr is absent in compound **9**. The stereochemistry of the α-carbons for each amino acid was deduced as L-configured based on the ribosomal biosynthetic origin.

Compound **10** was isolated as a yellow powder. Its molecular formula was determined to be $C_{46}H_{43}N_9O_8$ based on a protonated ion at *m*/*z* 850.3290 (calcd for $C_{46}H_{44}N_9O_8^+$, 850.3307, Δ -0.30 ppm) in HR-ESI-QTOF-MS data (Figure S74). The ^1H and ^{13}C NMR spectra of **10** (Figure S75 and S76) were identical to those of a known compound purified from *S. albus*/*azaAB* strain.[1]

Investigation of conditions for the spontaneous transformation of atropopeptides into DKPs

The excised atropopeptide derivatives **4**, **6** or **9** (1 μL, 20 mM) were added into Eppendorf tubes, respectively, dried and redissolved in 20 μL solvent (DMSO, DMSO with 0.5% acetic acid, or water) and incubated at room temperature for 48h. Subsequently, 2 μL of the reaction mixture were diluted with 30 μL methanol, and 5 μL of the resulting solution were subjected to LC-MS analysis.

Investigation of DKP formation directly in crude extracts resuspended in DMSO was performed as follows. Crude extracts obtained mainly from small-scale fermentation of LauA-Y2F-I3X mutants (as described in the "**Small-scale fermentation and LC-MS analysis**" section) were dissolved with 100 μL of DMSO without the addition of acid or base and incubated at room temperature for 10 days. Subsequently, 10 μL of each sample was diluted with 70 μL of methanol. Following centrifugation (13222 x g, 30 min), 5 μL of the resulting supernatant was subjected to LC-MS analysis to investigate DKP formation.

Molecular dynamic simulations

Before molecular dynamics simulations, geometry was optimized in Gaussian version 16.B.01 with b3lyp/6-31g(d,p). Molecular dynamics simulations were performed using Amber 22. The structure was prepared and parameterized using antechamber (options: "-fo prepi -c bcc -nc 0 -at gaff2") and parmchk2, tleap was then used to prepare the molecule for simulation. We first performed simulations in gas phase to sample conformational space. Steepest descent minimization was initiated in 250 steps of steepest gradient descent, followed by 250 steps of conjugate gradient descent. The production gas phase simulation was then run on the resulting structure for 1 μs with a time step of 2 fs, with a frame saved every 2 ps. We chose the structures from the frames with the five shortest distances, as measured by cpptraj, between the proposed reactive atoms and used them as starting points for solvated simulations. Molecules in explicit solvent were prepared in tleap and solvated in a truncated octagonal box with a buffer of 14 Å. Water solvation used the SPC/E water model and DMSO boxes and parameters were obtained from http://amber.manchester.ac.uk/.[14] Minimization of solvent was performed with a restraining force of 500.0 kcal/mol $Å^{-2}$ on the peptide was initiated in 500 steps, followed by 500 steps of conjugate gradient minimization. This was then followed by minimization of the entire system with 1000 steps of steepest descent minimization, followed by another 1500 steps of conjugate gradient minimization. Subsequent equilibration and heating of the system from 0 to 300 K using the Langevin temperature scheme and a 10.0 kcal/mol $Å^{-2}$ constraint on the peptide for 20 ps simulation were performed. Final equilibration at a constant pressure of 1 atm and constant temperature at 300 K for 100 ps was conducted to relax the system to an equilibrium density. Production simulations were run at constant pressure for at least 200 ns with a step of 2 fs with frames saved every 50 ps. Cpptraj was then used to extract distances between the N-terminus nitrogen and carbon in the carbonyl of the amide.

DTF calculations

All quantum chemical calculations were performed using the Gaussian 16, Revision A.03 program package.[15] Geometry optimizations were carried out without imposing symmetry constraints using density functional theory (DFT) at the ωB97XD/def2-SVP level of theory.[16, 17] Solvent effects were taken into account using the SMD implicit solvation model with dimethyl sulfoxide (DMSO, epsilon = 46.7) and water (epsilon = 78.36) as the solvent.[18] Harmonic vibrational frequency analyses were performed at the same level of theory to confirm that all optimized structures correspond to

true minima on the potential energy surface, as indicated by the absence of imaginary frequencies, and to obtain thermochemical corrections. Transition states were located and optimized at the same level of theory and were characterized by the presence of a single imaginary frequency corresponding to the reaction coordinate. Intrinsic reaction coordinate (IRC) calculations were further carried out to verify that each transition state properly connects the corresponding reactants and products. Thermochemical corrections to the Gibbs free energies were obtained using the GoodVibes program,[19] applying quasi-harmonic corrections to account for low-frequency vibrational modes.

Detailed mechanistic insights of diketopiperazine formation

In the initial step, the nucleophilic addition of N-20 to the carbonyl carbon C-8 is facilitated by proton transfer mediated by the carboxyl group of another molecule, leading to C–N bond formation and generation of a six-membered ring (**INT1a**, Figure S54). These elementary processes occur within a single highly coupled transition state with a calculated activation free energy of 9 kcal/mol, supporting that this reaction is kinetically feasible under ambient conditions.

For the subsequent C–N bond cleavage, two catalytic scenarios were investigated: one involving an undissociated carboxyl group (purple, Figure S54) and the other in its absence (green, Figure S54). Without participation of a carboxyl group, proton transfer and C–N bond cleavage proceeds through a concerted intramolecular pathway, in which the proton is directly transferred from the hydroxy group at C-8 to the nitrogen atom at C-2 within the same molecule. This 1,3-proton shift requires a highly strained four-membered-ring-like coordinate of a hemiaminal, resulting in a high free energy barrier of 28.2 kcal/mol. Such a large barrier indicates that this rearrangement is kinetically unfavorable under the modeled conditions without an external proton donor to relieve the geometric constraints.

In the presence of the undissociated carboxyl group, the carboxyl group in the model undergoes a conformational change to establish a hydrogen bonding network with the hydroxy group at C-8 and the amide nitrogen at C-2 (**INT1b**, Figure S54). This exothermic process with −1.8 kcal/mol results in a stabilized precursor for the subsequent proton shuttle mechanism. In the second step, the carboxyl group again mediates the proton transfer process. First, a proton is transferred from the carboxyl group to the nitrogen atom at C-2 through a thermodynamically degenerate transition state. Although this transition state corresponds to a maximum on the potential energy surface (red wireframe, Figure S54), its Gibbs free energy after thermal correction is slightly lower than

that of the product. Although the transition state was confirmed as a first-order saddle point by vibrational and IRC analyses, thermal corrections resulted in a slightly lower Gibbs free energy for the TS relative to the product (~1 kcal mol^{-1}). Similar shallow or submerged free-energy barriers have been attributed to entropy–enthalpy compensation and the sensitivity of RRHO/quasi-harmonic treatments to low-frequency vibrational modes.[20-21] Subsequently, the proton on the hydroxy group at C-8 is transferred to the carboxyl group, concomitant with cleavage of the C–N bond (**INT2**, Figure S54). This process proceeds through a well-defined transition state with a low free energy barrier of 3.7 kcal/mol. Overall, the carboxyl group functions as both proton donor and acceptor.

Throughout the process, DMSO is proposed to preserve the carboxyl group in its undissociated form. In contrast, the reaction is not observed in water (Figure S44), likely because the carboxyl group cannot function as proton donor and acceptor since it dissociates in water. In summary, our DFT calculations suggest that the carboxyl group of a neighboring atropopeptide molecule acts as both proton donor and acceptor during nucleophilic addition and C–N bond cleavage, ultimately leading to DKP formation.

Feeding experiments

The spores of *S. albus* and *S. albus* expressing *lauB1* were inoculated in 5 mL TSB medium in 50 mL flasks, with 50 μg/mL apramycin (for plasmid-bearing strains) and incubated at 28 °C with shaking at 200 rpm for two days. TSB medium (5 mL) served as the control. All cultures were supplemented with 5 mL fresh TSB medium and 6 μL of 20 mM **3** on the third day. After incubation for another 3 days, the cultures were harvested by centrifugation to afford the cell pellet and the supernatant. The cell pellet was extracted with 2 mL acetone. The compounds in the supernatant or TSB medium were absorbed onto Diaion HP-20 resin (5% w/v). After one day of incubation with resin, it was obtained by centrifugation and extracted with 2 mL acetone. The extracts were analyzed by LC-MS.

Crude enzyme extract assays

The spores of *S. albus* or *S. kanamyceticus* were inoculated into 20 mL TSB medium in 100 mL flasks and incubated at 28 °C with shaking at 200 rpm for two days. A single clone of *X. translucens* pv. *translucens* was inoculated into 20 mL LB medium in 100 mL flasks and cultured

under the same conditions. Cell pellets from 5 mL of culture were harvested, resuspended in 2 mL of Tris-HCl buffer (50 mM Tris-HCl, 100 mM NaCl, pH 7.6) and lysed by sonication. After centrifugation (13222 x g, 5 min, 4°C), the supernatant was collected and used as the crude enzyme extract for the reaction assays.

The 50 µL reaction mixture contained 0.05 mM **3** and either reaction buffer (50 mM Tris-HCl, 50 mM NaCl, pH 7.5), crude enzyme extract prepared in the same buffer or the boiled crude enzyme extract. Reactions were incubated at 30 °C for 1 h or 12 h and terminated by the addition of 50 µL of methanol. Following centrifugation (13222 x g, 30 min), 5 µL of the supernatant was subjected to LC-MS analysis.

Bioactivity investigation

(1) Antimicrobial assays

Disk diffusion assays were first used for the evaluation of the antimicrobial activity of compounds **6** and **7**. Compounds **6** and **7** were dissolved in water and DMSO, respectively at a concentration of 2.56 mg/mL. The ESKAPE pathogens (*Enterococcus faecium*, *Straphylococcus aureus*, *Klebsiella pneumonia*, *Acinetobacter baumannii*, *Pseudomonas aeruginosa*, *Enterobacter cloacae*) were used as reporter strains, which were cultured in LB medium at 37 °C (130 rpm) to an OD_{600} value of ~ 0.6. The cultures were mixed with LB agar to prepare the agar plates in a ratio of 1:100. Trimethoprim and ampicillin were used as positive controls. The 6 mm paper disks that were loaded with 5 µL of each compound, were placed onto the agar plates. The plates were incubated at 37 °C for 16 h. The antimicrobial activity was determined by the size of the inhibition zone.

(2) Antiviral assays

a) Cell culture

Madin-Darby canine kidney II (MDCK II) cells were maintained in Dulbecco's modified Eagle's medium (DMEM) GlutaMAX, supplemented with 10% FCS and 1% penicillin/streptomycin (P/S). The cells were incubated at 37 °C in a 5% CO_2 atmosphere. All media and supplements were purchased from Thermo Fisher Scientific.

b) Cytotoxity screening in cell culture

Cells were seeded in 96-well plates and incubated at 37 °C in a 5% CO_2 atmosphere until they reached 90% confluence, as determined by microscopy. Subsequently, the cells were treated

with compounds **6** and **7**, ionomycin, water or DMSO. Compounds **6** and **7** were dissolved in water and DMSO, respectively; ionomycin was dissolved in DMSO. All compounds were prepared as 10 mM stock solutions, with a final concentration of 100 µM in the assay. The final concentration of DMSO was 1% (vol/vol). Plates were incubated for 48 hours, after which cell viability was assessed by measuring ATP content using the CellTiter-Glo Luminescent Cell Viability Assay (Promega) according to the manufacturer's instructions. The luminescence was recorded using black 96-well plates in a Synergy H4 microplate reader (Biotek/Agilent). Luminescence readings were normalized to the untreated cells, which were set to 100%.

c) Virus preparation

The influenza viruses utilized in this study was strain A/Hamburg/05/2009 (H1N1pdm). The viruses were propagated in MDCK II cells with infection medium (DMEM GlutaMAX) supplemented with 0.2% BSA (bovine serum albumin, Carl Roth), 1% P/S and 1 µg/mL bovine N-tosyl-l-phenylalanine chloromethyl ketone (TPCK)-treated trypsin (Thermo Fisher Scientific) at a multiplicity of infection (MOI) of 0.001–1. Following a 48-hour incubation period, the cell supernatants were centrifuged (2,000 x g, 10 minutes, 4°C) and the clear supernatant subsequently stored at -80°C. Virus titers were determined by plaque assay.

d) Screening of antiviral activity in MDK II cells

MDCK II cells were inoculated with the specified viruses at a multiplicity of infection (MOI) of 0.001, resulting in a quantifiable cytopathic effect after 48 hours. Confluent MDCK II cells were washed twice with phosphate-buffered saline (PBS) and inoculated with the virus in the infection medium. Following a 60-minute incubation period, the cells were washed twice with PBS, after which they were treated with the compounds (stock concentration 10 mM, 100 µM in the assay) or the control aprotinin (10 mM stock in water, 100 µM) in infection medium supplemented with 1 µg/mL bovine TPCK-treated trypsin. Following a 48-hour incubation period at 37°C in a 5% CO_2 atmosphere, cell viability was assessed using the CellTiter-Glo assay. Luminescence values were subtracted from the blank (virus-treated cells with corresponding solvent control) and subsequently normalized to the aprotinin control, which was set to 100% antiviral activity.

Prediction of P450-precursor complex

Structural models of LauB1-LauA, LauB1-LauA-Y2F, LauB1-LauA-6S and ScaB-ScaA complexes were generated using AlphaFold 2 in multimer mode with standard parameters.[22] Heme cofactors

were subsequently incorporated using AlphaFill.[23] The resulting structures were visualized in pymol (http://www.pymol.org/).

DNA sequences

The sequence of the *xat* and *ska* BGCs are described below. The start and stop codons are underlined, precursor genes are highlighted in green and cytochrome P450 genes are highlighted in pink.

>*xat* BGC (*Xanthomonas translucens* DSM 18974)

ATGAACGTGCTTTTTTCGATCAAGCAATTGCTGTCTTCCAAGAAGAGCCTGAAATCGTACG
CCTGGTTACACTGGTATTGATCCGCCCCTTGCCATCGATGCGATGTGGTCCGTGCCTGGC
GAATGCCGATGCGCCAGGCACGGGCCTTTGCATTGCGGAGCGCGCGGATGTCCGCCATC
GTGCGTGACAGGCAGGCCCGCGCCACGGTTCTCGCGCCGCGGCTGCAGGCGTTGCTGC
GCGACCATCTGGGCCAGGACGCATTCCGCCTGGATGCCGATACGATCGGTGTCGCCGGG
CCGGCGTTGACCCACCGTTTGCTCGACGCCAGGCCGGCGACGGAATGGGAGCGCCCGAC
GTTCAAGCCGCTGCACGGGCGCTCGATTGCGCGCGCCGATGCGTCGAAGTTGATGCAGG
CGATCGGCCGCGACGTGCGCGAGGCATTGAAGCAGCCAGCTCCCCCGAACGCCGATCTG
TCCGGCCCGTGGCCCCACGTCGGCCATGTCTATCTGCGCGACCTGCTGCTCGGCGACGA
TCCACTGCGGCTGCGCCTGTTGATGGACCGGGTGCTGGAACTGACGCCCAAGCTGACCT
GGGCGGTCATCGCTGCGGGCGCGGTGGCGCCGCTTGCGCCTCAGGCGGGCGCTTCGGC
GCTGGCCACGCTGAGCGCGGCTGCAGGCGGCTACCACGAGCGGCGCTATGCGATGGGC
CTGTACCGGCGCACCGCCGCGCCGGTCTGCTTCACCATCTCCACGCTTGTCGCCAACGC
GCTGTGGCTCGGATCGCCGTTCGATGCCGGCACGTCGAACCGCAATATCCTCTACGAATC
GATGCGCCTGCTACCGCCGTCCTGGAACATCCTGCGCAACGCCTCGCCCGAATACATGGC
GCTGGACGCGCTGATCGGCGCCGCCGACGACGTGCTGGTGCTGCCGCTGCTCAGTCACC
GCGATCCGGCGCTGTGGGACGAACCGCAGGCGTTCCGTCCCGAGCGCTGGGACGGCTT
GGACCCCGACGTCCAGCCCGGCTATCTGCCGTTCGGCCACGCCTCCGAACGCTGCTGGG
GGCGGCACATGGTGATGCCGCTGGCGGAACGCTTGCTCGACCTGCTGCGCGGCCAGGG
CCTGGCCGCCAGTCCGCGGCAGACCCGCGCCGAGGTGCCGCTGGCCGGACTGCTGGGT
GTCGCTCAGGTCGACGTCGTCCGCCACTGA

>*ska* BGC (*Streptomyces kanamyceticus* strains ATCC 12853)

ATGAAGATCGTTCGCTCGCTCAAGAAGAGGATCACCGGAGAGAAGAGCCTCAAGGCGTAC
GCCTGGTACCACTGGTACTAGGCCGGCGACGCGCTCACCGCGCATCCCGCACGCTGA
CGGGGGTGCGCATCCCGTAGGGACGTAACCGCCGTCGAGACCGCGCCCGGCGTGCCCG
ACCAGCCGCCGGGTGCGGGCCTCCCGCACCTGGAGACCCCGATGCCCCCGACGCCCTC
CTCCGGTACCGACCGGCTGTCCAGGACCACCGTCTTCACGCCGCGACTCCATGCCCTGCT
GCGCGACCGCCGCGGTGAGGACGTGTTCCGCCTCGACTCCGACACCGTCGGCGTCGCC
GGGCCCGAGCAGAGCGACCGGATCCTCGCCGCCCGCCCGGCCACCGAACGCGAACGCC
CCACCTTCAAGCCGCTGCACGGGCGTTCGATCCCGCGTACCGAGGCGTCCACCGTCATG
CAGGCCATCGGCCGCGACGTACGCAGGGCGCTGCGCGAACCCGTGCCCGGGAACGTCG
ACCTCTCCGGTGAGTGGCCGCACGTCGGCCACGTCCACCTGCGCGATCTGATCCTGGGC
ACCGACCCGTACCGGCTGCGCATCCTCATGGACCGGACCCTGGAGCTGACGCCCAAACT

CACCTGGTCGGTGATCGCCGTCGGCGCCGCGCTGCCCGGCAGGCTCCGTCCCGGAGCG
CCCGTCACCAGCATCGCGGGCCTCACCGCGGACGCCACGGGCTACAGCGACCGGCGGT
ACGCGATGGGCCTGTACCGGCGGGCCGCGGCCCCGGTCTGCTTCACCGTCTCCACGCTC
GTCGCCAACGCGTACTGGCTGGGGGCGCCGTTCGACGACGAGACGCCGAACGAGCACAT
CCTCTACGAGTCCATGCGGCTGCTGCCGCCGTCCTGGAACATCCTGCGCAACGCGTCCCC
CGAGTACCCGGCCATCGACGACCGCATCGGCGCGGGCGACGACGTCCTGCTGCTGCCGC
TGCTCAGCCACCGGGACCCGGCGCTCTGGAAGGACCCCGACGAGTTCCGCCCCGAACGC
TGGGCCGCCCTCGACCCCGACGACCACCCCGGCTACCTCCCCTTCGGCCACGTCTCCGA
ACGCTGCTGGGGGCGGCACATGGTGATGCCGCTCGCTGGTCAACTCCTCGACATCCTGC
GCGCCGACGGCATGACGGTCGACCCGCGGCAGACCACCGCGAAGGTGCCCCTCGCCGG
GCTCCTCGGGGTCGACAAGGTGCGGCTCGTGCGGTCGGGGCGGGCGTAG

References:

1. C. L. Liu, Z. J. Wang, J. Shi, Z. Y. Yan, G. D. Zhang, R. H. Jiao, R. X. Tan and H. M. Ge, P450-Modified Multicyclic Cyclophane-Containing Ribosomally Synthesized and Post-Translationally Modified Peptides, *Angew. Chem. Int. Ed.*, 2024, **63**, e202314046.
2. D. Hanahan, Studies on transformation of *Escherichia coli* with plasmids, *J. Mol. Biol.*, 1983, **166**, 557-580.
3. D. J. MacNeil, K. M. Gewain, C. L. Ruby, G. Dezeny, P. H. Gibbons and T. MacNeil, Analysis of *Streptomyces avermitilis* genes required for avermectin biosynthesis utilizing a novel integration vector, *Gene*, 1992, **111**, 61-68.
4. T. P. Wyche, A. C. Ruzzini, L. Schwab, C. R. Currie and J. Clardy, Tryptorubin A: A Polycyclic Peptide from a Fungus-Derived Streptomycete, *J. Am. Chem. Soc.*, 2017, **139**, 12899-12902.
5. K. F. Chater and L. C. Wilde, Restriction of a bacteriophage of *Streptomyces albus* G involving endonuclease SalI, *J. Bacteriol.*, 1976, **128**, 644-650.
6. A. Erb, A. Luzhetskyy, U. Hardter and A. Bechthold, Cloning and sequencing of the biosynthetic gene cluster for saquayamycin Z and galtamycin B and the elucidation of the assembly of their saccharide chains, *Chembiochem*, 2009, **10**, 1392-1401.
7. S. Heeb, Y. Itoh, T. Nishijyo, U. Schnider, C. Keel, J. Wade, U. Walsh, F. O'Gara and D. Haas, Small, stable shuttle vectors based on the minimal pVS1 replicon for use in Gram-negative, plant-associated bacteria, *Mol. Plant. Microbe. Interact.*, 2000, **13**, 232-237.
8. F. Biermann, B. Tan, M. Breitenbach, Y. Kakumu, P. Nanudorn, Y. Dimitrova, A. S. Walker, R. Ueoka and E. J. N. Helfrich, Exploration, expansion and definition of the atropopeptide family of ribosomally synthesized and posttranslationally modified peptides, *Chem. Sci.*, 2024, **15**, 17506-17523.
9. A. Schafer, A. Tauch, W. Jager, J. Kalinowski, G. Thierbach and A. Puhler, Small mobilizable multi-purpose cloning vectors derived from the *Escherichia coli* plasmids pK18 and pK19: selection of defined deletions in the chromosome of *Corynebacterium glutamicum*, *Gene*, 1994, **145**, 69-73.
10. S. Pfeilmeier, A. Werz, M. Ote, M. Bortfeld-Miller, P. Kirner, A. Keppler, L. Hemmerle, C. G. Gabelein, G. C. Petti, S. Wolf, C. M. Pestalozzi and J. A. Vorholt, Leaf microbiome dysbiosis triggered by T2SS-dependent enzyme secretion from opportunistic *Xanthomonas* pathogens, *Nat. Microbiol.*, 2024, **9**, 136-149.
11. Y. Bai, D. B. Muller, G. Srinivas, R. Garrido-Oter, E. Potthoff, M. Rott, N. Dombrowski, P. C. Munch, S. Spaepen, M. Remus-Emsermann, B. Huttel, A. C. McHardy, J. A. Vorholt and P. Schulze-Lefert, Functional overlap of the *Arabidopsis* leaf and root microbiota, *Nature*, 2015, **528**, 364-369.
12. X. Wang, D. Zheng and R. Liang, An efficient electro-competent cells generation method of *Xanthomonas campestris* pv. campestris: its application for plasmid transformation and gene replacement, *Adv. Microb.*, 2016, **06**, 79-87.
13. B. Tan, P. Breunig, L. Arbib, Y. Kakumu, F. Biermann, K. Hardes, J. Hefendehl and E. J. N. Helfrich, Phylogeny-guided discovery of a promiscuous P450 macrocyclase for the production of diverse atropopeptides, *Chem. Sci.*, 2025, **16**, 16240-16249.

14. T. Fox and P. A. Kollman, Application of the RESP methodology in the parametrization of organic solvents, *J. Phys. Chem. B.*, 1998, **102**, 8070-8079.
15. M. J. Frisch, G. W. Trucks, H. B. Schlegel, G. E. Scuseria, M. A. Robb, J. R. Cheeseman, G. Scalmani, V. Barone, G. A. Petersson, H. Nakatsuji, X. Li, M. Caricato, A. V. Marenich, J. Bloino, B. G. Janesko, R. Gomperts, B. Mennucci, H. P. Hratchian, J. V. Ortiz, A. F. Izmaylov, J. L. Sonnenberg, Williams, F. Ding, F. Lipparini, F. Egidi, J. Goings, B. Peng, A. Petrone, T. Henderson, D. Ranasinghe, V. G. Zakrzewski, J. Gao, N. Rega, G. Zheng, W. Liang, M. Hada, M. Ehara, K. Toyota, R. Fukuda, J. Hasegawa, M. Ishida, T. Nakajima, Y. Honda, O. Kitao, H. Nakai, T. Vreven, K. Throssell, J. A. Montgomery Jr., J. E. Peralta, F. Ogliaro, M. J. Bearpark, J. J. Heyd, E. N. Brothers, K. N. Kudin, V. N. Staroverov, T. A. Keith, R. Kobayashi, J. Normand, K. Raghavachari, A. P. Rendell, J. C. Burant, S. S. Iyengar, J. Tomasi, M. Cossi, J. M. Millam, M. Klene, C. Adamo, R. Cammi, J. W. Ochterski, R. L. Martin, K. Morokuma, O. Farkas, J. B. Foresman and D. J. Fox, Gaussian 16 Rev. A.03, Wallingford, CT, 2016
16. J. D. Chai and M. Head-Gordon, Long-range corrected hybrid density functionals with damped atom-atom dispersion corrections, *Phys. Chem. Chem. Phys.*, 2008, **10**, 6615-6620.
17. F. Weigend and R. Ahlrichs, Balanced basis sets of split valence, triple zeta valence and quadruple zeta valence quality for H to Rn: Design and assessment of accuracy, *Phys. Chem. Chem. Phys.*, 2005, **7**, 3297-3305.
18. A. V. Marenich, C. J. Cramer and D. G. Truhlar, Universal solvation model based on solute electron density and on a continuum model of the solvent defined by the bulk dielectric constant and atomic surface tensions, *J. Phys. Chem. B.*, 2009, **113**, 6378-6396.
19. G. Luchini, J. Alegre-Requena, I. Funes-Ardoiz and R. Paton, GoodVibes: automated thermochemistry for heterogeneous computational chemistry data, *F1000Research*, 2020, **9**.
20. S. Grimme, Supramolecular Binding Thermodynamics by Dispersion-Corrected Density Functional Theory, *Chem-Eur. J.*, **2012**, 18, 9955-9964.
21. E. M. Y. Lee, T. Ludwig, B. Yu, A. R. Singh, F. Gygi, J. K. Norskov and J. J. de Pablo, Neural Network Sampling of the Free Energy Landscape for Nitrogen Dissociation on Ruthenium, *J. Phys. Chem. Lett.*, 2021, **12**, 2954-2962.
22. J. Jumper, R. Evans, A. Pritzel, T. Green, M. Figurnov, O. Ronneberger, K. Tunyasuvunakool, R. Bates, A. Zidek, A. Potapenko, A. Bridgland, C. Meyer, S. A. A. Kohl, A. J. Ballard, A. Cowie, B. Romera-Paredes, S. Nikolov, R. Jain, J. Adler, T. Back, S. Petersen, D. Reiman, E. Clancy, M. Zielinski, M. Steinegger, M. Pacholska, T. Berghammer, S. Bodenstein, D. Silver, O. Vinyals, A. W. Senior, K. Kavukcuoglu, P. Kohli and D. Hassabis, Highly accurate protein structure prediction with AlphaFold, *Nature*, 2021, **596**, 583-589.
23. M. L. Hekkelman, I. de Vries, R. P. Joosten and A. Perrakis, AlphaFill: enriching AlphaFold models with ligands and cofactors, *Nat. Methods*, 2023, **20**, 205-213.
